# Fish oil and inflammatory status alter the n-3 to n-6 balance of the endocannabinoid and oxylipin metabolomes in mouse plasma and tissues

**DOI:** 10.1007/s11306-012-0421-9

**Published:** 2012-04-11

**Authors:** Michiel G. J. Balvers, Kitty C. M. Verhoeckx, Sabina Bijlsma, Carina M. Rubingh, Jocelijn Meijerink, Heleen M. Wortelboer, Renger F. Witkamp

**Affiliations:** 1 Division of Human Nutrition, Wageningen University, PO Box 8129, 6700 EV Wageningen, The Netherlands; 2 TNO, PO Box 370, 3700 AJ Zeist, The Netherlands

**Keywords:** Endocannabinoids, Oxylipins, Fish oil, Inflammation, Lipidomics, Multivariate data analysis

## Abstract

It is well established that dietary intake of n-3 fatty acids is associated with anti-inflammatory effects, and this has been linked to modulation of the oxylipin and endocannabinoid metabolomes. However, the amount of data on specific tissue effects is limited, and it is not known how inflammation affects this relation. In the present study we systematically explored the combined effects of n-3 fatty acid diets and inflammation on the in vivo endocannabinoid and oxylipin metabolomes using a multicompartment, detailed targeted lipidomics approach. Male C57BL/6 mice received diets containing 0, 1, or 3 % w/w fish oil (FO) for 6 weeks, after which 2 mg/kg LPS or saline was administered i.p. Levels of endocannabinoids/*N*-acylethanolamines (NAEs) and oxylipins, covering n-3 and n-6 fatty acid derived compounds, were determined in plasma, liver, ileum and adipose tissue using LC–MS/MS. FO generally increased ‘n-3’ NAEs and oxylipins at the expense of compounds derived from other fatty acids, affecting all branches of the oxylipin metabolome. LPS generally increased levels of endocannabinoids/NAEs and oxylipins, with opposing effects across plasma and tissues. Multivariate data analysis revealed that separation between diet groups in the saline treated groups was primarily explained by decreases in other than n-3 derived compounds. In the LPS treated groups, the separation was primarily explained by increases in n-3 derived compounds. In conclusion, FO caused marked changes in the n-3 to n-6 balance of the endocannabinoid and oxylipin metabolomes, with specific effects depending on inflammatory status.

**Electronic supplementary material:**

The online version of this article (doi:10.1007/s11306-012-0421-9) contains supplementary material, which is available to authorized users.

## Introduction

**Fig. 1:**
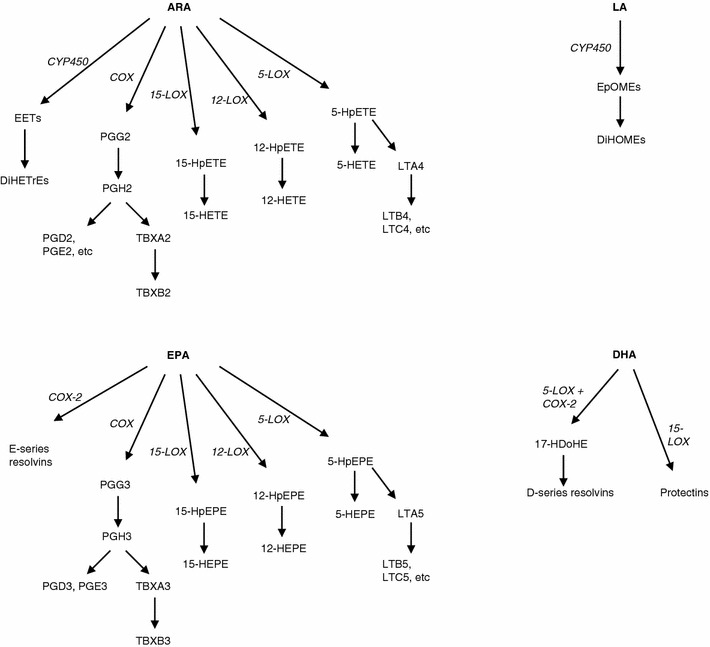
Overview of several enzymatic pathways involved in oxylipin synthesis, which are under investigation in the present paper. ARA, LA, EPA and DHA can serve as substrates, yielding distinct oxylipins and intermediates

Dietary intake of long-chain n-3 polyunsaturated fatty acids (PUFAs), like docosahexaenoic acid (DHA; 22:6 n-3) and eicosapentaenoic acid (EPA; 20:5 n-3), is known to have beneficial health effects in both humans and animals, which are partly explained by a reduction of inflammatory processes (Calder 2006, 2009a; Carpentier et al. 2006; Schmitz and Ecker 2008). The mechanisms behind this are not completely understood, but involve binding of n-3 PUFAs to GPR120 (Oh et al. 2010), their conversion to resolvins (Serhan et al. 2004), and the alteration of the eicosanoid balance (Calder 2009a). Increased dietary intake of n-3 PUFAs leads to enhanced incorporation of DHA and EPA in cell membranes, at the expense of incorporation of the n-6 PUFA arachidonic acid (ARA; 20:4 n-6). This results in decreased synthesis of ARA-derived eicosanoids, for example PGE_2_, after e.g. an inflammatory stimulus (Calder 2009b). At the same time, increased levels of n-3 fatty acid derived eicosanoids are observed. These n-3 fatty acid derived metabolites are often referred to as ‘3-series’ or ‘5-series’ oxylipins, comprising structures like prostaglandin D_3_ (PGD_3_), PGE_3_, thromboxane B_3_ (TBXB_3_), and 5-hydroxyeicosapentaenoic acid (5-HEPE), or leukotriene B_5_ (LTB_5_), respectively (see Fig. 1 for an overview of oxylipins and their origin). These compounds are in general also pro-inflammatory, but considered less potent than the ARA-derived metabolites under certain circumstances, thereby contributing to a reduction of the general inflammatory status and specific inflammatory processes associated with fish oil (FO) consumption (Calder 2006, 2009a; Schmitz and Ecker 2008).

Over the last decades, several endocannabinoids and related *N*-acyl ethanolamines (NAEs) have emerged as important regulators of metabolism and inflammation (De Petrocellis et al. 2000; Di Marzo 2008; Matias et al. 2006; O’Sullivan 2007). Like the oxylipins, these compounds are also derived from fatty acids following incorporation in cell membranes (Bisogno 2008; Ueda et al. 2010). Arachidonoyl ethanolamide (anandamide, AEA) and 2-arachidonoyl glycerol (2-AG) are two endocannabinoids which are derived from ARA, but combinations derived with other fatty acids also exist, such as palmitoyl ethanolamide (PEA) and the n-3 fatty acid derived NAEs docosahexaenoyl ethanolamide (DHEA), eicosapentaenoyl ethanolamide (EPEA). Both AEA and PEA are known for their anti-inflammatory properties (Cencioni et al. 2010; Re et al. 2007).

Several in vitro and animal studies have demonstrated a link between availability of specific fatty acids in the diet and the presence of endocannabinoids and related NAEs. Berger and coworkers reported enhanced levels of anandamide and 2-AG in piglet brain after feeding milk supplemented with ARA, with a diet rich in DHA showing even higher levels of its NAE metabolite, DHEA (Berger et al. 2001). Wood and coworkers showed that a two-week diet rich in DHA elevated plasma and brain levels of DHEA in mice, while decreasing plasma 2-AG (Wood et al. 2010). Artmann and coworkers demonstrated that feeding rats a FO diet, by nature rich in n-3 PUFAs, decreased jejunal levels of AEA and PEA, but increased the levels of n-3 NAEs DHEA and EPEA (Artmann et al. 2008). Fish oil also decreased adipose tissue levels of AEA and 2-AG in a rat model of obesity (Batetta et al. 2009). It thus seems that the profile of NAEs represents the relative abundance of fatty acids in the diet. Recently, is was shown that DHEA and EPEA display anti-inflammatory properties in macrophages and adipocytes (Balvers et al. 2010; Meijerink et al. 2011), indicating that these compounds might be involved in the anti-inflammatory effects which are related to dietary n-3 PUFA intake.

In addition to diet, inflammation is known to affect the synthesis and/or release of both oxylipins and NAEs (Maccarrone et al. 2001), but it is not known how inflammation itself affects e.g. DHEA and EPEA tissue levels in vivo. Moreover, it is not known if changes induced by dietary fatty acids also persist under inflammatory conditions, or if the effect of diet is different under inflammatory conditions.

In the present study, we systematically explored in detail the (combined) effect of dietary FO and inflammation on levels of endocannabinoids/NAEs and oxylipins in plasma, liver, ileum and adipose tissue of wild type C57BL/6 mice using a targeted lipidomic approach. In total, levels on 61 compounds were analyzed, including levels of PGE_3_, PGD_3_, TBX-B_3_, 5-HEPE, resolvin D_1_, DHEA and EPEA. Both univariate and multivariate data analysis tools were used to assess differences in metabolite patterns between the intervention groups. Our data show in detail that dietary intake of FO shifted the n-3 to n-6 balance in the endocannabinoid and oxylipin metabolomes in all tissues examined. In addition, the direction of this shift appeared to be affected by inflammation, and was different between the examined tissues.

## Materials and methods

### Chemicals and reagents

Lipopolysaccharide (0111: B4; LPS), indomethacin, paraoxon and butylated hydroxytoluene (BHT) were from Sigma (Steinheim, Germany). Phenylmethylsulfonyl fluoride (PMSF) was from Fluka (Steinheim, Germany). 12-[(tricyclo[3.3.1.13,7]dec-1-ylamino)carbonyl]amino]-dodecanoic acid (AUDA) and URB602 was purchased from Cayman (Ann Arbor, MI, USA). Milli-Q water (Milli-Q Advantage unit, Millipore, Amsterdam, The Netherlands) was used in all analyses. ULC-grade acetonitrile (ACN), formic acid (FA) and trifluoro acetic acid (TFA) were obtained from Biosolve (Valkenswaard, The Netherlands). LC–MS grade methanol was from Riedel-de-Häen (Steinheim, Germany). Isopropanol and ethanol were from JT Baker (Deventer, The Netherlands). All analytical and internal standards, except EPEA, were purchased from Cayman. EPEA was synthesized as described earlier (Plastina et al. 2009a). For oxylipins, stock solutions were prepared in ethanol, aliquoted and stored at −80 °C until analysis. For endocannabinoids/NAEs, stocks were prepared in ACN, aliquoted and stored at −80 °C until analysis. C8 SPE columns (Bond Elut; 200 mg, 3 mL) were from Varian Inc. (Lake Forest, CA, USA). HLB SPE columns (Oasis, 60 mg, 3 mL) were from Waters (Etten-Leur, The Netherlands). ELISA kits were from R&D Systems (Minneapolis, MN, USA).

### Animal experiment

Wild type male C57BL/6 mice were obtained from Harlan (Horst, The Netherlands) and housed two or three per cage in a temperature controlled environment with a 12 h light–dark cycle (light at 6.00–18.00). The mice, 4 weeks old at arrival, had free access to a standard run-in diet (AIN93-M, with a 4 % w/w fat content whereof 1 % soy bean oil and 3 % high-oleic acid sunflower oil (HOSF)) for 2 weeks. At the age of 6 weeks the mice were divided into three groups of 16 mice; group 1 was kept on the standard diet (control diet), group 2 received a diet containing AIN93-M with 1 % fish oil (1 % FO diet) (Marinol^®^), 2 % HOSF, and 1 % soy bean oil. The third group had access to a diet containing AIN93-M with 1 % soy bean oil and 3 % fish oil (3 % FO diet). The diets and water were available ad libitum. Diets were prepared by Research Diet Services (Wijk bij Duurstede, The Netherlands) and the Marinol^®^ was a kind gift from Lipid Nutrition (Wormerveer, The Netherlands). Diets were stored in air-tight bags at −20 °C until just before feeding, and fresh food was provided two times per week to minimize oxidation of the fatty acids in the diet. GC–MS based analysis of the diets confirmed that the correct amounts of DHA and EPA were present, and re-analysis after 4 weeks revealed that its amounts were stable under the described conditions (data not shown). Food consumption and animal weight were measured two times per week, revealing no differences between the diet groups.

The diets were continued for 6 weeks, after which the animals received either i.p. saline (eight mice per diet group) or 2 mg/kg LPS (eight mice per diet group). After 24 h, the animals were anesthetized, blood was collected from the orbital sinus and captured in 1.3 mL EDTA coated tubes (Sarstedt; Etten-Leur, The Netherlands) and put on ice until centrifugation (10′, 10,000 rpm at 4 °C). After centrifugation, plasma was aliquoted. For oxylipin analysis, 200 μL plasma was stored in 1 mL methanol containing paraoxon, BHT, AUDA, indomethacin, and PMSF to prevent oxylipin oxidation and breakdown. For endocannabinoid/NAE analysis, 100 μL plasma was stored in the presence of PMSF and URB602. Subsequently, the animals were sacrificed by cervical dislocation after which liver, ileum and adipose tissue were collected and immediately snap-frozen in liquid nitrogen. All plasma and tissue samples were stored at −80 °C until further analysis. Analysis of plasma IL-6 and MCP-1 levels confirmed that LPS had triggered an inflammatory response by showing strongly increased IL-6 and MCP-1 levels in LPS-treated mice (data not shown).

The study was conducted according to the Netherlands Law on Animal Experiments, and approved by the local Animal Experiments Committee of Wageningen University.

### Extraction of endocannabinoids/NAEs from plasma

Plasma (100 μL) was thawed and 400 μL extraction mixture containing 100 μM PMSF and internal standards (AEA-d8, 2-AG-d8 and OEA-d4) in ACN was added while the sample was gently vortexed. After subsequent centrifugation (5 min at 13,000 rpm and RT), the supernatant was transferred to a clean Eppendorf tube and evaporated to dryness in a vacuum concentrator (Scanvac; Lynge, Denmark). The dried extracts were reconstituted in 100 μL ACN containing 0.1 % TFA and used for LC–MS/MS analysis.

### Extraction of endocannabinoids/NAEs from tissues

Endocannabinoid/NAE were extracted from freeze–dried liver and ileum using a method adapted from a previously published protocol for plasma (Balvers et al. 2009). Approximately 50 mg freeze–dried liver or 10 mg freeze–dried ileum were extracted by adding 1 mL extraction mixture (ACN) and sonication. The samples were centrifuged (5 min at 14,000 rpm), the supernatant was transferred to a clean 15 mL tube, and this was repeated once. The pooled ACN fractions were diluted with MQ water containing 0.13 % TFA until the final ACN concentration was 20 % prior to SPE clean-up as described before (Balvers et al. 2009). In short, columns were washed with 20 % v/v ACN in MQ water containing 0.1 % TFA, eluted with 80 % v/v ACN in MQ water containing 0.1 % TFA and evaporated to dryness using vacuum centrifugation. The dried extracts were reconstituted in 100 μL ACN containing 0.1 % TFA and used for LC–MS/MS analysis.

For adipose tissue, approximately 100 mg ‘wet’ tissue was extracted with 1 mL extraction solution (ACN) by sonication. The samples were centrifuged for 5 min at 14,000 rpm and RT, the supernatant was transferred to a clean 2.0 mL Eppendorf tube, and the ACN extraction was repeated once. The 2 mL ACN extract was subsequently evaporated to dryness, reconstituted in 100 μL ACN containing 0.1 % TFA and used for LC–MS/MS analysis.

### LC–MS/MS analysis of endocannabinoids/NAEs

Two LC–MS/MS systems were used for endocannabinoid/NAE analysis. Plasma extracts were analyzed by UPLC coupled to a Xevo TQ-S mass spectrometer (Waters; Etten-Leur, The Netherlands) because high sensitivity was essential for adequate quantification in extracts obtained from 100 μL plasma samples. Liver, ileum and adipose tissue were analyzed on a Surveyor HPLC coupled to a TSQ Quantum Discovery mass spectrometer (Thermo Finnigan; Breda, The Netherlands).

For the UPLC-Xevo system, 3 μL plasma extract was injected on a Acquity C8 BEH UPLC column (2.1 × 100 mm, 1.7 μm) and was separated using gradient elution with a stable flow of 500 μL/min. The gradient started with 100 % A (40:40:20 v/v/v of MQ water:methanol:ACN with 0.1 % FA) which was maintained until 0.35 min, followed by a linear increase to 100 % B (7:3 v/v methanol:ACN with 0.1 % FA) which was achieved at 7.0 min and was maintained until 9.0 min. Finally, the column equilibrated for 3 min at 100 % A. The column was maintained at 60 °C during analysis, and the samples were kept at 10 °C. The MS was operating in selective reaction mode using electrospray ionization in positive ion mode, with a capillary voltage of 1.5 kV, a source temperature of 150 °C and a desolvation temperature of 500 °C. Cone voltage and collision energy were optimized for each compound individually (see supplemental data S-1 for parent and product *m/z* values). Peak identification and quantification was performed using MassLynx software version 4.1. Calibration curves were run in duplicate from which one regression equation was generated.

For the analysis of liver, ileum and adipose tissue, a TSQ Quantum Discovery was used as described before (Balvers et al. 2009). Five microliters extract was separated on an Xterra C8 MS column (2.1 × 150 mm, 3.5 μm) using gradient elution with a constant flow of 150 μL/min. The same solutions were used as in the Xevo system, but now 1 g/L ammonium acetate was added (the most dominant parent for 2-AG in this MS is the ammonium adduct). The gradient started with 100 % A which was maintained until 2.0 min, followed by a linear increase to 100 % B which was achieved at 8.00 min and maintained until 16.0 min, and the column was left to equilibrate for 5 min at 100 % A. The column was maintained at 40 °C during analysis and the samples were cooled at 4 °C. The MS was operating in selective reaction mode using electrospray ionization in positive ion mode, with a capillary voltage of 4.5 kV and a capillary temperature of 350 °C. Cone voltage and collision energy were optimized for each compound individually. Peak identification and quantification was performed using LCquan software version 2.5.5. Calibration curves were run in duplicate from which one regression equation was generated. Quality control samples were included in each analytical run to check the quality of the analysis and to correct for accuracy.

### Extraction of oxylipins from plasma

Internal standards were added to the plasma samples which were already precipitated with methanol (see section ‘2.2’), and the samples were put on ice for 30 min. Samples were subsequently centrifuged (5 min at 3,000×*g* and 4 °C) and the supernatant was transferred to a glass tube. Just before loading on activated HLB columns, 4.75 mL MQ water containing 0.1 % v/v FA was added to the methanol extract, diluting the extract to 20 % methanol. After loading, the columns were washed with 2 mL 20 % methanol in MQ water containing 0.1 % FA, and the columns were allowed to dry for 15 min. The SPE columns were eluted with 2 mL methanol and the samples were captured in tubes already containing 20 μL of 10 % glycerol and 500 μM BHT in ethanol. The tubes were placed in a water bath at 40 °C and the methanol was evaporated under a gentle stream of nitrogen, after which the samples were reconstituted in 100 μL ethanol containing another internal standard (CUDA) and immediately used for LC–MS/MS analysis.

### Extraction of oxylipins from tissues

The extraction of oxylipins from liver, ileum and adipose tissue was similar to plasma oxylipin extraction. Approximately 100 mg liver and adipose tissue, and 50 mg ileum was extracted with 1 mL methanol containing internal standards and sonication. After centrifugation (5 min at 3,000×*g* and 4 °C), the supernatants were transferred to clean tubes and the methanol extraction was repeated once. Just before loading on HLB SPE columns, 8 mL MQ water containing 0.1 % FA was added to the methanol extracts. For the SPE procedure and further, (see section ‘2.6’) Extraction of oxylipins from plasma’.

### LC–MS/MS analysis of oxylipins

All oxylipin analyses were performed on a UPLC coupled to a Xevo TQ-S mass spectrometer (Waters). Five microliters extract was injected on a Acquity C18 BEH UPLC column (2.1 × 100 mm, 1.7 μm) and was separated using gradient elution with a stable flow of 600 μL/min. The gradient started with 95 % A (MQ water with 0.1 % FA) and 5 % B (ACN with 0.1 % FA) followed by a linear increase to 70 % A and 30 % B which was achieved at 5.0 min. This was followed by a linear increase towards 50 % B which was achieved at 11.25 min and maintained until 13.25 min. The system was subsequently switched to 100 % B, which was achieved at 15.75 min and maintained until 16.75 min, after which the column was left to equilibrate at 5 % B for approximately 3 min. The column was maintained at 50 °C during analysis, and the samples were kept at 10 °C. The MS was operating in selective reaction mode using electrospray ionization in negative ion mode, with a capillary voltage of 3.3 kV, a source temperature of 150 °C and a desolvation temperature of 600 °C. Cone voltage and collision energy were optimized for each compound individually (see supplemental data S-1 for parent and product *m/z* values). Peak identification and quantification was performed using MassLynx software version 4.1. Calibration curves were run in duplicate from which one regression equation was generated. During data analysis, five peaks of unknown identity were found to be influenced by diet or LPS treatment, and these compounds are listed UK1–UK5. These peaks were visible in the transitions *m/*z 295.2 > 195.2 and *m/z* 295.2 > 171.1. ARA, DHA and EPA were also determined using this method. Quality control samples were included in each analytical run to check the quality of the analysis and to correct for accuracy.

### Data analysis

Univariate analysis was performed with SAS version 9.1 (2002–2003 by SAS Institute Inc., Cary, NC, USA). ANOVA assumptions were checked for each variable. If these assumptions were not met, rank transformation was applied for that particular variable. Partial tests were performed using Tukey–Kramer multiple comparison correction. Benjamini and Hochberg false discovery rate correction (*q* = 5 %) was applied to correct for false positives (Benjamini and Hochberg 1995). In all statistical tests that were performed, the null hypothesis (no effect) was rejected at the 0.05 level of probability (α = 5 %).

The added value of multivariate data analysis in addition to univariate statistics is that correlations between variables are taken into account, and thus also allows to reveal combinations of variables which are associated with differences between treatment groups. Multivariate data analysis summarizes all the variables into one variable by means of a linear combination, now called the ‘principal component’ (PC), which adds higher weights to variables that account for the highest level of variance in the original data. Using principal component analysis (PCA), we screened for group separation, outliers, (undesired) patterns and this was further analyzed with principal component discriminant analysis (PCDA). PCDA includes the original group designation of the animals in the model and is therefore called a supervised classification technique. PCA and PCDA were performed in the Matlab environment (R2008b, 1984–2008, The Mathworks Inc., Natick, MA, USA) using the PLS toolbox for Matlab version 5.0.3 (r 6466, 1995–2008, Eigenvector Research Inc., Wenatchee, WA, USA). PCA and PCDA are described in more detail elsewhere (Hoogerbrugge et al. 1983; Joliffe 1986). For all multivariate models data were autoscaled to mean zero and variance 1 for each variable. For PCDA, stability of the model was evaluated by 10-fold cross-validation, revealing correct classification rates of typically 80–100 %. PCA and PCDA were performed on the combined data (‘fused data’), containing data on both endocannabinoids/NAEs and oxylipins from plasma, liver ileum and adipose tissue combined in one data set.

## Results

### FO diet and inflammation alter the endocannabinoid/NAE balance

To investigate the effect of dietary n-3 fatty acids and inflammation on endocannabinoid/NAE and oxylipin levels, wild-type male C57BL/6 mice received a diet containing either no, 1 or 3 % w/w FO followed by either saline or 2 mg/kg LPS i.p. injection. Endocannabinoid/NAE levels were determined in plasma, liver, ileum and adipose tissue.

Detailed (quantitative) effects of dietary administration of fish-oil and administering LPS after 6 weeks compared to their relevant control treatments are provided in the supplemental data (S2-7), including some representative chromatograms (S-8). Significant differences between diet groups and LPS treatment were obtained with the ANOVA test and are summarized in Tables 1, 2 for endocannabinoids/NAEs and Tables 3, 4 for oxylipins. A *diet effect* is here defined as an effect of the diet which (in magnitude and direction) was the same for saline and LPS-treated mice. The term *LPS effect* refers to situations in which LPS induced a change in a concentration of a compound, which was similar for all diet groups. An *interaction effect* indicates that only certain (combinations of) diets with saline or LPS resulted in significant differences, and therefore separate comparisons (‘partial tests’) should be interpreted rather than main effects. Table 1 shows diet effects on NAEs/endocannabinoids, and 2 LPS effects. Compounds with an interaction effect are highlighted with * in the tables, with further details provided in the supplemental data (S-5).

**Table 1:** Effect of the fish oil diets on endocannabinoid/NAE levels in plasma, liver, ileum and adipose tissue (diet effect)

			Plasma	Liver	Ileum	Adi. tiss.
Ctrl vs 1 % FO	n-3 derived	EPEA	2.51	*	24.159	43.096
		DHEA	1.915	2.908	2.123	3.241
	Other	AEA	*	0.312	0.374	0.350
		2-AG	0.403	0.216	0.304	0.465
		DGLEA	0.282	–	0.650	0.562
		OEA	0.710	0.777	–	*
Ctrl vs 3 % FO	n-3 derived	EPEA	3.688	*	67.069	116.975
		DHEA	2.166	4.691	2.774	5.484
	Other	AEA	*	–	0.811	0.423
		2-AG	0.301	0.156	0.272	0.607
		DGLEA	0.250	–	↓	0.578
		OEA	0.522	0.663	–	*
		SEA	0.692	–	–	1.574
1 % FO vs 3 % FO	n-3 derived	EPEA	–	*	2.776	2.714
		DHEA	–	1.613	1.306	1.692
	Other	2-AG	–	0.724	–	1.304
		OEA	0.735	–	–	*
		SEA	0.700	–	–	1.284

Only statistically significant effects are listed, presented as fold-control values, calculated by comparing mean metabolite concentration from one diet to another. Means were calculated from saline and LPS treated animals together per diet group. ↑ or ↓ were used when the fold-control value would disagree with the outcome outcome of the ANOVA in cases of variables which were rank-transformed

*ND* the compound was not detected in the particular matrix, and *–* indicates that no statistical significant differences were observed

*^ ^Interaction effect (see supplemental data for details)

**Table 2:** Effect of LPS on endocannabinoid/NAE levels in plasma, liver, ileum and adipose tissue (LPS effect)

			Plasma	Liver	Ileum	Adi. tiss.
Saline vs LPS	n-3 derived	EPEA	2.019	*	–	1.766
		DHEA	2.101	4.130	1.338	1.584
	Other	AEA	*	3.375	1.274	–
		2-AG	0.651	–	–	1.366
		DGLEA	1.961	1.611	↑	–
		PEA	*	0.743	1.406	*
		OEA	3.097	2.276	1.260	*
		SEA	2.073	0.540	1.676	0.711

Only statistically significant effects are listed, presented as fold-control values, calculated by comparing mean metabolite concentration from the saline to the LPS treated groups. Means were calculated from the different diet groups together for the saline and LPS treated animals. ↑ or ↓ were used when the fold-control value would disagree with the outcome outcome of the ANOVA in cases of variables which were rank-transformed

*ND* the compound was not detected in the particular matrix, and *–* no statistical significant differences were observed

* Interaction effect (see supplemental data for details)

**Table 3:** Effect of the fish oil diets on oxylipin levels in plasma, liver, ileum and adipose tissue (diet effect)

	Plasma	Liver	Ileum	Adi. tiss.
Ctrl vs 1% FO				
Fatty acids				
ARA	*	0.326	–	0.520
DHA	1.931	–	2.128	–
EPA	*	13.93	24.182	29.425
n-3 derived oxylipins				
5-HEPE	4.358	7.474	9.459	34.105
12-HEPE	*	4.753	19.379	24.735
PGD_3_	ND	ND	18.828	3.937
PGE_3_	–	ND	15.534	12.614
17-HDoHE	–	–	3.014	2.694
10-17-DiHDoHE	ND	ND	2.566	3.352
19,20-DiHoPE	3.040	1.534	2.381	4.447
TBXB_3_	*	ND	20.657	4.809
n-6 oxylipins				
5,6 EET	–	0.163	–	–
11,12 EET	–	0.293	0.495	0.484
14,15 EET	0.492	0.319	0.476	0.497
LTB_4_	ND	3.356	0.470	*
LTD_4_	ND	ND	0.184	–
5,6-DiHETrE	–	0.215	0.392	0.383
8,9-DiHETrE	0.314	0.213	0.378	*
11,12-DiHETrE	0.280	0.249	0.419	0.378
14,15-DiHETrE	0.327	0.251	0.404	*
PGE_2_	–	0.205	–	–
PGF_2α_	–	0.234	–	–
8-iso-PGF_2α_	–	–	–	0.629
13,14-dihydro-15-keto-PGD_2_	ND	ND	0.292	ND
13,14-dihydro-15-keto-PGE_2_	↓	0.373	0.242	0.198
13,14-dihydro-15-keto-PGF_2α_	ND	0.432	0.255	0.473
12-HHTrE	–	0.107	–	0.298
5-HETE	0.416	0.410	–	0.632
11-HETE	–	0.233	–	0.417
12-HETE	–	0.150	–	–
15-HETE	–	0.211	–	0.252
20-HETE	ND	0.419	–	ND
TBXB_2_	–	0.209	–	–
13-HODE	–	0.570	–	–
9,10,13-TriHOME	–	*	–	0.686
Ctrl vs 3% FO				
Fatty acids				
ARA	*	0.302	–	–
DHA	1.642	–	2.828	–
EPA	*	19.597	37.806	54.972
17 keto- 4(z), 7(z), 10(z), 13 (z), 15 (E), 19(z)-DHA	ND	–	2.830	*
n-3 derived oxylipins				
5-HEPE	7.430	12.323	19.378	163.982
12-HEPE	*	7.684	33.107	55.445
PGD_3_	ND	ND	14.818	9.352
PGE_3_	–	ND	23.562	32.107
17-HDoHE	–	2.051	3.362	3.148
10-17-DiHDoHE	ND	ND	3.384	4.768
19,20-DiHoPE	5.717	2.244	4.172	18.053
TBXB_3_	*	ND	16.198	10.438
Other oxylipins				
5,6 EET	–	0.120	–	–
8,9 EET	ND	ND	0.329	–
11,12 EET	0.512	0.179	0.489	0.566
14,15 EET	0.492	0.277	0.537	–
LTB_4_	ND	3.524	0.357	*
LTD_4_	ND	ND	0.184	–
n-acetyl-leukotriene E_4_	ND	0.628	*	ND
5,6-DiHETrE	0.358	–	0.246	0.496
8,9-DiHETrE	0.481	0.186	0.328	*
11,12-DiHETrE	0.303	0.221	0.388	0.593
14,15-DiHETrE	0.562	0.224	0.366	*
PGD_2_	0.363	*	–	–
PGE_2_	0.397	0.229	–	–
PGF_2α_	–	0.259	–	–
8-iso-PGF_2α_	–	–	–	0.623
13,14-dihydro-15-keto-PGD_2_	ND	ND	0.180	ND
13,14-dihydro-15-keto-PGE_2_	0.479	0.275	0.157	0.244
13,14-dihydro-15-keto-PGF_2α_	ND	0.240	0.200	0.405
12-HHTrE	0.421	0.094	–	0.224
5-HETE	0.375	–	–	–
11-HETE	0.401	0.094	–	–
12-HETE	0.265	0.159	–	0.289
15-HETE	0.323	0.250	–	0.234
20-HETE	ND	0.447	–	ND
TBXB_2_	0.403	0.215	–	–
9-HODE	–	–	–	0.417
13-HODE	0.559	↓	–	0.308
lipoxin A_4_	ND	1.771	2.533	7.181
1% FO vs 3% FO				
Fatty acids				
DHA	–	–	1.329	–
EPA	*	–	1.563	1.868
17 keto- 4(z), 7(z), 10(z), 13 (z), 15 (E), 19(z)-DHA	ND	–	1.786	*
n-3 derived oxylipins				
5-HEPE	–	1.649	2.049	4.808
12-HEPE	*	–	1.708	2.242
PGD_3_	ND	ND	–	2.545
PGE_3_	–	ND	–	2.375
17-HDoHE	–	1.435	–	–
19,20-DiHoPE	–	1.463	-	4.060
Other oxylipins				
5-HETE	–	–	0.416	1.739
11-HETE	0.487	–	–	–
13-HODE	0.651	–	–	–
15-HETE	0.442	–	–	–
PGE_2_	0.492	–	–	–
13,14-dihydro-15-keto-PGE_2_	–	–	0.648	–
13,14-dihydro-15-keto-PGF_2α_	ND	0.555	–	–
lipoxin A_4_	ND	2.075	2.877	6.588
TBXB_2_	0.433	–	–	–

Only statistically significant effects are listed, presented as fold-control values, calculated by comparing mean metabolite concentration from one diet to another. Means were calculated from saline and LPS treated animals together per diet group. ↑ or ↓ were used when the fold-control value would disagree with the outcome outcome of the ANOVA in cases of variables which were rank-transformed

*ND* the compound was not detected in the particular matrix, and ‘*–*’ indicates that no statistical significant differences were observed

* Interaction effect (see supplemental data for details)

**Table 4:** Effect of LPS on oxylipin levels in plasma, liver, ileum and adipose tissue (LPS effect)

			Plasma	Liver	Ileum	Adi. tiss.
Saline vs LPS	Fatty acids	ARA	*	1.827	–	1.854
		DHA	2.033	1.590	–	–
		EPA	*	–	–	1.791
	n-3 derived oxylipins	5-HEPE	2.049	–	–	5.626
		PGD_3_	ND	ND	–	2.498
		PGE_3_	–	ND	3.256	4.217
		10(S)-17(S)-DiHDoHE	ND	ND	–	2.249
		19,20-DiHoPE	3.886	1.376	1.874	3.822
		TBXB_3_	*	ND	2.637	4.752
	Other oxylipins	5,6 EET	–	–	–	2.847
		11,12 EET	–	–	–	1.843
		14,15 EET	–	1.890	–	–
		LTB_4_	ND	0.538	–	*
		LTD_4_	ND	ND	–	2.445
		n-acetyl leukotriene E_4_	ND	1.627	*	ND
		5,6 DiHETrE	–	–	–	1.744
		8,9-DiHETrE	2.170	–	–	*
		11,12-DiHETrE	2.167	–	–	–
		14,15-DiHETrE	1.943	–	–	*
		PGE_2_	1.958	2.449	–	2.039
		PGF_2α_	–	–	–	1.777
		8-iso-PGF_2α_	–	1.778	–	–
		13,14-dihydro-15-keto-PGE_2_	2.600	–	–	1.243
		13,14-dihydro-15-keto-PGF_2α_	ND	1.815	–	–
		12-HHTrE	0.201	–	–	2.252
		5-HETE	2.240	–	–	2.435
		11-HETE	0.561	1.887	–	1.925
		12-HETE	–	0.782	–	–
		15-HETE	0.433	–	–	–
		20-HETE	ND	2.399	–	ND
		TBXB_2_	0.175	–	2.560	2.277
		9-HODE	1.267	–	2.768	2.014
		13-HODE	–	–	2.086	1.206
		9,10,13-TriHOME	1.301	*	–	–
		Lipoxin A_4_	ND	–	–	2.372

Only statistically significant effects are listed, presented as fold-control values, calculated by comparing mean metabolite concentration from the saline to the LPS groups. Means were calculated from the different diet groups together for saline and LPS treated animals.

*ND* the compound was not detected in the particular matrix, and *–* indicates that no statistical significant differences were observed

* Interaction effect (see supplemental data for details)

The FO diets altered endocannabinoid levels with different effects in plasma, liver, ileum and adipose tissue (Table 1). DHEA was increased by both FO diets in all compartments compared to control diet. For EPEA, an interaction effect (see supplemental data) was observed in liver, but the compound was increased by the FO diets in plasma, ileum and adipose tissue. The endocannabinoids/NAEs derived from other fatty acids, such as AEA and 2-AG, were in general decreased by the FO diets, but some deviations were observed. For instance, 2-AG levels in adipose tissue and liver were decreased in both FO groups compared to the control diet. When comparing the 1 % versus the 3 % FO group, liver 2-AG was lower in the 3 % group, but higher in adipose tissue. DGLEA (also known as DLE) in liver was not influenced by the diets, but was decreased in plasma, ileum and adipose tissue in the FO groups. OEA was decreased in liver and plasma, but not in ileum. When comparing the control diet group with the 3 % FO group, SEA displayed opposite effects in adipose tissue and plasma; FO was found to decrease plasma levels, but increased adipose tissue levels of SEA. This was also observed when comparing the 1 % versus the 3 % FO groups.

The effect of LPS on endocannabinoids appeared to be both compound and tissue specific (Table 2; for effects of LPS on endocannabinoids for each diet group, please refer to the supplemental data). LPS increased DHEA levels in all compartments, but for some compounds tissue-specific effects were seen. LPS decreased plasma 2-AG, whereas it increased adipose tissue 2-AG. A similar divergence is seen for SEA and PEA. LPS increased plasma and ileum SEA levels, but decreases liver and adipose tissue SEA levels. PEA levels were decreased in liver by LPS, but increased in ileum.

In summary, both the FO diets and the LPS treatment affected plasma and tissue endocannabinoid/NAE levels. In general, DHEA and EPEA were increased by the FO diets, and compounds derived from other fatty acids were decreased, with different effects for 1 and 3 % FO diets. LPS raised endocannabinoid/NAE levels in general, but opposing effects were seen for 2-AG, PEA and SEA across the tissues investigated.

### FO diet and inflammation alter the oxylipin balance

The results of the oxylipin analyses in plasma, liver, ileum and adipose tissue are presented in Tables 3 and 4, with Table 3 showing diet effects and Table 4 LPS effects. Compounds with an interaction effects are highlighted with an * in the tables, with details provided in the supplemental data (S-5 and S-6). UK compounds are not presented in Table 5, but can be found in the supplemental data.

**Table 5:** Top lists generated from PCA analysis

	PC1		PC2
P_AEA	0.13562	F_pea	0.148486
P_AA	0.128696	L_dhea	0.141097
P_DLE	0.12706	F_TBXB3	0.138751
L_13,14-dihydro-15-keto-PGF2a	0.120315	P_DHEA	0.137637
L_AA	0.12015	P_19,20-DiHoPE	0.137193
L_2-ag	0.116734	F_UK3	0.136532
P_11,12-DiHETrE	0.116219	F_5(S)-HETE	0.136382
L_8,9-DiHETrE	0.115152	F_17 keto- 4(z), 7(z), 10(z), 13 (z), 15 (E), 19(z)-DHA	0.135592
I_13,14-dihydro-15-keto-PGE2	0.114106	F_PGE3	0.133149
I_2-ag	0.113331	F_19,20-DiHoPE	0.133074
L_15(S)-HETE	0.112971	F_oea	0.132124
F_aea	0.112488	F_UK5	0.131976
L_EPA	−0.11245	F_UK2	0.128994
L_14,15 EET	0.111192	I_PGE3	0.128277
_L_5(S)-HEPE_	−0.11066	F_UK4	0.127957
L_11(S)-HETE	0.10907	P_DHA	0.127188
P_14,15-DiHETrE	0.107335	F_AA	0.125684
I_13,14-dihydro-15-keto-PGF2a	0.105203	F_5(S)-HEPE	0.12297
P_8,9-DiHETrE	0.104781	I_19,20-DiHoPE	0.121367
L_11,12 EET	0.103724	F_12,13-DiHOME	0.12071
I_13,14-dihydro-15-keto-PGD2	0.100174	P_PEA	0.120517
L_PGE2	0.100147	P_12,13-DiHOME	0.118135
I_12(S)-HEPE	−0.09961	F_lipoxin A4	0.115389
F_2-ag	0.099293	P_EPEA	0.115294
L_14,15-DiHETrE	0.098683	F_5,6 EET	0.115132
L_12(S)-HHTrE	0.098509	F_PGD3	0.114641
I_EPA	−0.09806	F_dhea	0.113431
F_dle	0.09791	L_epea	0.112967
F_13,14-dihydro-15-keto-PGF2a	0.097779	P_9,10-DiHOME	0.11289
F_EPA	−0.09662	F_9,10-DiHOME	0.109149

The D-scores represent the variable’s weight in the separation, and is expressed as the numerical output value as obtained from the PCA model; the further away from zero, the better this variable accounts for group separation. PC1 separated the diets, whereas PC2 separated between saline and LPS treatment

*P* plasma, *L* liver, *I* ileum, *F* adipose tissue

The FO diets decreased levels of ARA and increased DHA and EPA, confirming that the increased dietary intake of n-3 fatty acids was reflected in tissue fatty acid levels (Table 3). Furthermore, n-3 derived oxylipin levels were increased by the FO diets, with the most pronounced effects observed in ileum and adipose tissue. The oxylipins derived from other fatty acids were in general decreased by the FO diets, with some exceptions, and effects were not always consistent over all tissues tested. Levels of LTB_4_ were decreased in ileum and adipose tissue by the FO diets, but liver levels were increased. Lipoxin A_4_ levels were increased in the 3 % FO group compared to the control and 1 % FO diet in liver, ileum and adipose tissue. When comparing the 1 and 3 % FO diets, ileal 5-HETE levels were decreased in the 3 % FO group, but its level was increased in adipose tissue. The FO diets decreased oxylipins belonging to different branches of the fatty acid oxylipin cascade, including the cyclooxygenase pathway (COX; PGD_2_, PGE2 and their metabolites 13,14-dihydro-15-keto-PGD_2_ and –PGE_2_, PGF_2α_, TBXB_2_), the 15-lipo-oxygenase pathway (15-LOX; 15-HETE), 12-LOX (11-HETE and 12-HETE), 5-LOX (5-HETE, LTB_4_ and LTD_4_) and the cytochrome P450 pathways (EETs and DiHETrEs) (see Fig. 1 for an overview).

Treatment with LPS generally resulted in increased levels of fatty acids, n-3 derived oxylipins and other oxylipins, with the most compounds affected in plasma and adipose tissue, and the least number of compounds altered in ileum (Table 4; for effects of LPS on oxylipins for each diet group, please refer to the supplemental data). Again, opposing effects were observed between compartments for some components. LPS decreased plasma levels of 11-HETE, but increased liver levels. TBXB_2_ was decreased by LPS in plasma, but increased in ileum and adipose tissue. Effects on UK compounds are listed in the supplemental data.

### Multivariate data analysis shows separation between diet groups and LPS treatment

**Fig. 2:**
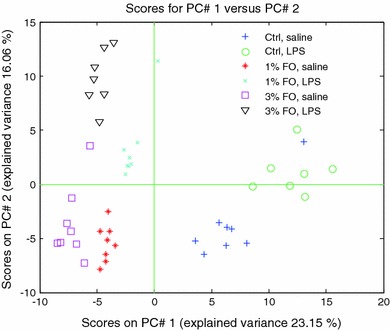
PCA analysis on fused data. The PCA plot shows good separation of the three diet groups. PC1 describes mainly the diet effect, and PC2 mainly the LPS effect

The univariate data analysis approach revealed that both FO and LPS altered endocannabinoid/NAE and oxylipin levels, and effects were seen in plasma, liver, ileum and adipose tissue. In total, 244 variables obtained in four compartments were evaluated, which were, due to complexity, further analyzed with multivariate data analysis to evaluate differences between treatment groups. Two methods were used, the unbiased PCA and the supervised PCDA. In the PCA plot (Fig. 2), a good separation of the six intervention groups can be seen. PC1 separated the diets, with negative loadings associated to n-3 fatty acid derived metabolites, and positive loadings belonging to other metabolites. From the top-30 variables relevant for group separation in PC1 (see Table 5), 13 variables were from liver, whereas plasma, ileum and adipose tissue were equally important. In total four metabolites were derived from n-3 fatty acids, and 26 were derived from other fatty acids. PC2 separated between saline and LPS treatment, containing equal numbers of n-3 derived- and other metabolites. From the top-30 variables accounting in PC2, 19 metabolites were from adipose tissue, with 15 n-3 fatty acid derived metabolites, and 15 derived from other fatty acids.

### The diet effect in the saline treated mice is explained by other variables than the diet effect in the LPS-treated mice

**Fig. 3:**
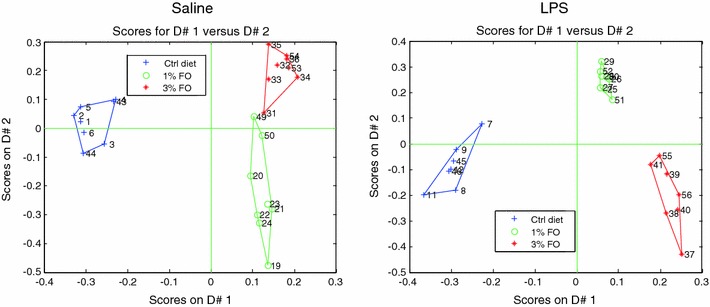
PCDA analysis, split for saline and LPS treated mice. A separation of diet groups is observed in both saline and LPS treated mice, with a more prominent separation in the LPS treated mice

To further explore differences between diet groups, PCDA was performed. The data was split for saline and LPS-treated mice, thus resulting in two separate PCDA plots. PCDA analyses showed that there is separation based on diet for both the saline and LPS-treated mice (Fig. 3). The contribution of a variable in the PCDA model is expressed as its D-score, with a positive score meaning an increase by the FO diets, and a negative score indicating a decrease. Analysis of D-scores focused on the 50 compounds with the highest D-scores as there was considerable decay in D-score values between the first and 50th compound, meaning that any differences within this range can be considered as a potentially meaningful difference. The analysis revealed that the diet groups are separated by increased levels of n-3 derived compounds in the FO groups, and compounds derived from other fatty acids were generally decreased by the FO diets (Table 6). In addition to this, both endocannabinoids/NAEs and oxylipins show up in the top of the rank lists, indicating that both classes of compounds are important to describe the diet effect. The ranking, number and origin of n-3 derived metabolites in the models is different between the saline and LPS treated animals. Out of the 50 compounds ranking highest for the saline treated mice, only 12 compounds are n-3 fatty acid derived metabolites, while for the LPS treated mice, the top-50 list contains 25 n-3 fatty acid derived metabolites. In addition to this, the majority of n-3 derived compounds in the LPS treated mice from this list originated from adipose tissue.

**Table 6:** Top lists for saline and LPS treated mice. Lists were generated from PCDA analysis, showing different patterns for saline and LPS treated mice

	Saline	D-score		LPS	D-score
	P_AEA	−4,6879		L_2-AG	−4,3994
	L_2-AG	−4,5742		P_AEA	−4,2737
	P_DGLEA	−4,5412	1	**F_EPA**	**4,2374**
	F_AEA	−4,4914	2	**F_12-HEPE**	**4,1634**
	P_ARA	−4,4486	3	**P_EPA**	**4,1441**
	P_11,12-DiHETrE	−4,4257		P_2-AG	−4,1053
1	**F_DHEA**	**4,3978**		P_AA	−4,0854
	P_14,15-DiHETrE	−4,3961	4	**I_PGE**_**3**_	**4,0450**
	L_ARA	−4,3915	5	**F_TBXB**_**3**_	**4,0374**
	L_13,14-dihydro-15-keto-PGF_2a_	−4,3025	6	**F_PGE**_**3**_	**3,9827**
	L_12-HETE	−4,2590		P_DGLEA	−3,9498
	F_8,9-DiHETrE	−4,2553		L_14,15-DiHETrE	−3,8977
	L_15-HETE	−4,2343	7	**F_DHEA**	**3,8946**
**2**	**L_5-HEPE**	**4,2162**	**8**	**I_EPA**	**3,8541**
	L_11-HETE	−4,1548	9	**L_EPEA**	**3,7505**
3	**L_EPA**	**4,1323**		L_11,12-DiHETrE	−3,7410
4	**P_EPA**	**4,1269**	10	**L_DHEA**	**3,7222**
	F_DGLEA	−4,1166		L_ARA	−3,6999
	P_8,9-DiHETrE	−4,0892	11	**L_EPA**	**3,6953**
	I_13,14-dihydro-15-keto-PGE_2_	−4,0783	12	**I_DHA**	**3,6912**
	P_OEA	−4,0592	13	**I_12-HEPE**	**3,6874**
	F_11,12 EET	−4,0186		L_8,9-DiHETrE	−3,6672
	F_OEA	−4,0148	14	**F_EPEA**	**3,6327**
	L_8,9-DiHETrE	−4,0079		L_13,14-dihydro-15-keto-PGF_2a_	−3,6261
	I_2-AG	−3,9897		I_2-AG	−3,6231
	F_9,10-DiHOME	−3,9147	15	**P_DHA**	**3,5903**
	P_9,10-DiHOME	−3,8645		F_AEA	−3,5678
	L_LTB4	3,8175		L_PGD_2_	−3,5455
	F_11,12-DiHETrE	−3,7600	16	**F_PGD**_**3**_	**3,5197**
	F_14,15-DiHETrE	−3,7376	17	**F_19,20-DiHoPE**	**3,5168**
	L_5,6 EET	−3,7094		L_AEA	−3,5158
	I_13,14-dihydro-15-keto-PGF_2a_	−3,7008	18	**I_19,20-DiHoPE**	**3,4924**
	P_12,13-DiHOME	−3,6962		L_14,15 EET	−3,4871
	F_12,13-DiHOME	−3,6718	19	**I_EPEA**	**3,4515**
5	**P_DHEA**	**3,6677**	20	**I_5-HEPE**	**3,4391**
	P_UK4	−3,6605		I_AEA	−3,4310
	L_14,15 EET	−3,6511		F_SEA	3,4187
6	**F_EPA**	**3,6069**		L_20-HETE	−3,4104
7	**I_19,20-DiHoPE**	**3,5938**		I_13,14-dihydro-15-keto-PGE_2_	−3,3956
	F_5,6-DiHETrE	−3,5357	21	**P_5-HEPE**	**3,3787**
	P_2-AG	−3,5155	22	**L_5-HEPE**	**3,3617**
8	**I_12-HEPE**	**3,4899**		L_TBXB_2_	−3,3200
	L_11,12 EET	−3,4781		L_12-HHTrE	−3,3155
9	F_EPEA	**3,4723**		P_5,6-DiHETrE	−3,3080
10	L_19,20-DiHoPE	**3,4679**	23	**F_5-HEPE**	**3,3048**
	L_12-HHTrE	−3,4525	24	**P_DHEA**	**3,2978**
	P_UK2	−3,4144		P_11,12-DiHETrE	−3,2966
	L_PGE_2_	−3,4143	25	**F_17 keto- 4(z), 7(z), 10(z), 13 (z), 15 (E), 19(z)-DHA**	**3,2931**
11	**I_EPA**	**3,3758**		L_11-HETE	−3,2792
12	**P_5-HEPE**	**3,3581**		P_15-HETE	−3,2618

The D-scores represent their weight, expressed as the numerical output value as obtained from the PCDA model; the further away from zero, the better this variable accounts for group separation. Negative scores indicate that the compound is decreased in the fish oil groups; positive scores mean that it is increased by fish oil. Decrease of other than n-3 derived compounds ranks relatively high in the saline diet effect, whereas an increase in n-3 derived compounds (printed in bold) ranks high for the diet effect in the LPS treated mice

*P* plasma, *L*  liver, *I* ileum, *F* adipose tissue

From these results, it can be concluded that the diet effect of FO in the saline treated animals is mainly explained by a decrease of compounds derived from other than n-3 fatty acids, and to a lesser extent by an increase of n-3 derived metabolites. However, for the LPS treated mice, the diet effect is principally explained by an increase of n-3 derived metabolites, and to a lesser extent by a decrease of metabolites derived from other than n-3 fatty acids.

## Discussion

Our results support the general idea that increasing dietary n-3 fatty intake results in increased levels of n-3 derived-endocannabinoids/NAEs and oxylipins. However, to the best of our knowledge, our study is the first one describing effects of dietary FO on the balance between the “endocannabinoid” and oxylipin pathways in such detail, in different compartments simultaneously, and in relation with inflammation. In addition, our study illustrates the risk of obtained potentially premature conclusions when only a few mediators are analyzed in a limited number of matrices. Several studies, focusing on for example AEA (anandamide) and 2-AG only, have concluded that dietary FO leads to an overall down regulation of the endocannabinoid system (Banni et al. 2011; Batetta et al. 2009). However, as we show other (n-3 derived-) endocannabinoids might be affected in an opposite direction following FO intake, and our data shows that the sum of all NAE levels in liver, ileum and plasma are actually quite stable with the different diets (data not shown). Although there are still several questions regarding their biological role, there are reports showing that n-3 derived ethanolamides have affinity for CB1 and CB2 receptors (Brown et al. 2010; Plastina et al. 2009b), and have anti-inflammatory properties (Balvers et al. 2010; Meijerink et al. 2011).

LPS was found to produce a general increase of in vivo endocannabinoid/NAE and oxylipin levels, although there were some exceptions (see below). Multivariate data analysis showed that the diet effect was also present during inflammatory conditions. Without LPS, the effect of a FO diet was mainly explained by a reduction of mediators other than those derived from n-3 fatty acids, and to a lesser degree by increased levels of n-3 derived metabolites. However, after LPS, the balance was shifted in favor of an increase of n-3 derived mediators while lower associations were found with reductions of non n-3 derived metabolites.

The relation between dietary fatty acid intake and the presence of endocannabinoids/NAEs and oxylipins in plasma and tissues has been established before (Banni and Di Marzo 2010; Hansen and Artmann 2008), but not under conditions of inflammation. Previous work with rats demonstrated that patterns of organ levels of NAEs follow the relative abundance of fatty acids in the diet (Artmann et al. 2008). Other work, investigating the effect of DHA on murine levels of endocannabinoids/NAEs in brain and plasma, showed strongest changes in plasma (Wood et al. 2010). Interestingly, plasma AEA levels were not significantly affected by DHA alone, whereas other NAEs were decreased by DHA. Other work, supplementing krill oil or menhaden oil to human subjects also did not show an effect on plasma AEA levels (Banni et al. 2011). Our work shows that 6 weeks of a FO diet is capable of reducing plasma AEA and 2-AG levels. This discrepancy might originate from differences in n-3 fatty acids sources, daily dose, or length of the period in which the n-3 fatty acids were supplemented.

Many studies analyze plasma levels of endocannabinoids/NAEs or oxylipins. The present work shows that plasma levels do not always reflect effects in liver, ileum or adipose tissue. For example, plasma 2-AG levels decreased after LPS, but were increased in adipose tissue, and similar divergences were also observed for PEA, SEA, several HETEs, and TBXB_2_. The origin and significance of these findings are not known yet, but this could be related to synthesis, release, uptake or breakdown which might be differentially regulated by LPS or other factors across different organs. Nevertheless, based on our results, extrapolating effects found in plasma to effects on peripheral tissues is not always appropriate. It should be noted that the recovery of endogenous metabolites from tissues might not be complete, potentially underestimating actual effects of the diet and inflammation in the tissues.

The LPS treated mice had a lower food intake combined with a small loss of body weight (data not shown), whereas the saline treated animals displayed normal food consumption and stable body weight. Previous work showed that levels of endocannabinoids and related NAEs depend on fasting status (Hansen and Diep 2009; Joosten et al. 2010; Li et al. 2011); their tissue levels being high during fasting, followed by a rapid postprandial decrease. Possibly, the effect of LPS on endocannabinoid levels might in part be mediated through such a ‘fasting’ effect. In addition, inflammation reduces FAAH expression, and inhibition of FAAH or monoacyl glycerol lipase (MGL) has been shown to reduce disease symptoms in several models of inflammation (Alhouayek et al. 2011; Maccarrone et al. 2001; Naidu et al. 2010). Similarly, studies using CB2 knock-out models under induced inflammatory conditions showed that increased levels of NAEs likely contribute to suppress inflammation (Bátkai et al. 2007). Together, this suggests that increased levels of endocannabinoids/NAEs are part of a normal response protecting against inflammatory stress. Previous work identified DHEA and EPEA as having anti-inflammatory properties in macrophages and adipocytes (Balvers et al. 2010; Meijerink et al. 2011), and these compounds could be another link between FO and its anti-inflammatory effects as n-3 derived NAEs were more effective than AEA in suppressing nitric oxide release from macrophages (Meijerink et al. 2011).

The FO diets also influenced levels of oxylipins, including metabolites from the COX, CYP450, and 5-LOX, 12-LOX and 15-LOX pathways (Fig. 1), and these effects were in general also seen during inflammatory conditions. Another strength of the present study is that we analyzed both n-6 and n-3 related oxylipins simultaneously in different compartments. In general, levels of n-3 fatty acid derived oxylipins (e.g. PGD_3_, PGE_3_, 5-HEPE, 12-HEPE and TBXB_3_) were increased with FO at the expense of oxylipins derived from other fatty acids (e.g. PGD_2_, PGE_2_, PGF_2α_, TBXB_2_ and members of the EET and HETE subclasses). A functional role in inflammation has been described for several of these compounds, and it is likely that the changes in profiles which are found in this study (and before) are causally related to the anti-inflammatory effects which are associated with n-3 fatty acid intake. For example, PGE_3_ is less potent than PGE_2_ in inducing COX-2 expression and IL-6 release (Bagga et al. 2003). A similar principle applies to the thromboxanes (Fischer and Weber 1983; von Schacky et al. 1985) and for 5-HETE/5-HEPE (Heidel et al. 1989), which were also altered by the FO diets.

Interestingly, the FO diets increased liver LTB_4_ levels, whereas ileum LTB_4_ levels were decreased. LTB_4_ has multiple pro-inflammatory functions in the immune system (Calder 2003), but the different effect of FO on organ levels of LTB_4_ is not understood. Lipoxin A_4_, a compound with anti-inflammatory properties (Schwab and Serhan 2006), was increased by the 3 % FO diet. This indicates that at least for this compound, which is synthesized from ARA, its levels are not directly related to dietary supply of precursors, but that other presently unknown factors are involved.

Levels of several EETs were reduced by the FO diets, especially in the liver. EETs play regulatory roles in heart and vascular physiology with effects on blood pressure regulation, but also have anti-inflammatory effects (Spector 2009). It thus seems that EETs do not play a role in the anti-inflammatory properties of n-3 fatty acids, but it should be noted that n-3 fatty acid derived EET analogues are reported to be endogenously present and have potent analgesic properties (Morisseau et al. 2010), but these specific EETs were not quantified in the current study. Another line of evidence suggests that EETs very specifically alter the release of either insulin or glucagon (Falck et al. 1983; Sacerdoti et al. 2003), pointing to a potential link between n-3 fatty acids and glucose metabolism. EETs might therefore also be part in mediating effects of dietary fatty acids on metabolism, but this relation has not been given much attention yet.

In the present study we did not detect resolvins in any of the samples. It might be that these compounds are not formed in quantities high enough to be detected with our method during the first 24 h after the initiation of the inflammatory response, or the detection limit of the analytical method was not sufficient to detect these compounds. The single time-point approach in the present study is a limitation of the work, and investigating a broader time range, e.g. studying multiple time points beyond 24 h after the initiation of inflammation, might reveal temporal changes in lipid mediators including resolvins.

The presence of 17-HDoHE (also known as 17-HDHA), a marker for resolvin synthesis (Poulsen et al. 2008) with anti-inflammatory properties (González-Périz et al. 2006), was increased by the FO diets. The FO diets as well as LPS increased levels of 10,17-DiHDoHE, (also known as protectin DX) which was previously shown to reduce inflammation and accelerate its resolution (Serhan et al. 2006). Altogether, the FO diets altered all branches in the oxylipin metabolome in a way that is largely associated with suppression of inflammation.

A major finding of this work is that the effects of FO were also persistent under inflammatory conditions. Multivariate data analysis revealed that both endocannabinoids and oxylipins are responsible for separation between diet groups. Under non-inflammatory conditions, the diet groups could be primarily separated based on the reduction of other than n-3 derived endocannabinoids and oxylipins. In contrast, with LPS treatment, the diet groups were primarily separated by increases in levels of n-3 fatty acid derived endocannabinoids and oxylipins. The combined approach of comparing normal versus inflammatory conditions was thus useful in demonstrating that effects of diet on oxylipins and endocannabinoids are depending on inflammatory status.

Recent evidence suggested that relatively high intakes of FO impairs the host’s resistance to microbial infection (Bonilla et al. 2010; Irons et al. 2003; Snel et al. 2010). In our study, the mice that had received 3 % FO showed relatively more severe signs of shock after LPS, and one mouse from the 3 % FO + LPS group died shortly before the end of the experiment. This would be in line with the notion that high FO intake might impair the host’s resistance to inflammatory stress, or to suppress the capability to overcome the inflammatory stimulus. We observed that plasma TBXB_2_ levels, a compound related to TBXA_2_ which is involved in vasoconstriction (Sellers and Stallone 2008), was decreased in the 3 % FO group compared to the 1 % FO group, but also by LPS. The combination 3 % FO and LPS treatment might have caused a decrease in TBXA_2_ levels below its physiological range, potentially increasing the risk of inducing excessive vasodilatation and shock. Alternatively, pre-treatment of rats with a CB1 blocker was effective in reducing hypotension after LPS administration (Varga et al. 1998), suggesting that increases in endocannabinoids after LPS might also contribute to the shock observed for the 3 % FO + LPS group. Future work should point out which (combination of) metabolites account for the impaired resistance in the 3 % FO + LPS group. Furthermore, future work should clarify which intake levels of n-3 fatty acids are beneficial to reduce symptoms of inflammatory diseases and where the inhibition of inflammation starts to interfere with an efficient response to an inflammatory stimulus.

In conclusion, dietary FO caused marked changes in the n-3 to n-6 balance of the endocannabinoid and oxylipin metabolomes, with specific effects depending on inflammatory status. The effects on metabolites are in line with the anti-inflammatory effects associated with n-3 fatty acid intake.

## Electronic supplementary material

Below is the link to the electronic supplementary material.

Supplementary material 1 (DOCX 21 kb)

Supplementary material 2 (DOC 3631 kb)

Supplementary material 3 (DOC 84 kb)

Supplementary material 4 (DOC 80 kb)

Supplementary material 5 (DOC 38 kb)

Supplementary material 6 (DOC 5337 kb)

Supplementary material 7 (DOCX 17 kb)

Supplementary material 8 (PPTX 370 kb)

## Abbreviations

2-AG: 2-Arachidonoyl glycerol
ACN: Acetonitrile
AEA: Arachidonoyl ethanolamide
ARA: Arachidonic acid
COX: Cyclooxygenase
CYP450: Cytochrome P450
DGLEA: Dihomo-γ-linolenoyl ethanolamide (also abbreviated as DLE)
DHA: Docosahexaenoic acid
DHEA: Docosahexaenoyl ethanolamide
DiHDoHE: Dihydroxydocosahexaenoic acid
DiHETrE: Dihydroxyeicosatrienoic acid (also abbreviated as DHET)
DiHOME: Dihydroxyoctadecenoic acid
DiHoPE: Dihydroxydocosapentaenoic acid (also abbreviated as DiHDPA)
EET: Epoxyeicosatrienoic acid
EPA: Eicosapentaenoic acids
EPEA: Eicosapentaenoyl ethanolamide
FO: Fish oil
HDoHE: Hydroxydocosahexaenoic acid
HEPE: Hydroxyeicosapentaenoic acid
HETE: Hydroxyeicosatetraenoic acid
HHTrE: Hydroxyheptadecatrienoic acid
HODE: Hydroxyoctadienoic acid
HOME: Hydroxyoctadecenoic acid
LA: Linoleic acid
LC–MS/MS: Liquid chromatrography–tandem mass spectrometry
LPS: Lipopolysaccharide
LOX: Lipooxygenase
LT: Leukotriene
NAE: N-Acyl ethanolamine
OEA: Oleoyl ethanolamide
PEA: Palmitoyl ethanolamide
PG: Prostaglandin
PUFA: Polyunsaturated fatty acid
SEA: Stearoyl ethanolamide
TBX: Thromboxane
