# Supplementary material for: Fish oil and inflammatory status alter the n-3 to n-6 balance of the endocannabinoid and oxylipin metabolomes in mouse plasma and tissues
Source: Metabolomics. 2012 Apr 11;8(6):1130–47. doi: 10.1007/s11306-012-0421-9 (PMC3483099; doi:10.1007/s11306-012-0421-9)
Supplement: Supplementary file 1 — Supplementary material 1 (DOCX 21 kb) [file 11306_2012_421_MOESM1_ESM.docx]

S-1

| *Compounds* | *Parent (m/z)* | *Product (m/z)* | *Retention time (min)* | *Internal standard* |
| --- | --- | --- | --- | --- |
|  | | | Xevo / Quantum |  |
| EPEA | 346 | 62 | 3.0 / 12.1 | AEA-d8 |
| AEA | 348 | 62 | 3.4 / 12.6 | AEA-d8 |
| 2-AG (NH_4_ adduct) | 379 (396) | 287 | 3.6 / 12.7 | 2-AG-d8 |
| PEA | 300 | 62 | 3.6 / 12.8 | AEA-d8 |
| DGLEA | 350 | 62 | 3.7 / 12.8 | AEA-d8 |
| DHEA | 372 | 62 | 3.8 / 12.5 | AEA-d8 |
| OEA | 326 | 62 | 3.8 / 13.0 | OEA-d4 |
| SEA | 328 | 62 | 4.3 / 13.5 | AEA-d8 |
|  | | | | |
| *Internal standards* | *Parent (m/z)* | *Product (m/z)* | *Retention time (min)* |  |
|  | | | | |
| AEA-d8 | 356 | 63 | 3.4 / 12.6 |  |
| 2-AG-d8 (NH_4_ adduct) | 387 (404) | 294 | 3.6 / 12.7 |  |
| OEA-d4 | 330 | 66 | 3.8 / 13.0 |  |

| *Compounds* | *Parent (m/z)* | | *Product (m/z)* | | *Retention time (min)* | | *Internal standard* | |
| --- | --- | --- | --- | --- | --- | --- | --- | --- |
|  | | | | |  | |  | |
| 2,3-dinor-8-iso-PGF_2a_ | 325.1 | | 237.2 | | 5.0 | | 8,9-DiHETrE-d11 | |
| TBXB_3_ | 367.1 | | 168.9 | | 5.9 | | TBXB_2_-d4 | |
| PGE_3_ | 349 | | 269.1 | | 6.1 | | PGE_2_-d4 | |
| PGF_2b_ | 353.2 | | 193.1 | | 6.1 | | 8-iso-PGF_2a_-d4 | |
| 8-iso-PGF_2a_ | 353.1 | | 193 | | 6.2 | | 8-iso-PGF_2a_-d4 | |
| PGD_3_ | 349.1 | | 269.1 | | 6.3 | | PGD_2_-d9 | |
| 11b-PGF_2a_ | 353.1 | | 193 | | 6.3 | | 11b-PGF_2a_-d4 | |
| 9,12,13-TriHOME * | 329.2 | | 211.1 | | 6.5 | | 8,9-DiHETrE-d11 | |
| 9,10,13-TriHOME * | 329.2 | | 171.1 | | 6.6 | | 8,9-DiHETrE-d11 | |
| TBXB_2_ | 369.1 | | 169 | | 6.6 | | TBXB_2_-d4 | |
| PGF_2a_ | 353.1 | | 193 | | 6.7 | | PGF_2a_-d4 | |
| PGE_2_ | 351.1 | | 271.2 | | 6.9 | | PGE_2_-d4 | |
| PGD_2_ | 351.1 | | 271.1 | | 7.1 | | PGD_2_-d9 | |
| Resolvin D_2_ | 375.1 | | 175 | | 7.3 | | 8,9-DiHETrE-d11 | |
| 13,14-dihydro-15-keto-PGE_2_ | 351.1 | | 175.1 | | 7.6 | | PGE_2_-d4 | |
| 13,14-dihydro-15-keto-PGF_2a_ | 353.1 | | 113.1 | | 7.6 | | 13-14-dihydro-15-keto-PGF2_a_-d4 | |
| Lipoxin A_4_ | 351.1 | | 114.9 | | 7.8 | | PGE_2_-d4 | |
| Resolvin D_1_ | 375.1 | | 140.8 | | 7.8 | | PGE_2_-d4 | |
| 13,14-dihydro-15-keto-PGD_2_ | 351.1 | | 175 | | 8.1 | | PGD_2_-d9 | |
| LTD_4_ | 495.1 | | 142.9 | | 8.6 | | LTD_4_-d5 | |
| PGB_2_ | 333.2 | | 174.9 | | 8.7 | | PGB_2_-d4 | |
| LTE_4_ | 438 | | 351 | | 9 | | LTD_4_-d5 | |
| Maresin | 359.6 | | 177.1 | | 9.6 | | 8,9-DiHETrE-d11 | |
| 10,17-DiHDoHE | 359.1 | | 152.9 | | 9.7 | | LTB_4_-d4 | |
| LTB_4_ | 335.1 | | 194.8 | | 10 | | LTB_4_-d4 | |
| 12,13-DiHOME | 313.2 | | 183 | | 10.1 | | 8,9-DiHETrE-d11 | |
| 9,10-DiHOME | 313.2 | | 201 | | 10.4 | | 8,9-DiHETrE-d11 | |
| 14,15-DiHETrE | 337.1 | | 207 | | 10.7 | | 8,9-DiHETrE-d11 | |
| 12-HHTrE | 279 | | 179.3 | | 10.8 | | 11b-PGF_2a_-d4 | |
| 19,20-DiHDoPE | 361.1 | | 272.7 | | 10.8 | | 8,9-DiHETrE-d11 | |
| 11,12-DiHETrE | 337.1 | | 166.9 | | 11.2 | | 8,9-DiHETrE-d11 | |
| n-acetyl LTE_4_ | 480 | | 351.1 | | 11.2 | | LTB_4_-d4 | |
| 8,9-DiHETrE | 337.1 | | 127 | | 11.6 | | 8,9-DiHETrE-d11 | |
| 15-deoxy-d-12,14-PGJ_2_ | 315 | | 271.1 | | 11.8 | | PGE_2_-d4 | |
| 20-HETE | 319.2 | | 275.3 | | 12 | | 20-HETE-d6 | |
| 5,6-DiHETrE | 337.1 | | 144.8 | | 12.2 | | 8,9-DiHETrE-d11 | |
| 12-HEPE | 317.2 | | 179 | | 12.3 | | 8,9-DiHETrE-d11 | |
| 13-HODE | 295.1 | | 195 | | 12.7 | | 13-HODE-d4 | |
| 5-HEPE | 317.2 | | 115 | | 12.8 | | 13-HODE-d4 | |
| 9-HODE | 295.1 | | 171 | | 12.9 | | 13-HODE-d4 | |
| 15-HETE | 319.2 | | 219.1 | | 13.3 | | 15-HETE-d8 | |
| UK1 | 295.2 | | 171.1 | | 13.5 | | 8,9-DiHETrE-d11 | |
| 17-HDoHE | 343.1 | | 281.4 | | 13.6 | | 13-HODE-d4 | |
| 11-HETE | 319.1 | | 167 | | 13.8 | | 15-HETE-d8 | |
| 12-HETE | 319.1 | | 179.2 | | 14 | | 15-HETE-d8 | |
| 17-keto-4(z),7(z),10(z),13(z),15(e),19(z) DHA | 341.5 | | 111 | | 14.1 | | 8,9-DiHETrE-d11 | |
| 5-HETE | 319.1 | | 203.1 | | 14.4 | | 15-HETE-d8 | |
| UK4 | 295.2 | | 195.2 | | 14.5 | | 8,9-DiHETrE-d11 | |
| 14,15-EET | 319.1 | | 219.2 | | 14.6 | | 14,15-EET-d11 | |
| UK2 | 295.2 | | 171.1 | | 14.6 | | 8,9-DiHETrE-d11 | |
| UK5 | 295.2 | | 195.2 | | 14.6 | | 8,9-DiHETrE-d11 | |
| UK3 | 295.2 | | 171.1 | | 14.7 | | 8,9-DiHETrE-d11 | |
| 11,12-EET | 319.1 | | 167 | | 14.8 | | 14,15-EET-d11 | |
| 8,9-EET | 319.1 | | 167 | | 14.8 | | 14,15-EET-d11 | |
| 5,6-EET | 319.1 | | 191.3 | | 14.9 | | 14,15-EET-d11 | |
| EPA | 301.1 | | 257.2 | | 15.3 | | ARA-d8 | |
| DHA | 327.1 | | 283.1 | | 15.5 | | ARA-d8 | |
| ARA | 303.1 | | 259.2 | | 15.6 | | ARA-d8 | |
| *Internal standards* | | *Parent (m/z)* | | *Product (m/z)* | | *Retention time (min)* | |  |
|  | | | | | | | | |
| 8-iso-PGF_2a_-d4 | | 357.1 | | 196.9 | | 6.2 | |  |
| 11b-PGF_2a_-d4 | | 357.1 | | 313.4 | | 6.3 | |  |
| TBXB_2_-d4 | | 373.1 | | 173 | | 6.6 | |  |
| PGF_2a_-d4 | | 357.1 | | 313.4 | | 6.7 | |  |
| PGE_2_-d4 | | 355.1 | | 275.1 | | 6.9 | |  |
| PGD_2_-d9 | | 360.3 | | 280.1 | | 7.1 | |  |
| 13-14-dihydro-15-keto-PGF2_a_-d4 | | 357.1 | | 187 | | 7.6 | |  |
| LTD_4_-d5 | | 500 | | 142.9 | | 8.6 | |  |
| PGB_2_-d4 | | 337.1 | | 178.9 | | 8.7 | |  |
| CUDA | | 339.1 | | 214.1 | | 10 | |  |
| LTB_4_-d4 | | 339.1 | | 197.1 | | 10 | |  |
| 8,9-DiHETrE-d11 | | 348.2 | | 127 | | 11.6 | |  |
| 20-HETE-d6 | | 325.1 | | 281.3 | | 12 | |  |
| 13-HODE-d4 | | 299.2 | | 198.2 | | 12.7 | |  |
| 15-HETE-d8 | | 327.2 | | 226.1 | | 13.3 | |  |
| 14,15-EET-d11 | | 330.2 | | 268.3 | | 14.6 | |  |
| ARA-d8 | | 311.2 | | 267.2 | | 15.6 | |  |
