# Supplementary material for: Fish oil and inflammatory status alter the n-3 to n-6 balance of the endocannabinoid and oxylipin metabolomes in mouse plasma and tissues
Source: Metabolomics. 2012 Apr 11;8(6):1130–47. doi: 10.1007/s11306-012-0421-9 (PMC3483099; doi:10.1007/s11306-012-0421-9)
Supplement: Supplementary file 2 — Supplementary material 2 (DOC 3631 kb) [file 11306_2012_421_MOESM2_ESM.doc]

| **Plasma** |  | 12(S)-HHTrE | 9(S)-HODE | 13(S)-HODE | EPA | AA | 15-deoxy-d-12,14-PGJ2 | 12(S)-HEPE | 5(S)-HEPE |
| --- | --- | --- | --- | --- | --- | --- | --- | --- | --- |
|  |  | ng/ml | ng/ml | ng/ml | ng/ml | ng/ml | ng/ml | ng/ml | ng/ml |
|  |  |  |  |  |  |  |  |  |  |
| ctrl, saline |  | 11,691 | 8,521 | 11,689 | 14,345 | 1795,198 | 0,100 | 0,562 | 0,100 |
|  |  | 4,396 | 0,993 | 1,682 | 22,549 | 1898,963 | 0,100 | 0,259 | 0,100 |
|  |  | 8,790 | 1,764 | 3,263 | 16,125 | 1734,386 | 0,100 | 0,402 | 0,100 |
|  |  | 1,698 | 1,384 | 2,377 | 13,937 | 1218,086 | 0,100 | 0,040 | 0,100 |
|  |  | 9,558 | 1,051 | 1,728 | 18,033 | 1856,846 | 0,100 | 0,542 | 0,100 |
|  |  | 1,792 | 1,119 | 1,963 | 12,384 | 1302,038 | 0,100 | 0,020 | 0,100 |
|  |  | 6,384 | 5,440 | 7,417 | 11,647 | 1495,863 | 0,100 | 0,206 | 0,100 |
|  |  | 2,271 | 1,228 | 2,523 | 13,683 | 1115,364 | 0,100 | 0,020 | 0,100 |
|  | *average* | *5,822* | *2,687* | *4,080* | *15,338* | *1552,093* | *0,100* | *0,256* | *0,100* |
|  | *SD* | *3,881* | *2,785* | *3,601* | *3,544* | *310,186* | *0,000* | *0,226* | *0,000* |
|  |  |  |  |  |  |  |  |  |  |
| ctrl, LPS |  | 1,823 | 2,344 | 3,016 | 27,190 | 2214,078 | 0,100 | 0,728 | 0,100 |
|  |  | 3,171 | 2,185 | 3,518 | 38,715 | 3060,171 | 0,100 | 0,581 | 0,100 |
|  |  | 1,549 | 1,397 | 1,554 | 27,599 | 2326,730 | 0,100 | 0,282 | 0,100 |
|  |  | 0,631 | 3,413 | 3,997 | 39,458 | 2793,306 | 0,100 | 0,140 | 0,100 |
|  |  | 2,737 | 2,738 | 3,571 | 36,091 | 3349,521 | 0,100 | 1,330 | 0,100 |
|  |  | 0,548 | 1,256 | 2,007 | 29,338 | 2607,253 | 0,100 | 0,250 | 0,100 |
|  |  | 2,153 | 2,472 | 2,728 | 31,487 | 2458,688 | 0,100 | 0,267 | 0,100 |
|  |  | 0,411 | 1,537 | 1,918 | 20,360 | 2206,365 | 0,100 | 0,206 | 0,100 |
|  | *average* | *1,628* | *2,168* | *2,788* | *31,280* | *2627,014* | *0,100* | *0,473* | *0,100* |
|  | *SD* | *1,040* | *0,739* | *0,891* | *6,532* | *414,669* | *0,000* | *0,400* | *0,000* |
|  |  |  |  |  |  |  |  |  |  |
| 1% FO, saline | | 7,051 | 5,595 | 6,439 | 514,157 | 736,147 | 0,100 | 21,924 | 0,291 |
|  |  | 1,974 | 1,320 | 2,451 | 603,776 | 638,446 | 0,100 | 3,846 | 0,307 |
|  |  | 9,453 | 1,128 | 1,814 | 476,503 | 619,971 | 0,100 | 29,850 | 0,666 |
|  |  | 3,404 | 1,106 | 2,060 | 673,124 | 778,582 | 0,100 | 8,188 | 0,344 |
|  |  | 5,148 | 1,000 | 2,260 | 420,626 | 570,883 | 0,100 | 7,782 | 0,229 |
|  |  | 6,045 | 1,047 | 2,329 | 719,353 | 938,449 | 0,100 | 16,629 | 0,529 |
|  |  | 4,635 | 0,900 | 1,823 | 616,958 | 650,442 | 0,100 | 14,779 | 0,237 |
|  |  | 8,147 | 1,776 | 3,829 | 580,164 | 691,595 | 0,100 | 22,729 | 0,373 |
|  | *average* | *5,732* | *1,734* | *2,876* | *575,583* | *703,064* | *0,100* | *15,716* | *0,372* |
|  | *SD* | *2,467* | *1,583* | *1,575* | *100,224* | *115,670* | *0,000* | *8,862* | *0,152* |
|  |  |  |  |  |  |  |  |  |  |
| 1% FO, LPS |  | 0,445 | 1,622 | 2,153 | 489,051 | 735,934 | 0,100 | 3,553 | 0,454 |
|  |  | 0,120 | 2,214 | 2,921 | 659,238 | 926,904 | 0,100 | 2,642 | 0,481 |
|  |  | 0,989 | 2,002 | 2,499 | 525,695 | 764,856 | 0,100 | 3,870 | 0,226 |
|  |  | 0,244 | 1,466 | 2,000 | 546,795 | 678,006 | 0,100 | 4,283 | 0,214 |
|  |  | 1,677 | 3,306 | 3,934 | 765,860 | 1016,056 | 0,100 | 5,692 | 0,518 |
|  |  | 2,387 | 4,967 | 6,372 | 737,566 | 1181,908 | 0,100 | 47,877 | 1,495 |
|  |  | 0,598 | 1,595 | 1,907 | 709,821 | 874,791 | 0,100 | 4,848 | 0,300 |
|  |  | 0,179 | 1,733 | 2,440 | 515,120 | 800,659 | 0,100 | 4,632 | 0,308 |
|  | *average* | *0,830* | *2,363* | *3,028* | *618,643* | *872,389* | *0,100* | *9,675* | *0,499* |
|  | *SD* | *0,814* | *1,205* | *1,498* | *111,541* | *165,845* | *0,000* | *15,463* | *0,418* |
|  |  |  |  |  |  |  |  |  |  |
| 3% FO, saline | | 7,497 | 1,020 | 1,298 | 300,010 | 452,803 | 0,100 | 14,940 | 0,442 |
|  |  | 0,469 | 0,767 | 0,905 | 508,439 | 332,344 | 0,100 | 1,827 | 0,157 |
|  |  | 2,882 | 1,102 | 1,959 | 519,038 | 376,137 | 0,100 | 8,179 | 0,404 |
|  |  | 3,627 | 0,927 | 1,660 | 478,105 | 433,131 | 0,100 | 13,724 | 0,778 |
|  |  | 2,087 | 0,946 | 1,488 | 821,632 | 551,591 | 0,100 | 16,097 | 0,294 |
|  |  | 1,426 | 0,745 | 0,959 | 478,542 | 333,254 | 0,100 | 5,718 | 0,481 |
|  |  | 2,598 | 1,032 | 1,221 | 1127,398 | 494,321 | 0,100 | 10,763 | 0,413 |
|  |  | 0,232 | 0,920 | 1,291 | 471,910 | 214,977 | 0,100 | 1,086 | 0,100 |
|  | *average* | *2,602* | *0,932* | *1,348* | *588,134* | *398,570* | *0,100* | *9,042* | *0,384* |
|  | *SD* | *2,296* | *0,125* | *0,351* | *261,067* | *106,624* | *0,000* | *5,814* | *0,210* |
|  |  |  |  |  |  |  |  |  |  |
| 3% FO, LPS | | 0,420 | 1,938 | 2,049 | 1175,330 | 976,684 | 0,100 | 12,574 | 0,946 |
|  |  | 0,178 | 2,107 | 2,657 | 1552,856 | 954,717 | 0,100 | 11,269 | 1,484 |
|  |  | 0,579 | 2,915 | 3,755 | 1821,134 | 1187,420 | 0,100 | 55,037 | 1,287 |
|  |  | 0,534 | 3,348 | 3,852 | 1414,432 | 1057,115 | 0,100 | 41,592 | 0,941 |
|  |  | 0,415 | 2,202 | 2,340 | 1152,799 | 837,365 | 0,100 | 9,169 | 0,431 |
|  |  | 0,361 | 1,498 | 1,561 | 1273,864 | 1152,851 | 0,100 | 6,336 | 1,091 |
|  |  | 0,214 | 1,760 | 1,814 | 1851,869 | 974,532 | 0,100 | 12,460 | 1,896 |
|  | *average* | *0,386* | *2,252* | *2,575* | *1463,183* | *1020,098* | *0,100* | *21,205* | *1,154* |
|  | *SD* | *0,150* | *0,655* | *0,910* | *290,018* | *121,530* | *0,000* | *19,043* | *0,464* |

| **Plasma** |  | 20(S)-HETE | 15(S)-HETE | 11(S)-HETE | 12(S)-HETE | 11,12 EET | 8,9 EET | 5,6 EET | 5(S)-HETE | 14,15 EET |
| --- | --- | --- | --- | --- | --- | --- | --- | --- | --- | --- |
|  |  | ng/ml | ng/ml | ng/ml | ng/ml | ng/ml | ng/ml | ng/ml | ng/ml | ng/ml |
|  |  |  |  |  |  |  |  |  |  |  |
| ctrl, saline |  | 0,800 | 0,563 | 0,689 | 65,939 | 0,105 | 0,300 | 0,800 | 0,089 | 0,201 |
|  |  | 0,800 | 0,190 | 0,362 | 20,571 | 0,104 | 0,300 | 0,670 | 0,095 | 0,050 |
|  |  | 0,800 | 0,518 | 0,594 | 48,668 | 0,079 | 0,300 | 0,050 | 0,026 | 0,050 |
|  |  | 0,800 | 0,119 | 0,124 | 2,587 | 0,053 | 0,300 | 0,968 | 0,015 | 0,050 |
|  |  | 0,800 | 0,508 | 0,515 | 63,223 | 0,010 | 0,300 | 1,173 | 0,058 | 0,082 |
|  |  | 0,800 | 0,153 | 0,191 | 3,930 | 0,010 | 0,300 | 0,050 | 0,030 | 0,126 |
|  |  | 0,800 | 0,290 | 0,378 | 25,907 | 0,050 | 0,300 | 0,050 | 0,041 | 0,050 |
|  |  | 0,800 | 0,148 | 0,145 | 8,091 | 0,010 | 0,300 | 0,050 | 0,010 | 0,050 |
|  | *average* | *0,800* | *0,311* | *0,374* | *29,864* | *0,053* | *0,300* | *0,476* | *0,045* | *0,082* |
|  | *SD* | *0,000* | *0,188* | *0,213* | *26,078* | *0,041* | *0,000* | *0,478* | *0,032* | *0,055* |
|  |  |  |  |  |  |  |  |  |  |  |
| ctrl, LPS |  | 0,800 | 0,229 | 0,269 | 26,889 | 0,125 | 0,300 | 0,633 | 0,057 | 0,060 |
|  |  | 0,800 | 0,266 | 0,306 | 27,267 | 0,150 | 0,300 | 0,647 | 0,105 | 0,179 |
|  |  | 0,800 | 0,122 | 0,186 | 13,434 | 0,085 | 0,300 | 0,847 | 0,046 | 0,050 |
|  |  | 0,800 | 0,154 | 0,243 | 4,460 | 0,186 | 0,300 | 0,050 | 0,111 | 0,436 |
|  |  | 0,800 | 0,352 | 0,421 | 49,700 | 0,150 | 0,300 | 2,294 | 0,104 | 0,094 |
|  |  | 0,800 | 0,091 | 0,132 | 5,475 | 0,056 | 0,300 | 0,875 | 0,122 | 0,050 |
|  |  | 0,800 | 0,171 | 0,229 | 13,461 | 0,010 | 0,300 | 0,050 | 0,010 | 0,050 |
|  |  | 0,800 | 0,111 | 0,162 | 4,200 | 0,010 | 0,300 | 0,050 | 0,076 | 0,050 |
|  | *average* | *0,800* | *0,187* | *0,243* | *18,111* | *0,096* | *0,300* | *0,681* | *0,079* | *0,121* |
|  | *SD* | *0,000* | *0,089* | *0,091* | *15,774* | *0,067* | *0,000* | *0,743* | *0,039* | *0,135* |
|  |  |  |  |  |  |  |  |  |  |  |
| 1% FO, saline |  | 0,800 | 0,384 | 0,417 | 27,599 | 0,010 | 0,300 | 0,050 | 0,010 | 0,050 |
|  |  | 0,800 | 0,125 | 0,150 | 5,797 | 0,010 | 0,300 | 0,050 | 0,010 | 0,050 |
|  |  | 0,800 | 0,528 | 0,624 | 37,947 | 0,046 | 0,300 | 0,050 | 0,010 | 0,050 |
|  |  | 0,800 | 0,185 | 0,199 | 10,050 | 0,010 | 0,300 | 0,050 | 0,010 | 0,050 |
|  |  | 0,800 | 0,275 | 0,347 | 15,225 | 0,047 | 0,300 | 0,050 | 0,010 | 0,050 |
|  |  | 0,800 | 0,334 | 0,382 | 26,657 | 0,010 | 0,300 | 0,050 | 0,028 | 0,050 |
|  |  | 0,800 | 0,151 | 0,204 | 18,352 | 0,010 | 0,300 | 0,050 | 0,010 | 0,050 |
|  |  | 0,800 | 0,303 | 0,460 | 26,947 | 0,010 | 0,300 | 0,050 | 0,010 | 0,050 |
|  | *average* | *0,800* | *0,285* | *0,348* | *21,071* | *0,019* | *0,300* | *0,050* | *0,012* | *0,050* |
|  | *SD* | *0,000* | *0,134* | *0,159* | *10,620* | *0,017* | *0,000* | *0,000* | *0,006* | *0,000* |
|  |  |  |  |  |  |  |  |  |  |  |
| 1% FO, LPS |  | 0,800 | 0,027 | 0,084 | 3,994 | 0,010 | 0,300 | 0,050 | 0,036 | 0,050 |
|  |  | 0,800 | 0,057 | 0,106 | 1,982 | 0,047 | 0,300 | 0,050 | 0,036 | 0,050 |
|  |  | 0,800 | 0,131 | 0,173 | 4,440 | 0,029 | 0,300 | 0,050 | 0,010 | 0,050 |
|  |  | 0,800 | 0,021 | 0,065 | 1,798 | 0,034 | 0,300 | 0,050 | 0,010 | 0,050 |
|  |  | 0,800 | 0,096 | 0,130 | 7,621 | 0,010 | 0,300 | 0,050 | 0,052 | 0,050 |
|  |  | 0,800 | 0,205 | 0,599 | 21,065 | 0,050 | 0,300 | 0,050 | 0,151 | 0,050 |
|  |  | 0,800 | 0,049 | 0,069 | 2,676 | 0,010 | 0,300 | 0,050 | 0,010 | 0,050 |
|  |  | 0,800 | 0,040 | 0,069 | 2,255 | 0,010 | 0,300 | 0,050 | 0,010 | 0,050 |
|  | *average* | *0,800* | *0,078* | *0,162* | *5,729* | *0,025* | *0,300* | *0,050* | *0,039* | *0,050* |
|  | *SD* | *0,000* | *0,063* | *0,180* | *6,484* | *0,017* | *0,000* | *0,000* | *0,048* | *0,000* |
|  |  |  |  |  |  |  |  |  |  |  |
| 3% FO, saline |  | 0,800 | 0,355 | 0,411 | 16,451 | 0,010 | 0,300 | 0,050 | 0,010 | 0,050 |
|  |  | 0,800 | 0,013 | 0,027 | 1,196 | 0,010 | 0,300 | 0,050 | 0,010 | 0,050 |
|  |  | 0,800 | 0,111 | 0,166 | 7,305 | 0,010 | 0,300 | 0,050 | 0,010 | 0,050 |
|  |  | 0,800 | 0,124 | 0,226 | 12,484 | 0,010 | 0,300 | 0,050 | 0,022 | 0,050 |
|  |  | 0,800 | 0,085 | 0,145 | 15,072 | 0,010 | 0,300 | 0,050 | 0,010 | 0,050 |
|  |  | 0,800 | 0,080 | 0,113 | 4,101 | 0,010 | 0,300 | 0,050 | 0,010 | 0,050 |
|  |  | 0,800 | 0,125 | 0,130 | 6,159 | 0,010 | 0,300 | 0,050 | 0,010 | 0,050 |
|  |  | 0,800 | 0,016 | 0,031 | 0,574 | 0,010 | 0,300 | 0,050 | 0,010 | 0,050 |
|  | *average* | *0,800* | *0,113* | *0,156* | *7,917* | *0,010* | *0,300* | *0,050* | *0,012* | *0,050* |
|  | *SD* | *0,000* | *0,107* | *0,123* | *6,118* | *0,000* | *0,000* | *0,000* | *0,004* | *0,000* |
|  |  |  |  |  |  |  |  |  |  |  |
| 3% FO, LPS |  | 0,800 | 0,078 | 0,095 | 4,065 | 0,010 | 0,300 | 0,050 | 0,049 | 0,050 |
|  |  | 0,800 | 0,033 | 0,076 | 2,004 | 0,053 | 0,300 | 0,050 | 0,010 | 0,050 |
|  |  | 0,800 | 0,018 | 0,099 | 8,476 | 0,010 | 0,300 | 0,050 | 0,038 | 0,050 |
|  |  | 0,800 | 0,074 | 0,139 | 10,770 | 0,010 | 0,300 | 0,050 | 0,096 | 0,050 |
|  |  | 0,800 | 0,027 | 0,063 | 2,980 | 0,010 | 0,300 | 0,050 | 0,010 | 0,050 |
|  |  | 0,800 | 0,036 | 0,055 | 1,967 | 0,010 | 0,300 | 0,954 | 0,045 | 0,050 |
|  |  | 0,800 | 0,034 | 0,087 | 1,589 | 0,010 | 0,300 | 0,563 | 0,010 | 0,050 |
|  | *average* | *0,800* | *0,043* | *0,087* | *4,550* | *0,016* | *0,300* | *0,252* | *0,037* | *0,050* |
|  | *SD* | *0,000* | *0,023* | *0,028* | *3,622* | *0,016* | *0,000* | *0,364* | *0,031* | *0,000* |

| **Plasma** |  | DHA | PGB2 | LTB4 | 14,15-DiHETrE | 11,12-DiHETrE | 5,6-DiHETrE | 17(S)-HDoHE | PGE3 | PGD3 |
| --- | --- | --- | --- | --- | --- | --- | --- | --- | --- | --- |
|  |  | ng/ml | ng/ml | ng/ml | ng/ml | ng/ml | ng/ml | ng/ml | ng/ml | ng/ml |
|  |  |  |  |  |  |  |  |  |  |  |
| ctrl, saline |  | 1832,818 | 0,157 | 0,040 | 0,209 | 0,130 | 0,053 | 0,500 | 0,050 | 0,200 |
|  |  | 2514,404 | 0,062 | 0,040 | 0,245 | 0,133 | 0,044 | 0,500 | 0,050 | 0,200 |
|  |  | 1757,192 | 0,054 | 0,040 | 0,210 | 0,093 | 0,015 | 0,500 | 0,050 | 0,200 |
|  |  | 1492,914 | 0,050 | 0,040 | 0,153 | 0,093 | 0,018 | 0,500 | 0,050 | 0,200 |
|  |  | 2061,075 | 0,097 | 0,040 | 0,222 | 0,108 | 0,022 | 0,500 | 0,050 | 0,200 |
|  |  | 1710,052 | 0,042 | 0,040 | 0,255 | 0,141 | 0,029 | 0,500 | 0,050 | 0,200 |
|  |  | 1386,683 | 0,020 | 0,040 | 0,120 | 0,061 | 0,015 | 0,500 | 0,050 | 0,200 |
|  |  | 1916,785 | 0,053 | 0,040 | 0,174 | 0,134 | 0,010 | 0,500 | 0,050 | 0,200 |
|  | *average* | *1833,990* | *0,067* | *0,040* | *0,198* | *0,111* | *0,026* | *0,500* | *0,050* | *0,200* |
|  | *SD* | *350,319* | *0,042* | *0,000* | *0,046* | *0,028* | *0,015* | *0,000* | *0,000* | *0,000* |
|  |  |  |  |  |  |  |  |  |  |  |
| ctrl, LPS |  | 3730,454 | 0,020 | 0,040 | 0,287 | 0,154 | 0,051 | 0,500 | 0,050 | 0,200 |
|  |  | 4787,355 | 0,034 | 0,040 | 0,406 | 0,274 | 0,058 | 0,500 | 0,050 | 0,200 |
|  |  | 4105,810 | 0,037 | 0,040 | 0,327 | 0,182 | 0,060 | 0,500 | 0,050 | 0,200 |
|  |  | 4511,218 | 0,076 | 0,040 | 0,423 | 0,278 | 0,095 | 0,500 | 0,050 | 0,200 |
|  |  | 4330,719 | 0,052 | 0,040 | 0,703 | 0,417 | 0,108 | 0,500 | 0,050 | 0,200 |
|  |  | 4023,596 | 0,062 | 0,040 | 0,321 | 0,185 | 0,145 | 0,500 | 0,050 | 0,200 |
|  |  | 4031,862 | 0,020 | 0,040 | 0,218 | 0,112 | 0,033 | 0,500 | 0,050 | 0,200 |
|  |  | 2965,024 | 0,020 | 0,040 | 0,287 | 0,233 | 0,070 | 0,500 | 0,050 | 0,200 |
|  | *average* | *4060,755* | *0,040* | *0,040* | *0,371* | *0,229* | *0,078* | *0,500* | *0,050* | *0,200* |
|  | *SD* | *550,028* | *0,021* | *0,000* | *0,149* | *0,095* | *0,036* | *0,000* | *0,000* | *0,000* |
|  |  |  |  |  |  |  |  |  |  |  |
| 1% FO, saline |  | 4163,603 | 0,082 | 0,040 | 0,060 | 0,044 | 0,037 | 0,500 | 0,071 | 0,200 |
|  |  | 5586,794 | 0,091 | 0,040 | 0,082 | 0,048 | 0,047 | 0,500 | 0,087 | 0,200 |
|  |  | 2799,856 | 0,085 | 0,040 | 0,062 | 0,023 | 0,030 | 0,500 | 0,083 | 0,200 |
|  |  | 5517,803 | 0,045 | 0,040 | 0,074 | 0,059 | 0,018 | 0,768 | 0,050 | 0,200 |
|  |  | 2564,272 | 0,040 | 0,040 | 0,059 | 0,022 | 0,015 | 0,533 | 0,050 | 0,200 |
|  |  | 5126,167 | 0,087 | 0,040 | 0,115 | 0,063 | 0,011 | 0,500 | 0,050 | 0,200 |
|  |  | 5254,231 | 0,020 | 0,040 | 0,072 | 0,021 | 0,015 | 0,500 | 0,050 | 0,200 |
|  |  | 4047,802 | 0,020 | 0,040 | 0,053 | 0,013 | 0,020 | 0,500 | 0,050 | 0,200 |
|  | *average* | *4382,566* | *0,059* | *0,040* | *0,072* | *0,037* | *0,024* | *0,538* | *0,061* | *0,200* |
|  | *SD* | *1196,593* | *0,031* | *0,000* | *0,020* | *0,019* | *0,013* | *0,094* | *0,016* | *0,000* |
|  |  |  |  |  |  |  |  |  |  |  |
| 1% FO, LPS |  | 5436,126 | 0,066 | 0,040 | 0,061 | 0,026 | 0,015 | 0,546 | 0,050 | 0,200 |
|  |  | 7659,189 | 0,075 | 0,040 | 0,196 | 0,070 | 0,015 | 0,394 | 0,050 | 0,200 |
|  |  | 6406,791 | 0,073 | 0,040 | 0,046 | 0,034 | 0,017 | 0,978 | 0,050 | 0,200 |
|  |  | 6219,134 | 0,042 | 0,040 | 0,045 | 0,044 | 0,015 | 0,523 | 0,050 | 0,200 |
|  |  | 7815,841 | 0,070 | 0,040 | 0,093 | 0,047 | 0,025 | 0,762 | 0,050 | 0,200 |
|  |  | 8990,346 | 0,139 | 0,040 | 0,296 | 0,158 | 0,063 | 5,192 | 0,050 | 0,200 |
|  |  | 7184,815 | 0,020 | 0,040 | 0,099 | 0,038 | 0,015 | 0,500 | 0,050 | 0,200 |
|  |  | 6267,164 | 0,020 | 0,040 | 0,075 | 0,050 | 0,015 | 0,500 | 0,050 | 0,200 |
|  | *average* | *6997,426* | *0,063* | *0,040* | *0,114* | *0,058* | *0,022* | *1,174* | *0,050* | *0,200* |
|  | *SD* | *1136,417* | *0,038* | *0,000* | *0,088* | *0,042* | *0,017* | *1,634* | *0,000* | *0,000* |
|  |  |  |  |  |  |  |  |  |  |  |
| 3% FO, saline |  | 2029,079 | 0,030 | 0,040 | 0,060 | 0,030 | 0,011 | 0,500 | 0,155 | 0,200 |
|  |  | 2835,815 | 0,020 | 0,040 | 0,060 | 0,012 | 0,015 | 0,500 | 0,221 | 0,200 |
|  |  | 2222,217 | 0,021 | 0,040 | 0,060 | 0,023 | 0,026 | 0,500 | 0,410 | 0,200 |
|  |  | 2217,859 | 0,087 | 0,040 | 0,060 | 0,012 | 0,015 | 0,500 | 1,123 | 0,200 |
|  |  | 4205,386 | 0,051 | 0,040 | 0,060 | 0,036 | 0,015 | 0,500 | 0,298 | 0,200 |
|  |  | 1665,974 | 0,020 | 0,040 | 0,060 | 0,016 | 0,015 | 0,500 | 0,219 | 0,200 |
|  |  | 5148,468 | 0,020 | 0,040 | 0,121 | 0,045 | 0,015 | 0,500 | 0,050 | 0,200 |
|  |  | 1819,826 | 0,020 | 0,040 | 0,028 | 0,013 | 0,015 | 0,500 | 0,050 | 0,200 |
|  | *average* | *2768,078* | *0,034* | *0,040* | *0,064* | *0,023* | *0,016* | *0,500* | *0,316* | *0,200* |
|  | *SD* | *1253,415* | *0,024* | *0,000* | *0,026* | *0,012* | *0,004* | *0,000* | *0,348* | *0,000* |
|  |  |  |  |  |  |  |  |  |  |  |
| 3% FO, LPS |  | 6252,681 | 0,020 | 0,040 | 0,202 | 0,099 | 0,042 | 0,500 | 0,050 | 0,200 |
|  |  | 7197,312 | 0,020 | 0,040 | 0,144 | 0,048 | 0,015 | 0,500 | 0,050 | 0,200 |
|  |  | 9442,812 | 0,020 | 0,040 | 0,210 | 0,112 | 0,027 | 0,500 | 0,050 | 0,200 |
|  |  | 8531,392 | 0,020 | 0,040 | 0,269 | 0,114 | 0,021 | 0,500 | 0,050 | 0,200 |
|  |  | 6231,066 | 0,020 | 0,040 | 0,133 | 0,059 | 0,015 | 0,500 | 0,050 | 0,200 |
|  |  | 7452,881 | 0,020 | 0,040 | 0,138 | 0,058 | 0,015 | 0,500 | 0,050 | 0,200 |
|  |  | 5326,733 | 0,020 | 0,040 | 0,196 | 0,098 | 0,015 | 0,634 | 0,050 | 0,200 |
|  | *average* | *7204,982* | *0,020* | *0,040* | *0,185* | *0,084* | *0,022* | *0,519* | *0,050* | *0,200* |
|  | *SD* | *1426,649* | *0,000* | *0,000* | *0,050* | *0,028* | *0,010* | *0,050* | *0,000* | *0,000* |

| **Plasma** |  | 13,14-dihydro-15-keto-PGD2 | 13,14-dihydro-15-keto-PGE2 | PGF2b | 8-iso-PGF2a | PGF2a | 10(S)-17(S)-DiHDoHE | 19,20-DiHoPE | TBXB3 | TBXB2 |
| --- | --- | --- | --- | --- | --- | --- | --- | --- | --- | --- |
|  |  | ng/ml | ng/ml | ng/ml | ng/ml | ng/ml | ng/ml | ng/ml | ng/ml | ng/ml |
|  |  |  |  |  |  |  |  |  |  |  |
| ctrl, saline |  | 0,020 | 0,103 | 0,100 | 0,018 | 0,573 | 0,200 | 0,519 | 0,045 | 10,581 |
|  |  | 0,020 | 0,130 | 0,100 | 0,017 | 0,358 | 0,200 | 0,489 | 0,021 | 3,734 |
|  |  | 0,020 | 0,219 | 0,100 | 0,021 | 0,342 | 0,200 | 0,485 | 0,023 | 9,648 |
|  |  | 0,020 | 0,194 | 0,100 | 0,023 | 0,272 | 0,200 | 0,429 | 0,015 | 1,468 |
|  |  | 0,020 | 0,176 | 0,100 | 0,009 | 0,358 | 0,200 | 0,369 | 0,042 | 8,969 |
|  |  | 0,020 | 0,177 | 0,100 | 0,012 | 0,311 | 0,200 | 0,571 | 0,034 | 1,448 |
|  |  | 0,020 | 0,247 | 0,100 | 0,015 | 0,504 | 0,200 | 0,379 | 0,026 | 5,986 |
|  |  | 0,020 | 0,182 | 0,100 | 0,018 | 0,261 | 0,200 | 0,785 | 0,020 | 2,100 |
|  | *average* | *0,020* | *0,178* | *0,100* | *0,017* | *0,372* | *0,200* | *0,503* | *0,028* | *5,492* |
|  | *SD* | *0,000* | *0,046* | *0,000* | *0,005* | *0,110* | *0,000* | *0,133* | *0,011* | *3,829* |
|  |  |  |  |  |  |  |  |  |  |  |
| ctrl, LPS |  | 0,020 | 0,402 | 0,100 | 0,045 | 0,369 | 0,200 | 1,430 | 0,029 | 1,388 |
|  |  | 0,020 | 0,443 | 0,100 | 0,008 | 0,358 | 0,200 | 1,510 | 0,034 | 3,090 |
|  |  | 0,020 | 0,289 | 0,100 | 0,013 | 0,311 | 0,200 | 0,976 | 0,019 | 1,761 |
|  |  | 0,020 | 0,390 | 0,100 | 0,019 | 0,396 | 0,200 | 1,298 | 0,034 | 0,395 |
|  |  | 0,020 | 0,454 | 0,100 | 0,025 | 0,415 | 0,200 | 2,473 | 0,056 | 2,501 |
|  |  | 0,020 | 0,201 | 0,100 | 0,011 | 0,289 | 0,200 | 1,279 | 0,015 | 0,404 |
|  |  | 0,020 | 0,182 | 0,100 | 0,027 | 0,410 | 0,200 | 1,016 | 0,047 | 1,858 |
|  |  | 0,020 | 0,218 | 0,100 | 0,017 | 0,286 | 0,200 | 1,444 | 0,018 | 0,317 |
|  | *average* | *0,020* | *0,322* | *0,100* | *0,021* | *0,354* | *0,200* | *1,428* | *0,032* | *1,464* |
|  | *SD* | *0,000* | *0,113* | *0,000* | *0,012* | *0,053* | *0,000* | *0,465* | *0,014* | *1,039* |
|  |  |  |  |  |  |  |  |  |  |  |
| 1% FO, saline |  | 0,020 | 0,159 | 0,100 | 0,026 | 0,322 | 0,200 | 2,001 | 0,211 | 7,612 |
|  |  | 0,020 | 0,173 | 0,100 | 0,026 | 0,311 | 0,200 | 1,942 | 0,085 | 1,797 |
|  |  | 0,020 | 0,082 | 0,100 | 0,016 | 0,328 | 0,200 | 1,272 | 0,353 | 9,419 |
|  |  | 0,020 | 0,047 | 0,100 | 0,015 | 0,233 | 0,200 | 1,843 | 0,070 | 3,358 |
|  |  | 0,020 | 0,113 | 0,100 | 0,024 | 0,389 | 0,200 | 0,793 | 0,159 | 5,154 |
|  |  | 0,020 | 0,189 | 0,100 | 0,040 | 0,397 | 0,200 | 2,149 | 0,172 | 6,443 |
|  |  | 0,020 | 0,050 | 0,100 | 0,032 | 0,252 | 0,200 | 1,408 | 0,093 | 4,052 |
|  |  | 0,020 | 0,320 | 0,100 | 0,020 | 0,450 | 0,200 | 1,785 | 0,255 | 8,523 |
|  | *average* | *0,020* | *0,142* | *0,100* | *0,025* | *0,335* | *0,200* | *1,649* | *0,175* | *5,795* |
|  | *SD* | *0,000* | *0,090* | *0,000* | *0,008* | *0,074* | *0,000* | *0,455* | *0,097* | *2,664* |
|  |  |  |  |  |  |  |  |  |  |  |
| 1% FO, LPS |  | 0,020 | 0,247 | 0,100 | 0,015 | 0,188 | 0,200 | 2,418 | 0,039 | 0,582 |
|  |  | 0,020 | 0,132 | 0,100 | 0,019 | 0,224 | 0,200 | 4,197 | 0,033 | 0,153 |
|  |  | 0,020 | 0,144 | 0,100 | 0,015 | 0,415 | 0,200 | 2,475 | 0,018 | 0,947 |
|  |  | 0,020 | 0,050 | 0,100 | 0,026 | 0,269 | 0,200 | 1,628 | 0,015 | 0,278 |
|  |  | 0,020 | 0,192 | 0,100 | 0,013 | 0,344 | 0,200 | 3,678 | 0,051 | 1,595 |
|  |  | 0,020 | 3,441 | 0,100 | 0,017 | 0,254 | 0,200 | 14,168 | 0,066 | 1,342 |
|  |  | 0,020 | 0,193 | 0,100 | 0,021 | 0,271 | 0,200 | 2,486 | 0,020 | 0,287 |
|  |  | 0,020 | 0,102 | 0,100 | 0,029 | 0,318 | 0,200 | 2,734 | 0,011 | 0,187 |
|  | *average* | *0,020* | *0,563* | *0,100* | *0,019* | *0,285* | *0,200* | *4,223* | *0,032* | *0,672* |
|  | *SD* | *0,000* | *1,164* | *0,000* | *0,006* | *0,072* | *0,000* | *4,097* | *0,019* | *0,560* |
|  |  |  |  |  |  |  |  |  |  |  |
| 3% FO, saline |  | 0,020 | 0,082 | 0,100 | 0,025 | 0,431 | 0,200 | 1,473 | 0,344 | 6,528 |
|  |  | 0,020 | 0,050 | 0,100 | 0,033 | 0,233 | 0,200 | 1,597 | 0,036 | 0,452 |
|  |  | 0,020 | 0,125 | 0,100 | 0,027 | 0,576 | 0,200 | 0,855 | 0,148 | 2,488 |
|  |  | 0,020 | 0,084 | 0,100 | 0,018 | 0,366 | 0,200 | 1,666 | 0,154 | 3,471 |
|  |  | 0,020 | 0,050 | 0,100 | 0,017 | 0,291 | 0,200 | 3,301 | 0,123 | 2,032 |
|  |  | 0,020 | 0,050 | 0,100 | 0,028 | 0,381 | 0,200 | 0,681 | 0,070 | 1,330 |
|  |  | 0,020 | 0,138 | 0,100 | 0,014 | 0,352 | 0,200 | 3,789 | 0,105 | 2,601 |
|  |  | 0,020 | 0,083 | 0,100 | 0,030 | 0,394 | 0,200 | 1,140 | 0,015 | 0,326 |
|  | *average* | *0,020* | *0,083* | *0,100* | *0,024* | *0,378* | *0,200* | *1,813* | *0,125* | *2,403* |
|  | *SD* | *0,000* | *0,034* | *0,000* | *0,007* | *0,101* | *0,000* | *1,131* | *0,102* | *1,986* |
|  |  |  |  |  |  |  |  |  |  |  |
| 3% FO, LPS |  | 0,020 | 0,154 | 0,100 | 0,008 | 0,261 | 0,200 | 11,854 | 0,018 | 0,360 |
|  |  | 0,020 | 0,143 | 0,100 | 0,028 | 0,350 | 0,200 | 6,862 | 0,027 | 0,082 |
|  |  | 0,020 | 0,154 | 0,100 | 0,015 | 0,408 | 0,200 | 14,380 | 0,047 | 0,245 |
|  |  | 0,020 | 0,121 | 0,100 | 0,016 | 0,316 | 0,200 | 14,073 | 0,052 | 0,410 |
|  |  | 0,020 | 0,096 | 0,100 | 0,023 | 0,342 | 0,200 | 6,537 | 0,051 | 0,317 |
|  |  | 0,020 | 0,228 | 0,100 | 0,023 | 0,275 | 0,200 | 5,507 | 0,012 | 0,238 |
|  |  | 0,020 | 0,242 | 0,100 | 0,012 | 0,138 | 0,200 | 9,097 | 0,026 | 0,137 |
|  | *average* | *0,020* | *0,163* | *0,100* | *0,018* | *0,299* | *0,200* | *9,758* | *0,033* | *0,256* |
|  | *SD* | *0,000* | *0,054* | *0,000* | *0,007* | *0,086* | *0,000* | *3,689* | *0,016* | *0,118* |

| **Plasma** |  | 8,9-DiHETrE | 13,14-dihydro-15-keto-PGF2a | lipoxin A4 | PGE2 | PGD2 | Leukotriene E4 | n-acetyl leukotriene E4 | Leukotriene D4 |
| --- | --- | --- | --- | --- | --- | --- | --- | --- | --- |
|  |  | ng/ml | ng/ml | ng/ml | ng/ml | ng/ml | ng/ml | ng/ml | ng/ml |
|  |  |  |  |  |  |  |  |  |  |
| ctrl, saline |  | 0,185 | 0,200 | 0,040 | 0,409 | 0,335 | 1,000 | 0,200 | 0,500 |
|  |  | 0,118 | 0,200 | 0,040 | 0,453 | 0,249 | 1,000 | 0,200 | 0,500 |
|  |  | 0,125 | 0,200 | 0,040 | 0,416 | 0,340 | 1,000 | 0,200 | 0,500 |
|  |  | 0,158 | 0,200 | 0,040 | 0,184 | 0,030 | 1,000 | 0,200 | 0,500 |
|  |  | 0,154 | 0,200 | 0,040 | 0,398 | 0,350 | 1,000 | 0,200 | 0,500 |
|  |  | 0,142 | 0,200 | 0,040 | 0,107 | 0,054 | 1,000 | 0,200 | 0,500 |
|  |  | 0,030 | 0,200 | 0,040 | 0,279 | 0,179 | 1,000 | 0,200 | 0,500 |
|  |  | 0,110 | 0,200 | 0,040 | 0,306 | 0,032 | 1,000 | 0,200 | 0,500 |
|  | *average* | *0,128* | *0,200* | *0,040* | *0,319* | *0,196* | *1,000* | *0,200* | *0,500* |
|  | *SD* | *0,046* | *0,000* | *0,000* | *0,123* | *0,142* | *0,000* | *0,000* | *0,000* |
|  |  |  |  |  |  |  |  |  |  |
| ctrl, LPS |  | 0,243 | 0,200 | 0,040 | 0,558 | 0,235 | 1,000 | 0,200 | 0,500 |
|  |  | 0,352 | 0,200 | 0,040 | 0,521 | 0,196 | 1,000 | 0,200 | 0,500 |
|  |  | 0,347 | 0,200 | 0,040 | 0,886 | 0,261 | 1,000 | 0,200 | 0,500 |
|  |  | 0,433 | 0,200 | 0,040 | 0,649 | 0,030 | 1,000 | 0,200 | 0,500 |
|  |  | 0,339 | 0,200 | 0,040 | 0,613 | 0,320 | 1,000 | 0,200 | 0,500 |
|  |  | 0,206 | 0,200 | 0,040 | 0,298 | 0,058 | 1,000 | 0,200 | 0,500 |
|  |  | 0,078 | 0,200 | 0,040 | 0,923 | 0,306 | 1,000 | 0,200 | 0,500 |
|  |  | 0,238 | 0,200 | 0,040 | 0,347 | 0,084 | 1,000 | 0,200 | 0,500 |
|  | *average* | *0,280* | *0,200* | *0,040* | *0,599* | *0,186* | *1,000* | *0,200* | *0,500* |
|  | *SD* | *0,111* | *0,000* | *0,000* | *0,224* | *0,114* | *0,000* | *0,000* | *0,000* |
|  |  |  |  |  |  |  |  |  |  |
| 1% FO, saline |  | 0,064 | 0,200 | 0,040 | 0,409 | 0,138 | 1,000 | 0,200 | 0,500 |
|  |  | 0,047 | 0,200 | 0,040 | 0,094 | 0,200 | 1,000 | 0,200 | 0,500 |
|  |  | 0,035 | 0,200 | 0,040 | 0,287 | 0,160 | 1,000 | 0,200 | 0,500 |
|  |  | 0,031 | 0,200 | 0,040 | 0,234 | 0,157 | 1,000 | 0,200 | 0,500 |
|  |  | 0,018 | 0,200 | 0,040 | 0,153 | 0,186 | 1,000 | 0,200 | 0,500 |
|  |  | 0,024 | 0,200 | 0,040 | 0,321 | 0,204 | 1,000 | 0,200 | 0,500 |
|  |  | 0,030 | 0,200 | 0,040 | 0,234 | 0,135 | 1,000 | 0,200 | 0,500 |
|  |  | 0,030 | 0,200 | 0,040 | 0,347 | 0,239 | 1,000 | 0,200 | 0,500 |
|  | *average* | *0,035* | *0,200* | *0,040* | *0,260* | *0,177* | *1,000* | *0,200* | *0,500* |
|  | *SD* | *0,015* | *0,000* | *0,000* | *0,103* | *0,036* | *0,000* | *0,000* | *0,000* |
|  |  |  |  |  |  |  |  |  |  |
| 1% FO, LPS |  | 0,064 | 0,200 | 0,040 | 0,461 | 0,028 | 1,000 | 0,200 | 0,500 |
|  |  | 0,093 | 0,200 | 0,040 | 0,159 | 0,020 | 1,000 | 0,200 | 0,500 |
|  |  | 0,069 | 0,200 | 0,040 | 0,391 | 0,083 | 1,000 | 0,200 | 0,500 |
|  |  | 0,019 | 0,200 | 0,040 | 0,239 | 0,037 | 1,000 | 0,200 | 0,500 |
|  |  | 0,082 | 0,200 | 0,040 | 0,472 | 0,117 | 1,000 | 0,200 | 0,500 |
|  |  | 0,269 | 0,200 | 0,040 | 1,296 | 0,293 | 1,000 | 0,200 | 0,500 |
|  |  | 0,086 | 0,200 | 0,040 | 0,498 | 0,086 | 1,000 | 0,200 | 0,500 |
|  |  | 0,047 | 0,200 | 0,040 | 0,333 | 0,079 | 1,000 | 0,200 | 0,500 |
|  | *average* | *0,091* | *0,200* | *0,040* | *0,481* | *0,093* | *1,000* | *0,200* | *0,500* |
|  | *SD* | *0,076* | *0,000* | *0,000* | *0,350* | *0,087* | *0,000* | *0,000* | *0,000* |
|  |  |  |  |  |  |  |  |  |  |
| 3% FO, saline |  | 0,049 | 0,200 | 0,040 | 0,197 | 0,171 | 1,000 | 0,200 | 0,500 |
|  |  | 0,044 | 0,200 | 0,040 | 0,087 | 0,032 | 1,000 | 0,200 | 0,500 |
|  |  | 0,030 | 0,200 | 0,040 | 0,098 | 0,049 | 1,000 | 0,200 | 0,500 |
|  |  | 0,030 | 0,200 | 0,040 | 0,170 | 0,136 | 1,000 | 0,200 | 0,500 |
|  |  | 0,030 | 0,200 | 0,040 | 0,148 | 0,037 | 1,000 | 0,200 | 0,500 |
|  |  | 0,030 | 0,200 | 0,040 | 0,046 | 0,030 | 1,000 | 0,200 | 0,500 |
|  |  | 0,063 | 0,200 | 0,040 | 0,105 | 0,095 | 1,000 | 0,200 | 0,500 |
|  |  | 0,030 | 0,200 | 0,040 | 0,020 | 0,050 | 1,000 | 0,200 | 0,500 |
|  | *average* | *0,038* | *0,200* | *0,040* | *0,109* | *0,075* | *1,000* | *0,200* | *0,500* |
|  | *SD* | *0,013* | *0,000* | *0,000* | *0,060* | *0,053* | *0,000* | *0,000* | *0,000* |
|  |  |  |  |  |  |  |  |  |  |
| 3% FO, LPS |  | 0,100 | 0,200 | 0,040 | 0,297 | 0,133 | 1,000 | 0,200 | 0,500 |
|  |  | 0,105 | 0,200 | 0,040 | 0,259 | 0,043 | 1,000 | 0,200 | 0,500 |
|  |  | 0,162 | 0,200 | 0,040 | 0,197 | 0,076 | 1,000 | 0,200 | 0,500 |
|  |  | 0,182 | 0,200 | 0,040 | 0,397 | 0,084 | 1,000 | 0,200 | 0,500 |
|  |  | 0,092 | 0,200 | 0,040 | 0,288 | 0,032 | 1,000 | 0,200 | 0,500 |
|  |  | 0,030 | 0,200 | 0,040 | 0,171 | 0,030 | 1,000 | 0,200 | 0,500 |
|  |  | 0,134 | 0,200 | 0,040 | 0,256 | 0,042 | 1,000 | 0,200 | 0,500 |
|  | *average* | *0,115* | *0,200* | *0,040* | *0,266* | *0,063* | *1,000* | *0,200* | *0,500* |
|  | *SD* | *0,050* | *0,000* | *0,000* | *0,074* | *0,037* | *0,000* | *0,000* | *0,000* |

| **Plasma** |  | 17 keto- 4(z), 7(z), 10(z), 13 (z), 15 (E), 19(z)-DHA | 12,13-DiHOME | 9,10-DiHOME | 9,12,13-TriHOME | 9,10,13-TriHOME | UK1 | UK2 | UK3 | UK4 | UK5 |
| --- | --- | --- | --- | --- | --- | --- | --- | --- | --- | --- | --- |
|  |  | ng/ml | ng/ml | ng/ml | RR | RR | RR | RR | RR | RR | RR |
|  |  |  |  |  |  |  |  |  |  |  |  |
| ctrl, saline |  | 2,300 | 193,063 | 85,328 | 1001,442 | 2049,695 | 0,575 | 0,038 | 0,025 | 0,016 | 0,043 |
|  |  | 2,300 | 243,777 | 97,595 | 344,668 | 616,196 | 0,032 | 0,024 | 0,020 | 0,020 | 0,021 |
|  |  | 2,300 | 198,749 | 80,196 | 359,210 | 692,992 | 0,037 | 0,033 | 0,014 | 0,019 | 0,027 |
|  |  | 2,300 | 155,454 | 74,275 | 412,268 | 757,695 | 0,022 | 0,014 | 0,012 | 0,022 | 0,019 |
|  |  | 2,300 | 195,278 | 70,119 | 283,992 | 537,483 | 0,011 | 0,022 | 0,007 | 0,021 | 0,013 |
|  |  | 2,300 | 155,297 | 69,766 | 300,036 | 611,232 | 0,030 | 0,013 | 0,015 | 0,007 | 0,016 |
|  |  | 2,300 | 171,545 | 75,129 | 526,117 | 1142,180 | 0,119 | 0,017 | 0,016 | 0,010 | 0,023 |
|  |  | 2,300 | 364,089 | 141,725 | 327,429 | 711,443 | 0,064 | 0,016 | 0,015 | 0,011 | 0,017 |
|  | *average* | *2,300* | *209,656* | *86,767* | *444,395* | *889,864* | *0,111* | *0,022* | *0,015* | *0,016* | *0,022* |
|  | *SD* | *0,000* | *68,619* | *24,026* | *237,686* | *503,365* | *0,190* | *0,009* | *0,005* | *0,006* | *0,010* |
|  |  |  |  |  |  |  |  |  |  |  |  |
| ctrl, LPS |  | 2,300 | 246,870 | 163,594 | 540,776 | 1152,021 | 0,123 | 0,047 | 0,033 | 0,020 | 0,047 |
|  |  | 2,300 | 366,456 | 184,573 | 472,315 | 1316,417 | 0,068 | 0,049 | 0,042 | 0,026 | 0,048 |
|  |  | 2,300 | 168,478 | 126,677 | 346,473 | 764,002 | 0,078 | 0,041 | 0,044 | 0,007 | 0,032 |
|  |  | 2,300 | 350,270 | 199,027 | 867,015 | 1794,088 | 0,283 | 0,056 | 0,075 | 0,046 | 0,069 |
|  |  | 2,300 | 498,807 | 244,873 | 432,560 | 1109,563 | 0,142 | 0,063 | 0,041 | 0,035 | 0,053 |
|  |  | 2,300 | 182,959 | 108,622 | 689,583 | 1639,737 | 0,073 | 0,026 | 0,014 | 0,017 | 0,026 |
|  |  | 2,300 | 116,845 | 64,600 | 491,953 | 1084,148 | 0,149 | 0,020 | 0,015 | 0,007 | 0,023 |
|  |  | 2,300 | 161,434 | 122,523 | 368,324 | 683,989 | 0,072 | 0,023 | 0,018 | 0,013 | 0,025 |
|  | *average* | *2,300* | *261,515* | *151,811* | *526,125* | *1192,995* | *0,124* | *0,041* | *0,035* | *0,021* | *0,040* |
|  | *SD* | *0,000* | *131,576* | *57,399* | *174,492* | *385,400* | *0,072* | *0,016* | *0,020* | *0,014* | *0,017* |
|  |  |  |  |  |  |  |  |  |  |  |  |
| 1% FO, saline |  | 2,300 | 58,027 | 32,439 | 625,113 | 1280,332 | 0,132 | 0,016 | 0,024 | 0,008 | 0,011 |
|  |  | 2,300 | 150,118 | 71,505 | 390,984 | 798,503 | 0,061 | 0,010 | 0,008 | 0,008 | 0,010 |
|  |  | 2,300 | 35,757 | 35,519 | 358,452 | 677,503 | 0,036 | 0,011 | 0,015 | 0,005 | 0,014 |
|  |  | 2,300 | 119,989 | 48,630 | 342,563 | 655,396 | 0,026 | 0,019 | 0,013 | 0,008 | 0,019 |
|  |  | 2,300 | 33,139 | 26,499 | 467,994 | 998,312 | 0,038 | 0,006 | 0,007 | 0,004 | 0,008 |
|  |  | 2,300 | 151,876 | 63,622 | 310,296 | 746,112 | 0,031 | 0,015 | 0,012 | 0,009 | 0,021 |
|  |  | 2,300 | 75,569 | 34,949 | 219,415 | 454,785 | 0,041 | 0,010 | 0,009 | 0,011 | 0,010 |
|  |  | 2,300 | 108,870 | 45,847 | 393,571 | 683,143 | 0,097 | 0,010 | 0,009 | 0,008 | 0,022 |
|  | *average* | *2,300* | *91,668* | *44,876* | *388,548* | *786,761* | *0,058* | *0,012* | *0,012* | *0,008* | *0,014* |
|  | *SD* | *0,000* | *47,931* | *15,830* | *119,556* | *250,797* | *0,038* | *0,004* | *0,005* | *0,002* | *0,006* |
|  |  |  |  |  |  |  |  |  |  |  |  |
| 1% FO, LPS |  | 2,300 | 106,129 | 59,413 | 279,087 | 619,631 | 0,056 | 0,017 | 0,017 | 0,009 | 0,015 |
|  |  | 2,300 | 363,151 | 135,542 | 473,994 | 953,039 | 0,094 | 0,025 | 0,024 | 0,018 | 0,018 |
|  |  | 2,300 | 134,207 | 67,193 | 370,839 | 785,075 | 0,052 | 0,013 | 0,032 | 0,007 | 0,024 |
|  |  | 2,300 | 76,399 | 61,627 | 368,661 | 640,441 | 0,063 | 0,021 | 0,015 | 0,011 | 0,029 |
|  |  | 2,300 | 238,974 | 112,486 | 572,443 | 1182,039 | 0,098 | 0,038 | 0,038 | 0,008 | 0,031 |
|  |  | 2,300 | 1100,488 | 406,561 | 719,894 | 1528,909 | 0,154 | 0,028 | 0,029 | 0,016 | 0,017 |
|  |  | 2,300 | 130,793 | 86,615 | 227,414 | 535,839 | 0,051 | 0,022 | 0,032 | 0,018 | 0,027 |
|  |  | 2,300 | 174,125 | 128,061 | 477,652 | 797,576 | 0,072 | 0,028 | 0,025 | 0,020 | 0,026 |
|  | *average* | *2,300* | *290,533* | *132,187* | *436,248* | *880,318* | *0,080* | *0,024* | *0,027* | *0,013* | *0,023* |
|  | *SD* | *0,000* | *339,605* | *114,813* | *160,045* | *333,183* | *0,035* | *0,008* | *0,008* | *0,005* | *0,006* |
|  |  |  |  |  |  |  |  |  |  |  |  |
| 3% FO, saline |  | 2,300 | 14,183 | 15,740 | 387,863 | 774,927 | 0,011 | 0,011 | 0,017 | 0,012 | 0,020 |
|  |  | 2,300 | 26,697 | 15,674 | 300,760 | 509,201 | 0,013 | 0,008 | 0,017 | 0,004 | 0,009 |
|  |  | 2,300 | 20,475 | 20,356 | 342,066 | 635,247 | 0,021 | 0,003 | 0,008 | 0,005 | 0,013 |
|  |  | 2,300 | 15,429 | 15,979 | 483,808 | 583,086 | 0,036 | 0,004 | 0,012 | 0,001 | 0,016 |
|  |  | 2,300 | 102,110 | 40,856 | 228,338 | 520,241 | 0,016 | 0,015 | 0,009 | 0,001 | 0,012 |
|  |  | 2,300 | 14,222 | 15,267 | 308,306 | 631,111 | 0,019 | 0,008 | 0,008 | 0,001 | 0,007 |
|  |  | 2,300 | 194,529 | 65,140 | 209,346 | 386,299 | 0,011 | 0,011 | 0,003 | 0,007 | 0,014 |
|  |  | 2,300 | 28,060 | 21,022 | 181,426 | 389,373 | 0,026 | 0,006 | 0,008 | 0,007 | 0,010 |
|  | *average* | *2,300* | *51,963* | *26,254* | *305,239* | *553,685* | *0,019* | *0,008* | *0,010* | *0,005* | *0,013* |
|  | *SD* | *0,000* | *64,617* | *17,874* | *100,384* | *131,243* | *0,008* | *0,004* | *0,005* | *0,004* | *0,004* |
|  |  |  |  |  |  |  |  |  |  |  |  |
| 3% FO, LPS |  | 2,300 | 353,220 | 122,400 | 354,749 | 798,931 | 0,049 | 0,017 | 0,027 | 0,011 | 0,022 |
|  |  | 2,300 | 297,731 | 110,148 | 373,822 | 735,050 | 0,076 | 0,017 | 0,012 | 0,007 | 0,016 |
|  |  | 2,300 | 509,885 | 204,551 | 534,960 | 1147,033 | 0,126 | 0,013 | 0,023 | 0,014 | 0,034 |
|  |  | 2,300 | 522,413 | 172,561 | 390,761 | 850,870 | 0,079 | 0,023 | 0,024 | 0,012 | 0,026 |
|  |  | 2,300 | 344,015 | 129,423 | 389,898 | 801,083 | 0,059 | 0,029 | 0,021 | 0,002 | 0,017 |
|  |  | 2,300 | 308,327 | 121,952 | 263,612 | 530,945 | 0,072 | 0,023 | 0,032 | 0,013 | 0,026 |
|  |  | 2,300 | 421,892 | 152,494 | 513,255 | 940,475 | 0,036 | 0,019 | 0,033 | 0,013 | 0,019 |
|  | *average* | *2,300* | *393,926* | *144,790* | *403,008* | *829,198* | *0,071* | *0,020* | *0,025* | *0,011* | *0,023* |
|  | *SD* | *0,000* | *92,616* | *33,816* | *93,541* | *188,590* | *0,029* | *0,005* | *0,007* | *0,004* | *0,006* |

| **Plasma** |  | EPEA | DHEA | AEA | 2-AG | DLE | PEA | OEA | SEA |
| --- | --- | --- | --- | --- | --- | --- | --- | --- | --- |
|  |  | ng/ml | ng/ml | ng/ml | ng/ml | ng/ml | ng/ml | ng/ml | ng/ml |
|  |  |  |  |  |  |  |  |  |  |
| ctrl, saline |  | 0,100 | 0,330 | 0,366 | 21,827 | 0,065 | 3,342 | 5,822 | 1,886 |
|  |  | 0,100 | 0,394 | 0,379 | 32,683 | 0,060 | 3,485 | 5,727 | 2,275 |
|  |  | 0,100 | 0,326 | 0,248 | 49,751 | 0,037 | 3,087 | 4,567 | 2,875 |
|  |  | 0,100 | 0,244 | 0,232 | 35,021 | 0,076 | 2,915 | 3,787 | 2,320 |
|  |  | 0,100 | 0,289 | 0,326 | 22,147 | 0,069 | 3,735 | 4,703 | 2,473 |
|  |  | 0,100 | 0,235 | 0,265 | 27,023 | 0,047 | 2,768 | 4,248 | 1,671 |
|  |  | 0,100 | 0,253 | 0,262 | 69,053 | 0,053 | 2,975 | 4,149 | 2,672 |
|  |  | 0,100 | 0,483 | 0,307 | 38,427 | 0,074 | 4,569 | 6,721 | 4,456 |
|  | *average* | *0,100* | *0,319* | *0,298* | *36,991* | *0,060* | *3,360* | *4,966* | *2,578* |
|  | *SD* | *0,000* | *0,085* | *0,055* | *15,879* | *0,014* | *0,584* | *1,014* | *0,853* |
|  |  |  |  |  |  |  |  |  |  |
| ctrl, LPS |  | 0,100 | 0,604 | 0,531 | 21,661 | 0,113 | 7,144 | 16,963 | 6,823 |
|  |  | 0,100 | 0,699 | 0,430 | 26,358 | 0,067 | 5,012 | 10,427 | 4,492 |
|  |  | 0,100 | 0,633 | 0,369 | 22,981 | 0,079 | 5,261 | 12,172 | 4,661 |
|  |  | 0,100 | 0,575 | 0,436 | 23,141 | 0,094 | 5,208 | 10,707 | 4,393 |
|  |  | 0,100 | 0,829 | 0,566 | 29,933 | 0,107 | 6,492 | 14,185 | 5,938 |
|  |  | 0,100 | 0,551 | 0,405 | 20,006 | 0,093 | 4,563 | 10,951 | 3,805 |
|  |  | 0,100 | 0,488 | 0,406 | 31,757 | 0,095 | 5,326 | 12,816 | 5,191 |
|  |  | 0,100 | 0,616 | 0,510 | 19,908 | 0,104 | 7,318 | 15,441 | 6,780 |
|  | *average* | *0,100* | *0,625* | *0,457* | *24,468* | *0,094* | *5,791* | *12,958* | *5,260* |
|  | *SD* | *0,000* | *0,103* | *0,070* | *4,458* | *0,015* | *1,042* | *2,386* | *1,136* |
|  |  |  |  |  |  |  |  |  |  |
| 1% FO, saline |  | 0,100 | 0,607 | 0,057 | 9,374 | 0,010 | 2,715 | 3,231 | 2,562 |
|  |  | 0,100 | 0,540 | 0,034 | 14,887 | 0,010 | 2,407 | 3,259 | 2,170 |
|  |  | 0,100 | 0,587 | 0,039 | 14,703 | 0,010 | 3,236 | 2,239 | 3,269 |
|  |  | 0,211 | 0,712 | 0,048 | 23,998 | 0,010 | 2,882 | 3,745 | 2,363 |
|  |  | 0,100 | 0,706 | 0,054 | 16,363 | 0,010 | 2,737 | 2,433 | 2,695 |
|  |  | 0,313 | 0,701 | 0,068 | 18,761 | 0,010 | 3,354 | 4,099 | 2,736 |
|  |  | 0,326 | 0,793 | 0,063 | 19,975 | 0,010 | 3,255 | 3,757 | 2,981 |
|  |  | 0,100 | 0,580 | 0,029 | 13,063 | 0,010 | 2,872 | 2,941 | 3,106 |
|  | *average* | *0,169* | *0,653* | *0,049* | *16,390* | *0,010* | *2,932* | *3,213* | *2,735* |
|  | *SD* | *0,101* | *0,087* | *0,014* | *4,500* | *0,000* | *0,326* | *0,654* | *0,373* |
|  |  |  |  |  |  |  |  |  |  |
| 1% FO, LPS |  | 0,245 | 0,783 | 0,081 | 11,322 | 0,010 | 10,026 | 7,308 | 8,934 |
|  |  | 0,547 | 1,259 | 0,079 | 10,291 | 0,034 | 4,482 | 8,448 | 3,637 |
|  |  | 0,167 | 0,838 | 0,058 | 7,996 | 0,033 | 4,514 | 7,150 | 3,655 |
|  |  | 0,366 | 0,916 | 0,062 | 5,710 | 0,028 | 4,391 | 8,048 | 3,524 |
|  |  | 0,100 | 1,024 | 0,088 | 6,608 | 0,018 | 4,458 | 7,665 | 3,251 |
|  |  | 0,664 | 2,250 | 0,159 | 12,304 | 0,065 | 9,475 | 15,782 | 7,858 |
|  |  | 0,206 | 0,939 | 0,080 | 5,384 | 0,033 | 4,309 | 8,454 | 4,180 |
|  |  | 0,407 | 1,224 | 0,089 | 7,592 | 0,045 | 6,039 | 13,238 | 5,084 |
|  | *average* | *0,338* | *1,154* | *0,087* | *8,401* | *0,033* | *5,962* | *9,512* | *5,015* |
|  | *SD* | *0,195* | *0,474* | *0,031* | *2,611* | *0,017* | *2,409* | *3,195* | *2,179* |
|  |  |  |  |  |  |  |  |  |  |
| 3% FO, saline |  | 0,100 | 0,456 | 0,008 | 10,776 | 0,010 | 2,144 | 1,497 | 1,443 |
|  |  | 0,100 | 0,479 | 0,005 | 17,242 | 0,010 | 2,333 | 1,792 | 1,783 |
|  |  | 0,235 | 0,459 | 0,021 | 6,789 | 0,010 | 1,614 | 1,191 | 1,031 |
|  |  | 0,391 | 0,851 | 0,046 | 8,490 | 0,010 | 2,308 | 2,258 | 1,833 |
|  |  | 0,286 | 0,809 | 0,030 | 11,906 | 0,010 | 2,550 | 2,136 | 2,036 |
|  |  | 0,297 | 0,581 | 0,023 | 10,943 | 0,010 | 2,169 | 1,436 | 1,522 |
|  |  | 0,181 | 0,467 | 0,028 | 6,998 | 0,010 | 2,323 | 1,629 | 1,678 |
|  |  | 0,144 | 0,648 | 0,030 | 6,801 | 0,010 | 2,245 | 1,583 | 1,278 |
|  | *average* | *0,217* | *0,594* | *0,024* | *9,993* | *0,010* | *2,211* | *1,690* | *1,576* |
|  | *SD* | *0,104* | *0,161* | *0,013* | *3,574* | *0,000* | *0,271* | *0,358* | *0,325* |
|  |  |  |  |  |  |  |  |  |  |
| 3% FO, LPS |  | 0,448 | 1,615 | 0,063 | 8,670 | 0,010 | 5,094 | 7,857 | 3,280 |
|  |  | 0,627 | 1,508 | 0,066 | 8,182 | 0,037 | 4,861 | 7,645 | 3,535 |
|  |  | 0,703 | 1,840 | 0,087 | 8,773 | 0,034 | 6,664 | 9,299 | 4,787 |
|  |  | 1,023 | 2,147 | 0,102 | 8,270 | 0,047 | 6,266 | 9,605 | 4,352 |
|  |  | 0,100 | 1,158 | 0,056 | 9,152 | 0,045 | 5,605 | 7,768 | 4,114 |
|  |  | 0,322 | 1,112 | 0,035 | 7,356 | 0,025 | 4,709 | 6,647 | 4,132 |
|  |  | 0,574 | 1,205 | 0,063 | 8,417 | 0,010 | 5,494 | 7,833 | 3,860 |
|  | *average* | *0,543* | *1,512* | *0,067* | *8,403* | *0,030* | *5,528* | *8,093* | *4,009* |
|  | *SD* | *0,294* | *0,387* | *0,022* | *0,568* | *0,015* | *0,724* | *1,021* | *0,505* |

| **Liver** |  | 12(S)-HHTrE | 9(S)-HODE | 13(S)-HODE | EPA | AA | 15-deoxy-d-12,14-PGJ2 | 12(S)-HEPE | 5(S)-HEPE |
| --- | --- | --- | --- | --- | --- | --- | --- | --- | --- |
|  |  | ng/gram tissue weight | ng/gram tissue weight | ng/gram tissue weight | ng/gram tissue weight | ng/gram tissue weight | ng/gram tissue weight | ng/gram tissue weight | ng/gram tissue weight |
|  |  |  |  |  |  |  |  |  |  |
| ctrl, saline |  | 0,628 | 6,411 | 19,630 | 503,177 | 13141,592 | 0,100 | 4,776 | 0,528 |
|  |  | 3,933 | 3,466 | 14,922 | 347,803 | 18037,225 | 0,100 | 7,836 | 0,647 |
|  |  | 0,711 | 5,149 | 28,448 | 400,238 | 19092,792 | 0,100 | 4,160 | 0,784 |
|  |  | 0,955 | 1,744 | 13,850 | 224,174 | 12983,140 | 0,100 | 0,815 | 0,874 |
|  |  | 1,573 | 1,743 | 36,883 | 238,933 | 14834,071 | 0,100 | 0,852 | 0,100 |
|  |  | 1,347 | 1,637 | 13,330 | 210,141 | 15856,376 | 0,100 | 0,985 | 0,406 |
|  |  | 1,343 | 2,839 | 12,614 | 269,438 | 10494,276 | 0,100 | 1,593 | 0,220 |
|  |  | 1,307 | 2,350 | 9,654 | 293,004 | 12968,671 | 0,100 | 1,933 | 0,100 |
|  | *average* | 1,475 | 3,167 | 18,666 | 310,863 | 14676,018 | 0,100 | 2,869 | 0,457 |
|  | *SD* | 1,048 | 1,761 | 9,350 | 100,858 | 2874,671 | 0,000 | 2,516 | 0,301 |
|  |  |  |  |  |  |  |  |  |  |
| ctrl, LPS |  | 1,211 | 2,295 | 6,972 | 361,532 | 19412,792 | 0,100 | 0,450 | 0,100 |
|  |  | 3,401 | 2,458 | 10,035 | 545,279 | 36520,825 | 0,100 | 1,097 | 0,932 |
|  |  | 11,041 | 3,383 | 19,358 | 572,180 | 32656,290 | 0,100 | 1,430 | 0,405 |
|  |  | 6,024 | 3,282 | 13,059 | 302,298 | 24953,438 | 0,100 | 2,620 | 0,595 |
|  |  | 6,726 | 3,453 | 10,677 | 413,515 | 41276,370 | 0,100 | 1,231 | 0,745 |
|  |  | 9,726 | 6,304 | 19,705 | 361,824 | 36031,760 | 0,100 | 9,939 | 1,087 |
|  |  | 2,342 | 1,265 | 3,920 | 236,167 | 15508,533 | 0,100 | 1,022 | 0,277 |
|  |  | 2,541 | 3,108 | 9,242 | 457,415 | 20255,602 | 0,100 | 0,350 | 0,178 |
|  | *average* | *5,376* | *3,194* | *11,621* | *406,276* | *28326,951* | *0,100* | *2,267* | *0,540* |
|  | *SD* | *3,620* | *1,457* | *5,571* | *115,412* | *9511,163* | *0,000* | *3,177* | *0,361* |
|  |  |  |  |  |  |  |  |  |  |
| 1% FO, saline |  | 0,742 | 7,039 | 24,372 | 6373,580 | 8361,407 | 0,100 | 28,133 | 4,867 |
|  |  | 0,427 | 1,360 | 7,980 | 4843,170 | 5888,595 | 0,100 | 10,814 | 2,540 |
|  |  | 0,299 | 1,880 | 10,538 | 6699,457 | 6087,471 | 0,100 | 19,323 | 6,510 |
|  |  | 0,167 | 1,254 | 6,552 | 7339,819 | 7993,055 | 0,100 | 14,626 | 4,367 |
|  |  | 0,460 | 1,097 | 9,931 | 4998,036 | 5859,478 | 0,100 | 22,302 | 4,506 |
|  |  | 0,293 | 2,423 | 25,649 | 6179,098 | 8268,515 | 0,100 | 33,863 | 5,809 |
|  |  | 0,446 | 1,235 | 5,660 | 4902,358 | 4538,807 | 0,100 | 12,588 | 4,281 |
|  |  | 0,222 | 1,592 | 7,071 | 6139,973 | 5269,916 | 0,100 | 16,078 | 4,286 |
|  | *average* | *0,382* | *2,235* | *12,219* | *5934,436* | *6533,405* | *0,100* | *19,716* | *4,646* |
|  | *SD* | *0,181* | *1,988* | *8,069* | *924,909* | *1470,006* | *0,000* | *7,991* | *1,175* |
|  |  |  |  |  |  |  |  |  |  |
| 1% FO, LPS |  | 0,251 | 1,628 | 3,538 | 4205,207 | 8874,133 | 0,100 | 3,663 | 1,484 |
|  |  | 0,154 | 0,893 | 2,977 | 2794,616 | 5594,032 | 0,100 | 2,103 | 2,000 |
|  |  | 0,535 | 1,263 | 3,815 | 3294,152 | 5230,575 | 0,100 | 5,316 | 2,893 |
|  |  | 0,545 | 3,825 | 8,143 | 5583,109 | 10908,452 | 0,100 | 7,324 | 6,556 |
|  |  | 0,124 | 1,437 | 4,692 | 3706,745 | 4915,568 | 0,100 | 2,572 | 1,098 |
|  |  | 0,501 | 2,552 | 6,606 | 3540,770 | 8986,019 | 0,100 | 4,714 | 2,990 |
|  |  | 0,244 | 1,303 | 4,081 | 4130,384 | 7152,628 | 0,100 | 4,918 | 2,416 |
|  |  | 0,480 | 3,135 | 6,592 | 5186,353 | 8321,019 | 0,100 | 6,964 | 3,036 |
|  | *average* | *0,354* | *2,005* | *5,055* | *4055,167* | *7497,803* | *0,100* | *4,697* | *2,809* |
|  | *SD* | *0,178* | *1,044* | *1,834* | *940,858* | *2137,122* | *0,000* | *1,882* | *1,674* |
|  |  |  |  |  |  |  |  |  |  |
| 3% FO, saline |  | 0,150 | 1,401 | 3,502 | 2376,664 | 2207,219 | 0,100 | 6,619 | 4,069 |
|  |  | 0,150 | 0,973 | 3,407 | 3734,528 | 2502,808 | 0,100 | 5,857 | 3,407 |
|  |  | 0,150 | 22,076 | 44,363 | 6509,579 | 3440,678 | 0,100 | 17,270 | 8,489 |
|  |  | 0,145 | 1,435 | 5,906 | 6446,583 | 3999,304 | 0,100 | 46,533 | 7,948 |
|  |  | 0,366 | 1,309 | 5,069 | 7399,900 | 4527,400 | 0,100 | 14,233 | 5,976 |
|  |  | 0,150 | 2,268 | 5,594 | 12709,038 | 4105,046 | 0,100 | 12,706 | 9,160 |
|  |  | 0,534 | 0,816 | 4,489 | 5822,146 | 5254,654 | 0,100 | 18,188 | 6,231 |
|  |  | 0,089 | 0,773 | 4,400 | 7085,578 | 3593,796 | 0,100 | 16,638 | 4,922 |
|  | *average* | *0,217* | *3,881* | *9,591* | *6510,502* | *3703,863* | *0,100* | *17,255* | *6,275* |
|  | *SD* | *0,152* | *7,367* | *14,078* | *3042,934* | *1006,781* | *0,000* | *12,713* | *2,106* |
|  |  |  |  |  |  |  |  |  |  |
| 3% FO, LPS |  | 0,473 | 1,828 | 4,934 | 6334,703 | 10467,197 | 0,100 | 7,438 | 4,886 |
|  |  | 0,150 | 77,179 | 195,892 | 7797,761 | 11934,791 | 0,100 | 9,136 | 8,965 |
|  |  | 1,528 | 2,720 | 11,432 | 5979,774 | 9577,976 | 0,100 | 13,921 | 4,062 |
|  |  | 0,278 | 1,146 | 2,977 | 3267,749 | 4480,315 | 0,100 | 10,861 | 2,530 |
|  |  | 0,150 | 2,034 | 5,063 | 6804,360 | 6243,386 | 0,100 | 11,648 | 6,656 |
|  |  | 0,247 | 2,278 | 7,781 | 8536,160 | 10402,891 | 0,100 | 32,731 | 5,045 |
|  |  | 0,292 | 4,100 | 10,646 | 14597,285 | 14652,122 | 0,100 | 72,229 | 9,833 |
|  | *average* | *0,445* | *13,041* | *34,103* | *7616,827* | *9679,811* | *0,100* | *22,566* | *5,997* |
|  | *SD* | *0,490* | *28,297* | *71,410* | *3500,073* | *3410,909* | *0,000* | *23,487* | *2,641* |

| **Liver** |  | 20(S)-HETE | 15(S)-HETE | 11(S)-HETE | 12(S)-HETE | 11,12 EET | 8,9 EET | 5,6 EET | 5(S)-HETE | 14,15 EET | DHA |
| --- | --- | --- | --- | --- | --- | --- | --- | --- | --- | --- | --- |
|  |  | ng/gram tissue weight | ng/gram tissue weight | ng/gram tissue weight | ng/gram tissue weight | ng/gram tissue weight | ng/gram tissue weight | ng/gram tissue weight | ng/gram tissue weight | ng/gram tissue weight | ng/gram tissue weight |
|  |  |  |  |  |  |  |  |  |  |  |  |
| ctrl, saline |  | 0,800 | 0,933 | 0,358 | 8,944 | 0,431 | 0,300 | 3,622 | 0,386 | 0,808 | 10077,927 |
|  |  | 0,800 | 1,132 | 0,696 | 22,634 | 0,385 | 0,300 | 3,849 | 0,282 | 0,858 | 11527,558 |
|  |  | 0,800 | 1,467 | 0,469 | 12,664 | 0,720 | 0,300 | 6,363 | 0,308 | 0,680 | 15376,697 |
|  |  | 0,800 | 1,594 | 0,374 | 9,116 | 0,288 | 0,300 | 1,213 | 0,331 | 0,645 | 11510,272 |
|  |  | 0,800 | 2,526 | 0,548 | 26,278 | 0,523 | 0,300 | 3,283 | 0,299 | 0,258 | 10523,933 |
|  |  | 0,800 | 2,463 | 0,757 | 15,778 | 1,130 | 0,300 | 3,262 | 0,305 | 1,310 | 12758,786 |
|  |  | 0,800 | 1,591 | 0,312 | 16,169 | 0,227 | 0,300 | 1,286 | 0,204 | 0,589 | 9010,039 |
|  |  | 0,800 | 1,515 | 0,298 | 15,174 | 0,511 | 0,300 | 2,382 | 0,144 | 0,717 | 10183,046 |
|  | *average* | 0,800 | 1,652 | 0,476 | 15,845 | 0,527 | 0,300 | 3,158 | 0,282 | 0,733 | 11371,032 |
|  | *SD* | 0,000 | 0,569 | 0,176 | 6,077 | 0,287 | 0,000 | 1,642 | 0,075 | 0,296 | 1978,109 |
|  |  |  |  |  |  |  |  |  |  |  |  |
| ctrl, LPS |  | 3,102 | 0,737 | 0,401 | 1,773 | 0,854 | 0,300 | 6,811 | 0,246 | 0,986 | 16247,306 |
|  |  | 3,469 | 2,213 | 0,890 | 8,143 | 1,092 | 0,300 | 6,058 | 0,357 | 1,742 | 28257,166 |
|  |  | 3,833 | 2,736 | 1,241 | 14,241 | 0,797 | 0,300 | 8,166 | 0,333 | 1,340 | 22469,692 |
|  |  | 2,808 | 1,425 | 0,843 | 8,541 | 0,852 | 0,300 | 6,401 | 0,214 | 1,692 | 19837,037 |
|  |  | 4,760 | 2,123 | 1,212 | 5,628 | 1,473 | 0,300 | 10,120 | 0,526 | 2,855 | 34859,872 |
|  |  | 5,843 | 2,453 | 1,684 | 58,162 | 2,025 | 0,300 | 24,572 | 0,612 | 1,884 | 30468,450 |
|  |  | 1,363 | 0,737 | 0,430 | 3,914 | 0,147 | 0,300 | 2,877 | 0,186 | 0,648 | 11589,289 |
|  |  | 3,273 | 1,319 | 0,793 | 2,601 | 0,659 | 0,300 | 4,548 | 0,349 | 1,280 | 15772,763 |
|  | *average* | *3,557* | *1,718* | *0,937* | *12,875* | *0,987* | *0,300* | *8,694* | *0,353* | *1,553* | *22437,697* |
|  | *SD* | *1,332* | *0,771* | *0,431* | *18,730* | *0,562* | *0,000* | *6,774* | *0,149* | *0,668* | *8105,240* |
|  |  |  |  |  |  |  |  |  |  |  |  |
| 1% FO, saline |  | 0,800 | 0,585 | 0,182 | 4,424 | 0,523 | 0,300 | 1,106 | 0,173 | 0,636 | 25814,422 |
|  |  | 0,800 | 0,414 | 0,144 | 3,102 | 0,217 | 0,300 | 1,407 | 0,078 | 0,285 | 16984,700 |
|  |  | 0,800 | 0,286 | 0,133 | 2,211 | 0,189 | 0,300 | 1,211 | 0,107 | 0,388 | 20614,700 |
|  |  | 0,800 | 0,338 | 0,140 | 1,924 | 0,010 | 0,300 | 1,960 | 0,134 | 0,231 | 23850,865 |
|  |  | 0,800 | 0,310 | 0,129 | 4,346 | 0,179 | 0,300 | 2,467 | 0,108 | 0,097 | 14887,107 |
|  |  | 0,800 | 0,682 | 0,221 | 6,397 | 0,259 | 0,300 | 0,050 | 0,145 | 0,481 | 21688,786 |
|  |  | 0,800 | 0,159 | 0,104 | 1,602 | 0,128 | 0,300 | 0,050 | 0,096 | 0,270 | 18727,653 |
|  |  | 0,800 | 0,306 | 0,159 | 3,380 | 0,230 | 0,300 | 0,050 | 0,124 | 0,381 | 20159,893 |
|  | *average* | *0,800* | *0,385* | *0,152* | *3,423* | *0,217* | *0,300* | *1,038* | *0,121* | *0,346* | *20341,016* |
|  | *SD* | *0,000* | *0,171* | *0,036* | *1,596* | *0,146* | *0,000* | *0,925* | *0,030* | *0,165* | *3541,879* |
|  |  |  |  |  |  |  |  |  |  |  |  |
| 1% FO, LPS |  | 0,800 | 0,335 | 0,185 | 0,764 | 0,010 | 0,300 | 0,050 | 0,216 | 0,269 | 19816,641 |
|  |  | 0,800 | 0,205 | 0,110 | 0,469 | 0,161 | 0,300 | 1,823 | 0,039 | 0,243 | 17445,561 |
|  |  | 0,800 | 0,318 | 0,150 | 1,237 | 0,232 | 0,300 | 0,050 | 0,155 | 0,373 | 17254,415 |
|  |  | 0,800 | 0,496 | 0,248 | 1,384 | 0,742 | 0,300 | 3,928 | 0,193 | 0,849 | 35793,762 |
|  |  | 0,800 | 0,181 | 0,103 | 0,475 | 0,010 | 0,300 | 0,050 | 0,046 | 0,368 | 19206,109 |
|  |  | 0,800 | 0,485 | 0,282 | 0,915 | 0,194 | 0,300 | 1,136 | 0,192 | 0,378 | 26502,485 |
|  |  | 1,553 | 0,255 | 0,104 | 0,717 | 0,249 | 0,300 | 0,050 | 0,077 | 0,202 | 18552,250 |
|  |  | 1,859 | 0,327 | 0,235 | 1,005 | 0,214 | 0,300 | 0,050 | 0,201 | 0,377 | 26509,892 |
|  | *average* | *1,027* | *0,325* | *0,177* | *0,871* | *0,226* | *0,300* | *0,892* | *0,140* | *0,382* | *22635,139* |
|  | *SD* | *0,427* | *0,117* | *0,071* | *0,332* | *0,229* | *0,000* | *1,398* | *0,074* | *0,201* | *6495,883* |
|  |  |  |  |  |  |  |  |  |  |  |  |
| 3% FO, saline |  | 0,800 | 0,073 | 0,094 | 0,846 | 0,191 | 0,300 | 0,050 | 0,159 | 0,224 | 8271,015 |
|  |  | 0,800 | 0,165 | 0,093 | 0,644 | 0,010 | 0,300 | 0,050 | 0,076 | 0,100 | 12296,203 |
|  |  | 0,800 | 0,435 | 0,210 | 1,977 | 0,177 | 0,300 | 0,050 | 0,196 | 0,328 | 25915,215 |
|  |  | 0,800 | 0,211 | 0,113 | 3,476 | 0,010 | 0,300 | 0,050 | 0,244 | 0,277 | 14571,393 |
|  |  | 0,800 | 0,331 | 0,151 | 2,670 | 0,173 | 0,300 | 0,050 | 0,217 | 0,287 | 16872,099 |
|  |  | 0,800 | 0,255 | 0,159 | 1,251 | 0,010 | 0,300 | 0,050 | 0,269 | 0,100 | 20603,099 |
|  |  | 0,800 | 0,221 | 0,124 | 2,453 | 0,010 | 0,300 | 0,050 | 0,089 | 0,100 | 20800,105 |
|  |  | 0,800 | 0,187 | 0,106 | 1,522 | 0,010 | 0,300 | 0,050 | 0,042 | 0,100 | 14310,048 |
|  | *average* | *0,800* | *0,235* | *0,131* | *1,855* | *0,074* | *0,300* | *0,050* | *0,162* | *0,189* | *16704,897* |
|  | *SD* | *0,000* | *0,110* | *0,040* | *0,973* | *0,088* | *0,000* | *0,000* | *0,084* | *0,100* | *5576,465* |
|  |  |  |  |  |  |  |  |  |  |  |  |
| 3% FO, LPS |  | 0,800 | 0,451 | 0,244 | 0,756 | 0,156 | 0,300 | 4,321 | 0,496 | 0,845 | 23154,122 |
|  |  | 0,800 | 1,761 | 0,768 | 1,878 | 0,619 | 0,300 | 0,050 | 0,996 | 0,853 | 29739,835 |
|  |  | 0,800 | 0,628 | 0,333 | 2,811 | 0,174 | 0,300 | 0,050 | 0,165 | 0,243 | 19718,110 |
|  |  | 0,800 | 0,333 | 0,203 | 1,910 | 0,010 | 0,300 | 1,014 | 0,217 | 0,132 | 16624,612 |
|  |  | 0,800 | 0,298 | 0,170 | 0,998 | 0,010 | 0,300 | 0,050 | 0,382 | 0,364 | 33822,061 |
|  |  | 0,800 | 0,351 | 0,206 | 4,711 | 0,010 | 0,300 | 0,050 | 0,144 | 0,692 | 40603,041 |
|  |  | 3,415 | 0,622 | 0,307 | 6,322 | 0,465 | 0,300 | 4,692 | 0,204 | 0,100 | 59759,498 |
|  | *average* | *1,174* | *0,635* | *0,319* | *2,770* | *0,206* | *0,300* | *1,461* | *0,372* | *0,461* | *31917,326* |
|  | *SD* | *0,988* | *0,514* | *0,207* | *2,046* | *0,244* | *0,000* | *2,113* | *0,303* | *0,329* | *14823,851* |

| **Liver** |  | PGB2 | LTB4 | 14,15-DiHETrE | 11,12-DiHETrE | 5,6-DiHETrE | 17(S)-HDoHE | PGE3 | PGD3 | 13,14-dihydro-15-keto-PGD2 | 13,14-dihydro-15-keto-PGE2 | PGF2b |
| --- | --- | --- | --- | --- | --- | --- | --- | --- | --- | --- | --- | --- |
|  |  | ng/gram tissue weight | ng/gram tissue weight | ng/gram tissue weight | ng/gram tissue weight | ng/gram tissue weight | ng/gram tissue weight | ng/gram tissue weight | ng/gram tissue weight | ng/gram tissue weight | ng/gram tissue weight | ng/gram tissue weight |
|  |  |  |  |  |  |  |  |  |  |  |  |  |
| ctrl, saline |  | 0,050 | 0,040 | 5,024 | 1,630 | 0,069 | 2,343 | 0,100 | 0,200 | 0,020 | 0,511 | 0,100 |
|  |  | 0,050 | 0,040 | 7,085 | 3,109 | 0,152 | 1,596 | 0,100 | 0,200 | 0,020 | 0,100 | 0,100 |
|  |  | 0,050 | 0,040 | 6,730 | 2,855 | 0,133 | 2,178 | 0,100 | 0,200 | 0,020 | 0,395 | 0,100 |
|  |  | 0,050 | 0,040 | 6,730 | 2,432 | 0,101 | 1,323 | 0,100 | 0,200 | 0,020 | 0,350 | 0,100 |
|  |  | 0,050 | 0,040 | 5,245 | 2,260 | 0,076 | 2,247 | 0,100 | 0,200 | 0,020 | 0,934 | 0,100 |
|  |  | 0,050 | 0,040 | 24,517 | 8,973 | 0,108 | 3,310 | 0,100 | 0,200 | 0,020 | 0,359 | 0,100 |
|  |  | 0,050 | 0,040 | 2,676 | 1,113 | 0,029 | 3,109 | 0,100 | 0,200 | 0,020 | 0,100 | 0,100 |
|  |  | 0,050 | 0,040 | 4,070 | 1,448 | 0,018 | 2,479 | 0,100 | 0,200 | 0,020 | 0,100 | 0,100 |
|  | *average* | 0,050 | 0,040 | 7,760 | 2,978 | 0,086 | 2,323 | 0,100 | 0,200 | 0,020 | 0,356 | 0,100 |
|  | *SD* | 0,000 | 0,000 | 6,935 | 2,519 | 0,047 | 0,673 | 0,000 | 0,000 | 0,000 | 0,282 | 0,000 |
|  |  |  |  |  |  |  |  |  |  |  |  |  |
| ctrl, LPS |  | 0,050 | 0,040 | 7,886 | 2,308 | 0,077 | 1,040 | 0,100 | 0,200 | 0,020 | 0,528 | 0,100 |
|  |  | 0,050 | 0,040 | 11,477 | 4,434 | 0,103 | 1,645 | 0,100 | 0,200 | 0,020 | 0,100 | 0,100 |
|  |  | 0,050 | 0,040 | 9,694 | 3,261 | 0,146 | 4,304 | 0,100 | 0,200 | 0,020 | 0,735 | 0,100 |
|  |  | 0,050 | 0,040 | 11,961 | 4,374 | 0,178 | 1,753 | 0,100 | 0,200 | 0,020 | 0,331 | 0,100 |
|  |  | 0,198 | 0,040 | 13,926 | 4,808 | 0,257 | 2,444 | 0,100 | 0,200 | 0,020 | 0,291 | 0,100 |
|  |  | 0,050 | 0,040 | 13,280 | 5,420 | 0,257 | 2,942 | 0,100 | 0,200 | 0,020 | 0,795 | 0,100 |
|  |  | 0,050 | 0,040 | 3,754 | 1,551 | 0,025 | 0,949 | 0,100 | 0,200 | 0,020 | 0,311 | 0,100 |
|  |  | 0,050 | 0,040 | 7,197 | 2,479 | 0,038 | 1,254 | 0,100 | 0,200 | 0,020 | 0,100 | 0,100 |
|  | *average* | *0,068* | *0,040* | *9,897* | *3,579* | *0,135* | *2,042* | *0,100* | *0,200* | *0,020* | *0,399* | *0,100* |
|  | *SD* | *0,052* | *0,000* | *3,455* | *1,379* | *0,091* | *1,143* | *0,000* | *0,000* | *0,000* | *0,264* | *0,000* |
|  |  |  |  |  |  |  |  |  |  |  |  |  |
| 1% FO, saline |  | 0,050 | 0,154 | 2,285 | 1,066 | 0,042 | 4,499 | 0,100 | 0,200 | 0,020 | 0,100 | 0,100 |
|  |  | 0,050 | 0,185 | 1,910 | 0,831 | 0,026 | 2,115 | 0,100 | 0,200 | 0,020 | 0,430 | 0,100 |
|  |  | 0,050 | 0,133 | 1,908 | 0,885 | 0,033 | 4,335 | 0,100 | 0,200 | 0,020 | 0,100 | 0,100 |
|  |  | 0,050 | 0,270 | 2,990 | 1,330 | 0,020 | 2,925 | 0,100 | 0,200 | 0,020 | 0,100 | 0,100 |
|  |  | 0,050 | 0,201 | 3,486 | 1,389 | 0,024 | 4,414 | 0,100 | 0,200 | 0,020 | 0,100 | 0,100 |
|  |  | 0,050 | 0,313 | 3,892 | 1,662 | 0,075 | 6,696 | 0,100 | 0,200 | 0,020 | 0,100 | 0,100 |
|  |  | 0,050 | 0,199 | 2,080 | 0,934 | 0,017 | 1,820 | 0,100 | 0,200 | 0,020 | 0,100 | 0,100 |
|  |  | 0,050 | 0,212 | 1,719 | 0,633 | 0,011 | 4,540 | 0,100 | 0,200 | 0,020 | 0,100 | 0,100 |
|  | *average* | *0,050* | *0,208* | *2,534* | *1,091* | *0,031* | *3,918* | *0,100* | *0,200* | *0,020* | *0,141* | *0,100* |
|  | *SD* | *0,000* | *0,059* | *0,817* | *0,342* | *0,020* | *1,581* | *0,000* | *0,000* | *0,000* | *0,117* | *0,000* |
|  |  |  |  |  |  |  |  |  |  |  |  |  |
| 1% FO, LPS |  | 0,050 | 0,040 | 2,274 | 0,539 | 0,026 | 1,655 | 0,100 | 0,200 | 0,020 | 0,100 | 0,100 |
|  |  | 0,050 | 0,040 | 1,698 | 0,388 | 0,020 | 1,846 | 0,100 | 0,200 | 0,020 | 0,100 | 0,100 |
|  |  | 0,050 | 0,040 | 1,768 | 0,438 | 0,011 | 1,981 | 0,100 | 0,200 | 0,020 | 0,100 | 0,100 |
|  |  | 0,050 | 0,040 | 2,021 | 0,617 | 0,015 | 2,954 | 0,100 | 0,200 | 0,020 | 0,100 | 0,100 |
|  |  | 0,050 | 0,040 | 1,049 | 0,323 | 0,007 | 1,249 | 0,100 | 0,200 | 0,020 | 0,100 | 0,100 |
|  |  | 0,835 | 0,076 | 2,452 | 0,581 | 0,031 | 3,975 | 0,100 | 0,200 | 0,020 | 0,100 | 0,100 |
|  |  | 0,050 | 0,124 | 2,017 | 0,731 | 0,011 | 3,323 | 0,100 | 0,200 | 0,020 | 0,264 | 0,100 |
|  |  | 0,050 | 0,081 | 1,943 | 0,693 | 0,010 | 1,570 | 0,100 | 0,200 | 0,020 | 0,257 | 0,100 |
|  | *average* | *0,148* | *0,060* | *1,903* | *0,539* | *0,016* | *2,319* | *0,100* | *0,200* | *0,020* | *0,140* | *0,100* |
|  | *SD* | *0,277* | *0,031* | *0,424* | *0,146* | *0,009* | *0,974* | *0,000* | *0,000* | *0,000* | *0,074* | *0,000* |
|  |  |  |  |  |  |  |  |  |  |  |  |  |
| 3% FO, saline |  | 0,050 | 0,203 | 2,964 | 1,058 | 0,025 | 2,718 | 0,100 | 0,200 | 0,020 | 0,100 | 0,100 |
|  |  | 0,050 | 0,093 | 1,200 | 0,346 | 0,037 | 3,568 | 0,100 | 0,200 | 0,020 | 0,100 | 0,100 |
|  |  | 0,050 | 0,174 | 2,391 | 0,862 | 0,060 | 5,139 | 0,100 | 0,200 | 0,020 | 0,100 | 0,100 |
|  |  | 0,050 | 0,176 | 1,751 | 0,652 | 0,020 | 4,779 | 0,100 | 0,200 | 0,020 | 0,100 | 0,100 |
|  |  | 0,050 | 0,078 | 2,200 | 0,707 | 0,029 | 5,952 | 0,100 | 0,200 | 0,020 | 0,100 | 0,100 |
|  |  | 0,050 | 0,156 | 1,154 | 0,450 | 0,022 | 1,437 | 0,100 | 0,200 | 0,020 | 0,100 | 0,100 |
|  |  | 0,050 | 0,201 | 2,675 | 1,088 | 0,019 | 6,266 | 0,100 | 0,200 | 0,020 | 0,100 | 0,100 |
|  |  | 0,050 | 0,198 | 1,975 | 0,679 | 0,011 | 3,410 | 0,100 | 0,200 | 0,020 | 0,159 | 0,100 |
|  | *average* | *0,050* | *0,160* | *2,039* | *0,730* | *0,028* | *4,158* | *0,100* | *0,200* | *0,020* | *0,107* | *0,100* |
|  | *SD* | *0,000* | *0,049* | *0,653* | *0,264* | *0,015* | *1,665* | *0,000* | *0,000* | *0,000* | *0,021* | *0,000* |
|  |  |  |  |  |  |  |  |  |  |  |  |  |
| 3% FO, LPS |  | 0,050 | 0,091 | 2,036 | 0,591 | 0,051 | 3,019 | 0,100 | 0,200 | 0,020 | 0,100 | 0,100 |
|  |  | 0,050 | 0,121 | 1,748 | 0,564 | 0,210 | 4,586 | 0,100 | 0,200 | 0,020 | 0,100 | 0,100 |
|  |  | 0,050 | 0,147 | 1,831 | 0,662 | 0,030 | 8,459 | 0,100 | 0,200 | 0,020 | 0,100 | 0,100 |
|  |  | 0,050 | 0,073 | 1,661 | 0,484 | 0,043 | 2,514 | 0,100 | 0,200 | 0,020 | 0,100 | 0,100 |
|  |  | 0,050 | 0,040 | 1,371 | 0,485 | 0,020 | 2,429 | 0,100 | 0,200 | 0,020 | 0,100 | 0,100 |
|  |  | 0,050 | 0,074 | 1,433 | 0,549 | 0,022 | 6,275 | 0,100 | 0,200 | 0,020 | 0,100 | 0,100 |
|  |  | 0,050 | 0,289 | 3,265 | 1,687 | 0,059 | 6,577 | 0,100 | 0,200 | 0,020 | 0,100 | 0,100 |
|  | *average* | *0,050* | *0,119* | *1,906* | *0,717* | *0,062* | *4,837* | *0,100* | *0,200* | *0,020* | *0,100* | *0,100* |
|  | *SD* | *0,000* | *0,083* | *0,641* | *0,432* | *0,067* | *2,337* | *0,000* | *0,000* | *0,000* | *0,000* | *0,000* |

| **Liver** |  | 8-iso-PGF2a | PGF2a | 10(S)-17(S)-DiHDoHE | 19,20-DiHoPE | TBXB3 | TBXB2 | 8,9-DiHETrE | 13,14-dihydro-15-keto-PGF2a | lipoxin A4 | PGE2 | PGD2 |
| --- | --- | --- | --- | --- | --- | --- | --- | --- | --- | --- | --- | --- |
|  |  | ng/gram tissue weight | ng/gram tissue weight | ng/gram tissue weight | ng/gram tissue weight | ng/gram tissue weight | ng/gram tissue weight | ng/gram tissue weight | ng/gram tissue weight | ng/gram tissue weight | ng/gram tissue weight | ng/gram tissue weight |
|  |  |  |  |  |  |  |  |  |  |  |  |  |
| ctrl, saline |  | 0,020 | 0,836 | 0,200 | 3,079 | 0,010 | 0,331 | 0,951 | 2,551 | 0,066 | 0,480 | 0,060 |
|  |  | 0,020 | 6,037 | 0,200 | 3,425 | 0,010 | 3,268 | 1,719 | 3,338 | 0,131 | 0,356 | 0,700 |
|  |  | 0,020 | 1,298 | 0,200 | 3,639 | 0,010 | 0,892 | 1,229 | 3,626 | 0,328 | 0,370 | 0,819 |
|  |  | 0,020 | 1,527 | 0,200 | 3,326 | 0,010 | 1,082 | 1,146 | 3,273 | 0,191 | 0,417 | 0,610 |
|  |  | 0,020 | 1,343 | 0,200 | 3,425 | 0,010 | 1,637 | 1,301 | 2,646 | 0,210 | 1,248 | 0,809 |
|  |  | 0,092 | 1,797 | 0,200 | 8,046 | 0,010 | 1,091 | 1,675 | 4,400 | 0,122 | 1,893 | 0,817 |
|  |  | 0,068 | 2,471 | 0,200 | 1,961 | 0,010 | 0,518 | 0,513 | 3,702 | 0,230 | 0,643 | 0,520 |
|  |  | 0,059 | 2,831 | 0,200 | 2,964 | 0,010 | 0,512 | 0,452 | 3,525 | 0,040 | 1,371 | 0,308 |
|  | *average* | 0,040 | 2,267 | 0,200 | 3,733 | 0,010 | 1,166 | 1,123 | 3,382 | 0,165 | 0,847 | 0,580 |
|  | *SD* | 0,029 | 1,655 | 0,000 | 1,818 | 0,000 | 0,947 | 0,472 | 0,594 | 0,094 | 0,581 | 0,275 |
|  |  |  |  |  |  |  |  |  |  |  |  |  |
| ctrl, LPS |  | 0,048 | 1,770 | 0,200 | 5,279 | 0,010 | 1,070 | 1,865 | 3,630 | 0,258 | 0,472 | 0,534 |
|  |  | 0,071 | 4,457 | 0,200 | 9,899 | 0,010 | 1,327 | 2,951 | 4,973 | 0,040 | 1,727 | 1,233 |
|  |  | 0,148 | 8,314 | 0,200 | 5,674 | 0,010 | 3,275 | 2,625 | 6,212 | 0,040 | 3,354 | 1,775 |
|  |  | 0,119 | 7,214 | 0,200 | 8,964 | 0,010 | 2,874 | 2,419 | 6,883 | 0,040 | 2,393 | 1,147 |
|  |  | 0,091 | 5,936 | 0,200 | 10,921 | 0,010 | 2,357 | 3,363 | 6,229 | 0,203 | 1,965 | 1,245 |
|  |  | 0,153 | 13,636 | 0,200 | 6,396 | 0,010 | 3,835 | 2,995 | 8,745 | 0,176 | 4,779 | 2,025 |
|  |  | 0,112 | 3,001 | 0,200 | 2,125 | 0,010 | 0,869 | 0,578 | 3,175 | 0,234 | 1,196 | 0,564 |
|  |  | 0,113 | 2,530 | 0,200 | 4,393 | 0,010 | 0,838 | 1,504 | 5,285 | 0,157 | 1,316 | 0,579 |
|  | *average* | *0,107* | *5,857* | *0,200* | *6,707* | *0,010* | *2,056* | *2,287* | *5,642* | *0,144* | *2,150* | *1,138* |
|  | *SD* | *0,036* | *3,897* | *0,000* | *2,990* | *0,000* | *1,184* | *0,921* | *1,795* | *0,091* | *1,366* | *0,563* |
|  |  |  |  |  |  |  |  |  |  |  |  |  |
| 1% FO, saline |  | 0,099 | 0,885 | 0,200 | 6,541 | 0,010 | 0,433 | 0,404 | 1,055 | 0,234 | 0,305 | 0,741 |
|  |  | 0,038 | 0,841 | 0,200 | 6,104 | 0,010 | 0,622 | 0,433 | 1,229 | 0,159 | 0,265 | 0,508 |
|  |  | 0,062 | 0,785 | 0,200 | 6,958 | 0,010 | 0,197 | 0,383 | 1,179 | 0,271 | 0,176 | 0,410 |
|  |  | 0,109 | 0,732 | 0,200 | 9,373 | 0,010 | 0,199 | 0,448 | 1,523 | 0,309 | 0,086 | 0,596 |
|  |  | 0,044 | 0,836 | 0,200 | 10,875 | 0,010 | 0,693 | 0,527 | 1,135 | 0,231 | 0,248 | 0,413 |
|  |  | 0,043 | 0,981 | 0,200 | 11,960 | 0,010 | 0,353 | 0,601 | 1,869 | 0,040 | 0,157 | 0,513 |
|  |  | 0,063 | 1,180 | 0,200 | 6,673 | 0,010 | 0,444 | 0,356 | 1,853 | 0,040 | 0,305 | 0,564 |
|  |  | 0,052 | 1,570 | 0,200 | 5,837 | 0,010 | 0,212 | 0,175 | 1,656 | 0,170 | 0,308 | 0,442 |
|  | *average* | *0,064* | *0,976* | *0,200* | *8,040* | *0,010* | *0,394* | *0,416* | *1,437* | *0,182* | *0,231* | *0,523* |
|  | *SD* | *0,027* | *0,277* | *0,000* | *2,362* | *0,000* | *0,192* | *0,126* | *0,330* | *0,100* | *0,083* | *0,111* |
|  |  |  |  |  |  |  |  |  |  |  |  |  |
| 1% FO, LPS |  | 0,055 | 0,910 | 0,200 | 7,370 | 0,010 | 0,307 | 0,451 | 2,619 | 0,040 | 0,274 | 0,124 |
|  |  | 0,058 | 0,908 | 0,200 | 7,690 | 0,010 | 0,256 | 0,166 | 1,773 | 0,040 | 0,169 | 0,073 |
|  |  | 0,083 | 0,876 | 0,200 | 8,071 | 0,010 | 0,330 | 0,226 | 2,657 | 0,040 | 0,760 | 0,132 |
|  |  | 0,062 | 1,051 | 0,200 | 8,178 | 0,010 | 0,177 | 0,317 | 2,377 | 0,201 | 0,658 | 0,215 |
|  |  | 0,057 | 0,897 | 0,200 | 5,813 | 0,010 | 0,323 | 0,123 | 0,200 | 0,040 | 0,326 | 0,060 |
|  |  | 0,060 | 1,190 | 0,200 | 12,193 | 0,010 | 0,501 | 0,400 | 4,594 | 0,040 | 0,327 | 0,292 |
|  |  | 0,051 | 0,780 | 0,200 | 8,466 | 0,010 | 0,248 | 0,303 | 3,817 | 0,140 | 0,332 | 0,082 |
|  |  | 0,079 | 0,801 | 0,200 | 5,992 | 0,010 | 0,102 | 0,502 | 1,675 | 0,112 | 0,215 | 0,228 |
|  | *average* | *0,063* | *0,927* | *0,200* | *7,972* | *0,010* | *0,281* | *0,311* | *2,464* | *0,082* | *0,382* | *0,151* |
|  | *SD* | *0,011* | *0,134* | *0,000* | *1,969* | *0,000* | *0,118* | *0,135* | *1,345* | *0,062* | *0,211* | *0,085* |
|  |  |  |  |  |  |  |  |  |  |  |  |  |
| 3% FO, saline |  | 0,020 | 0,933 | 0,200 | 9,597 | 0,010 | 0,100 | 0,217 | 0,200 | 0,259 | 0,078 | 0,202 |
|  |  | 0,020 | 0,809 | 0,200 | 6,769 | 0,010 | 0,150 | 0,106 | 0,200 | 0,199 | 0,119 | 0,313 |
|  |  | 0,020 | 1,004 | 0,200 | 8,384 | 0,010 | 0,525 | 0,265 | 0,200 | 0,365 | 0,303 | 0,371 |
|  |  | 0,020 | 1,128 | 0,200 | 8,590 | 0,010 | 0,431 | 0,177 | 0,200 | 0,357 | 0,111 | 0,321 |
|  |  | 0,020 | 1,362 | 0,200 | 12,577 | 0,010 | 0,528 | 0,265 | 1,014 | 0,261 | 0,255 | 0,376 |
|  |  | 0,020 | 1,347 | 0,200 | 7,169 | 0,010 | 0,171 | 0,050 | 0,626 | 0,574 | 0,126 | 0,259 |
|  |  | 0,061 | 1,167 | 0,200 | 13,990 | 0,010 | 0,579 | 0,277 | 1,518 | 0,327 | 0,225 | 0,563 |
|  |  | 0,033 | 1,057 | 0,200 | 7,808 | 0,010 | 0,248 | 0,242 | 0,584 | 0,232 | 0,177 | 0,060 |
|  | *average* | *0,027* | *1,101* | *0,200* | *9,361* | *0,010* | *0,342* | *0,200* | *0,568* | *0,322* | *0,174* | *0,308* |
|  | *SD* | *0,015* | *0,192* | *0,000* | *2,600* | *0,000* | *0,195* | *0,083* | *0,485* | *0,118* | *0,080* | *0,146* |
|  |  |  |  |  |  |  |  |  |  |  |  |  |
| 3% FO, LPS |  | 0,020 | 0,818 | 0,200 | 15,094 | 0,010 | 0,272 | 0,333 | 1,078 | 0,576 | 0,732 | 0,200 |
|  |  | 0,020 | 0,995 | 0,200 | 14,140 | 0,010 | 0,213 | 0,524 | 1,306 | 0,040 | 0,401 | 0,497 |
|  |  | 0,072 | 1,541 | 0,200 | 16,135 | 0,010 | 0,769 | 0,293 | 2,269 | 0,147 | 0,957 | 0,233 |
|  |  | 0,075 | 0,851 | 0,200 | 12,051 | 0,010 | 0,311 | 0,412 | 0,763 | 0,099 | 0,502 | 0,150 |
|  |  | 0,078 | 0,506 | 0,200 | 11,614 | 0,010 | 0,144 | 0,289 | 1,617 | 0,299 | 0,186 | 0,060 |
|  |  | 0,089 | 0,961 | 0,200 | 12,104 | 0,010 | 0,373 | 0,196 | 2,776 | 0,206 | 0,691 | 0,060 |
|  |  | 0,081 | 1,296 | 0,200 | 19,641 | 0,010 | 0,370 | 1,122 | 1,888 | 0,155 | 0,284 | 0,060 |
|  | *average* | *0,062* | *0,995* | *0,200* | *14,397* | *0,010* | *0,350* | *0,453* | *1,671* | *0,218* | *0,536* | *0,180* |
|  | *SD* | *0,029* | *0,337* | *0,000* | *2,875* | *0,000* | *0,202* | *0,313* | *0,700* | *0,178* | *0,272* | *0,157* |

| **Liver** |  | | Leukotriene E4 | n-acetyl leukotriene E4 | Leukotriene D4 | 17 keto- 4(z), 7(z), 10(z), 13 (z), 15 (E), 19(z)-DHA | 12,13-DiHOME | 9,10-DiHOME | 9,12,13-TriHOME | 9,10,13-TriHOME | UK1 | UK2 | UK3 | UK4 | UK5 |
| --- | --- | --- | --- | --- | --- | --- | --- | --- | --- | --- | --- | --- | --- | --- | --- |
|  |  | | ng/gram tissue weight | ng/gram tissue weight | ng/gram tissue weight | ng/gram tissue weight | ng/gram tissue weight | ng/gram tissue weight | RR | RR | RR | RR | RR | RR | RR |
|  |  | |  |  |  |  |  |  |  |  |  |  |  |  |  |
| ctrl, saline |  | | 1,000 | 0,200 | 0,500 | 14,786 | 814,823 | 281,343 | 6554,518 | 5771,786 | 0,386 | 0,085 | 0,070 | 0,095 | 0,075 |
|  |  | | 1,000 | 0,200 | 0,500 | 9,696 | 819,729 | 282,894 | 3494,966 | 3413,908 | 0,287 | 0,069 | 0,091 | 0,036 | 0,080 |
|  |  | | 1,000 | 0,200 | 0,500 | 15,187 | 899,607 | 378,327 | 5567,185 | 5072,185 | 0,964 | 0,059 | 0,080 | 0,015 | 0,098 |
|  | |  | 1,000 | 0,200 | 0,500 | 8,100 | 653,597 | 243,520 | 6169,370 | 5562,427 | 0,248 | 0,029 | 0,021 | 0,009 | 0,063 |
|  |  | | 1,000 | 0,200 | 0,500 | 5,134 | 759,996 | 263,218 | 5565,355 | 4149,539 | 0,431 | 0,051 | 0,063 | 0,019 | 0,053 |
|  |  | | 1,000 | 0,200 | 0,500 | 16,616 | 1411,185 | 333,350 | 3414,312 | 2817,447 | 0,363 | 0,085 | 0,064 | 0,032 | 0,095 |
|  |  | | 1,000 | 0,200 | 0,500 | 1,200 | 355,310 | 131,867 | 4823,586 | 4949,314 | 1,340 | 0,028 | 0,025 | 0,019 | 0,059 |
|  |  | | 1,000 | 0,200 | 0,500 | 12,922 | 422,266 | 155,317 | 3911,034 | 3586,081 | 0,607 | 0,039 | 0,046 | 0,014 | 0,044 |
|  | *average* | | 1,000 | 0,200 | 0,500 | 10,455 | 767,064 | 258,729 | 4937,541 | 4415,336 | 0,578 | 0,056 | 0,058 | 0,030 | 0,071 |
|  | *SD* | | 0,000 | 0,000 | 0,000 | 5,415 | 324,888 | 82,814 | 1218,346 | 1081,452 | 0,383 | 0,023 | 0,025 | 0,028 | 0,020 |
|  |  | |  |  |  |  |  |  |  |  |  |  |  |  |  |
| ctrl, LPS |  | | 1,000 | 0,613 | 0,500 | 5,553 | 419,390 | 217,164 | 6427,816 | 6231,542 | 1,521 | 0,062 | 0,106 | 0,033 | 0,070 |
|  |  | | 1,000 | 0,200 | 0,500 | 26,077 | 671,537 | 322,018 | 2668,982 | 2460,232 | 0,372 | 0,093 | 0,119 | 0,034 | 0,106 |
|  |  | | 1,000 | 0,441 | 0,500 | 21,888 | 427,660 | 301,602 | 3853,820 | 3288,734 | 0,480 | 0,114 | 0,135 | 0,043 | 0,144 |
|  |  | | 1,000 | 0,685 | 0,500 | 46,779 | 841,082 | 396,517 | 3000,733 | 2832,061 | 0,462 | 0,177 | 0,156 | 0,091 | 0,168 |
|  |  | | 1,000 | 0,380 | 0,500 | 89,822 | 874,774 | 359,558 | 3783,421 | 3437,385 | 0,357 | 0,133 | 0,186 | 0,098 | 0,169 |
|  |  | | 1,000 | 0,200 | 0,500 | 66,523 | 676,856 | 401,146 | 4886,460 | 4735,765 | 0,773 | 0,093 | 0,223 | 0,091 | 0,235 |
|  |  | | 1,000 | 0,583 | 0,500 | 7,241 | 253,454 | 118,324 | 2767,973 | 2697,970 | 0,198 | 0,026 | 0,033 | 0,008 | 0,041 |
|  |  | | 1,000 | 0,392 | 0,500 | 7,956 | 295,535 | 202,227 | 2888,012 | 2646,376 | 0,164 | 0,066 | 0,074 | 0,022 | 0,088 |
|  | *average* | | *1,000* | *0,437* | *0,500* | *33,980* | *557,536* | *289,820* | *3784,652* | *3541,258* | *0,541* | *0,096* | *0,129* | *0,052* | *0,128* |
|  | *SD* | | *0,000* | *0,182* | *0,000* | *31,053* | *240,634* | *101,544* | *1303,946* | *1305,076* | *0,439* | *0,047* | *0,061* | *0,035* | *0,063* |
|  |  | |  |  |  |  |  |  |  |  |  |  |  |  |  |
| 1% FO, saline |  | | 1,000 | 0,200 | 0,500 | 55,506 | 685,787 | 303,616 | 11853,396 | 11737,747 | 1,005 | 0,698 | 0,145 | 0,307 | 0,206 |
|  |  | | 1,000 | 0,200 | 0,500 | 60,796 | 575,009 | 250,991 | 4797,853 | 4506,012 | 0,315 | 0,054 | 0,060 | 0,033 | 0,075 |
|  |  | | 1,000 | 0,200 | 0,500 | 82,117 | 521,593 | 228,090 | 2781,003 | 2335,568 | 0,236 | 0,053 | 0,058 | 0,024 | 0,077 |
|  |  | | 1,000 | 0,200 | 0,500 | 86,687 | 664,616 | 238,125 | 2348,309 | 2209,898 | 0,165 | 0,076 | 0,073 | 0,026 | 0,139 |
|  |  | | 1,000 | 0,200 | 0,500 | 60,435 | 702,222 | 288,931 | 2885,019 | 2579,695 | 0,174 | 0,070 | 0,069 | 0,019 | 0,083 |
|  |  | | 1,000 | 0,200 | 0,500 | 84,874 | 883,829 | 357,426 | 4105,242 | 3120,837 | 0,357 | 0,076 | 0,067 | 0,026 | 0,102 |
|  |  | | 1,000 | 0,200 | 0,500 | 12,476 | 422,913 | 237,941 | 5086,260 | 5088,799 | 0,302 | 0,027 | 0,021 | 0,012 | 0,017 |
|  |  | | 1,000 | 0,200 | 0,500 | 15,934 | 452,615 | 222,262 | 2661,293 | 2595,083 | 0,197 | 0,041 | 0,014 | 0,016 | 0,037 |
|  | *average* | | *1,000* | *0,200* | *0,500* | *57,353* | *613,573* | *265,923* | *4564,797* | *4271,705* | *0,344* | *0,137* | *0,063* | *0,058* | *0,092* |
|  | *SD* | | *0,000* | *0,000* | *0,000* | *29,241* | *151,622* | *46,964* | *3121,374* | *3195,141* | *0,276* | *0,227* | *0,040* | *0,101* | *0,059* |
|  |  | |  |  |  |  |  |  |  |  |  |  |  |  |  |
| 1% FO, LPS |  | | 1,000 | 0,727 | 0,500 | 23,998 | 245,160 | 171,325 | 1820,687 | 1865,549 | 0,213 | 0,048 | 0,103 | 0,019 | 0,090 |
|  |  | | 1,000 | 0,435 | 0,500 | 25,894 | 307,015 | 153,515 | 1482,028 | 1324,211 | 0,170 | 0,031 | 0,059 | 0,016 | 0,059 |
|  |  | | 1,000 | 0,200 | 0,500 | 22,072 | 314,488 | 156,918 | 1560,815 | 1588,660 | 0,252 | 0,041 | 0,064 | 0,025 | 0,069 |
|  |  | | 1,000 | 0,200 | 0,500 | 59,767 | 238,531 | 210,422 | 2474,603 | 2589,125 | 0,280 | 0,067 | 0,166 | 0,036 | 0,148 |
|  |  | | 1,000 | 0,200 | 0,500 | 20,098 | 251,248 | 192,890 | 1821,990 | 1533,337 | 0,372 | 0,035 | 0,071 | 0,028 | 0,065 |
|  |  | | 1,000 | 0,552 | 0,500 | 9,400 | 508,104 | 244,738 | 2764,065 | 2605,627 | 0,234 | 0,038 | 0,067 | 0,017 | 0,058 |
|  |  | | 1,000 | 0,200 | 0,500 | 36,635 | 284,969 | 273,984 | 2336,309 | 2306,806 | 0,199 | 0,044 | 0,066 | 0,013 | 0,045 |
|  |  | | 1,000 | 0,200 | 0,500 | 6,711 | 256,856 | 236,724 | 2327,693 | 2265,147 | 0,137 | 0,072 | 0,078 | 0,021 | 0,068 |
|  | *average* | | *1,000* | *0,339* | *0,500* | *25,572* | *300,796* | *205,064* | *2073,524* | *2009,808* | *0,232* | *0,047* | *0,084* | *0,022* | *0,075* |
|  | *SD* | | *0,000* | *0,208* | *0,000* | *16,706* | *88,488* | *44,105* | *464,651* | *498,463* | *0,072* | *0,015* | *0,036* | *0,007* | *0,032* |
|  |  | |  |  |  |  |  |  |  |  |  |  |  |  |  |
| 3% FO, saline |  | | 1,000 | 0,200 | 0,500 | 8,534 | 438,348 | 171,360 | 1773,197 | 1871,279 | 0,068 | 0,019 | 0,029 | 0,021 | 0,031 |
|  |  | | 1,000 | 0,200 | 0,500 | 23,267 | 212,663 | 120,066 | 1762,710 | 1794,191 | 0,081 | 0,021 | 0,045 | 0,010 | 0,037 |
|  |  | | 1,000 | 0,200 | 0,500 | 11,994 | 604,878 | 355,934 | 17339,791 | 19785,334 | 2,540 | 0,053 | 0,047 | 0,025 | 0,044 |
|  |  | | 1,000 | 0,200 | 0,500 | 1,200 | 343,221 | 161,285 | 2498,089 | 2468,065 | 0,129 | 0,034 | 0,046 | 0,007 | 0,029 |
|  |  | | 1,000 | 0,200 | 0,500 | 29,846 | 451,147 | 154,721 | 2284,315 | 2225,409 | 0,151 | 0,025 | 0,032 | 0,016 | 0,023 |
|  |  | | 1,000 | 0,200 | 0,500 | 7,765 | 305,035 | 155,305 | 1870,489 | 2020,846 | 0,168 | 0,044 | 0,044 | 0,018 | 0,050 |
|  |  | | 1,000 | 0,200 | 0,500 | 20,329 | 440,640 | 163,860 | 2304,994 | 2134,149 | 0,188 | 0,017 | 0,017 | 0,014 | 0,024 |
|  |  | | 1,000 | 0,200 | 0,500 | 11,443 | 370,233 | 136,193 | 3502,301 | 3449,654 | 0,147 | 0,019 | 0,020 | 0,012 | 0,030 |
|  | *average* | | *1,000* | *0,200* | *0,500* | *14,297* | *395,771* | *177,341* | *4166,986* | *4468,616* | *0,434* | *0,029* | *0,035* | *0,015* | *0,034* |
|  | *SD* | | *0,000* | *0,000* | *0,000* | *9,407* | *116,982* | *74,009* | *5352,515* | *6210,755* | *0,852* | *0,013* | *0,012* | *0,006* | *0,009* |
|  |  | |  |  |  |  |  |  |  |  |  |  |  |  |  |
| 3% FO, LPS |  | | 1,000 | 0,200 | 0,500 | 63,081 | 426,931 | 208,548 | 2754,034 | 2696,037 | 0,279 | 0,039 | 0,050 | 0,022 | 0,061 |
|  |  | | 1,000 | 0,200 | 0,500 | 60,538 | 685,518 | 484,507 | 35257,653 | 36660,738 | 14,929 | 0,702 | 0,573 | 0,374 | 0,483 |
|  |  | | 1,000 | 0,200 | 0,500 | 51,286 | 437,390 | 238,117 | 3630,916 | 3173,530 | 0,389 | 0,062 | 0,097 | 0,021 | 0,123 |
|  |  | | 1,000 | 0,200 | 0,500 | 30,841 | 460,290 | 188,192 | 2655,744 | 2429,247 | 0,189 | 0,041 | 0,030 | 0,006 | 0,030 |
|  |  | | 1,000 | 0,200 | 0,500 | 47,784 | 345,558 | 177,944 | 2527,015 | 2535,840 | 0,210 | 0,052 | 0,044 | 0,015 | 0,075 |
|  |  | | 1,000 | 0,200 | 0,500 | 60,564 | 430,562 | 217,365 | 3570,391 | 2822,197 | 0,276 | 0,061 | 0,052 | 0,026 | 0,065 |
|  |  | | 1,000 | 0,200 | 0,500 | 88,818 | 620,886 | 441,132 | 5386,343 | 4409,374 | 0,521 | 0,049 | 0,074 | 0,035 | 0,085 |
|  | *average* | | *1,000* | *0,200* | *0,500* | *57,559* | *486,734* | *279,401* | *7968,871* | *7818,137* | *2,399* | *0,144* | *0,131* | *0,071* | *0,132* |
|  | *SD* | | *0,000* | *0,000* | *0,000* | *17,674* | *120,629* | *127,417* | *12073,036* | *12735,945* | *5,526* | *0,246* | *0,196* | *0,134* | *0,157* |

| **Liver** |  | epea | dhea | aea | 2-ag | dle | pea | oea | sea |
| --- | --- | --- | --- | --- | --- | --- | --- | --- | --- |
|  |  | ng/gram tissue weight | ng/gram tissue weight | ng/gram tissue weight | ng/gram tissue weight | ng/gram tissue weight | ng/gram tissue weight | ng/gram tissue weight | ng/gram tissue weight |
|  |  |  |  |  |  |  |  |  |  |
| ctrl, saline |  | 0,100 | 0,811 | 1,732 | 10699,006 | 0,339 | 78,312 | 41,401 | 41,980 |
|  |  | 0,100 | 0,946 | 0,512 | 10132,700 | 0,131 | 78,213 | 29,340 | 43,906 |
|  |  | 0,100 | 0,669 | 0,302 | 10159,606 | 0,224 | 94,539 | 30,370 | 50,893 |
|  |  | 0,100 | 1,228 | 0,316 | 9599,750 | 0,270 | 105,561 | 33,696 | 65,162 |
|  |  | 0,100 | 0,599 | 0,299 | 13899,340 | 0,264 | 87,579 | 40,031 | 52,154 |
|  |  | 0,100 | 0,753 | 0,441 | 9588,983 | 0,171 | 109,866 | 35,096 | 53,791 |
|  |  | 0,100 | 1,040 | 0,100 | 12334,877 | 0,226 | 73,290 | 26,514 | 45,001 |
|  |  | 0,100 | 1,101 | 0,100 | 7374,612 | 0,170 | 63,368 | 25,102 | 35,049 |
|  | *average* | 0,100 | 0,893 | 0,475 | 10473,609 | 0,224 | 86,341 | 32,694 | 48,492 |
|  | *SD* | 0,000 | 0,221 | 0,528 | 1949,928 | 0,067 | 16,125 | 5,963 | 9,114 |
|  |  |  |  |  |  |  |  |  |  |
| ctrl, LPS |  | 0,100 | 6,787 | 4,229 | 6315,880 | 0,743 | 95,073 | 91,383 | 36,815 |
|  |  | 0,100 | 3,847 | 4,542 | 8929,360 | 0,469 | 97,341 | 66,744 | 35,098 |
|  |  | 0,100 | 6,269 | 6,029 | 8086,361 | 0,752 | 83,806 | 105,498 | 26,876 |
|  |  | 0,100 | 5,524 | 4,640 | 7600,673 | 0,610 | 73,218 | 79,694 | 27,440 |
|  |  | 0,100 | 7,804 | 6,366 | 8086,049 | 1,301 | 110,614 | 106,191 | 28,961 |
|  |  | 0,100 | 3,099 | 3,153 | 6963,921 | 0,583 | 68,176 | 55,240 | 29,161 |
|  |  | 0,100 | 2,523 | 1,453 | 6931,483 | 0,195 | 45,948 | 44,783 | 22,699 |
|  |  | 0,100 | 6,013 | 5,066 | 7297,037 | 0,770 | 81,978 | 96,595 | 28,275 |
|  | *average* | *0,100* | *5,233* | *4,435* | *7526,345* | *0,678* | *82,019* | *80,766* | *29,416* |
|  | *SD* | *0,000* | *1,875* | *1,572* | *827,116* | *0,315* | *19,987* | *23,193* | *4,537* |
|  |  |  |  |  |  |  |  |  |  |
| 1% FO, saline |  | 0,100 | 3,804 | 0,100 | 1577,208 | 0,128 | 128,819 | 33,690 | 77,386 |
|  |  | 0,100 | 2,260 | 0,100 | 1706,428 | 0,120 | 93,411 | 26,074 | 56,877 |
|  |  | 0,100 | 2,561 | 0,100 | 2153,822 | 0,062 | 95,250 | 22,174 | 49,185 |
|  |  | 0,100 | 2,324 | 0,100 | 2012,882 | 0,105 | 94,415 | 27,164 | 45,439 |
|  |  | 0,100 | 1,925 | 0,100 | 2627,862 | 0,168 | 88,588 | 24,704 | 42,746 |
|  |  | 0,100 | 3,223 | 0,100 | 2024,794 | 0,089 | 93,502 | 29,038 | 46,980 |
|  |  | 0,100 | 2,893 | 0,100 | 1798,123 | 0,182 | 97,713 | 34,239 | 38,612 |
|  |  | 0,100 | 3,827 | 0,100 | 2209,431 | 0,113 | 122,748 | 35,302 | 63,949 |
|  | *average* | *0,100* | *2,852* | *0,100* | *2013,819* | *0,121* | *101,806* | *29,048* | *52,647* |
|  | *SD* | *0,000* | *0,714* | *0,000* | *330,947* | *0,039* | *15,103* | *4,872* | *12,810* |
|  |  |  |  |  |  |  |  |  |  |
| 1% FO, LPS |  | 22,311 | 21,522 | 2,812 | 2176,361 | 0,780 | 88,899 | 98,085 | 40,600 |
|  |  | 3,656 | 10,519 | 0,479 | 1941,052 | 0,186 | 44,352 | 38,496 | 18,601 |
|  |  | 6,398 | 10,392 | 0,786 | 1728,767 | 0,157 | 53,677 | 42,405 | 27,208 |
|  |  | 11,356 | 14,745 | 1,946 | 1711,244 | 0,446 | 69,739 | 63,502 | 24,432 |
|  |  | 10,809 | 16,008 | 1,380 | 1943,535 | 0,327 | 62,863 | 44,789 | 23,386 |
|  |  | 13,101 | 23,418 | 1,996 | 1984,613 | 0,755 | 103,050 | 76,485 | 34,566 |
|  |  | 14,491 | 15,146 | 1,440 | 1909,038 | 0,378 | 82,636 | 62,513 | 36,056 |
|  |  | 7,133 | 7,978 | 0,616 | 1611,287 | 0,261 | 46,026 | 46,737 | 18,872 |
|  | *average* | *11,157* | *14,966* | *1,432* | *1875,737* | *0,411* | *68,905* | *59,127* | *27,965* |
|  | *SD* | *5,789* | *5,413* | *0,800* | *181,577* | *0,240* | *21,212* | *20,403* | *8,221* |
|  |  |  |  |  |  |  |  |  |  |
| 3% FO, saline |  | 5,519 | 7,600 | 0,306 | 1120,447 | 0,128 | 133,322 | 31,132 | 62,315 |
|  |  | 4,334 | 8,062 | 0,306 | 1291,833 | 0,099 | 125,828 | 21,840 | 59,276 |
|  |  | 10,067 | 7,053 | 3,970 | 1076,376 | 1,179 | 114,078 | 23,712 | 57,058 |
|  |  | 3,197 | 3,336 | 0,100 | 1364,455 | 0,102 | 110,571 | 18,718 | 48,800 |
|  |  | 10,182 | 5,240 | 0,522 | 1626,723 | 0,125 | 122,319 | 28,215 | 56,229 |
|  |  | 4,699 | 2,856 | 0,100 | 1592,169 | 0,067 | 103,408 | 15,429 | 49,366 |
|  |  | 21,161 | 11,605 | 7,231 | 1656,698 | 2,065 | 133,418 | 25,958 | 67,237 |
|  |  | 13,096 | 8,136 | 0,100 | 1865,986 | 0,139 | 151,589 | 21,945 | 76,943 |
|  | *average* | *9,032* | *6,736* | *1,579* | *1449,336* | *0,488* | *124,317* | *23,369* | *59,653* |
|  | *SD* | *6,015* | *2,857* | *2,635* | *279,730* | *0,739* | *15,329* | *5,065* | *9,302* |
|  |  |  |  |  |  |  |  |  |  |
| 3% FO, LPS |  | 16,250 | 30,982 | 2,132 | 1407,362 | 0,286 | 96,335 | 62,568 | 31,557 |
|  |  | 20,884 | 26,979 | 1,980 | 1297,671 | 0,328 | 92,558 | 56,056 | 27,451 |
|  |  | 21,987 | 32,595 | 2,407 | 1330,852 | 0,342 | 131,197 | 74,505 | 36,308 |
|  |  | 10,640 | 14,491 | 0,818 | 1506,820 | 0,157 | 56,438 | 40,660 | 22,381 |
|  |  | 15,140 | 17,018 | 0,923 | 1417,405 | 0,332 | 67,747 | 38,959 | 27,070 |
|  |  | 8,608 | 19,263 | 0,700 | 1295,841 | 0,109 | 52,092 | 57,065 | 30,124 |
|  |  | 11,201 | 20,329 | 0,873 | 1270,733 | 0,216 | 72,342 | 47,207 | 31,762 |
|  | *average* | *14,959* | *23,094* | *1,405* | *1360,955* | *0,253* | *81,244* | *53,860* | *29,522* |
|  | *SD* | *5,153* | *7,081* | *0,732* | *85,545* | *0,093* | *27,622* | *12,634* | *4,413* |

| **Ileum** |  | 12(S)-HHTrE | 9(S)-HODE | 13(S)-HODE | EPA | AA | 15-deoxy-d-12,14-PGJ2 | 12(S)-HEPE | 5(S)-HEPE |
| --- | --- | --- | --- | --- | --- | --- | --- | --- | --- |
|  |  | ng/gram tissue weight | ng/gram tissue weight | ng/gram tissue weight | ng/gram tissue weight | ng/gram tissue weight | ng/gram tissue weight | ng/gram tissue weight | ng/gram tissue weight |
|  |  |  |  |  |  |  |  |  |  |
| ctrl, saline |  | 148,801 | 40,157 | 152,035 | 156,058 | 35122,002 | 0,100 | 6,204 | 0,724 |
|  |  | 171,881 | 55,533 | 351,902 | 182,218 | 40794,342 | 0,654 | 5,695 | 1,351 |
|  |  | 92,807 | 33,588 | 101,845 | 96,021 | 26677,011 | 1,096 | 2,283 | 0,390 |
|  |  | 52,440 | 58,467 | 112,439 | 146,872 | 39521,689 | 0,549 | 4,582 | 0,230 |
|  |  | 153,264 | 72,595 | 140,115 | 158,512 | 41558,845 | 0,376 | 6,460 | 0,587 |
|  |  | 746,167 | 53,438 | 162,334 | 134,425 | 53363,881 | 1,058 | 3,403 | 1,179 |
|  |  | 656,660 | 139,612 | 154,333 | 469,664 | 54400,944 | 0,543 | 4,933 | 0,304 |
|  |  | 1187,682 | 589,185 | 497,158 | 1953,449 | 119206,744 | 6,669 | 12,953 | 0,591 |
|  | *average* | *401,213* | *130,322* | *209,020* | *412,153* | *51330,682* | *1,381* | *5,814* | *0,669* |
|  | *SD* | *413,577* | *188,274* | *140,112* | *633,486* | *28877,081* | *2,162* | *3,210* | *0,404* |
|  |  |  |  |  |  |  |  |  |  |
| ctrl, LPS |  | 172,007 | 171,203 | 211,485 | 163,516 | 60036,869 | 1,299 | 4,688 | 0,323 |
|  |  | 119,689 | 105,897 | 121,615 | 219,183 | 47135,671 | 0,975 | 1,981 | 1,045 |
|  |  | 115,903 | 124,040 | 194,125 | 411,220 | 98749,783 | 0,218 | 5,323 | 0,100 |
|  |  | 132,538 | 294,896 | 253,169 | 149,946 | 30005,439 | 0,930 | 3,512 | 1,214 |
|  |  | 84,393 | 85,784 | 129,331 | 130,125 | 32743,159 | 0,941 | 3,749 | 0,496 |
|  |  | 329,950 | 595,173 | 469,084 | 593,784 | 65978,137 | 0,901 | 7,997 | 1,062 |
|  |  | 1447,132 | 967,527 | 696,983 | 816,013 | 101988,486 | 1,532 | 11,403 | 1,048 |
|  | *average* | *343,087* | *334,931* | *296,542* | *354,827* | *62376,792* | *0,971* | *5,522* | *0,755* |
|  | *SD* | *493,479* | *330,484* | *211,459* | *264,714* | *29065,656* | *0,408* | *3,193* | *0,439* |
|  |  |  |  |  |  |  |  |  |  |
| 1% FO, saline |  | 126,378 | 98,576 | 144,074 | 8134,395 | 23726,169 | 1,328 | 76,840 | 8,256 |
|  |  | 50,326 | 45,469 | 70,514 | 8642,389 | 26041,338 | 0,289 | 28,200 | 3,370 |
|  |  | 113,905 | 55,874 | 89,253 | 6719,877 | 18643,172 | 2,921 | 70,192 | 1,921 |
|  |  | 85,624 | 63,621 | 88,068 | 7603,909 | 20981,825 | 0,824 | 34,217 | 2,181 |
|  |  | 93,093 | 102,547 | 140,802 | 5210,864 | 19085,903 | 2,689 | 87,686 | 3,067 |
|  |  | 72,998 | 66,064 | 106,649 | 6139,639 | 18239,259 | 1,153 | 56,120 | 3,162 |
|  |  | 301,270 | 493,168 | 357,989 | 14495,886 | 44818,170 | 0,771 | 183,730 | 15,941 |
|  |  | 155,369 | 275,896 | 223,341 | 8962,717 | 26834,451 | 0,451 | 135,620 | 7,921 |
|  | *average* | *124,870* | *150,152* | *152,586* | *8238,710* | *24796,286* | *1,303* | *84,075* | *5,727* |
|  | *SD* | *78,347* | *157,118* | *95,937* | *2831,811* | *8748,382* | *0,988* | *52,421* | *4,808* |
|  |  |  |  |  |  |  |  |  |  |
| 1% FO, LPS |  | 148,483 | 436,070 | 374,328 | 6788,266 | 24324,783 | 0,290 | 90,822 | 9,048 |
|  |  | 111,801 | 304,912 | 264,292 | 7657,474 | 31689,444 | 0,336 | 55,972 | 6,294 |
|  |  | 164,215 | 472,624 | 577,065 | 7989,239 | 29007,452 | 2,734 | 192,964 | 4,497 |
|  |  | 235,599 | 419,871 | 403,406 | 9087,906 | 39747,824 | 0,778 | 144,260 | 10,400 |
|  |  | 155,582 | 575,548 | 686,273 | 9767,535 | 37679,470 | 0,509 | 193,312 | 4,850 |
|  |  | 221,081 | 487,410 | 487,195 | 24236,140 | 91786,530 | 0,394 | 205,776 | 15,016 |
|  |  | 194,223 | 569,503 | 392,232 | 8578,676 | 31117,529 | 0,701 | 93,874 | 8,260 |
|  |  | 123,488 | 383,558 | 314,138 | 9101,430 | 49800,264 | 0,345 | 110,841 | 9,789 |
|  | *average* | *169,309* | *456,187* | *437,366* | *10400,833* | *41894,162* | *0,761* | *135,978* | *8,519* |
|  | *SD* | *44,321* | *91,195* | *139,518* | *5668,733* | *21610,671* | *0,817* | *56,467* | *3,429* |
|  |  |  |  |  |  |  |  |  |  |
| 3% FO, saline |  | 105,173 | 102,600 | 116,169 | 6858,632 | 21942,568 | 0,100 | 99,002 | 12,897 |
|  |  | 96,171 | 103,096 | 117,426 | 9865,861 | 26198,280 | 1,854 | 205,125 | 6,501 |
|  |  | 78,719 | 68,582 | 52,467 | 11477,681 | 29885,626 | 0,124 | 62,331 | 14,358 |
|  |  | 107,128 | 145,504 | 132,738 | 12669,946 | 31732,929 | 1,380 | 142,289 | 24,772 |
|  |  | 114,537 | 177,741 | 154,284 | 14022,252 | 31066,665 | 0,471 | 171,093 | 10,829 |
|  |  | 96,949 | 99,939 | 92,974 | 9554,069 | 24608,523 | 0,171 | 226,199 | 9,092 |
|  |  | 225,730 | 205,880 | 183,108 | 35773,459 | 84149,974 | 0,503 | 299,961 | 31,257 |
|  |  | 221,439 | 186,711 | 187,510 | 20055,474 | 31771,317 | 1,023 | 258,728 | 8,702 |
|  | *average* | *130,731* | *136,257* | *129,584* | *15034,672* | *35169,485* | *0,703* | *183,091* | *14,801* |
|  | *SD* | *58,268* | *49,758* | *45,443* | *9244,119* | *20121,651* | *0,650* | *80,344* | *8,689* |
|  |  |  |  |  |  |  |  |  |  |
| 3% FO, LPS |  | 338,278 | 324,497 | 303,596 | 9590,108 | 34838,159 | 2,447 | 288,418 | 23,796 |
|  |  | 1093,385 | 237,671 | 216,045 | 16711,041 | 41509,547 | 0,100 | 133,920 | 9,389 |
|  |  | 251,381 | 282,206 | 241,239 | 11200,892 | 32872,611 | 0,584 | 211,607 | 10,687 |
|  |  | 155,608 | 461,558 | 300,307 | 14265,197 | 33095,880 | 0,476 | 155,961 | 10,920 |
|  |  | 879,994 | 346,318 | 262,998 | 14815,466 | 45389,820 | 1,613 | 119,044 | 10,450 |
|  |  | 174,781 | 682,927 | 570,469 | 15783,155 | 37668,271 | 0,389 | 328,859 | 27,572 |
|  |  | 254,358 | 201,826 | 140,082 | 15912,810 | 35309,484 | 1,360 | 117,001 | 7,684 |
|  | *average* | *449,684* | *362,429* | *290,676* | *14039,810* | *37240,539* | *0,996* | *193,544* | *14,357* |
|  | *SD* | *376,709* | *164,336* | *135,402* | *2651,874* | *4665,040* | *0,840* | *85,649* | *7,890* |

| **Ileum** |  | 20(S)-HETE | 15(S)-HETE | 11(S)-HETE | 12(S)-HETE | 11,12 EET | 8,9 EET | 5,6 EET | 5(S)-HETE | 14,15 EET | DHA | PGB2 |
| --- | --- | --- | --- | --- | --- | --- | --- | --- | --- | --- | --- | --- |
|  |  | ng/gram tissue weight | ng/gram tissue weight | ng/gram tissue weight | ng/gram tissue weight | ng/gram tissue weight | ng/gram tissue weight | ng/gram tissue weight | ng/gram tissue weight | ng/gram tissue weight | ng/gram tissue weight | ng/gram tissue weight |
|  |  |  |  |  |  |  |  |  |  |  |  |  |
| ctrl, saline |  | 4,297 | 57,796 | 52,591 | 297,859 | 0,903 | 2,035 | 0,761 | 1,443 | 0,296 | 7965,781 | 0,050 |
|  |  | 2,999 | 71,734 | 48,650 | 355,026 | 0,650 | 4,315 | 1,770 | 0,757 | 0,559 | 9814,300 | 0,050 |
|  |  | 1,517 | 26,545 | 24,474 | 112,038 | 0,770 | 2,312 | 0,050 | 0,609 | 0,599 | 6500,582 | 0,050 |
|  |  | 4,692 | 29,452 | 22,107 | 167,955 | 1,253 | 6,017 | 2,092 | 0,783 | 1,316 | 10004,159 | 0,050 |
|  |  | 5,540 | 66,405 | 60,595 | 337,724 | 0,592 | 1,283 | 0,050 | 0,913 | 0,464 | 11356,214 | 0,050 |
|  |  | 4,295 | 37,256 | 32,107 | 178,546 | 1,751 | 7,612 | 0,843 | 1,063 | 1,339 | 15773,274 | 0,050 |
|  |  | 8,270 | 131,260 | 167,571 | 321,746 | 1,695 | 7,661 | 0,050 | 1,727 | 2,033 | 8564,640 | 0,050 |
|  |  | 8,332 | 219,040 | 235,407 | 823,037 | 4,096 | 8,563 | 3,544 | 1,618 | 6,217 | 28371,689 | 0,050 |
|  | *average* | *4,993* | *79,936* | *80,438* | *324,241* | *1,464* | *4,975* | *1,145* | *1,114* | *1,603* | *12293,830* | *0,050* |
|  | *SD* | *2,370* | *65,406* | *78,064* | *220,814* | *1,154* | *2,877* | *1,247* | *0,427* | *1,954* | *7061,712* | *0,000* |
|  |  |  |  |  |  |  |  |  |  |  |  |  |
| ctrl, LPS |  | 6,449 | 57,017 | 54,815 | 209,057 | 0,834 | 4,796 | 4,390 | 1,680 | 1,349 | 12934,456 | 0,050 |
|  |  | 11,473 | 43,969 | 64,027 | 89,083 | 0,379 | 3,786 | 1,890 | 1,789 | 0,804 | 12003,814 | 0,050 |
|  |  | 5,169 | 40,926 | 41,901 | 208,658 | 1,810 | 5,603 | 0,050 | 0,480 | 0,774 | 20429,063 | 0,050 |
|  |  | 9,796 | 46,586 | 57,824 | 155,995 | 1,496 | 4,122 | 2,500 | 0,897 | 0,853 | 6738,417 | 0,050 |
|  |  | 6,498 | 38,048 | 36,173 | 179,788 | 0,872 | 1,720 | 0,050 | 0,680 | 0,632 | 6837,792 | 0,050 |
|  |  | 4,913 | 169,369 | 199,781 | 582,837 | 1,208 | 4,987 | 4,115 | 2,293 | 0,920 | 10751,583 | 0,050 |
|  |  | 12,479 | 199,592 | 231,094 | 730,231 | 2,015 | 11,195 | 8,526 | 2,744 | 1,668 | 20784,802 | 0,050 |
|  | *average* | *8,111* | *85,072* | *97,945* | *307,950* | *1,231* | *5,173* | *3,074* | *1,509* | *1,000* | *12925,704* | *0,050* |
|  | *SD* | *3,095* | *68,725* | *81,319* | *245,269* | *0,582* | *2,932* | *2,960* | *0,853* | *0,370* | *5758,874* | *0,000* |
|  |  |  |  |  |  |  |  |  |  |  |  |  |
| 1% FO, saline |  | 5,996 | 27,601 | 27,607 | 132,702 | 1,235 | 4,254 | 0,050 | 0,687 | 0,439 | 20876,311 | 0,050 |
|  |  | 2,254 | 14,164 | 15,418 | 53,802 | 0,613 | 1,945 | 1,327 | 0,666 | 0,647 | 20729,786 | 0,050 |
|  |  | 4,420 | 40,131 | 39,284 | 164,281 | 0,010 | 5,539 | 0,050 | 0,735 | 0,527 | 20538,101 | 0,050 |
|  |  | 3,476 | 27,979 | 28,750 | 65,887 | 1,014 | 5,356 | 0,050 | 0,829 | 0,220 | 24036,751 | 0,050 |
|  |  | 3,852 | 49,904 | 50,430 | 196,462 | 0,507 | 6,028 | 0,050 | 1,202 | 1,505 | 17881,650 | 0,050 |
|  |  | 3,545 | 33,089 | 28,953 | 120,761 | 0,548 | 2,487 | 0,050 | 0,710 | 0,249 | 17093,664 | 0,050 |
|  |  | 11,965 | 106,587 | 137,926 | 273,648 | 0,741 | 2,190 | 0,050 | 1,704 | 0,247 | 40108,168 | 0,050 |
|  |  | 6,067 | 49,923 | 56,792 | 154,847 | 0,184 | 1,322 | 0,050 | 0,780 | 0,100 | 23370,039 | 0,050 |
|  | *average* | *5,197* | *43,672* | *48,145* | *145,299* | *0,606* | *3,640* | *0,210* | *0,914* | *0,492* | *23079,309* | *0,050* |
|  | *SD* | *3,023* | *28,122* | *38,650* | *70,692* | *0,401* | *1,863* | *0,451* | *0,362* | *0,447* | *7276,754* | *0,000* |
|  |  |  |  |  |  |  |  |  |  |  |  |  |
| 1% FO, LPS |  | 3,629 | 84,214 | 114,502 | 152,047 | 0,551 | 1,072 | 0,050 | 2,765 | 0,259 | 19729,780 | 0,050 |
|  |  | 5,077 | 57,052 | 79,039 | 104,976 | 0,396 | 1,809 | 0,050 | 1,355 | 0,376 | 22128,600 | 0,050 |
|  |  | 5,230 | 120,342 | 119,653 | 470,100 | 0,605 | 3,656 | 0,050 | 2,179 | 1,150 | 24556,142 | 0,050 |
|  |  | 3,392 | 123,172 | 145,706 | 354,329 | 0,523 | 1,834 | 3,308 | 3,067 | 0,668 | 29431,772 | 0,050 |
|  |  | 4,718 | 110,701 | 112,812 | 439,615 | 0,731 | 1,796 | 0,050 | 2,198 | 0,100 | 29716,928 | 0,050 |
|  |  | 7,502 | 83,111 | 77,234 | 339,666 | 1,279 | 4,538 | 2,194 | 2,114 | 1,081 | 56979,433 | 0,050 |
|  |  | 3,875 | 60,560 | 91,512 | 142,170 | 0,374 | 1,443 | 0,050 | 0,851 | 0,415 | 29988,086 | 0,050 |
|  |  | 4,029 | 53,249 | 69,156 | 193,584 | 0,755 | 1,807 | 0,050 | 1,830 | 1,020 | 31383,496 | 0,050 |
|  | *average* | *4,681* | *86,550* | *101,202* | *274,561* | *0,652* | *2,244* | *0,725* | *2,045* | *0,634* | *30489,279* | *0,050* |
|  | *SD* | *1,324* | *28,631* | *26,239* | *143,394* | *0,289* | *1,196* | *1,285* | *0,713* | *0,407* | *11497,601* | *0,000* |
|  |  |  |  |  |  |  |  |  |  |  |  |  |
| 3% FO, saline |  | 1,637 | 17,878 | 22,292 | 79,199 | 0,496 | 2,107 | 1,336 | 0,367 | 0,555 | 22359,695 | 0,050 |
|  |  | 3,073 | 32,829 | 31,920 | 152,988 | 0,010 | 1,544 | 0,050 | 0,351 | 0,100 | 28524,511 | 0,050 |
|  |  | 2,824 | 15,476 | 21,472 | 39,091 | 0,402 | 0,853 | 0,050 | 0,476 | 0,250 | 25667,733 | 0,050 |
|  |  | 4,681 | 30,515 | 34,584 | 89,288 | 0,769 | 0,300 | 0,050 | 0,010 | 0,100 | 35283,720 | 0,050 |
|  |  | 2,163 | 33,022 | 40,596 | 127,968 | 0,279 | 0,300 | 0,050 | 0,218 | 0,453 | 34892,834 | 0,050 |
|  |  | 8,341 | 25,498 | 26,695 | 105,285 | 0,357 | 0,986 | 0,050 | 0,241 | 0,274 | 28987,723 | 0,050 |
|  |  | 8,004 | 47,137 | 52,093 | 196,869 | 3,109 | 10,045 | 3,281 | 1,364 | 3,637 | 59537,946 | 0,050 |
|  |  | 6,887 | 48,485 | 50,477 | 189,611 | 0,389 | 0,300 | 0,050 | 0,010 | 0,514 | 37018,651 | 0,050 |
|  | *average* | *4,701* | *31,355* | *35,016* | *122,537* | *0,726* | *2,054* | *0,615* | *0,380* | *0,735* | *34034,102* | *0,050* |
|  | *SD* | *2,698* | *12,049* | *11,869* | *55,066* | *0,986* | *3,294* | *1,168* | *0,431* | *1,186* | *11487,646* | *0,000* |
|  |  |  |  |  |  |  |  |  |  |  |  |  |
| 3% FO, LPS |  | 8,963 | 38,460 | 48,280 | 130,059 | 0,774 | 2,189 | 1,569 | 0,391 | 0,421 | 30200,976 | 0,050 |
|  |  | 9,489 | 30,351 | 27,607 | 127,384 | 0,550 | 1,364 | 1,398 | 0,360 | 0,494 | 40828,961 | 0,050 |
|  |  | 7,407 | 44,340 | 55,686 | 148,880 | 0,398 | 1,732 | 1,572 | 0,717 | 0,708 | 35663,818 | 0,050 |
|  |  | 6,103 | 53,850 | 78,647 | 111,714 | 0,276 | 0,690 | 0,050 | 0,338 | 0,439 | 40242,814 | 0,050 |
|  |  | 5,264 | 38,678 | 53,620 | 144,444 | 0,702 | 2,012 | 0,050 | 0,439 | 0,446 | 37609,679 | 0,050 |
|  |  | 6,097 | 79,936 | 88,797 | 302,986 | 0,517 | 0,300 | 1,867 | 2,331 | 0,714 | 38865,327 | 0,050 |
|  |  | 4,981 | 28,557 | 44,588 | 118,351 | 0,260 | 0,300 | 0,050 | 0,417 | 0,317 | 38414,160 | 0,050 |
|  | *average* | *6,900* | *44,882* | *56,746* | *154,831* | *0,497* | *1,227* | *0,937* | *0,713* | *0,505* | *37403,676* | *0,050* |
|  | *SD* | *1,773* | *17,639* | *20,762* | *66,646* | *0,199* | *0,798* | *0,841* | *0,725* | *0,150* | *3603,221* | *0,000* |

| **Ileum** |  | LTB4 | 14,15-DiHETrE | 11,12-DiHETrE | 5,6-DiHETrE | 17(S)-HDoHE | PGE3 | PGD3 | 13,14-dihydro-15-keto-PGD2 | 13,14-dihydro-15-keto-PGE2 | PGF2b | 8-iso-PGF2a | PGF2a |
| --- | --- | --- | --- | --- | --- | --- | --- | --- | --- | --- | --- | --- | --- |
|  |  | ng/gram tissue weight | ng/gram tissue weight | ng/gram tissue weight | ng/gram tissue weight | ng/gram tissue weight | ng/gram tissue weight | ng/gram tissue weight | ng/gram tissue weight | ng/gram tissue weight | ng/gram tissue weight | ng/gram tissue weight | ng/gram tissue weight |
|  |  |  |  |  |  |  |  |  |  |  |  |  |  |
| ctrl, saline |  | 0,392 | 1,720 | 1,520 | 1,788 | 74,644 | 0,030 | 0,200 | 0,415 | 141,308 | 2,818 | 2,262 | 136,476 |
|  |  | 1,146 | 0,927 | 0,511 | 1,066 | 149,533 | 0,030 | 0,200 | 0,174 | 89,381 | 2,637 | 2,595 | 117,329 |
|  |  | 1,668 | 0,582 | 0,523 | 0,781 | 50,430 | 0,030 | 0,200 | 0,065 | 22,796 | 0,234 | 0,810 | 56,907 |
|  |  | 0,199 | 1,299 | 0,757 | 1,276 | 88,168 | 0,030 | 0,200 | 0,145 | 83,648 | 0,409 | 0,752 | 47,460 |
|  |  | 0,417 | 1,474 | 0,994 | 0,913 | 123,543 | 0,030 | 0,200 | 0,256 | 100,146 | 0,927 | 2,300 | 154,166 |
|  |  | 0,148 | 1,097 | 1,061 | 1,945 | 91,117 | 0,030 | 0,200 | 0,067 | 52,643 | 1,031 | 0,944 | 66,597 |
|  |  | 0,481 | 0,879 | 0,702 | 0,316 | 105,719 | 7,779 | 1,646 | 0,272 | 70,781 | 5,251 | 2,876 | 265,862 |
|  |  | 0,456 | 2,080 | 1,701 | 0,505 | 319,641 | 13,579 | 13,341 | 0,442 | 78,317 | 10,568 | 7,182 | 564,375 |
|  | *average* | *0,613* | *1,257* | *0,971* | *1,074* | *125,349* | *2,692* | *2,023* | *0,229* | *79,877* | *2,984* | *2,465* | *176,147* |
|  | *SD* | *0,523* | *0,489* | *0,443* | *0,576* | *84,058* | *5,167* | *4,601* | *0,144* | *34,588* | *3,486* | *2,087* | *171,991* |
|  |  |  |  |  |  |  |  |  |  |  |  |  |  |
| ctrl, LPS |  | 0,162 | 1,148 | 1,215 | 2,113 | 108,181 | 5,480 | 0,200 | 0,229 | 81,400 | 1,575 | 1,706 | 83,821 |
|  |  | 0,294 | 1,086 | 1,093 | 1,240 | 45,668 | 3,866 | 0,200 | 1,098 | 229,933 | 1,600 | 1,948 | 68,493 |
|  |  | 0,184 | 2,397 | 2,814 | 3,027 | 84,457 | 6,457 | 0,200 | 0,068 | 16,467 | 1,213 | 1,410 | 59,937 |
|  |  | 0,466 | 0,948 | 0,935 | 1,011 | 95,969 | 6,360 | 0,200 | 0,400 | 120,387 | 2,029 | 1,612 | 86,460 |
|  |  | 0,072 | 0,961 | 0,873 | 0,861 | 87,956 | 2,282 | 0,200 | 0,247 | 104,424 | 1,691 | 1,151 | 57,304 |
|  |  | 0,221 | 0,732 | 0,828 | 0,448 | 212,560 | 15,863 | 5,548 | 0,482 | 109,460 | 5,591 | 4,395 | 231,832 |
|  |  | 0,151 | 0,888 | 0,868 | 0,871 | 272,298 | 27,274 | 0,436 | 0,859 | 97,469 | 7,468 | 3,856 | 231,236 |
|  | *average* | *0,222* | *1,166* | *1,232* | *1,367* | *129,584* | *9,655* | *0,998* | *0,483* | *108,506* | *3,024* | *2,297* | *117,012* |
|  | *SD* | *0,128* | *0,559* | *0,711* | *0,894* | *81,293* | *8,901* | *2,009* | *0,369* | *63,549* | *2,467* | *1,283* | *78,997* |
|  |  |  |  |  |  |  |  |  |  |  |  |  |  |
| 1% FO, saline |  | 1,515 | 0,904 | 0,612 | 0,916 | 277,206 | 28,874 | 31,445 | 0,020 | 9,506 | 0,794 | 0,721 | 40,754 |
|  |  | 0,040 | 0,552 | 0,506 | 0,950 | 143,507 | 7,335 | 0,200 | 0,037 | 7,012 | 0,564 | 0,413 | 22,815 |
|  |  | 0,040 | 0,458 | 0,355 | 0,519 | 195,097 | 25,545 | 14,937 | 0,083 | 17,059 | 1,266 | 1,037 | 58,077 |
|  |  | 0,040 | 0,596 | 0,384 | 0,529 | 129,917 | 19,110 | 16,192 | 0,033 | 11,233 | 0,956 | 0,820 | 54,369 |
|  |  | 0,040 | 0,576 | 0,488 | 0,747 | 235,412 | 35,372 | 48,868 | 0,069 | 12,953 | 1,354 | 1,040 | 53,276 |
|  |  | 0,078 | 0,781 | 0,513 | 0,632 | 220,749 | 24,894 | 19,972 | 0,095 | 21,629 | 0,873 | 0,608 | 42,466 |
|  |  | 0,275 | 0,683 | 0,815 | 0,350 | 493,226 | 224,435 | 159,200 | 0,130 | 27,648 | 3,397 | 1,950 | 113,798 |
|  |  | 0,130 | 0,545 | 0,711 | 0,789 | 468,641 | 157,858 | 103,669 | 0,099 | 7,000 | 3,461 | 1,479 | 84,451 |
|  | *average* | *0,270* | *0,637* | *0,548* | *0,679* | *270,469* | *65,428* | *49,310* | *0,071* | *14,255* | *1,583* | *1,009* | *58,751* |
|  | *SD* | *0,510* | *0,145* | *0,157* | *0,209* | *138,458* | *80,018* | *54,643* | *0,038* | *7,369* | *1,167* | *0,499* | *28,337* |
|  |  |  |  |  |  |  |  |  |  |  |  |  |  |
| 1% FO, LPS |  | 0,236 | 0,256 | 0,265 | 0,109 | 290,904 | 273,839 | 162,765 | 0,103 | 38,859 | 2,840 | 1,771 | 89,549 |
|  |  | 0,071 | 0,346 | 0,336 | 0,216 | 270,242 | 198,359 | 80,338 | 0,179 | 53,058 | 1,574 | 1,166 | 61,364 |
|  |  | 0,145 | 0,259 | 0,326 | 0,103 | 588,059 | 213,006 | 246,020 | 0,173 | 41,642 | 2,790 | 2,210 | 108,128 |
|  |  | 0,040 | 0,324 | 0,313 | 0,166 | 556,098 | 302,948 | 92,872 | 0,069 | 29,414 | 2,934 | 1,798 | 89,431 |
|  |  | 0,040 | 0,491 | 0,504 | 0,214 | 652,535 | 231,861 | 272,682 | 0,075 | 23,039 | 2,399 | 2,107 | 111,926 |
|  |  | 0,197 | 0,334 | 0,338 | 0,643 | 753,774 | 364,651 | 83,026 | 0,298 | 40,460 | 3,268 | 1,232 | 48,540 |
|  |  | 0,288 | 0,347 | 0,417 | 0,242 | 420,938 | 220,962 | 83,433 | 0,116 | 10,978 | 4,051 | 1,810 | 82,937 |
|  |  | 0,061 | 0,402 | 0,446 | 0,473 | 444,106 | 127,167 | 65,253 | 0,045 | 9,447 | 1,829 | 1,315 | 60,300 |
|  | *average* | *0,135* | *0,345* | *0,368* | *0,271* | *497,082* | *241,599* | *135,799* | *0,132* | *30,862* | *2,711* | *1,676* | *81,522* |
|  | *SD* | *0,096* | *0,076* | *0,080* | *0,189* | *170,874* | *71,960* | *81,972* | *0,082* | *15,502* | *0,788* | *0,397* | *23,009* |
|  |  |  |  |  |  |  |  |  |  |  |  |  |  |
| 3% FO, saline |  | 0,454 | 0,187 | 0,166 | 0,130 | 226,036 | 44,812 | 20,126 | 0,033 | 14,753 | 1,212 | 0,587 | 33,815 |
|  |  | 0,040 | 0,392 | 0,286 | 0,592 | 439,908 | 101,793 | 101,864 | 0,020 | 1,950 | 2,047 | 1,193 | 62,579 |
|  |  | 0,088 | 0,254 | 0,186 | 0,229 | 169,401 | 92,060 | 52,957 | 0,071 | 14,017 | 0,984 | 0,673 | 31,597 |
|  |  | 0,040 | 0,330 | 0,158 | 0,114 | 246,359 | 87,779 | 125,567 | 0,020 | 0,200 | 2,449 | 1,448 | 68,753 |
|  |  | 0,040 | 0,491 | 0,333 | 0,205 | 422,027 | 121,719 | 85,053 | 0,020 | 10,252 | 3,714 | 1,341 | 64,142 |
|  |  | 0,040 | 0,228 | 0,236 | 0,107 | 366,925 | 114,631 | 93,726 | 0,020 | 14,167 | 2,017 | 1,076 | 57,059 |
|  |  | 0,263 | 1,668 | 1,854 | 0,753 | 501,005 | 160,203 | 43,149 | 0,166 | 41,842 | 2,993 | 1,395 | 80,561 |
|  |  | 0,040 | 0,342 | 0,327 | 0,162 | 426,344 | 190,639 | 97,646 | 0,060 | 6,621 | 2,808 | 1,428 | 92,797 |
|  | *average* | *0,126* | *0,486* | *0,443* | *0,287* | *349,751* | *114,205* | *77,511* | *0,051* | *12,975* | *2,278* | *1,143* | *61,413* |
|  | *SD* | *0,153* | *0,487* | *0,574* | *0,246* | *120,067* | *44,997* | *35,263* | *0,051* | *12,937* | *0,911* | *0,341* | *20,989* |
|  |  |  |  |  |  |  |  |  |  |  |  |  |  |
| 3% FO, LPS |  | 0,233 | 0,683 | 0,505 | 0,496 | 425,240 | 351,546 | 58,225 | 0,020 | 5,176 | 2,240 | 0,948 | 54,566 |
|  |  | 0,040 | 0,327 | 0,421 | 0,337 | 293,642 | 237,560 | 10,495 | 0,020 | 8,024 | 4,427 | 0,704 | 32,382 |
|  |  | 0,040 | 0,293 | 0,330 | 0,175 | 553,628 | 304,105 | 56,754 | 0,125 | 7,865 | 3,907 | 1,105 | 59,700 |
|  |  | 0,119 | 0,320 | 0,363 | 0,119 | 412,956 | 594,824 | 133,700 | 0,151 | 57,629 | 3,034 | 1,595 | 88,404 |
|  |  | 0,040 | 0,302 | 0,348 | 0,572 | 390,434 | 409,633 | 20,076 | 0,020 | 6,064 | 3,982 | 1,064 | 50,547 |
|  |  | 0,786 | 0,571 | 0,568 | 0,353 | 1146,627 | 408,272 | 245,559 | 0,171 | 9,188 | 3,569 | 1,982 | 96,750 |
|  |  | 0,040 | 0,286 | 0,271 | 0,116 | 400,605 | 273,158 | 20,428 | 0,020 | 7,226 | 1,552 | 0,908 | 41,266 |
|  | *average* | *0,185* | *0,398* | *0,401* | *0,310* | *517,590* | *368,443* | *77,891* | *0,075* | *14,453* | *3,244* | *1,187* | *60,516* |
|  | *SD* | *0,274* | *0,161* | *0,104* | *0,182* | *287,654* | *119,140* | *84,900* | *0,070* | *19,084* | *1,033* | *0,445* | *23,763* |

| **Ileum** |  | 10(S)-17(S)-DiHDoHE | 19,20-DiHoPE | TBXB3 | TBXB2 | 8,9-DiHETrE | 13,14-dihydro-15-keto-PGF2a | lipoxin A4 | PGE2 | PGD2 | Leukotriene E4 | n-acetyl leukotriene E4 | Leukotriene D4 |
| --- | --- | --- | --- | --- | --- | --- | --- | --- | --- | --- | --- | --- | --- |
|  |  | ng/gram tissue weight | ng/gram tissue weight | ng/gram tissue weight | ng/gram tissue weight | ng/gram tissue weight | ng/gram tissue weight | ng/gram tissue weight | ng/gram tissue weight | ng/gram tissue weight | ng/gram tissue weight | ng/gram tissue weight | ng/gram tissue weight |
|  |  |  |  |  |  |  |  |  |  |  |  |  |  |
| ctrl, saline |  | 1,431 | 1,727 | 0,223 | 49,712 | 2,059 | 94,181 | 1,672 | 321,606 | 447,027 | 0,749 | 0,200 | 0,500 |
|  |  | 1,969 | 0,992 | 0,155 | 60,045 | 1,282 | 34,555 | 0,893 | 479,192 | 429,458 | 1,148 | 0,200 | 1,187 |
|  |  | 0,796 | 0,726 | 0,099 | 13,801 | 0,446 | 17,897 | 0,100 | 90,526 | 114,185 | 2,109 | 0,200 | 3,430 |
|  |  | 1,081 | 1,193 | 0,092 | 23,828 | 1,150 | 72,620 | 0,331 | 155,289 | 117,902 | 0,580 | 0,200 | 1,038 |
|  |  | 1,275 | 1,316 | 0,073 | 39,789 | 1,243 | 84,884 | 0,100 | 335,978 | 438,724 | 0,486 | 0,200 | 1,697 |
|  |  | 1,057 | 1,321 | 0,112 | 29,041 | 1,549 | 26,139 | 2,376 | 180,776 | 217,626 | 0,320 | 0,200 | 0,964 |
|  |  | 0,740 | 1,915 | 0,120 | 42,840 | 0,403 | 49,255 | 1,224 | 847,362 | 526,190 | 3,478 | 0,200 | 5,105 |
|  |  | 5,893 | 1,589 | 0,067 | 57,344 | 1,238 | 107,315 | 0,100 | 1162,854 | 2778,090 | 1,023 | 0,200 | 6,245 |
|  | *average* | *1,780* | *1,347* | *0,118* | *39,550* | *1,171* | *60,856* | *0,849* | *446,698* | *633,650* | *1,237* | *0,200* | *2,521* |
|  | *SD* | *1,707* | *0,390* | *0,051* | *16,351* | *0,544* | *33,513* | *0,854* | *375,094* | *881,039* | *1,063* | *0,000* | *2,156* |
|  |  |  |  |  |  |  |  |  |  |  |  |  |  |
| ctrl, LPS |  | 1,111 | 2,162 | 0,140 | 51,401 | 2,697 | 26,766 | 0,992 | 676,330 | 565,698 | 5,170 | 3,701 | 0,846 |
|  |  | 0,384 | 1,861 | 0,363 | 123,885 | 1,635 | 104,278 | 0,906 | 490,449 | 741,305 | 0,471 | 0,200 | 1,873 |
|  |  | 1,233 | 3,299 | 0,135 | 42,727 | 4,093 | 21,272 | 1,128 | 530,545 | 384,922 | 7,945 | 3,738 | 0,500 |
|  |  | 0,748 | 1,598 | 0,156 | 68,357 | 1,320 | 43,450 | 0,797 | 492,487 | 495,739 | 0,527 | 0,234 | 1,813 |
|  |  | 0,883 | 1,488 | 0,071 | 44,261 | 1,021 | 25,714 | 0,100 | 305,785 | 335,894 | 0,654 | 0,660 | 0,500 |
|  |  | 1,647 | 1,103 | 0,318 | 161,357 | 0,811 | 49,941 | 2,171 | 1197,892 | 989,431 | 3,208 | 0,857 | 4,091 |
|  |  | 2,719 | 2,655 | 0,693 | 162,309 | 1,238 | 101,009 | 2,772 | 1367,088 | 885,967 | 30,118 | 19,580 | 10,997 |
|  | *average* | *1,247* | *2,024* | *0,268* | *93,471* | *1,831* | *53,204* | *1,266* | *722,939* | *628,422* | *6,870* | *4,138* | *2,946* |
|  | *SD* | *0,761* | *0,751* | *0,215* | *54,267* | *1,170* | *35,294* | *0,903* | *400,182* | *250,268* | *10,628* | *6,981* | *3,763* |
|  |  |  |  |  |  |  |  |  |  |  |  |  |  |
| 1% FO, saline |  | 5,635 | 3,777 | 0,443 | 20,634 | 0,705 | 8,874 | 0,258 | 134,844 | 158,376 | 0,500 | 0,200 | 0,500 |
|  |  | 1,197 | 3,627 | 0,103 | 9,320 | 0,742 | 5,740 | 1,726 | 46,842 | 137,377 | 0,500 | 0,200 | 0,500 |
|  |  | 3,642 | 3,064 | 0,479 | 18,519 | 0,417 | 20,652 | 1,533 | 133,339 | 206,537 | 0,500 | 0,200 | 0,500 |
|  |  | 0,885 | 3,508 | 0,283 | 10,655 | 0,391 | 6,531 | 0,916 | 106,512 | 247,972 | 0,500 | 0,200 | 0,500 |
|  |  | 3,864 | 3,781 | 0,313 | 16,433 | 0,475 | 13,669 | 1,307 | 168,140 | 477,132 | 0,500 | 0,200 | 0,500 |
|  |  | 2,188 | 4,098 | 0,330 | 17,746 | 0,704 | 9,189 | 0,743 | 123,311 | 278,470 | 0,500 | 0,200 | 0,500 |
|  |  | 3,784 | 3,713 | 8,643 | 76,350 | 1,070 | 23,391 | 1,607 | 523,310 | 560,986 | 0,500 | 0,200 | 0,500 |
|  |  | 4,061 | 3,942 | 2,789 | 30,396 | 0,974 | 8,146 | 3,539 | 317,117 | 383,599 | 0,500 | 0,200 | 0,500 |
|  | *average* | *3,157* | *3,689* | *1,673* | *25,007* | *0,685* | *12,024* | *1,454* | *194,177* | *306,306* | *0,500* | *0,200* | *0,500* |
|  | *SD* | *1,605* | *0,311* | *2,948* | *21,728* | *0,250* | *6,644* | *0,978* | *153,882* | *153,329* | *0,000* | *0,000* | *0,000* |
|  |  |  |  |  |  |  |  |  |  |  |  |  |  |
| 1% FO, LPS |  | 2,900 | 3,334 | 8,496 | 97,183 | 0,342 | 26,262 | 0,466 | 535,276 | 724,238 | 0,500 | 0,200 | 0,500 |
|  |  | 1,563 | 4,368 | 5,652 | 77,270 | 0,240 | 23,449 | 0,279 | 459,302 | 416,489 | 0,500 | 0,200 | 0,500 |
|  |  | 9,197 | 3,917 | 2,508 | 62,520 | 0,142 | 14,984 | 0,100 | 601,118 | 1082,470 | 0,500 | 0,200 | 0,500 |
|  |  | 5,107 | 4,030 | 10,775 | 106,508 | 0,906 | 14,793 | 0,736 | 895,341 | 746,928 | 0,500 | 0,200 | 0,500 |
|  |  | 6,358 | 3,736 | 2,204 | 49,807 | 0,576 | 7,899 | 0,481 | 567,917 | 1038,164 | 0,500 | 0,200 | 0,500 |
|  |  | 6,077 | 7,138 | 8,055 | 78,852 | 0,382 | 29,175 | 3,703 | 743,664 | 379,784 | 0,500 | 3,524 | 0,500 |
|  |  | 3,521 | 4,153 | 3,225 | 64,034 | 0,515 | 15,376 | 0,989 | 468,621 | 458,001 | 0,500 | 1,072 | 0,500 |
|  |  | 2,895 | 3,174 | 7,769 | 79,252 | 0,371 | 5,155 | 0,040 | 483,858 | 446,208 | 0,500 | 0,200 | 0,500 |
|  | *average* | *4,702* | *4,231* | *6,086* | *76,928* | *0,434* | *17,137* | *0,849* | *594,387* | *661,535* | *0,500* | *0,724* | *0,500* |
|  | *SD* | *2,472* | *1,241* | *3,179* | *18,531* | *0,235* | *8,537* | *1,195* | *152,774* | *282,206* | *0,000* | *1,171* | *0,000* |
|  |  |  |  |  |  |  |  |  |  |  |  |  |  |
| 3% FO, saline |  | 1,997 | 1,378 | 1,300 | 11,975 | 0,106 | 12,167 | 1,500 | 91,397 | 134,639 | 0,500 | 0,200 | 0,500 |
|  |  | 7,904 | 5,047 | 1,245 | 16,258 | 0,402 | 2,641 | 1,700 | 196,602 | 308,730 | 0,500 | 0,200 | 0,500 |
|  |  | 0,961 | 1,653 | 5,274 | 26,125 | 0,204 | 10,951 | 2,082 | 143,228 | 167,137 | 0,500 | 0,200 | 0,500 |
|  |  | 2,802 | 3,658 | 0,780 | 6,566 | 0,030 | 1,941 | 7,526 | 177,068 | 447,959 | 0,500 | 0,200 | 0,500 |
|  |  | 4,670 | 4,067 | 0,979 | 8,742 | 0,030 | 1,994 | 5,417 | 180,640 | 343,152 | 0,500 | 0,200 | 0,500 |
|  |  | 3,344 | 3,187 | 2,374 | 16,402 | 0,184 | 1,358 | 2,049 | 167,062 | 335,005 | 0,500 | 0,200 | 0,500 |
|  |  | 7,981 | 7,397 | 3,289 | 31,319 | 1,602 | 37,566 | 3,654 | 285,133 | 139,112 | 0,500 | 0,200 | 0,500 |
|  |  | 7,966 | 4,596 | 2,008 | 19,393 | 0,239 | 7,980 | 2,343 | 307,505 | 213,077 | 0,500 | 0,200 | 0,500 |
|  | *average* | *4,703* | *3,873* | *2,156* | *17,098* | *0,349* | *9,575* | *3,284* | *193,579* | *261,101* | *0,500* | *0,200* | *0,500* |
|  | *SD* | *2,889* | *1,928* | *1,508* | *8,428* | *0,520* | *12,107* | *2,146* | *71,204* | *114,261* | *0,000* | *0,000* | *0,000* |
|  |  |  |  |  |  |  |  |  |  |  |  |  |  |
| 3% FO, LPS |  | 6,205 | 17,772 | 0,651 | 32,580 | 0,750 | 10,513 | 2,765 | 225,352 | 78,545 | 0,500 | 26,961 | 0,500 |
|  |  | 3,903 | 8,341 | 1,124 | 13,622 | 0,461 | 10,979 | 4,393 | 165,111 | 102,102 | 0,500 | 18,604 | 0,500 |
|  |  | 5,562 | 11,005 | 4,723 | 35,892 | 0,299 | 6,018 | 3,828 | 307,093 | 298,523 | 0,500 | 8,302 | 0,500 |
|  |  | 3,853 | 12,389 | 9,189 | 48,714 | 0,546 | 17,756 | 0,609 | 615,863 | 506,907 | 10,165 | 5,741 | 0,500 |
|  |  | 3,988 | 8,164 | 0,870 | 14,110 | 0,526 | 15,590 | 10,651 | 325,833 | 131,909 | 47,712 | 17,307 | 0,500 |
|  |  | 13,133 | 6,285 | 10,473 | 107,980 | 0,718 | 22,628 | 0,945 | 681,325 | 547,595 | 0,500 | 0,200 | 0,500 |
|  |  | 3,444 | 9,133 | 1,347 | 17,631 | 0,265 | 11,445 | 3,255 | 233,459 | 152,503 | 0,500 | 9,317 | 0,500 |
|  | *average* | *5,727* | *10,441* | *4,054* | *38,647* | *0,509* | *13,561* | *3,778* | *364,862* | *259,726* | *8,625* | *12,347* | *0,500* |
|  | *SD* | *3,420* | *3,796* | *4,196* | *33,223* | *0,187* | *5,496* | *3,341* | *201,925* | *196,205* | *17,608* | *9,074* | *0,000* |

| **Ileum** |  | 17 keto- 4(z), 7(z), 10(z), 13 (z), 15 (E), 19(z)-DHA | 12,13-DiHOME | 9,10-DiHOME | 9,12,13-TriHOME | 9,10,13-TriHOME | UK1 | UK2 | UK3 | UK4 | UK5 |
| --- | --- | --- | --- | --- | --- | --- | --- | --- | --- | --- | --- |
|  |  | ng/gram tissue weight | ng/gram tissue weight | ng/gram tissue weight | RR | RR | RR | RR | RR | RR | RR |
|  |  |  |  |  |  |  |  |  |  |  |  |
| ctrl, saline |  | 89,787 | 491,591 | 858,780 | 8332,511 | 18557,885 | 2,465 | 0,089 | 0,093 | 0,037 | 0,067 |
|  |  | 165,404 | 461,224 | 737,677 | 10064,637 | 14224,795 | 2,311 | 0,165 | 0,146 | 0,097 | 0,132 |
|  |  | 123,881 | 259,006 | 395,346 | 8169,067 | 9616,958 | 1,738 | 0,066 | 0,092 | 0,061 | 0,105 |
|  |  | 280,660 | 432,605 | 835,476 | 23723,145 | 15426,222 | 2,452 | 0,167 | 0,233 | 0,096 | 0,332 |
|  |  | 148,158 | 431,806 | 698,978 | 14606,056 | 11131,252 | 1,828 | 0,086 | 0,067 | 0,035 | 0,085 |
|  |  | 188,090 | 471,266 | 906,990 | 38901,648 | 25724,944 | 5,599 | 0,206 | 0,198 | 0,072 | 0,144 |
|  |  | 9,923 | 440,742 | 664,919 | 18046,050 | 13112,571 | 5,706 | 0,127 | 0,093 | 0,039 | 0,186 |
|  |  | 217,875 | 769,435 | 1284,795 | 37427,354 | 32168,524 | 10,382 | 0,566 | 0,178 | 0,089 | 0,300 |
|  | *average* | *152,972* | *469,709* | *797,870* | *19908,809* | *17495,394* | *4,060* | *0,184* | *0,137* | *0,066* | *0,169* |
|  | *SD* | *82,140* | *140,630* | *252,765* | *12435,580* | *7754,396* | *3,014* | *0,162* | *0,060* | *0,027* | *0,098* |
|  |  |  |  |  |  |  |  |  |  |  |  |
| ctrl, LPS |  | 105,459 | 366,197 | 823,504 | 8402,206 | 7213,625 | 2,581 | 0,165 | 0,115 | 0,057 | 0,149 |
|  |  | 68,879 | 444,500 | 756,612 | 13424,912 | 11107,321 | 2,604 | 0,092 | 0,117 | 0,039 | 0,109 |
|  |  | 80,980 | 763,493 | 1495,055 | 16415,374 | 14834,717 | 3,499 | 0,181 | 0,061 | 0,038 | 0,251 |
|  |  | 82,429 | 400,139 | 655,218 | 14144,562 | 11023,118 | 2,785 | 0,301 | 0,208 | 0,105 | 0,194 |
|  |  | 41,160 | 373,359 | 583,875 | 14068,206 | 10809,445 | 2,163 | 0,101 | 0,100 | 0,046 | 0,116 |
|  |  | 50,514 | 441,218 | 895,959 | 27187,933 | 20462,295 | 5,628 | 0,151 | 0,137 | 0,019 | 0,185 |
|  |  | 111,305 | 424,565 | 1017,618 | 16714,238 | 32895,891 | 7,675 | 0,220 | 0,127 | 0,084 | 0,173 |
|  | *average* | *77,247* | *459,067* | *889,692* | *15765,347* | *15478,059* | *3,848* | *0,173* | *0,124* | *0,055* | *0,168* |
|  | *SD* | *26,088* | *137,744* | *303,716* | *5730,025* | *8733,881* | *2,043* | *0,072* | *0,045* | *0,030* | *0,049* |
|  |  |  |  |  |  |  |  |  |  |  |  |
| 1% FO, saline |  | 215,104 | 539,267 | 944,333 | 13229,756 | 11169,990 | 7,157 | 0,276 | 0,181 | 0,068 | 0,381 |
|  |  | 101,496 | 562,040 | 900,778 | 28752,275 | 23315,510 | 4,777 | 0,288 | 0,193 | 0,078 | 0,221 |
|  |  | 146,149 | 363,922 | 694,008 | 12747,086 | 11431,939 | 4,993 | 0,203 | 0,199 | 0,040 | 0,263 |
|  |  | 106,516 | 431,435 | 744,593 | 14257,960 | 11917,954 | 6,653 | 0,350 | 0,133 | 0,093 | 0,331 |
|  |  | 149,678 | 515,621 | 925,435 | 21599,236 | 17105,633 | 9,570 | 0,401 | 0,401 | 0,076 | 0,443 |
|  |  | 297,087 | 712,644 | 1162,159 | 11355,954 | 8528,735 | 4,322 | 0,378 | 0,266 | 0,077 | 0,327 |
|  |  | 48,278 | 605,378 | 1344,528 | 22729,378 | 16250,118 | 5,815 | 0,247 | 0,126 | 0,066 | 0,129 |
|  |  | 121,336 | 792,064 | 1597,732 | 49180,970 | 43018,552 | 8,067 | 0,176 | 0,069 | 0,097 | 0,107 |
|  | *average* | *148,206* | *565,296* | *1039,196* | *21731,577* | *17842,304* | *6,419* | *0,290* | *0,196* | *0,074* | *0,275* |
|  | *SD* | *76,788* | *139,575* | *308,766* | *12650,709* | *11176,060* | *1,802* | *0,081* | *0,102* | *0,017* | *0,118* |
|  |  |  |  |  |  |  |  |  |  |  |  |
| 1% FO, LPS |  | 173,704 | 325,009 | 623,115 | 8783,638 | 7244,938 | 4,673 | 0,177 | 0,166 | 0,022 | 0,097 |
|  |  | 96,553 | 330,042 | 587,607 | 8064,056 | 6367,425 | 1,984 | 0,109 | 0,040 | 0,029 | 0,112 |
|  |  | 185,137 | 360,240 | 678,496 | 11525,133 | 7340,248 | 6,710 | 0,289 | 0,118 | 0,085 | 0,226 |
|  |  | 216,910 | 292,754 | 623,963 | 10099,005 | 8966,378 | 5,314 | 0,207 | 0,197 | 0,040 | 0,186 |
|  |  | 178,332 | 578,249 | 1034,551 | 18129,110 | 11659,773 | 6,594 | 0,128 | 0,183 | 0,043 | 0,243 |
|  |  | 722,783 | 534,771 | 1062,169 | 11571,671 | 9475,249 | 5,805 | 0,260 | 0,221 | 0,038 | 0,233 |
|  |  | 105,414 | 485,292 | 911,197 | 15707,210 | 16844,769 | 5,332 | 0,171 | 0,090 | 0,105 | 0,126 |
|  |  | 117,907 | 423,861 | 821,302 | 14296,250 | 11436,799 | 4,654 | 0,234 | 0,176 | 0,058 | 0,133 |
|  | *average* | *224,593* | *416,277* | *792,800* | *12272,009* | *9916,947* | *5,133* | *0,197* | *0,149* | *0,052* | *0,169* |
|  | *SD* | *205,815* | *106,410* | *192,094* | *3502,033* | *3397,876* | *1,488* | *0,063* | *0,061* | *0,029* | *0,060* |
|  |  |  |  |  |  |  |  |  |  |  |  |
| 3% FO, saline |  | 315,519 | 308,429 | 579,851 | 10376,209 | 11191,258 | 3,015 | 0,248 | 0,087 | 0,078 | 0,184 |
|  |  | 172,030 | 341,433 | 683,567 | 12949,592 | 11511,957 | 2,568 | 0,161 | 0,088 | 0,047 | 0,040 |
|  |  | 198,499 | 252,275 | 523,423 | 15739,984 | 14942,366 | 1,286 | 0,052 | 0,020 | 0,040 | 0,076 |
|  |  | 391,283 | 334,021 | 812,269 | 59652,257 | 64884,124 | 6,536 | 0,152 | 0,054 | 0,022 | 0,076 |
|  |  | 236,251 | 506,459 | 1023,036 | 22736,564 | 22520,466 | 3,255 | 0,065 | 0,029 | 0,029 | 0,043 |
|  |  | 184,446 | 310,671 | 654,654 | 13872,072 | 12444,872 | 1,895 | 0,083 | 0,066 | 0,028 | 0,106 |
|  |  | 786,799 | 1285,476 | 2435,793 | 21817,316 | 20389,661 | 8,944 | 0,764 | 0,326 | 0,181 | 0,380 |
|  |  | 216,394 | 580,420 | 1137,732 | 24270,684 | 22973,907 | 4,400 | 0,229 | 0,198 | 0,070 | 0,175 |
|  | *average* | *312,653* | *489,898* | *981,291* | *22676,835* | *22607,326* | *3,987* | *0,219* | *0,108* | *0,062* | *0,135* |
|  | *SD* | *205,409* | *340,015* | *625,242* | *15775,356* | *17759,699* | *2,575* | *0,232* | *0,104* | *0,052* | *0,113* |
|  |  |  |  |  |  |  |  |  |  |  |  |
| 3% FO, LPS |  | 770,796 | 744,932 | 1907,713 | 10213,821 | 16292,463 | 3,729 | 0,237 | 0,084 | 0,059 | 0,145 |
|  |  | 451,037 | 311,497 | 740,829 | 2683,314 | 5766,427 | 1,602 | 0,098 | 0,076 | 0,033 | 0,129 |
|  |  | 351,683 | 439,437 | 769,082 | 9517,665 | 18716,868 | 2,491 | 0,215 | 0,144 | 0,061 | 0,132 |
|  |  | 174,353 | 626,695 | 807,073 | 6754,881 | 10269,106 | 2,381 | 0,060 | 0,049 | 0,025 | 0,090 |
|  |  | 155,989 | 474,817 | 818,957 | 7214,411 | 16273,110 | 4,223 | 0,213 | 0,146 | 0,079 | 0,172 |
|  |  | 407,543 | 612,722 | 1084,052 | 21445,152 | 16599,105 | 6,523 | 0,196 | 0,160 | 0,029 | 0,174 |
|  |  | 180,415 | 431,381 | 667,298 | 4727,949 | 8337,710 | 2,568 | 0,117 | 0,137 | 0,037 | 0,135 |
|  | *average* | *355,974* | *520,211* | *970,715* | *8936,742* | *13179,256* | *3,360* | *0,162* | *0,114* | *0,046* | *0,140* |
|  | *SD* | *218,934* | *147,442* | *433,241* | *6096,105* | *4974,670* | *1,649* | *0,069* | *0,043* | *0,020* | *0,029* |

| **Ileum** |  | epea | dhea | aea | 2-ag | dle | pea | oea | sea |
| --- | --- | --- | --- | --- | --- | --- | --- | --- | --- |
|  |  | ng/gram tissue weight | ng/gram tissue weight | ng/gram tissue weight | ng/gram tissue weight | ng/gram tissue weight | ng/gram tissue weight | ng/gram tissue weight | ng/gram tissue weight |
|  |  |  |  |  |  |  |  |  |  |
| ctrl, saline |  | 0,100 | 3,191 | 9,888 | 15660,420 | 0,775 | 72,588 | 94,634 | 121,659 |
|  |  | 0,100 | 3,587 | 5,657 | 39900,508 | 1,319 | 190,693 | 362,116 | 105,095 |
|  |  | 0,100 | 7,607 | 6,760 | 63609,357 | 2,000 | 155,701 | 190,680 | 80,699 |
|  |  | 0,100 | 5,155 | 5,176 | 56535,391 | 1,364 | 95,350 | 125,872 | 84,249 |
|  |  | 0,100 | 5,289 | 8,850 | 61923,862 | 1,717 | 90,432 | 102,003 | 68,106 |
|  |  | 0,100 | 10,083 | 5,489 | 71734,095 | 0,726 | 92,660 | 140,392 | 53,864 |
|  |  | 7,150 | 3,906 | 1,847 | 36109,251 | 0,669 | 59,370 | 75,705 | 73,661 |
|  |  | 8,524 | 13,182 | 16,746 | 77302,834 | 4,223 | 65,318 | 99,072 | 34,374 |
|  | *average* | *2,034* | *6,500* | *7,552* | *52846,965* | *1,599* | *102,764* | *148,809* | *77,713* |
|  | *SD* | *3,600* | *3,549* | *4,446* | *20692,434* | *1,165* | *46,351* | *93,192* | *27,486* |
|  |  |  |  |  |  |  |  |  |  |
| ctrl, LPS |  | 0,100 | 12,475 | 14,912 | 24218,676 | 2,268 | 109,556 | 184,891 | 113,720 |
|  |  | 0,100 | 10,339 | 24,705 | 37542,047 | 3,544 | 128,947 | 154,175 | 143,352 |
|  |  | 0,100 | 26,189 | 33,267 | 69087,329 | 3,180 | 110,697 | 180,475 | 73,978 |
|  |  | 0,100 | 12,009 | 15,939 | 77768,690 | 2,663 | 134,416 | 151,130 | 151,110 |
|  |  | 0,100 | 14,472 | 28,348 | 67948,942 | 3,139 | 119,784 | 120,014 | 123,916 |
|  |  | 0,100 | 6,108 | 14,285 | 26016,223 | 1,806 | 71,031 | 122,730 | 70,105 |
|  |  | 0,100 | 10,526 | 12,234 | 35284,014 | 1,700 | 106,473 | 126,953 | 80,544 |
|  | *average* | *0,100* | *13,160* | *20,527* | *48266,560* | *2,614* | *111,558* | *148,624* | *108,103* |
|  | *SD* | *0,000* | *6,297* | *8,178* | *22539,654* | *0,716* | *20,678* | *26,850* | *33,520* |
|  |  |  |  |  |  |  |  |  |  |
| 1% FO, saline |  | 53,785 | 35,077 | 9,861 | 23792,339 | 2,528 | 98,839 | 68,069 | 101,400 |
|  |  | 15,358 | 11,424 | 1,751 | 11042,525 | 0,526 | 69,894 | 68,611 | 61,592 |
|  |  | 15,690 | 12,871 | 3,656 | 15843,157 | 0,919 | 78,010 | 80,994 | 42,896 |
|  |  | 12,089 | 15,743 | 3,038 | 16191,378 | 0,485 | 76,224 | 87,459 | 120,175 |
|  |  | 14,935 | 11,393 | 2,409 | 11858,821 | 0,785 | 60,733 | 72,221 | 43,097 |
|  |  | 15,848 | 13,959 | 2,637 | 15732,367 | 0,452 | 47,778 | 68,011 | 44,497 |
|  |  | 44,251 | 10,572 | 5,500 | 14963,857 | 0,974 | 72,821 | 142,827 | 31,182 |
|  |  | 10,237 | 13,761 | 1,679 | 10132,964 | 0,247 | 49,621 | 59,238 | 34,624 |
|  | *average* | *22,774* | *15,600* | *3,816* | *14944,676* | *0,865* | *69,240* | *80,929* | *59,933* |
|  | *SD* | *16,514* | *8,047* | *2,731* | *4295,602* | *0,717* | *16,617* | *26,469* | *33,016* |
|  |  |  |  |  |  |  |  |  |  |
| 1% FO, LPS |  | 20,351 | 11,137 | 4,518 | 13150,266 | 1,141 | 74,934 | 117,455 | 53,510 |
|  |  | 20,683 | 20,226 | 5,694 | 15822,704 | 1,319 | 93,090 | 111,301 | 82,559 |
|  |  | 16,335 | 19,837 | 6,416 | 13522,074 | 1,073 | 102,059 | 130,600 | 89,232 |
|  |  | 14,602 | 17,854 | 5,464 | 13962,149 | 0,940 | 93,097 | 105,765 | 73,935 |
|  |  | 30,103 | 19,904 | 4,551 | 24912,625 | 1,028 | 110,875 | 118,326 | 84,217 |
|  |  | 82,530 | 52,582 | 14,801 | 17420,893 | 5,735 | 252,120 | 200,108 | 182,812 |
|  |  | 37,696 | 36,584 | 8,895 | 22363,810 | 2,295 | 126,025 | 135,612 | 92,101 |
|  |  | 8,022 | 32,716 | 3,340 | 9024,198 | 1,402 | 119,998 | 136,705 | 83,566 |
|  | *average* | *28,790* | *26,355* | *6,710* | *16272,340* | *1,867* | *121,525* | *131,984* | *92,742* |
|  | *SD* | *23,585* | *13,408* | *3,659* | *5188,803* | *1,620* | *55,236* | *29,740* | *38,320* |
|  |  |  |  |  |  |  |  |  |  |
| 3% FO, saline |  | 29,382 | 20,913 | 2,411 | 18686,334 | 0,499 | 89,152 | 92,523 | 44,729 |
|  |  | 24,692 | 23,135 | 1,671 | 11634,204 | 0,173 | 56,140 | 51,938 | 29,627 |
|  |  | 30,016 | 17,015 | 6,502 | 20054,452 | 1,730 | 73,445 | 41,582 | 71,556 |
|  |  | 179,684 | 40,324 | 14,880 | 13207,333 | 1,716 | 81,565 | 76,847 | 30,669 |
|  |  | 54,704 | 22,468 | 3,970 | 12397,811 | 0,597 | 81,631 | 91,888 | 35,681 |
|  |  | 17,707 | 13,220 | 1,052 | 6810,214 | 0,164 | 47,305 | 31,587 | 34,013 |
|  |  | 239,806 | 67,367 | 89,348 | 17059,338 | 24,906 | 167,533 | 209,155 | 76,037 |
|  |  | 74,639 | 17,388 | 3,363 | 10838,881 | 0,649 | 108,279 | 111,887 | 41,554 |
|  | *average* | *81,329* | *27,729* | *15,400* | *13836,071* | *3,804* | *88,131* | *88,426* | *45,483* |
|  | *SD* | *82,919* | *17,957* | *30,205* | *4443,948* | *8,548* | *37,231* | *56,119* | *18,236* |
|  |  |  |  |  |  |  |  |  |  |
| 3% FO, LPS |  | 43,794 | 25,799 | 4,344 | 16522,385 | 0,785 | 116,747 | 120,479 | 82,584 |
|  |  | 113,455 | 36,745 | 10,857 | 15969,997 | 1,753 | 145,325 | 133,036 | 116,552 |
|  |  | 61,695 | 25,796 | 8,150 | 14198,412 | 1,651 | 160,512 | 138,632 | 137,232 |
|  |  | 72,076 | 26,731 | 6,160 | 10822,756 | 1,774 | 144,407 | 134,067 | 111,843 |
|  |  | 45,948 | 22,341 | 5,391 | 12390,919 | 1,100 | 118,544 | 119,617 | 95,515 |
|  |  | 49,227 | 27,188 | 8,022 | 13788,429 | 1,244 | 122,520 | 99,926 | 99,441 |
|  |  | 36,790 | 24,635 | 4,922 | 15496,915 | 0,431 | 121,872 | 105,323 | 98,979 |
|  | *average* | *60,427* | *27,033* | *6,835* | *14169,973* | *1,248* | *132,847* | *121,583* | *106,021* |
|  | *SD* | *26,203* | *4,569* | *2,299* | *2043,281* | *0,516* | *17,057* | *14,811* | *17,661* |

| **Adi. tiss.** |  | 12(S)-HHTrE | 9(S)-HODE | 13(S)-HODE | EPA | AA | 15-deoxy-d-12,14-PGJ2 | 12(S)-HEPE | 5(S)-HEPE |
| --- | --- | --- | --- | --- | --- | --- | --- | --- | --- |
|  |  | ng/gram tissue weight | ng/gram tissue weight | ng/gram tissue weight | ng/gram tissue weight | ng/gram tissue weight | ng/gram tissue weight | ng/gram tissue weight | ng/gram tissue weight |
|  |  |  |  |  |  |  |  |  |  |
| ctrl, saline |  | 0,574 | 46,520 | 59,229 | 65,427 | 6847,355 | 0,100 | 0,367 | 0,100 |
|  |  | 0,545 | 41,627 | 63,061 | 74,937 | 9283,457 | 0,100 | 0,320 | 0,100 |
|  |  | 0,396 | 43,653 | 60,017 | 89,635 | 7697,893 | 0,100 | 0,364 | 0,100 |
|  |  | 0,400 | 36,333 | 57,109 | 92,069 | 8319,496 | 0,100 | 0,648 | 0,100 |
|  |  | 1,158 | 65,722 | 138,919 | 90,577 | 8969,521 | 0,100 | 0,733 | 0,100 |
|  |  | 2,373 | 55,006 | 132,305 | 114,071 | 12058,560 | 0,100 | 1,496 | 0,100 |
|  |  | 1,059 | 90,813 | 164,003 | 333,037 | 12096,841 | 0,100 | 4,107 | 0,100 |
|  |  | 12,377 | 384,218 | 950,363 | 304,446 | 20548,826 | 0,100 | 10,159 | 0,334 |
|  | *average* | *2,360* | *95,486* | *203,126* | *145,525* | *10727,744* | *0,100* | *2,274* | *0,129* |
|  | *SD* | *4,100* | *117,957* | *304,996* | *108,104* | *4396,647* | *0,000* | *3,428* | *0,083* |
|  |  |  |  |  |  |  |  |  |  |
| ctrl, LPS |  | 1,218 | 58,134 | 72,507 | 126,002 | 12616,372 | 0,100 | 0,556 | 0,100 |
|  |  | 5,393 | 144,381 | 110,591 | 146,129 | 15753,191 | 0,100 | 1,477 | 0,100 |
|  |  | 1,336 | 77,922 | 77,628 | 136,841 | 12284,117 | 0,100 | 0,343 | 0,370 |
|  |  | 3,937 | 165,974 | 150,331 | 103,409 | 11525,893 | 0,100 | 0,407 | 0,100 |
|  |  | 1,905 | 74,375 | 112,995 | 150,270 | 12213,669 | 0,100 | 0,878 | 0,100 |
|  |  | 2,765 | 124,331 | 160,432 | 104,139 | 11312,589 | 0,100 | 0,901 | 0,100 |
|  |  | 13,586 | 185,663 | 213,387 | 206,367 | 19757,969 | 0,100 | 4,393 | 0,100 |
|  |  | 5,403 | 193,553 | 298,036 | 232,775 | 16832,689 | 0,100 | 5,277 | 0,167 |
|  | *average* | *4,443* | *128,041* | *149,488* | *150,741* | *14037,061* | *0,100* | *1,779* | *0,142* |
|  | *SD* | *4,053* | *52,930* | *75,786* | *46,387* | *3062,074* | *0,000* | *1,934* | *0,095* |
|  |  |  |  |  |  |  |  |  |  |
| 1% FO, saline |  | 0,136 | 36,951 | 31,058 | 1773,222 | 2522,137 | 0,100 | 6,191 | 0,910 |
|  |  | 0,143 | 16,476 | 26,106 | 2185,922 | 3519,246 | 0,100 | 7,944 | 1,585 |
|  |  | 0,072 | 14,798 | 17,924 | 1273,379 | 2170,150 | 0,100 | 6,000 | 0,464 |
|  |  | 0,282 | 32,132 | 46,019 | 3096,291 | 4927,824 | 0,100 | 20,419 | 2,333 |
|  |  | 0,425 | 14,337 | 22,071 | 1638,646 | 2723,616 | 0,100 | 6,601 | 1,039 |
|  |  | 0,284 | 30,494 | 52,600 | 4089,186 | 5510,282 | 0,100 | 23,143 | 2,594 |
|  |  | 0,393 | 30,062 | 32,604 | 4602,543 | 6308,712 | 0,100 | 43,304 | 3,550 |
|  |  | 1,260 | 61,662 | 88,356 | 5890,397 | 5976,042 | 0,100 | 89,071 | 4,230 |
|  | *average* | *0,374* | *29,614* | *39,592* | *3068,698* | *4207,251* | *0,100* | *25,334* | *2,088* |
|  | *SD* | *0,379* | *15,647* | *22,892* | *1651,086* | *1665,867* | *0,000* | *28,804* | *1,332* |
|  |  |  |  |  |  |  |  |  |  |
| 1% FO, LPS |  | 1,968 | 131,054 | 109,483 | 7324,491 | 9606,631 | 0,100 | 69,509 | 12,918 |
|  |  | 1,127 | 83,352 | 125,957 | 5214,781 | 7528,181 | 0,100 | 57,509 | 6,298 |
|  |  | 1,748 | 116,118 | 115,924 | 5475,837 | 7587,950 | 0,100 | 80,853 | 7,964 |
|  |  | 0,915 | 93,822 | 129,843 | 5231,522 | 8180,493 | 0,100 | 67,216 | 3,938 |
|  |  | 1,486 | 77,624 | 133,563 | 4315,948 | 6600,410 | 0,100 | 41,063 | 7,230 |
|  |  | 0,574 | 69,460 | 62,795 | 8267,294 | 11042,982 | 0,100 | 106,306 | 6,916 |
|  |  | 3,546 | 109,914 | 170,804 | 4943,895 | 9233,020 | 0,100 | 98,598 | 6,395 |
|  |  | 1,867 | 135,389 | 119,276 | 4418,006 | 9569,212 | 0,100 | 78,364 | 5,711 |
|  | *average* | *1,654* | *102,092* | *120,956* | *5648,972* | *8668,610* | *0,100* | *74,927* | *7,171* |
|  | *SD* | *0,906* | *24,752* | *29,977* | *1406,368* | *1445,747* | *0,000* | *21,149* | *2,610* |
|  |  |  |  |  |  |  |  |  |  |
| 3% FO, saline |  | 0,088 | 15,146 | 20,186 | 1757,526 | 2902,442 | 0,100 | 29,754 | 2,670 |
|  |  | 0,530 | 35,696 | 51,298 | 5792,234 | 4393,463 | 0,100 | 92,628 | 5,247 |
|  |  | 0,263 | 17,550 | 25,454 | 4137,617 | 5065,252 | 0,100 | 44,289 | 3,992 |
|  |  | 0,731 | 19,872 | 27,717 | 4535,793 | 3615,272 | 0,100 | 50,979 | 2,307 |
|  |  | 0,864 | 34,797 | 51,024 | 5965,790 | 6923,847 | 0,100 | 143,017 | 7,485 |
|  |  | 0,357 | 18,855 | 32,746 | 7990,473 | 3834,914 | 0,100 | 86,885 | 4,072 |
|  |  | 0,601 | 33,072 | 51,307 | 10266,648 | 10826,120 | 0,100 | 179,375 | 17,772 |
|  |  | 0,277 | 22,776 | 61,802 | 7251,112 | 5156,441 | 0,100 | 145,814 | 6,728 |
|  | *average* | *0,464* | *24,720* | *40,192* | *5962,149* | *5339,719* | *0,100* | *96,592* | *6,284* |
|  | *SD* | *0,263* | *8,422* | *15,398* | *2601,521* | *2529,620* | *0,000* | *54,543* | *4,982* |
|  |  |  |  |  |  |  |  |  |  |
| 3% FO, LPS |  | 1,299 | 69,293 | 63,236 | 9345,528 | 14886,276 | 0,100 | 116,427 | 58,847 |
|  |  | 1,271 | 63,006 | 76,492 | 7458,618 | 13553,505 | 0,100 | 185,871 | 34,583 |
|  |  | 1,187 | 64,048 | 55,149 | 9423,467 | 13924,788 | 0,100 | 89,822 | 69,949 |
|  |  | 1,426 | 97,256 | 68,483 | 14331,150 | 19792,152 | 0,100 | 165,516 | 45,089 |
|  |  | 0,653 | 58,475 | 56,516 | 10883,638 | 12389,138 | 0,100 | 121,062 | 25,642 |
|  |  | 1,296 | 86,864 | 95,730 | 11587,476 | 14801,227 | 0,100 | 94,395 | 26,738 |
|  |  | 0,601 | 61,846 | 78,649 | 11420,298 | 14822,798 | 0,100 | 139,736 | 22,774 |
|  | *average* | *1,105* | *71,541* | *70,608* | *10635,739* | *14881,412* | *0,100* | *130,404* | *40,518* |
|  | *SD* | *0,334* | *14,690* | *14,299* | *2178,195* | *2345,746* | *0,000* | *35,643* | *18,186* |

| **Adi. tiss.** |  | 20(S)-HETE | 15(S)-HETE | 11(S)-HETE | 12(S)-HETE | 11,12 EET | 8,9 EET | 5,6 EET | 5(S)-HETE | 14,15 EET | DHA | PGB2 | LTB4 |
| --- | --- | --- | --- | --- | --- | --- | --- | --- | --- | --- | --- | --- | --- |
|  |  | ng/gram tissue weight | ng/gram tissue weight | ng/gram tissue weight | ng/gram tissue weight | ng/gram tissue weight | ng/gram tissue weight | ng/gram tissue weight | ng/gram tissue weight | ng/gram tissue weight | ng/gram tissue weight | ng/gram tissue weight | ng/gram tissue weight |
|  |  |  |  |  |  |  |  |  |  |  |  |  |  |
| ctrl, saline |  | 0,800 | 1,070 | 0,297 | 10,482 | 0,189 | 0,800 | 0,050 | 0,165 | 0,157 | 3687,143 | 0,047 | 0,040 |
|  |  | 0,800 | 1,314 | 0,310 | 11,478 | 0,144 | 0,525 | 0,050 | 0,196 | 0,201 | 6095,779 | 0,149 | 0,040 |
|  |  | 0,800 | 1,105 | 0,314 | 8,271 | 0,151 | 0,333 | 0,050 | 0,270 | 0,127 | 5335,734 | 0,067 | 0,040 |
|  |  | 0,800 | 1,375 | 0,388 | 11,397 | 0,099 | 0,800 | 0,050 | 0,305 | 0,187 | 6920,563 | 0,120 | 0,040 |
|  |  | 0,800 | 2,991 | 1,017 | 27,111 | 0,137 | 0,091 | 1,134 | 0,266 | 0,154 | 4632,087 | 0,077 | 0,040 |
|  |  | 0,800 | 4,546 | 0,807 | 41,313 | 0,109 | 0,277 | 1,968 | 0,251 | 0,378 | 9231,293 | 0,107 | 0,040 |
|  |  | 0,800 | 3,726 | 0,659 | 44,302 | 0,162 | 0,800 | 2,004 | 0,359 | 0,319 | 8738,636 | 0,121 | 0,040 |
|  |  | 0,800 | 22,029 | 4,364 | 225,355 | 0,290 | 0,800 | 2,345 | 0,765 | 0,267 | 9350,102 | 0,100 | 0,040 |
|  | *average* | *0,800* | *4,769* | *1,020* | *47,464* | *0,160* | *0,553* | *0,956* | *0,322* | *0,224* | *6748,917* | *0,098* | *0,040* |
|  | *SD* | *0,000* | *7,099* | *1,378* | *73,297* | *0,060* | *0,289* | *1,026* | *0,189* | *0,089* | *2177,676* | *0,033* | *0,000* |
|  |  |  |  |  |  |  |  |  |  |  |  |  |  |
| ctrl, LPS |  | 0,800 | 1,997 | 0,693 | 20,924 | 0,058 | 0,964 | 1,956 | 0,508 | 0,300 | 12332,068 | 0,138 | 0,040 |
|  |  | 0,800 | 1,798 | 1,474 | 32,113 | 0,153 | 0,800 | 1,917 | 0,435 | 0,157 | 11185,097 | 0,102 | 0,040 |
|  |  | 0,800 | 1,469 | 0,783 | 9,821 | 0,269 | 0,800 | 1,438 | 0,422 | 0,277 | 10958,531 | 0,132 | 0,040 |
|  |  | 0,800 | 2,273 | 1,230 | 17,028 | 0,126 | 0,800 | 0,050 | 0,349 | 0,100 | 8766,298 | 0,125 | 0,040 |
|  |  | 0,800 | 2,930 | 0,929 | 28,800 | 0,154 | 0,308 | 0,050 | 0,418 | 0,142 | 11918,034 | 0,161 | 0,040 |
|  |  | 0,800 | 3,399 | 1,082 | 26,266 | 0,192 | 0,800 | 0,050 | 0,388 | 0,147 | 10244,318 | 0,086 | 0,040 |
|  |  | 0,800 | 9,112 | 3,912 | 88,391 | 0,161 | 0,800 | 2,735 | 0,559 | 0,445 | 8168,142 | 0,103 | 0,123 |
|  |  | 0,800 | 5,252 | 2,182 | 88,555 | 0,261 | 0,284 | 1,756 | 0,764 | 0,421 | 9310,669 | 0,117 | 0,040 |
|  | *average* | *0,800* | *3,529* | *1,535* | *38,987* | *0,172* | *0,695* | *1,244* | *0,480* | *0,249* | *10360,395* | *0,120* | *0,050* |
|  | *SD* | *0,000* | *2,555* | *1,070* | *31,327* | *0,069* | *0,253* | *1,053* | *0,132* | *0,133* | *1503,052* | *0,024* | *0,029* |
|  |  |  |  |  |  |  |  |  |  |  |  |  |  |
| 1% FO, saline |  | 0,800 | 0,481 | 0,175 | 4,602 | 0,065 | 0,800 | 0,050 | 0,099 | 0,100 | 8574,707 | 0,074 | 0,040 |
|  |  | 0,800 | 0,604 | 0,173 | 7,180 | 0,035 | 0,065 | 0,050 | 0,130 | 0,100 | 11663,261 | 0,150 | 0,040 |
|  |  | 0,800 | 0,390 | 0,099 | 5,044 | 0,067 | 0,800 | 0,050 | 0,037 | 0,100 | 2890,947 | 0,102 | 0,040 |
|  |  | 0,800 | 0,610 | 0,208 | 6,857 | 0,043 | 0,290 | 0,050 | 0,102 | 0,230 | 10294,705 | 0,131 | 0,040 |
|  |  | 0,800 | 0,693 | 0,210 | 7,149 | 0,010 | 0,800 | 0,050 | 0,075 | 0,100 | 6553,654 | 0,084 | 0,040 |
|  |  | 0,800 | 0,822 | 0,228 | 9,473 | 0,031 | 0,053 | 0,050 | 0,059 | 0,109 | 10986,841 | 0,135 | 0,040 |
|  |  | 0,800 | 0,472 | 0,330 | 8,565 | 0,010 | 0,800 | 0,050 | 0,244 | 0,100 | 10422,640 | 0,086 | 0,040 |
|  |  | 0,800 | 1,949 | 0,751 | 33,703 | 0,050 | 0,118 | 0,050 | 0,210 | 0,106 | 15963,024 | 0,156 | 0,040 |
|  | *average* | *0,800* | *0,753* | *0,272* | *10,322* | *0,039* | *0,466* | *0,050* | *0,119* | *0,118* | *9668,722* | *0,115* | *0,040* |
|  | *SD* | *0,000* | *0,502* | *0,204* | *9,584* | *0,022* | *0,365* | *0,000* | *0,073* | *0,045* | *3838,394* | *0,032* | *0,000* |
|  |  |  |  |  |  |  |  |  |  |  |  |  |  |
| 1% FO, LPS |  | 0,800 | 1,442 | 1,095 | 15,485 | 0,145 | 0,066 | 3,116 | 0,939 | 0,100 | 7334,396 | 0,117 | 0,040 |
|  |  | 0,800 | 1,240 | 0,617 | 18,200 | 0,110 | 0,800 | 0,050 | 0,271 | 0,125 | 6594,671 | 0,082 | 0,040 |
|  |  | 0,800 | 1,563 | 1,000 | 18,347 | 0,010 | 0,800 | 0,050 | 0,383 | 0,103 | 9275,532 | 0,076 | 0,040 |
|  |  | 0,800 | 0,902 | 0,488 | 17,081 | 0,310 | 0,800 | 1,173 | 0,271 | 0,161 | 8682,445 | 0,155 | 0,040 |
|  |  | 0,800 | 1,677 | 0,598 | 18,781 | 0,161 | 0,800 | 0,050 | 0,319 | 0,115 | 9006,330 | 0,069 | 0,040 |
|  |  | 0,800 | 0,511 | 0,552 | 14,871 | 0,059 | 0,800 | 2,185 | 0,389 | 0,107 | 8803,309 | 0,141 | 0,040 |
|  |  | 0,800 | 2,442 | 1,324 | 25,936 | 0,121 | 0,800 | 1,464 | 0,290 | 0,124 | 5672,712 | 0,090 | 0,040 |
|  |  | 0,800 | 0,929 | 0,676 | 19,167 | 0,058 | 0,800 | 0,050 | 0,238 | 0,097 | 6044,385 | 0,104 | 0,040 |
|  | *average* | *0,800* | *1,338* | *0,794* | *18,484* | *0,122* | *0,708* | *1,017* | *0,388* | *0,117* | *7676,723* | *0,104* | *0,040* |
|  | *SD* | *0,000* | *0,591* | *0,305* | *3,385* | *0,091* | *0,260* | *1,179* | *0,229* | *0,021* | *1442,881* | *0,031* | *0,000* |
|  |  |  |  |  |  |  |  |  |  |  |  |  |  |
| 3% FO, saline |  | 0,800 | 0,578 | 0,163 | 6,657 | 0,066 | 0,800 | 0,050 | 0,121 | 0,100 | 4478,489 | 0,098 | 0,040 |
|  |  | 0,800 | 1,704 | 0,469 | 20,403 | 0,010 | 0,800 | 0,050 | 0,208 | 0,100 | 12289,325 | 0,087 | 0,040 |
|  |  | 0,800 | 0,694 | 0,248 | 7,713 | 0,060 | 0,800 | 0,050 | 0,287 | 0,121 | 8172,193 | 0,114 | 0,040 |
|  |  | 0,800 | 0,725 | 0,196 | 10,066 | 0,010 | 0,800 | 0,050 | 0,081 | 0,100 | 11059,397 | 0,132 | 0,040 |
|  |  | 0,800 | 0,929 | 0,516 | 15,155 | 0,080 | 0,800 | 1,662 | 0,295 | 0,093 | 3952,341 | 0,117 | 0,040 |
|  |  | 0,800 | 0,960 | 0,295 | 15,355 | 0,037 | 0,800 | 0,050 | 0,118 | 0,100 | 11962,094 | 0,072 | 0,040 |
|  |  | 0,800 | 0,953 | 0,395 | 14,297 | 0,068 | 0,800 | 2,156 | 0,322 | 0,244 | 6819,495 | 0,161 | 0,040 |
|  |  | 0,800 | 1,126 | 0,253 | 21,927 | 0,010 | 0,107 | 0,050 | 0,217 | 0,100 | 11628,576 | 0,155 | 0,040 |
|  | *average* | *0,800* | *0,959* | *0,317* | *13,947* | *0,043* | *0,713* | *0,515* | *0,206* | *0,120* | *8795,239* | *0,117* | *0,040* |
|  | *SD* | *0,000* | *0,349* | *0,129* | *5,555* | *0,030* | *0,245* | *0,871* | *0,091* | *0,051* | *3417,843* | *0,031* | *0,000* |
|  |  |  |  |  |  |  |  |  |  |  |  |  |  |
| 3% FO, LPS |  | 0,800 | 0,779 | 0,862 | 9,275 | 0,051 | 0,815 | 4,051 | 0,826 | 0,100 | 9542,905 | 0,110 | 0,366 |
|  |  | 0,800 | 1,397 | 0,839 | 13,842 | 0,243 | 0,800 | 3,064 | 0,620 | 0,373 | 5433,057 | 0,083 | 0,227 |
|  |  | 0,800 | 0,781 | 0,659 | 6,598 | 0,158 | 0,244 | 0,944 | 0,837 | 0,117 | 6920,076 | 0,096 | 0,141 |
|  |  | 0,800 | 1,199 | 1,122 | 11,320 | 0,265 | 0,800 | 3,464 | 0,954 | 0,284 | 11987,681 | 0,124 | 0,074 |
|  |  | 0,800 | 0,692 | 0,591 | 10,879 | 0,101 | 0,800 | 1,249 | 0,668 | 0,176 | 9270,497 | 0,084 | 0,067 |
|  |  | 0,800 | 1,055 | 0,777 | 12,972 | 0,093 | 0,800 | 0,050 | 0,729 | 0,226 | 11088,938 | 0,122 | 0,054 |
|  |  | 0,800 | 0,975 | 0,515 | 10,779 | 0,158 | 0,800 | 1,668 | 0,327 | 0,249 | 8631,741 | 0,098 | 0,054 |
|  | *average* | *0,800* | *0,982* | *0,766* | *10,809* | *0,153* | *0,723* | *2,070* | *0,709* | *0,218* | *8982,128* | *0,102* | *0,141* |
|  | *SD* | *0,000* | *0,255* | *0,203* | *2,388* | *0,079* | *0,211* | *1,474* | *0,202* | *0,096* | *2267,170* | *0,017* | *0,118* |

| **Adi. tiss.** |  | 14,15-DiHETrE | 11,12-DiHETrE | 5,6-DiHETrE | 17(S)-HDoHE | PGE3 | PGD3 | 13,14-dihydro-15-keto-PGD2 | 13,14-dihydro-15-keto-PGE2 | PGF2b | 8-iso-PGF2a | PGF2a | 10(S)-17(S)-DiHDoHE |
| --- | --- | --- | --- | --- | --- | --- | --- | --- | --- | --- | --- | --- | --- |
|  |  | ng/gram tissue weight | ng/gram tissue weight | ng/gram tissue weight | ng/gram tissue weight | ng/gram tissue weight | ng/gram tissue weight | ng/gram tissue weight | ng/gram tissue weight | ng/gram tissue weight | ng/gram tissue weight | ng/gram tissue weight | ng/gram tissue weight |
|  |  |  |  |  |  |  |  |  |  |  |  |  |  |
| ctrl, saline |  | 0,525 | 0,233 | 0,051 | 3,955 | 0,100 | 0,200 | 0,050 | 0,108 | 0,100 | 0,099 | 0,700 | 0,048 |
|  |  | 0,703 | 0,248 | 0,064 | 4,015 | 0,100 | 0,200 | 0,050 | 0,184 | 0,100 | 0,082 | 0,850 | 0,065 |
|  |  | 0,656 | 0,229 | 0,054 | 2,406 | 0,100 | 0,200 | 0,050 | 0,126 | 0,100 | 0,074 | 0,597 | 0,040 |
|  |  | 0,801 | 0,366 | 0,084 | 4,351 | 0,100 | 0,200 | 0,050 | 0,086 | 0,100 | 0,058 | 0,677 | 0,047 |
|  |  | 0,667 | 0,247 | 0,034 | 6,392 | 0,100 | 0,200 | 0,050 | 0,282 | 0,100 | 0,062 | 1,193 | 0,150 |
|  |  | 0,797 | 0,387 | 0,098 | 11,500 | 0,294 | 0,200 | 0,050 | 0,565 | 0,100 | 0,070 | 1,749 | 0,233 |
|  |  | 0,426 | 0,151 | 0,016 | 10,110 | 0,065 | 0,200 | 0,050 | 0,552 | 0,100 | 0,081 | 2,338 | 0,229 |
|  |  | 0,417 | 0,231 | 0,066 | 28,693 | 0,422 | 0,200 | 0,050 | 3,321 | 0,100 | 0,173 | 10,168 | 1,076 |
|  | *average* | *0,624* | *0,262* | *0,058* | *8,928* | *0,160* | *0,200* | *0,050* | *0,653* | *0,100* | *0,087* | *2,284* | *0,236* |
|  | *SD* | *0,152* | *0,078* | *0,026* | *8,598* | *0,127* | *0,000* | *0,000* | *1,095* | *0,000* | *0,037* | *3,243* | *0,349* |
|  |  |  |  |  |  |  |  |  |  |  |  |  |  |
| ctrl, LPS |  | 0,527 | 0,259 | 0,078 | 11,950 | 0,100 | 0,200 | 0,050 | 0,223 | 0,100 | 0,042 | 1,191 | 0,157 |
|  |  | 0,652 | 0,382 | 0,096 | 2,283 | 0,100 | 0,200 | 0,050 | 0,394 | 0,100 | 0,124 | 2,601 | 0,060 |
|  |  | 0,425 | 0,189 | 0,075 | 6,881 | 0,100 | 0,200 | 0,050 | 0,343 | 0,100 | 0,084 | 1,607 | 0,097 |
|  |  | 0,543 | 0,267 | 0,061 | 6,555 | 0,100 | 0,200 | 0,050 | 0,892 | 0,100 | 0,106 | 2,241 | 0,072 |
|  |  | 0,647 | 0,314 | 0,170 | 13,700 | 0,100 | 0,200 | 0,050 | 1,220 | 0,100 | 0,065 | 1,869 | 0,166 |
|  |  | 0,329 | 0,221 | 0,071 | 10,816 | 0,100 | 0,200 | 0,050 | 0,609 | 0,100 | 0,084 | 2,152 | 0,163 |
|  |  | 0,378 | 0,222 | 0,039 | 23,262 | 0,405 | 0,200 | 0,050 | 1,087 | 0,100 | 0,214 | 8,468 | 0,548 |
|  |  | 0,305 | 0,171 | 0,017 | 13,649 | 0,155 | 0,200 | 0,050 | 0,713 | 0,100 | 0,093 | 4,208 | 0,242 |
|  | *average* | *0,476* | *0,253* | *0,076* | *11,137* | *0,145* | *0,200* | *0,050* | *0,685* | *0,100* | *0,102* | *3,042* | *0,188* |
|  | *SD* | *0,137* | *0,069* | *0,045* | *6,304* | *0,107* | *0,000* | *0,000* | *0,361* | *0,000* | *0,052* | *2,369* | *0,157* |
|  |  |  |  |  |  |  |  |  |  |  |  |  |  |
| 1% FO, saline |  | 0,115 | 0,088 | 0,009 | 9,668 | 0,100 | 0,200 | 0,050 | 0,118 | 0,100 | 0,060 | 0,395 | 0,185 |
|  |  | 0,158 | 0,106 | 0,017 | 20,913 | 0,100 | 0,200 | 0,050 | 0,100 | 0,100 | 0,073 | 0,682 | 0,208 |
|  |  | 0,077 | 0,065 | 0,010 | 3,688 | 0,100 | 0,200 | 0,050 | 0,100 | 0,100 | 0,039 | 0,470 | 0,067 |
|  |  | 0,323 | 0,158 | 0,018 | 28,207 | 0,498 | 0,465 | 0,050 | 0,103 | 0,100 | 0,059 | 0,672 | 0,331 |
|  |  | 0,116 | 0,067 | 0,010 | 7,636 | 0,100 | 0,200 | 0,050 | 0,100 | 0,100 | 0,079 | 0,789 | 0,164 |
|  |  | 0,202 | 0,081 | 0,032 | 32,908 | 0,621 | 0,204 | 0,050 | 0,078 | 0,100 | 0,047 | 0,657 | 0,434 |
|  |  | 0,258 | 0,065 | 0,005 | 3,706 | 0,828 | 1,281 | 0,050 | 0,066 | 0,100 | 0,072 | 1,493 | 0,060 |
|  |  | 0,205 | 0,104 | 0,025 | 35,715 | 1,653 | 1,590 | 0,050 | 0,117 | 0,100 | 0,047 | 0,947 | 0,743 |
|  | *average* | *0,182* | *0,092* | *0,016* | *17,805* | *0,500* | *0,542* | *0,050* | *0,098* | *0,100* | *0,059* | *0,763* | *0,274* |
|  | *SD* | *0,082* | *0,031* | *0,009* | *13,280* | *0,547* | *0,565* | *0,000* | *0,018* | *0,000* | *0,014* | *0,341* | *0,227* |
|  |  |  |  |  |  |  |  |  |  |  |  |  |  |
| 1% FO, LPS |  | 0,283 | 0,155 | 0,099 | 26,457 | 3,624 | 0,997 | 0,050 | 0,100 | 0,100 | 0,041 | 1,521 | 0,838 |
|  |  | 0,259 | 0,088 | 0,012 | 29,124 | 3,161 | 0,793 | 0,050 | 0,101 | 0,100 | 0,059 | 0,865 | 1,347 |
|  |  | 0,268 | 0,149 | 0,039 | 31,772 | 5,361 | 2,471 | 0,050 | 0,382 | 0,100 | 0,067 | 1,676 | 0,892 |
|  |  | 0,170 | 0,067 | 0,027 | 21,143 | 2,599 | 0,740 | 0,050 | 0,068 | 0,100 | 0,056 | 1,085 | 0,674 |
|  |  | 0,212 | 0,077 | 0,018 | 45,606 | 2,078 | 0,420 | 0,050 | 0,103 | 0,100 | 0,051 | 1,231 | 1,667 |
|  |  | 0,298 | 0,155 | 0,031 | 13,306 | 2,683 | 0,741 | 0,050 | 0,369 | 0,100 | 0,071 | 1,057 | 0,412 |
|  |  | 0,191 | 0,075 | 0,026 | 104,289 | 3,896 | 1,349 | 0,050 | 0,112 | 0,100 | 0,066 | 2,631 | 2,767 |
|  |  | 0,170 | 0,060 | 0,033 | 18,315 | 3,375 | 0,748 | 0,050 | 0,100 | 0,100 | 0,064 | 1,238 | 0,577 |
|  | *average* | *0,231* | *0,103* | *0,036* | *36,252* | *3,347* | *1,032* | *0,050* | *0,167* | *0,100* | *0,060* | *1,413* | *1,147* |
|  | *SD* | *0,052* | *0,042* | *0,027* | *29,179* | *1,006* | *0,638* | *0,000* | *0,129* | *0,000* | *0,010* | *0,556* | *0,773* |
|  |  |  |  |  |  |  |  |  |  |  |  |  |  |
| 3% FO, saline |  | 0,143 | 0,053 | 0,015 | 14,696 | 0,293 | 0,212 | 0,050 | 0,100 | 0,100 | 0,042 | 0,898 | 0,409 |
|  |  | 0,186 | 0,076 | 0,011 | 44,092 | 2,527 | 0,589 | 0,050 | 0,125 | 0,100 | 0,054 | 0,936 | 1,197 |
|  |  | 0,094 | 0,043 | 0,012 | 12,809 | 0,934 | 0,551 | 0,050 | 0,100 | 0,100 | 0,048 | 0,693 | 0,305 |
|  |  | 0,083 | 0,034 | 0,008 | 13,140 | 0,652 | 0,336 | 0,050 | 0,100 | 0,100 | 0,068 | 0,948 | 0,172 |
|  |  | 0,405 | 0,140 | 0,015 | 28,172 | 5,171 | 3,182 | 0,050 | 0,176 | 0,100 | 0,053 | 0,709 | 0,897 |
|  |  | 0,098 | 0,062 | 0,014 | 15,423 | 1,942 | 0,280 | 0,050 | 0,100 | 0,100 | 0,042 | 0,755 | 0,535 |
|  |  | 0,698 | 0,259 | 0,058 | 52,407 | 3,764 | 1,925 | 0,050 | 0,100 | 0,100 | 0,041 | 1,133 | 0,889 |
|  |  | 0,164 | 0,093 | 0,018 | 33,882 | 1,433 | 0,361 | 0,050 | 0,100 | 0,100 | 0,059 | 0,737 | 1,147 |
|  | *average* | *0,234* | *0,095* | *0,019* | *26,828* | *2,090* | *0,930* | *0,050* | *0,113* | *0,100* | *0,051* | *0,851* | *0,694* |
|  | *SD* | *0,214* | *0,074* | *0,016* | *15,423* | *1,672* | *1,065* | *0,000* | *0,027* | *0,000* | *0,010* | *0,154* | *0,391* |
|  |  |  |  |  |  |  |  |  |  |  |  |  |  |
| 3% FO, LPS |  | 0,901 | 0,238 | 0,069 | 28,380 | 10,739 | 4,965 | 0,050 | 0,472 | 0,100 | 0,069 | 1,605 | 1,147 |
|  |  | 0,771 | 0,270 | 0,054 | 49,823 | 8,077 | 2,734 | 0,050 | 0,221 | 0,100 | 0,047 | 0,954 | 2,112 |
|  |  | 0,414 | 0,138 | 0,037 | 45,815 | 6,677 | 2,095 | 0,050 | 0,211 | 0,100 | 0,065 | 1,125 | 1,561 |
|  |  | 1,062 | 0,290 | 0,048 | 17,741 | 12,051 | 3,722 | 0,050 | 0,122 | 0,100 | 0,079 | 1,041 | 0,657 |
|  |  | 0,556 | 0,182 | 0,039 | 18,761 | 6,568 | 1,676 | 0,050 | 0,140 | 0,100 | 0,089 | 1,319 | 0,905 |
|  |  | 0,532 | 0,193 | 0,038 | 53,805 | 5,088 | 1,717 | 0,050 | 0,279 | 0,100 | 0,060 | 1,958 | 1,147 |
|  |  | 0,684 | 0,217 | 0,064 | 44,791 | 7,522 | 3,709 | 0,050 | 0,100 | 0,100 | 0,068 | 0,715 | 2,080 |
|  | *average* | *0,703* | *0,219* | *0,050* | *37,017* | *8,103* | *2,945* | *0,050* | *0,221* | *0,100* | *0,068* | *1,245* | *1,373* |
|  | *SD* | *0,226* | *0,053* | *0,013* | *15,073* | *2,462* | *1,235* | *0,000* | *0,127* | *0,000* | *0,013* | *0,421* | *0,565* |

| **Adi. tiss.** |  | 19,20-DiHoPE | TBXB3 | TBXB2 | 8,9-DiHETrE | 13,14-dihydro-15-keto-PGF2a | lipoxin A4 | PGE2 | PGD2 | Leukotriene E4 | n-acetyl leukotriene E4 | Leukotriene D4 |
| --- | --- | --- | --- | --- | --- | --- | --- | --- | --- | --- | --- | --- |
|  |  | ng/gram tissue weight | ng/gram tissue weight | ng/gram tissue weight | ng/gram tissue weight | ng/gram tissue weight | ng/gram tissue weight | ng/gram tissue weight | ng/gram tissue weight | ng/gram tissue weight | ng/gram tissue weight | ng/gram tissue weight |
|  |  |  |  |  |  |  |  |  |  |  |  |  |
| ctrl, saline |  | 0,584 | 0,048 | 0,094 | 0,401 | 0,278 | 0,040 | 0,666 | 1,359 | 0,800 | 0,200 | 0,500 |
|  |  | 0,793 | 0,044 | 0,114 | 0,490 | 0,200 | 0,040 | 0,990 | 1,353 | 0,800 | 0,200 | 0,500 |
|  |  | 0,428 | 0,026 | 0,053 | 0,397 | 0,224 | 0,040 | 0,451 | 1,565 | 0,800 | 0,200 | 0,500 |
|  |  | 0,913 | 0,032 | 0,071 | 0,471 | 0,200 | 0,040 | 0,928 | 2,393 | 0,800 | 0,200 | 0,500 |
|  |  | 0,356 | 0,037 | 0,214 | 0,346 | 0,325 | 0,040 | 1,678 | 4,392 | 0,800 | 0,200 | 0,500 |
|  |  | 1,132 | 0,032 | 0,555 | 0,511 | 0,335 | 0,040 | 2,802 | 5,485 | 0,800 | 0,200 | 0,500 |
|  |  | 0,506 | 0,021 | 0,606 | 0,253 | 0,441 | 0,040 | 3,131 | 2,483 | 0,800 | 0,200 | 0,500 |
|  |  | 0,757 | 0,080 | 7,582 | 0,243 | 1,042 | 0,040 | 53,193 | 15,629 | 6,602 | 0,200 | 5,079 |
|  | *average* | *0,684* | *0,040* | *1,161* | *0,389* | *0,381* | *0,040* | *7,980* | *4,332* | *1,525* | *0,200* | *1,072* |
|  | *SD* | *0,263* | *0,018* | *2,604* | *0,102* | *0,280* | *0,000* | *18,295* | *4,804* | *2,051* | *0,000* | *1,619* |
|  |  |  |  |  |  |  |  |  |  |  |  |  |
| ctrl, LPS |  | 0,994 | 0,049 | 0,268 | 0,280 | 0,375 | 0,040 | 2,317 | 1,146 | 0,800 | 0,200 | 0,500 |
|  |  | 0,677 | 0,033 | 2,421 | 0,359 | 0,460 | 0,040 | 6,870 | 7,301 | 0,800 | 0,200 | 0,500 |
|  |  | 0,437 | 0,032 | 0,241 | 0,178 | 0,355 | 0,040 | 2,589 | 1,355 | 0,800 | 0,200 | 0,500 |
|  |  | 0,789 | 0,040 | 1,725 | 0,356 | 1,240 | 0,040 | 11,714 | 7,649 | 0,800 | 0,200 | 0,693 |
|  |  | 1,127 | 0,052 | 0,854 | 0,421 | 1,119 | 0,040 | 4,961 | 2,765 | 0,800 | 0,200 | 0,709 |
|  |  | 0,643 | 0,038 | 0,989 | 0,210 | 0,628 | 0,040 | 8,197 | 5,795 | 0,800 | 0,200 | 0,500 |
|  |  | 0,616 | 0,060 | 6,807 | 0,254 | 1,007 | 0,040 | 48,617 | 12,569 | 3,711 | 0,200 | 2,797 |
|  |  | 0,495 | 0,058 | 3,432 | 0,239 | 0,935 | 0,040 | 12,624 | 11,277 | 0,984 | 0,200 | 0,815 |
|  | *average* | *0,722* | *0,045* | *2,092* | *0,287* | *0,765* | *0,040* | *12,236* | *6,232* | *1,187* | *0,200* | *0,877* |
|  | *SD* | *0,237* | *0,011* | *2,197* | *0,084* | *0,353* | *0,000* | *15,182* | *4,320* | *1,022* | *0,000* | *0,786* |
|  |  |  |  |  |  |  |  |  |  |  |  |  |
| 1% FO, saline |  | 1,500 | 0,047 | 0,058 | 0,030 | 0,200 | 0,040 | 0,244 | 0,812 | 0,800 | 0,200 | 0,500 |
|  |  | 1,836 | 0,020 | 0,088 | 0,109 | 0,200 | 0,040 | 0,371 | 0,617 | 0,800 | 0,200 | 0,500 |
|  |  | 0,420 | 0,025 | 0,063 | 0,032 | 0,200 | 0,040 | 0,196 | 0,311 | 0,800 | 0,200 | 0,500 |
|  |  | 2,902 | 0,080 | 0,165 | 0,086 | 0,200 | 0,040 | 0,656 | 1,371 | 0,800 | 0,200 | 0,500 |
|  |  | 0,405 | 0,050 | 0,099 | 0,113 | 0,200 | 0,040 | 0,345 | 1,339 | 0,800 | 0,200 | 0,500 |
|  |  | 2,357 | 0,070 | 0,114 | 0,083 | 0,200 | 0,040 | 0,635 | 0,310 | 0,800 | 0,200 | 0,500 |
|  |  | 2,165 | 0,083 | 0,252 | 0,140 | 0,436 | 0,040 | 0,726 | 2,750 | 0,800 | 0,200 | 0,500 |
|  |  | 3,613 | 0,148 | 0,361 | 0,202 | 0,200 | 0,040 | 1,767 | 4,130 | 0,800 | 0,200 | 0,500 |
|  | *average* | *1,900* | *0,065* | *0,150* | *0,099* | *0,229* | *0,040* | *0,617* | *1,455* | *0,800* | *0,200* | *0,500* |
|  | *SD* | *1,122* | *0,041* | *0,106* | *0,056* | *0,083* | *0,000* | *0,505* | *1,341* | *0,000* | *0,000* | *0,000* |
|  |  |  |  |  |  |  |  |  |  |  |  |  |
| 1% FO, LPS |  | 2,606 | 0,378 | 0,764 | 0,319 | 0,200 | 0,040 | 4,527 | 3,734 | 0,800 | 0,200 | 1,248 |
|  |  | 6,507 | 0,223 | 0,463 | 0,167 | 0,200 | 0,040 | 2,735 | 1,372 | 0,800 | 0,200 | 0,500 |
|  |  | 4,297 | 0,496 | 0,728 | 0,199 | 0,412 | 0,040 | 4,489 | 5,485 | 0,800 | 0,200 | 0,678 |
|  |  | 1,942 | 0,285 | 0,363 | 0,05 | 0,473 | 0,040 | 2,558 | 1,769 | 0,800 | 0,200 | 0,500 |
|  |  | 3,234 | 0,172 | 0,386 | 0,155 | 0,200 | 0,040 | 3,129 | 1,162 | 0,800 | 0,200 | 1,466 |
|  |  | 10,916 | 0,212 | 0,309 | 0,174 | 0,615 | 0,098 | 1,581 | 1,902 | 0,800 | 0,200 | 0,500 |
|  |  | 3,168 | 0,552 | 1,523 | 0,115 | 0,200 | 0,040 | 6,349 | 7,860 | 0,800 | 0,200 | 0,608 |
|  |  | 2,142 | 0,431 | 1,084 | 0,115 | 0,200 | 0,040 | 4,734 | 3,419 | 0,800 | 0,200 | 0,958 |
|  | *average* | *4,351* | *0,344* | *0,703* | *0,178* | *0,312* | *0,047* | *3,763* | *3,338* | *0,800* | *0,200* | *0,807* |
|  | *SD* | *3,027* | *0,142* | *0,423* | *0,069* | *0,165* | *0,020* | *1,531* | *2,340* | *0,000* | *0,000* | *0,376* |
|  |  |  |  |  |  |  |  |  |  |  |  |  |
| 3% FO, saline |  | 2,056 | 0,033 | 0,046 | 0,053 | 0,200 | 0,040 | 0,464 | 0,636 | 0,800 | 0,200 | 0,500 |
|  |  | 4,050 | 0,122 | 0,207 | 0,076 | 0,200 | 0,040 | 1,089 | 1,520 | 0,800 | 0,200 | 0,500 |
|  |  | 1,924 | 0,079 | 0,082 | 0,031 | 0,200 | 0,139 | 0,738 | 1,440 | 0,800 | 0,200 | 0,500 |
|  |  | 1,071 | 0,119 | 0,276 | 0,024 | 0,200 | 0,106 | 0,467 | 1,210 | 0,800 | 0,200 | 0,500 |
|  |  | 6,231 | 0,317 | 0,222 | 0,140 | 0,200 | 0,380 | 1,553 | 2,895 | 0,800 | 0,200 | 0,500 |
|  |  | 1,460 | 0,058 | 0,196 | 0,043 | 0,200 | 0,116 | 1,093 | 1,042 | 0,800 | 0,200 | 0,500 |
|  |  | 14,937 | 0,320 | 0,268 | 0,224 | 0,200 | 0,307 | 1,475 | 2,360 | 0,800 | 0,200 | 0,500 |
|  |  | 4,355 | 0,091 | 0,206 | 0,087 | 0,200 | 0,040 | 0,939 | 0,864 | 0,800 | 0,200 | 0,500 |
|  | *average* | *4,511* | *0,143* | *0,188* | *0,085* | *0,200* | *0,146* | *0,977* | *1,496* | *0,800* | *0,200* | *0,500* |
|  | *SD* | *4,563* | *0,113* | *0,082* | *0,067* | *0,000* | *0,129* | *0,412* | *0,769* | *0,000* | *0,000* | *0,000* |
|  |  |  |  |  |  |  |  |  |  |  |  |  |
| 3% FO, LPS |  | 28,629 | 0,953 | 0,652 | 0,275 | 0,680 | 0,407 | 4,179 | 5,257 | 5,928 | 0,200 | 8,006 |
|  |  | 27,269 | 0,895 | 0,655 | 0,350 | 0,200 | 0,574 | 2,948 | 2,165 | 4,621 | 0,200 | 2,659 |
|  |  | 15,323 | 0,776 | 0,582 | 0,167 | 0,200 | 0,603 | 2,835 | 2,660 | 1,316 | 0,200 | 6,024 |
|  |  | 27,860 | 0,929 | 0,682 | 0,447 | 0,200 | 0,666 | 4,088 | 4,325 | 2,580 | 0,200 | 2,541 |
|  |  | 15,078 | 0,551 | 0,476 | 0,435 | 0,200 | 0,355 | 2,996 | 2,521 | 0,800 | 0,200 | 1,659 |
|  |  | 15,119 | 0,573 | 0,907 | 0,167 | 0,200 | 0,175 | 5,092 | 4,171 | 0,800 | 0,200 | 2,167 |
|  |  | 24,988 | 0,843 | 0,374 | 0,487 | 0,200 | 0,362 | 2,505 | 2,496 | 0,800 | 0,200 | 0,627 |
|  | *average* | *22,038* | *0,788* | *0,618* | *0,332* | *0,269* | *0,449* | *3,520* | *3,371* | *2,406* | *0,200* | *3,383* |
|  | *SD* | *6,517* | *0,165* | *0,169* | *0,133* | *0,181* | *0,173* | *0,942* | *1,194* | *2,092* | *0,000* | *2,634* |

| **Adi. tiss.** |  | 17 keto- 4(z), 7(z), 10(z), 13 (z), 15 (E), 19(z)-DHA | 12,13-DiHOME | 9,10-DiHOME | 9,12,13-TriHOME | 9,10,13-TriHOME | UK1 | UK2 | UK3 | UK4 | UK5 |
| --- | --- | --- | --- | --- | --- | --- | --- | --- | --- | --- | --- |
|  |  | ng/gram tissue weight | ng/gram tissue weight | ng/gram tissue weight | RR | RR | RR | RR | RR | RR | RR |
|  |  |  |  |  |  |  |  |  |  |  |  |
| ctrl, saline |  | 2,300 | 1015,214 | 487,515 | 11181,259 | 11268,515 | 3,164 | 0,154 | 0,081 | 0,074 | 0,090 |
|  |  | 2,300 | 1359,179 | 543,413 | 4436,586 | 4241,513 | 0,703 | 0,167 | 0,099 | 0,083 | 0,105 |
|  |  | 2,300 | 1416,818 | 608,099 | 4508,794 | 4092,408 | 1,319 | 0,182 | 0,077 | 0,086 | 0,092 |
|  |  | 2,300 | 1421,549 | 684,278 | 3210,464 | 2931,860 | 0,707 | 0,126 | 0,098 | 0,071 | 0,099 |
|  |  | 2,300 | 1372,767 | 556,640 | 7684,643 | 5988,635 | 1,624 | 0,224 | 0,100 | 0,099 | 0,124 |
|  |  | 2,300 | 1298,832 | 684,010 | 5952,668 | 3880,942 | 1,137 | 0,184 | 0,099 | 0,075 | 0,128 |
|  |  | 2,300 | 914,639 | 441,757 | 13498,413 | 10077,432 | 5,826 | 0,177 | 0,119 | 0,080 | 0,142 |
|  |  | 2,300 | 1282,789 | 520,774 | 17684,412 | 5961,730 | 2,396 | 0,131 | 0,091 | 0,045 | 0,122 |
|  | *average* | *2,300* | *1260,224* | *565,811* | *8519,655* | *6055,379* | *2,110* | *0,168* | *0,096* | *0,077* | *0,113* |
|  | *SD* | *0,000* | *190,662* | *87,772* | *5128,319* | *3047,126* | *1,722* | *0,032* | *0,013* | *0,016* | *0,019* |
|  |  |  |  |  |  |  |  |  |  |  |  |
| ctrl, LPS |  | 2,300 | 973,132 | 449,711 | 6103,647 | 4854,570 | 1,069 | 0,174 | 0,157 | 0,095 | 0,127 |
|  |  | 2,300 | 1329,449 | 731,583 | 7004,267 | 6136,460 | 1,771 | 0,236 | 0,204 | 0,129 | 0,247 |
|  |  | 2,300 | 674,158 | 464,814 | 7531,542 | 6320,656 | 1,738 | 0,234 | 0,183 | 0,136 | 0,162 |
|  |  | 2,300 | 1440,525 | 662,620 | 4884,271 | 3824,110 | 1,275 | 0,174 | 0,145 | 0,131 | 0,169 |
|  |  | 2,300 | 1501,389 | 664,613 | 4886,842 | 3694,209 | 1,051 | 0,216 | 0,167 | 0,106 | 0,136 |
|  |  | 2,300 | 730,414 | 444,301 | 5571,415 | 3893,082 | 1,124 | 0,225 | 0,156 | 0,126 | 0,181 |
|  |  | 2,300 | 618,126 | 323,528 | 6036,393 | 3129,567 | 0,871 | 0,111 | 0,079 | 0,063 | 0,086 |
|  |  | 2,300 | 541,594 | 338,752 | 7647,807 | 5054,164 | 1,380 | 0,296 | 0,239 | 0,148 | 0,237 |
|  | *average* | *2,300* | *976,098* | *509,990* | *6208,273* | *4613,352* | *1,285* | *0,208* | *0,166* | *0,117* | *0,168* |
|  | *SD* | *0,000* | *393,692* | *156,017* | *1095,585* | *1175,669* | *0,327* | *0,055* | *0,047* | *0,027* | *0,054* |
|  |  |  |  |  |  |  |  |  |  |  |  |
| 1% FO, saline |  | 2,300 | 282,057 | 162,056 | 4075,711 | 3568,588 | 1,286 | 0,070 | 0,055 | 0,038 | 0,074 |
|  |  | 2,300 | 559,524 | 260,619 | 1598,079 | 1325,766 | 0,331 | 0,073 | 0,065 | 0,015 | 0,046 |
|  |  | 2,300 | 118,443 | 102,271 | 1772,365 | 1466,889 | 0,415 | 0,065 | 0,031 | 0,010 | 0,042 |
|  |  | 2,300 | 901,967 | 462,058 | 2942,786 | 2289,953 | 0,984 | 0,150 | 0,082 | 0,060 | 0,123 |
|  |  | 2,300 | 174,487 | 124,241 | 2583,183 | 1952,214 | 0,513 | 0,055 | 0,049 | 0,018 | 0,067 |
|  |  | 2,300 | 738,296 | 363,732 | 3312,932 | 2519,457 | 0,852 | 0,179 | 0,064 | 0,062 | 0,130 |
|  |  | 2,300 | 513,860 | 278,168 | 2802,476 | 2420,221 | 1,851 | 0,087 | 0,102 | 0,037 | 0,074 |
|  |  | 2,300 | 706,474 | 356,183 | 3701,559 | 2462,530 | 1,097 | 0,144 | 0,109 | 0,022 | 0,112 |
|  | *average* | *2,300* | *499,388* | *263,666* | *2848,636* | *2250,702* | *0,916* | *0,103* | *0,070* | *0,033* | *0,084* |
|  | *SD* | *0,000* | *283,793* | *127,595* | *866,230* | *701,342* | *0,508* | *0,047* | *0,027* | *0,020* | *0,034* |
|  |  |  |  |  |  |  |  |  |  |  |  |
| 1% FO, LPS |  | 58,144 | 756,144 | 481,666 | 9355,009 | 6827,191 | 2,834 | 0,235 | 0,241 | 0,109 | 0,204 |
|  |  | 2,300 | 1248,203 | 469,628 | 5064,663 | 2687,613 | 0,994 | 0,158 | 0,146 | 0,079 | 0,145 |
|  |  | 100,780 | 1067,896 | 443,471 | 5091,771 | 3847,745 | 2,008 | 0,178 | 0,139 | 0,073 | 0,137 |
|  |  | 2,300 | 404,532 | 293,829 | 6951,600 | 5078,248 | 1,176 | 0,238 | 0,188 | 0,120 | 0,147 |
|  |  | 35,753 | 848,442 | 364,528 | 6428,806 | 4775,312 | 1,025 | 0,203 | 0,154 | 0,096 | 0,122 |
|  |  | 137,685 | 1614,336 | 651,435 | 5050,552 | 4130,087 | 1,559 | 0,256 | 0,231 | 0,166 | 0,165 |
|  |  | 2,300 | 793,829 | 433,751 | 10898,849 | 8040,717 | 1,108 | 0,233 | 0,242 | 0,104 | 0,223 |
|  |  | 2,300 | 483,911 | 290,971 | 6786,718 | 5182,021 | 1,320 | 0,278 | 0,258 | 0,137 | 0,144 |
|  | *average* | *42,695* | *902,161* | *428,660* | *6953,496* | *5071,117* | *1,503* | *0,222* | *0,200* | *0,110* | *0,161* |
|  | *SD* | *52,387* | *398,733* | *116,872* | *2147,793* | *1693,071* | *0,634* | *0,040* | *0,049* | *0,031* | *0,035* |
|  |  |  |  |  |  |  |  |  |  |  |  |
| 3% FO, saline |  | 2,300 | 154,854 | 87,845 | 2517,538 | 2337,480 | 0,448 | 0,043 | 0,075 | 0,039 | 0,055 |
|  |  | 2,300 | 252,761 | 153,082 | 3878,456 | 3566,084 | 0,850 | 0,072 | 0,062 | 0,029 | 0,069 |
|  |  | 2,300 | 191,263 | 123,032 | 2667,176 | 2516,006 | 0,580 | 0,028 | 0,043 | 0,024 | 0,052 |
|  |  | 2,300 | 148,192 | 91,766 | 3019,246 | 2301,723 | 0,628 | 0,042 | 0,045 | 0,018 | 0,054 |
|  |  | 2,300 | 924,505 | 333,823 | 2888,199 | 2375,997 | 1,080 | 0,194 | 0,140 | 0,078 | 0,109 |
|  |  | 2,300 | 148,570 | 106,741 | 2649,559 | 1881,746 | 0,940 | 0,048 | 0,029 | 0,018 | 0,042 |
|  |  | 2,300 | 1417,315 | 466,236 | 3967,275 | 2779,203 | 0,901 | 0,141 | 0,150 | 0,079 | 0,128 |
|  |  | 2,300 | 349,179 | 198,141 | 8409,035 | 5991,313 | 0,671 | 0,052 | 0,065 | 0,029 | 0,065 |
|  | *average* | *2,300* | *448,330* | *195,083* | *3749,560* | *2968,694* | *0,762* | *0,078* | *0,076* | *0,039* | *0,072* |
|  | *SD* | *0,000* | *469,940* | *136,128* | *1962,122* | *1315,293* | *0,213* | *0,059* | *0,045* | *0,025* | *0,030* |
|  |  |  |  |  |  |  |  |  |  |  |  |
| 3% FO, LPS |  | 304,400 | 2153,626 | 714,934 | 3530,973 | 2964,276 | 1,269 | 0,183 | 0,159 | 0,085 | 0,171 |
|  |  | 291,405 | 2214,148 | 800,480 | 5692,567 | 4891,836 | 1,756 | 0,339 | 0,191 | 0,112 | 0,203 |
|  |  | 218,072 | 1277,340 | 461,969 | 5018,906 | 4108,661 | 0,956 | 0,208 | 0,166 | 0,110 | 0,169 |
|  |  | 254,718 | 2596,523 | 900,135 | 7650,641 | 5784,086 | 1,981 | 0,399 | 0,275 | 0,188 | 0,283 |
|  |  | 86,933 | 1635,601 | 566,992 | 3757,700 | 2877,741 | 1,070 | 0,233 | 0,133 | 0,124 | 0,129 |
|  |  | 144,886 | 2398,804 | 896,605 | 7501,698 | 6106,634 | 1,635 | 0,356 | 0,272 | 0,154 | 0,254 |
|  |  | 130,046 | 1962,202 | 669,797 | 4985,804 | 3688,535 | 1,402 | 0,278 | 0,240 | 0,164 | 0,211 |
|  | *average* | *204,351* | *2034,035* | *715,845* | *5448,327* | *4345,967* | *1,439* | *0,285* | *0,205* | *0,134* | *0,203* |
|  | *SD* | *84,811* | *453,264* | *164,586* | *1635,999* | *1292,246* | *0,372* | *0,082* | *0,057* | *0,036* | *0,053* |

| **Adi. tiss.** |  | epea | dhea | aea | 2-ag | dle | pea | oea | sea |
| --- | --- | --- | --- | --- | --- | --- | --- | --- | --- |
|  |  | ng/gram tissue weight | ng/gram tissue weight | ng/gram tissue weight | ng/gram tissue weight | ng/gram tissue weight | ng/gram tissue weight | ng/gram tissue weight | ng/gram tissue weight |
|  |  |  |  |  |  |  |  |  |  |
| ctrl, saline |  | 0,100 | 2,332 | 3,913 | 128,385 | 1,062 | 32,989 | 98,379 | 23,331 |
|  |  | 0,100 | 2,298 | 3,142 | 182,905 | 0,778 | 31,523 | 98,635 | 22,495 |
|  |  | 0,100 | 2,382 | 3,251 | 238,407 | 0,836 | 31,203 | 105,012 | 27,107 |
|  |  | 0,100 | 1,949 | 3,469 | 163,916 | 1,045 | 35,891 | 108,850 | 35,672 |
|  |  | 0,100 | 2,311 | 3,093 | 166,472 | 0,745 | 29,213 | 98,040 | 24,402 |
|  |  | 0,100 | 2,372 | 3,822 | 229,567 | 1,135 | 36,376 | 110,786 | 30,036 |
|  |  | 0,100 | 2,063 | 2,424 | 211,190 | 0,694 | 32,571 | 109,981 | 20,614 |
|  |  | 0,100 | 2,328 | 2,622 | 451,197 | 0,521 | 38,314 | 107,278 | 20,556 |
|  | *average* | *0,100* | *2,254* | *3,217* | *221,505* | *0,852* | *33,510* | *104,620* | *25,527* |
|  | *SD* | *0,000* | *0,159* | *0,523* | *99,801* | *0,211* | *3,067* | *5,476* | *5,208* |
|  |  |  |  |  |  |  |  |  |  |
| ctrl, LPS |  | 0,100 | 3,016 | 3,848 | 180,064 | 1,027 | 52,595 | 196,225 | 22,299 |
|  |  | 0,100 | 2,276 | 2,173 | 281,049 | 0,478 | 27,043 | 91,307 | 15,466 |
|  |  | 0,100 | 2,326 | 2,683 | 241,204 | 0,734 | 40,955 | 142,988 | 20,284 |
|  |  | 0,100 | 2,261 | 2,635 | 294,399 | 0,562 | 27,509 | 111,281 | 15,300 |
|  |  | 0,100 | 2,782 | 2,432 | 321,928 | 0,838 | 35,860 | 135,900 | 17,143 |
|  |  | 0,100 | 2,537 | 3,132 | 251,663 | 0,908 | 36,422 | 125,688 | 20,127 |
|  |  | 0,100 | 2,552 | 3,016 | 522,894 | 0,776 | 37,056 | 145,874 | 17,862 |
|  |  | 0,100 | 2,388 | 2,793 | 373,702 | 0,699 | 37,208 | 144,155 | 17,844 |
|  | *average* | *0,100* | *2,517* | *2,839* | *308,363* | *0,753* | *36,831* | *136,677* | *18,291* |
|  | *SD* | *0,000* | *0,267* | *0,509* | *103,925* | *0,178* | *8,013* | *30,592* | *2,451* |
|  |  |  |  |  |  |  |  |  |  |
| 1% FO, saline |  | 2,257 | 6,573 | 0,825 | 139,838 | 0,353 | 24,469 | 71,268 | 35,200 |
|  |  | 2,200 | 6,371 | 0,909 | 89,940 | 0,472 | 30,722 | 76,046 | 34,022 |
|  |  | 3,305 | 5,940 | 1,129 | 134,598 | 0,363 | 14,913 | 46,044 | 35,349 |
|  |  | 2,890 | 6,708 | 1,058 | 91,287 | 0,382 | 30,969 | 82,973 | 24,363 |
|  |  | 2,677 | 5,940 | 1,174 | 103,240 | 0,384 | 17,113 | 50,282 | 29,804 |
|  |  | 2,854 | 6,303 | 1,250 | 76,798 | 0,364 | 28,009 | 77,329 | 22,962 |
|  |  | 3,152 | 6,680 | 1,325 | 125,066 | 0,437 | 37,360 | 87,828 | 29,152 |
|  |  | 3,126 | 7,230 | 1,042 | 116,613 | 0,410 | 35,703 | 85,998 | 38,028 |
|  | *average* | *2,808* | *6,468* | *1,089* | *109,672* | *0,395* | *27,407* | *72,221* | *31,110* |
|  | *SD* | *0,408* | *0,429* | *0,167* | *22,884* | *0,041* | *8,130* | *15,844* | *5,457* |
|  |  |  |  |  |  |  |  |  |  |
| 1% FO, LPS |  | 6,979 | 7,708 | 1,276 | 106,313 | 0,529 | 56,334 | 150,716 | 21,452 |
|  |  | 4,728 | 7,663 | 0,795 | 112,376 | 0,431 | 42,664 | 141,051 | 21,272 |
|  |  | 3,106 | 7,262 | 0,801 | 181,247 | 0,390 | 38,005 | 116,756 | 20,114 |
|  |  | 7,140 | 9,057 | 0,928 | 141,538 | 0,529 | 57,026 | 172,471 | 20,567 |
|  |  | 3,188 | 7,064 | 0,783 | 135,406 | 0,410 | 36,654 | 113,961 | 17,963 |
|  |  | 12,385 | 16,284 | 1,743 | 120,986 | 0,722 | 79,493 | 271,741 | 32,924 |
|  |  | 4,507 | 8,246 | 0,791 | 148,114 | 0,463 | 44,512 | 152,441 | 24,533 |
|  |  | 4,459 | 8,704 | 1,139 | 149,805 | 0,576 | 48,514 | 179,836 | 22,112 |
|  | *average* | *5,812* | *8,999* | *1,032* | *136,973* | *0,506* | *50,400* | *162,372* | *22,617* |
|  | *SD* | *3,053* | *3,022* | *0,342* | *24,121* | *0,109* | *13,970* | *49,974* | *4,558* |
|  |  |  |  |  |  |  |  |  |  |
| 3% FO, saline |  | 4,947 | 8,306 | 1,163 | 107,709 | 0,258 | 29,325 | 58,566 | 40,912 |
|  |  | 7,821 | 10,502 | 1,431 | 101,203 | 0,423 | 34,453 | 54,984 | 43,679 |
|  |  | 6,054 | 7,973 | 1,063 | 126,710 | 0,416 | 22,198 | 39,058 | 40,801 |
|  |  | 8,800 | 11,411 | 1,450 | 125,377 | 0,418 | 23,675 | 42,668 | 49,060 |
|  |  | 6,435 | 9,372 | 1,226 | 126,361 | 0,376 | 40,735 | 60,614 | 31,539 |
|  |  | 15,531 | 9,618 | 1,296 | 136,869 | 0,455 | 21,627 | 23,303 | 51,817 |
|  |  | 12,145 | 10,145 | 1,303 | 149,107 | 0,409 | 39,507 | 58,603 | 30,189 |
|  |  | 9,338 | 8,169 | 1,145 | 203,841 | 0,312 | 20,500 | 26,709 | 33,551 |
|  | *average* | *8,884* | *9,437* | *1,260* | *134,647* | *0,383* | *29,002* | *45,563* | *40,194* |
|  | *SD* | *3,498* | *1,230* | *0,137* | *31,766* | *0,066* | *8,274* | *14,910* | *7,981* |
|  |  |  |  |  |  |  |  |  |  |
| 3% FO, LPS |  | 12,532 | 15,386 | 1,130 | 163,826 | 0,612 | 73,817 | 159,855 | 29,549 |
|  |  | 23,458 | 23,566 | 1,649 | 203,774 | 0,702 | 119,659 | 186,921 | 30,187 |
|  |  | 13,266 | 16,213 | 1,271 | 212,119 | 0,537 | 80,833 | 182,299 | 28,379 |
|  |  | 22,964 | 23,466 | 1,944 | 180,048 | 0,760 | 110,652 | 198,969 | 27,734 |
|  |  | 13,217 | 14,990 | 1,362 | 184,759 | 0,469 | 75,804 | 170,112 | 26,171 |
|  |  | 5,296 | 10,136 | 0,762 | 217,438 | 0,356 | 50,061 | 114,755 | 29,394 |
|  |  | 13,659 | 16,988 | 1,006 | 173,280 | 0,452 | 77,250 | 160,605 | 24,304 |
|  | *average* | *14,913* | *17,249* | *1,304* | *190,749* | *0,555* | *84,011* | *167,645* | *27,960* |
|  | *SD* | *6,366* | *4,811* | *0,397* | *20,490* | *0,144* | *23,665* | *27,329* | *2,095* |
