# Supplementary material for: Fish oil and inflammatory status alter the n-3 to n-6 balance of the endocannabinoid and oxylipin metabolomes in mouse plasma and tissues
Source: Metabolomics. 2012 Apr 11;8(6):1130–47. doi: 10.1007/s11306-012-0421-9 (PMC3483099; doi:10.1007/s11306-012-0421-9)
Supplement: Supplementary file 3 — Supplementary material 3 (DOC 84 kb) [file 11306_2012_421_MOESM3_ESM.doc]

| **Diet effect** | plasma | liver | ileum | adi. tiss. |
| --- | --- | --- | --- | --- |
| *Compound* | *p-value* | | | |
| 11-HETE | 0,0043 | <.0001 |  | 0,0311 |
| 11,12 EET | 0,0216 | 0,0052 | 0,0003 | 0,0009 |
| 11,12-DiHETrE | <.0001 | <.0001 | 0,0002 | 0,0002 |
| 12-HEPE |  | 0,0001 | <.0001 | <.0001 |
| 12-HETE | 0,0026 | 0,0001 |  | 0,0326 |
| 12-HHTrE | 0,0178 | <.0001 |  | 0,0207 |
| 13-HODE | 0,0071 | 0,0202 |  | 0,0061 |
| 13,14-dihydro-15-keto-PGD2 |  |  | <.0001 |  |
| 13,14-dihydro-15-keto-PGE2 | 0,0004 | 0,0131 | <.0001 | <.0001 |
| 13,14-dihydro-15-keto-PGF2a |  | <.0001 | 0,0002 | 0,0134 |
| 14,15 EET | 0,0006 | <.0001 | 0,017 | 0,0151 |
| 14,15-DiHETrE | <.0001 | <.0001 | <.0001 |  |
| 15-HETE | 0,0009 | <.0001 |  | 0,0003 |
| 17 keto- 4(z), 7(z), 10(z), 13 (z), 15 (E), 19(z)-DHA |  |  | 0,0019 |  |
| 17-HDoHE |  | 0,0006 | 0,0009 | 0,0045 |
| 10,17-DiHDoHE |  |  | 0,0003 | 0,0008 |
| 19,20-DiHoPE | <.0001 | 0,0002 | <.0001 | <.0001 |
| 20-HETE |  | 0,0185 |  |  |
| 2-AG | <.0001 | <.0001 | <.0001 | <.0001 |
| 5-HEPE | <.0001 | <.0001 | <.0001 | <.0001 |
| 5-HETE | 0,0003 | 0,0026 | 0,023 | 0,0138 |
| 5,6-EET |  | <.0001 |  |  |
| 5,6-DiHETrE | 0,0229 | 0,0102 | 0,0011 | 0,0038 |
| 8,9-EET |  |  | 0,0036 |  |
| 8,9-DiHETrE | 0,0004 | <.0001 | 0,0009 |  |
| 8-iso-PGF2a |  |  |  | 0,0007 |
| 9-HODE |  |  |  | 0,0123 |
| ARA |  | <.0001 |  | 0,0035 |
| DHA | <.0001 |  | <.0001 |  |
| EPA |  | <.0001 | <.0001 | <.0001 |
| Lipoxin A4 |  | 0,0035 | 0,0047 | <.0001 |
| LTB4 |  | 0,0001 | 0,0009 |  |
| LTD4 |  |  | <.0001 |  |
| n-acetyl LTE4 |  | 0,0091 |  |  |
| PGD2 | 0,0042 |  |  |  |
| PGD3 |  |  | 0,0001 | <.0001 |
| PGE2 | <.0001 | <.0001 |  |  |
| PGE3 |  |  | <.0001 | <.0001 |
| PGF2a |  | 0,001 |  |  |
| TBXB2 | 0,0157 | <.0001 |  |  |
| TBXB3 |  |  | 0,0001 | <.0001 |
| 9,10,13-trihome |  |  |  | 0,0306 |
| AEA |  | 0,0156 | 0,0007 | <.0001 |
| DHEA | <.0001 | <.0001 | <.0001 | <.0001 |
| DLE | <.0001 |  | 0,0124 | <.0001 |
| EPEA | 0,0001 |  | <.0001 | <.0001 |
| OEA | <.0001 | 0,0041 |  |  |
| SEA | 0,0007 |  |  | 0,0009 |
| UK2 | 0,0087 |  |  |  |
| UK4 | 0,0131 |  |  |  |
| UK5 | 0,0067 |  |  |  |
