# Supplementary material for: Fish oil and inflammatory status alter the n-3 to n-6 balance of the endocannabinoid and oxylipin metabolomes in mouse plasma and tissues
Source: Metabolomics. 2012 Apr 11;8(6):1130–47. doi: 10.1007/s11306-012-0421-9 (PMC3483099; doi:10.1007/s11306-012-0421-9)
Supplement: Supplementary file 4 — Supplementary material 4 (DOC 80 kb) [file 11306_2012_421_MOESM4_ESM.doc]

| **LPS effect** | plasma | liver | ileum | adi. tiss. |
| --- | --- | --- | --- | --- |
| *compound* | *p-value* | | | |
| 11-HETE | 0,0058 | 0,0002 |  | 0,0005 |
| 5,6-EET |  |  |  | 0,02 |
| 5,6-DiHETrE |  |  |  | 0,0053 |
| 12-HETE |  | 0,0116 |  |  |
| 11,12-EET |  |  |  | 0,0006 |
| 11,12-DiHETrE | <.0001 |  |  |  |
| 12-HHTrE | <.0001 |  |  | 0,0002 |
| 13,14-dihydro-15-keto-PGE2 | 0,0012 |  |  | 0,0014 |
| 13,14-dihydro-15-keto-PGF2a |  | <.0001 |  |  |
| 14,15-EET |  | 0,0177 |  |  |
| 14,15-DiHETrE | <.0001 |  |  |  |
| 15-HETE | 0,0004 |  |  |  |
| 10,17-DiHDoHE |  |  |  | 0,0088 |
| 19,20-DiHoPE | <.0001 | 0,0148 | 0,0037 | 0,0001 |
| 2-AG | 0,0021 |  |  | 0,0017 |
| 5-HEPE | 0,0077 |  |  | 0,0001 |
| 5-HETE | 0,0036 |  |  | <.0001 |
| 20-HETE |  | 0,001 |  |  |
| 8-iso-PGF2a |  | 0,0014 |  |  |
| 8,9-DiHETrE | 0,0007 |  |  |  |
| 9(S)-HODE | <.0001 |  | 0,0154 | <.0001 |
| 13-HODE |  |  | 0,0082 | 0,0016 |
| 9,10,13-TriHOME | 0,0019 |  |  |  |
| LTB4 |  | 0,0029 |  | <.0001 |
| LTD4 |  |  |  | 0,0005 |
| n-acetyl LTE4 |  | 0,0002 |  |  |
| Lipoxin A4 |  |  |  | 0,0157 |
| PGD3 |  |  |  | 0,0087 |
| PGE2 | <.0001 | 0,0004 |  | 0,0003 |
| PGE3 |  |  | 0,0007 | 0,0003 |
| PGF2a |  |  |  | 0,0056 |
| TBXB2 | <.0001 |  | 0,0046 | 0,0011 |
| TBXB3 |  |  | 0,0166 | 0,0004 |
| ARA |  | 0,0014 |  | 0,0005 |
| DHA | <.0001 | 0,0125 |  |  |
| EPA |  |  |  | 0,0111 |
| AEA |  | <.0001 | 0,0019 |  |
| DHEA | <.0001 | <.0001 | 0,0014 | <.0001 |
| DLE | <.0001 | 0,0008 | 0,0038 |  |
| EPEA | 0,0115 |  |  | 0,0014 |
| OEA | <.0001 | <.0001 | 0,0133 |  |
| PEA |  | 0,0035 | 0,0059 |  |
| SEA | <.0001 | <.0001 | 0,0011 | 0,0004 |
| UK1 | 0,0011 |  |  | 0,0153 |
| UK2 | 0,0019 |  |  |  |
| UK3 | 0,0003 | 0,0238 |  | <.0001 |
| UK4 |  |  |  | <.0001 |
| UK5 | 0,0012 |  |  | <.0001 |
