# Supplementary material for: Fish oil and inflammatory status alter the n-3 to n-6 balance of the endocannabinoid and oxylipin metabolomes in mouse plasma and tissues
Source: Metabolomics. 2012 Apr 11;8(6):1130–47. doi: 10.1007/s11306-012-0421-9 (PMC3483099; doi:10.1007/s11306-012-0421-9)
Supplement: Supplementary file 5 — Supplementary material 5 (DOC 38 kb) [file 11306_2012_421_MOESM5_ESM.doc]

|  | **compound** | **pvalue** |
| --- | --- | --- |
| **interaction effect** | L_PGD2 | 0,0002 |
|  | L_9,12,13-TriHOME | 0,0004 |
|  | L_9,10,13-TriHOME | 0,0036 |
|  | L_epea | 0,0004 |
|  | I_n-acetyl leukotriene E4 | 0,0016 |
|  | P_EPA | 0,0013 |
|  | P_ARA | 0,0029 |
|  | P_12-HEPE | 0,0042 |
|  | P_TBXB3 | 0,0034 |
|  | P_12,13-DiHOME | <.0001 |
|  | P_9,10-DiHOME | 0,0014 |
|  | P_AEA | 0,0002 |
|  | P_PEA | 0,0024 |
|  | F_LTB4 | <.0001 |
|  | F_14,15-DiHETrE | 0,0017 |
|  | F_8,9-DiHETrE | 0,004 |
|  | F_17 keto- 4(z), 7(z), 10(z), 13 (z), 15 (E), 19(z)-DHA | 0,0003 |
|  | F_12,13-DiHOME | <.0001 |
|  | F_9,10-DiHOME | 0,0004 |
|  | F_UK2 | 0,0009 |
|  | F_pea | 0,0014 |
|  | F_oea | <.0001 |
