# Supplementary material for: Fish oil and inflammatory status alter the n-3 to n-6 balance of the endocannabinoid and oxylipin metabolomes in mouse plasma and tissues
Source: Metabolomics. 2012 Apr 11;8(6):1130–47. doi: 10.1007/s11306-012-0421-9 (PMC3483099; doi:10.1007/s11306-012-0421-9)
Supplement: Supplementary file 6 — Supplementary material 6 (DOC 5337 kb) [file 11306_2012_421_MOESM6_ESM.doc]

| **Compound** | **Diet** | **LPS** | **vs** | **Diet** | **LPS** | **Estimate** | **P-value** |  | **Compound** | **Diet** | **LPS** | **vs** | **Diet** | **LPS** | **Estimate** | **P-value** |
| --- | --- | --- | --- | --- | --- | --- | --- | --- | --- | --- | --- | --- | --- | --- | --- | --- |
|  |  |  |  |  |  |  |  |  |  |  |  |  |  |  |  |  |
| P_AA | control | no |  | diet_1 | no | 843,76 | 0,0008 |  | P_AA | control | no |  | control | yes | -1060,07 | 0,0001 |
| P_AA | control | no |  | diet_3 | no | 1148,54 | <.0001 |  | P_AA | diet_1 | no |  | diet_1 | yes | -169,26 | 0,7928 |
| P_AA | diet_1 | no |  | diet_3 | no | 304,79 | 0,279 |  | P_AA | diet_3 | no |  | diet_3 | yes | -621,81 | 0,0095 |
| P_AA | control | yes |  | diet_1 | yes | 1734,56 | <.0001 |  |  |  |  |  |  |  |  |  |
| P_AA | control | yes |  | diet_3 | yes | 1586,8 | <.0001 |  |  |  |  |  |  |  |  |  |
| P_AA | diet_1 | yes |  | diet_3 | yes | -147,76 | 0,8779 |  |  |  |  |  |  |  |  |  |
|  |  |  |  |  |  |  |  |  |  |  |  |  |  |  |  |  |
|  |  |  |  |  |  |  |  |  |  |  |  |  |  |  |  |  |
| P_EPA | control | no |  | diet_1 | no | -22,625 | <.0001 |  | P_EPA | control | no |  | control | yes | -7,2321 | 0,1227 |
| P_EPA | control | no |  | diet_3 | no | -20,5 | <.0001 |  | P_EPA | diet_1 | no |  | diet_1 | yes | -2,875 | 0,8915 |
| P_EPA | diet_1 | no |  | diet_3 | no | 2,125 | 0,9679 |  | P_EPA | diet_3 | no |  | diet_3 | yes | -17,875 | <.0001 |
| P_EPA | control | yes |  | diet_1 | yes | -18,2679 | <.0001 |  |  |  |  |  |  |  |  |  |
| P_EPA | control | yes |  | diet_3 | yes | -31,1429 | <.0001 |  |  |  |  |  |  |  |  |  |
| P_EPA | diet_1 | yes |  | diet_3 | yes | -12,875 | 0,0005 |  |  |  |  |  |  |  |  |  |
|  |  |  |  |  |  |  |  |  |  |  |  |  |  |  |  |  |
|  |  |  |  |  |  |  |  |  |  |  |  |  |  |  |  |  |
| P_DHA | control | no |  | diet_1 | no | -2548,58 | 0,0003 |  | P_DHA | control | no |  | control | yes | -2162,41 | 0,004 |
| P_DHA | control | no |  | diet_3 | no | -934,09 | 0,5014 |  | P_DHA | diet_1 | no |  | diet_1 | yes | -2614,86 | 0,0002 |
| P_DHA | diet_1 | no |  | diet_3 | no | 1614,49 | 0,0442 |  | P_DHA | diet_3 | no |  | diet_3 | yes | -4436,9 | <.0001 |
| P_DHA | control | yes |  | diet_1 | yes | -3001,02 | <.0001 |  |  |  |  |  |  |  |  |  |
| P_DHA | control | yes |  | diet_3 | yes | -3208,58 | <.0001 |  |  |  |  |  |  |  |  |  |
| P_DHA | diet_1 | yes |  | diet_3 | yes | -207,56 | 0,9989 |  |  |  |  |  |  |  |  |  |
|  |  |  |  |  |  |  |  |  |  |  |  |  |  |  |  |  |
|  |  |  |  |  |  |  |  |  |  |  |  |  |  |  |  |  |
| P_12(S)-HHTrE | control | no |  | diet_1 | no | 0,09019 | 1 |  | P_12(S)-HHTrE | control | no |  | control | yes | 4,052 | 0,0125 |
| P_12(S)-HHTrE | control | no |  | diet_3 | no | 3,2202 | 0,061 |  | P_12(S)-HHTrE | diet_1 | no |  | diet_1 | yes | 4,9024 | 0,001 |
| P_12(S)-HHTrE | diet_1 | no |  | diet_3 | no | 3,13 | 0,0736 |  | P_12(S)-HHTrE | diet_3 | no |  | diet_3 | yes | 2,2165 | 0,3973 |
| P_12(S)-HHTrE | control | yes |  | diet_1 | yes | 0,9405 | 0,962 |  |  |  |  |  |  |  |  |  |
| P_12(S)-HHTrE | control | yes |  | diet_3 | yes | 1,3846 | 0,8483 |  |  |  |  |  |  |  |  |  |
| P_12(S)-HHTrE | diet_1 | yes |  | diet_3 | yes | 0,4441 | 0,9988 |  |  |  |  |  |  |  |  |  |

| **Compound** | **Diet** | **LPS** | **vs** | **Diet** | **LPS** | **Estimate** | **P-value** |  | **Compound** | **Diet** | **LPS** | **vs** | **Diet** | **LPS** | **Estimate** | **P-value** |
| --- | --- | --- | --- | --- | --- | --- | --- | --- | --- | --- | --- | --- | --- | --- | --- | --- |
| P_9(S)-HODE | control | no |  | diet_1 | no | 5,5 | 0,8803 |  | P_9(S)-HODE | control | no |  | control | yes | -6,3929 | 0,8196 |
| P_9(S)-HODE | control | no |  | diet_3 | no | 17,5 | 0,0143 |  | P_9(S)-HODE | diet_1 | no |  | diet_1 | yes | -13,25 | 0,1114 |
| P_9(S)-HODE | diet_1 | no |  | diet_3 | no | 12 | 0,1843 |  | P_9(S)-HODE | diet_3 | no |  | diet_3 | yes | -26,8929 | <.0001 |
| P_9(S)-HODE | control | yes |  | diet_1 | yes | -1,3571 | 0,9998 |  |  |  |  |  |  |  |  |  |
| P_9(S)-HODE | control | yes |  | diet_3 | yes | -3 | 0,9931 |  |  |  |  |  |  |  |  |  |
| P_9(S)-HODE | diet_1 | yes |  | diet_3 | yes | -1,6429 | 0,9995 |  |  |  |  |  |  |  |  |  |
|  |  |  |  |  |  |  |  |  |  |  |  |  |  |  |  |  |
|  |  |  |  |  |  |  |  |  |  |  |  |  |  |  |  |  |
| P_13(S)-HODE | control | no |  | diet_1 | no | 1,625 | 0,9997 |  | P_13(S)-HODE | control | no |  | control | yes | 1,4286 | 0,9999 |
| P_13(S)-HODE | control | no |  | diet_3 | no | 22,125 | 0,004 |  | P_13(S)-HODE | diet_1 | no |  | diet_1 | yes | -2,875 | 0,9954 |
| P_13(S)-HODE | diet_1 | no |  | diet_3 | no | 20,5 | 0,009 |  | P_13(S)-HODE | diet_3 | no |  | diet_3 | yes | -19,6964 | 0,0183 |
| P_13(S)-HODE | control | yes |  | diet_1 | yes | -2,6786 | 0,9972 |  |  |  |  |  |  |  |  |  |
| P_13(S)-HODE | control | yes |  | diet_3 | yes | 1 | 1 |  |  |  |  |  |  |  |  |  |
| P_13(S)-HODE | diet_1 | yes |  | diet_3 | yes | 3,6786 | 0,9878 |  |  |  |  |  |  |  |  |  |
|  |  |  |  |  |  |  |  |  |  |  |  |  |  |  |  |  |
|  |  |  |  |  |  |  |  |  |  |  |  |  |  |  |  |  |
| P_12(S)-HEPE | control | no |  | diet_1 | no | -28,8125 | <.0001 |  | P_12(S)-HEPE | control | no |  | control | yes | -3,7589 | 0,9147 |
| P_12(S)-HEPE | control | no |  | diet_3 | no | -22,6875 | <.0001 |  | P_12(S)-HEPE | diet_1 | no |  | diet_1 | yes | 10,5 | 0,0627 |
| P_12(S)-HEPE | diet_1 | no |  | diet_3 | no | 6,125 | 0,5466 |  | P_12(S)-HEPE | diet_3 | no |  | diet_3 | yes | -6,7143 | 0,484 |
| P_12(S)-HEPE | control | yes |  | diet_1 | yes | -14,5536 | 0,0048 |  |  |  |  |  |  |  |  |  |
| P_12(S)-HEPE | control | yes |  | diet_3 | yes | -25,6429 | <.0001 |  |  |  |  |  |  |  |  |  |
| P_12(S)-HEPE | diet_1 | yes |  | diet_3 | yes | -11,0893 | 0,0547 |  |  |  |  |  |  |  |  |  |
|  |  |  |  |  |  |  |  |  |  |  |  |  |  |  |  |  |
|  |  |  |  |  |  |  |  |  |  |  |  |  |  |  |  |  |
| P_5(S)-HEPE | control | no |  | diet_1 | no | -18,75 | <.0001 |  | P_5(S)-HEPE | control | no |  | control | yes | -3,55E-15 | 1 |
| P_5(S)-HEPE | control | no |  | diet_3 | no | -18,125 | <.0001 |  | P_5(S)-HEPE | diet_1 | no |  | diet_1 | yes | -2,1875 | 0,9856 |
| P_5(S)-HEPE | diet_1 | no |  | diet_3 | no | 0,625 | 1 |  | P_5(S)-HEPE | diet_3 | no |  | diet_3 | yes | -14,375 | 0,0021 |
| P_5(S)-HEPE | control | yes |  | diet_1 | yes | -20,9375 | <.0001 |  |  |  |  |  |  |  |  |  |
| P_5(S)-HEPE | control | yes |  | diet_3 | yes | -32,5 | <.0001 |  |  |  |  |  |  |  |  |  |
| P_5(S)-HEPE | diet_1 | yes |  | diet_3 | yes | -11,5625 | 0,0202 |  |  |  |  |  |  |  |  |  |

| **Compound** | **Diet** | **LPS** | | **vs** | | **Diet** | | **LPS** | | **Estimate** | | **P-value** | |  | | **Compound** | | **Diet** | | **LPS** | | **vs** | | **Diet** | | **LPS** | | **Estimate** | | **P-value** | |
| --- | --- | --- | --- | --- | --- | --- | --- | --- | --- | --- | --- | --- | --- | --- | --- | --- | --- | --- | --- | --- | --- | --- | --- | --- | --- | --- | --- | --- | --- | --- | --- |
| P_15(S)-HETE | control | no |  | | diet_1 | | no | | 0,02556 | | 0,9978 | |  | | P_15(S)-HETE | | control | | no | |  | | control | | yes | | 0,1192 | | 0,3695 | |  |
| P_15(S)-HETE | control | no |  | | diet_3 | | no | | 0,1974 | | 0,0179 | |  | | P_15(S)-HETE | | diet_1 | | no | |  | | diet_1 | | yes | | 0,2074 | | 0,0113 | |  |
| P_15(S)-HETE | diet_1 | no |  | | diet_3 | | no | | 0,1719 | | 0,0541 | |  | | P_15(S)-HETE | | diet_3 | | no | |  | | diet_3 | | yes | | 0,07094 | | 0,8437 | |  |
| P_15(S)-HETE | control | yes |  | | diet_1 | | yes | | 0,1138 | | 0,4215 | |  | |  | |  | |  | |  | |  | |  | |  | |  | |  |
| P_15(S)-HETE | control | yes |  | | diet_3 | | yes | | 0,1491 | | 0,18 | |  | |  | |  | |  | |  | |  | |  | |  | |  | |  |
| P_15(S)-HETE | diet_1 | yes |  | | diet_3 | | yes | | 0,03537 | | 0,9913 | |  | |  | |  | |  | |  | |  | |  | |  | |  | |  |
|  |  |  |  | |  | |  | |  | |  | |  | |  | |  | |  | |  | |  | |  | |  | |  | |  |
|  |  |  |  | |  | |  | |  | |  | |  | |  | |  | |  | |  | |  | |  | |  | |  | |  |
| P_11(S)-HETE | control | no |  | | diet_1 | | no | | 0,02669 | | 0,9992 | |  | | P_11(S)-HETE | | control | | no | |  | | control | | yes | | 0,131 | | 0,541 | |  |
| P_11(S)-HETE | control | no |  | | diet_3 | | no | | 0,2186 | | 0,0576 | |  | | P_11(S)-HETE | | diet_1 | | no | |  | | diet_1 | | yes | | 0,1861 | | 0,1493 | |  |
| P_11(S)-HETE | diet_1 | no |  | | diet_3 | | no | | 0,1919 | | 0,1275 | |  | | P_11(S)-HETE | | diet_3 | | no | |  | | diet_3 | | yes | | 0,06838 | | 0,9476 | |  |
| P_11(S)-HETE | control | yes |  | | diet_1 | | yes | | 0,08179 | | 0,894 | |  | |  | |  | |  | |  | |  | |  | |  | |  | |  |
| P_11(S)-HETE | control | yes |  | | diet_3 | | yes | | 0,1559 | | 0,3843 | |  | |  | |  | |  | |  | |  | |  | |  | |  | |  |
| P_11(S)-HETE | diet_1 | yes |  | | diet_3 | | yes | | 0,07413 | | 0,9275 | |  | |  | |  | |  | |  | |  | |  | |  | |  | |  |
|  |  |  |  | |  | |  | |  | |  | |  | |  | |  | |  | |  | |  | |  | |  | |  | |  |
|  |  |  |  | |  | |  | |  | |  | |  | |  | |  | |  | |  | |  | |  | |  | |  | |  |
| P_12(S)-HETE | control | no |  | | diet_1 | | no | | 8,7929 | | 0,8011 | |  | | P_12(S)-HETE | | control | | no | |  | | control | | yes | | 9,8037 | | 0,7476 | |  |
| P_12(S)-HETE | control | no |  | | diet_3 | | no | | 21,9469 | | 0,0331 | |  | | P_12(S)-HETE | | diet_1 | | no | |  | | diet_1 | | yes | | 15,3429 | | 0,256 | |  |
| P_12(S)-HETE | diet_1 | no |  | | diet_3 | | no | | 13,154 | | 0,4203 | |  | | P_12(S)-HETE | | diet_3 | | no | |  | | diet_3 | | yes | | 3,3677 | | 0,997 | |  |
| P_12(S)-HETE | control | yes |  | | diet_1 | | yes | | 14,3321 | | 0,3635 | |  | |  | |  | |  | |  | |  | |  | |  | |  | |  |
| P_12(S)-HETE | control | yes |  | | diet_3 | | yes | | 15,5109 | | 0,3131 | |  | |  | |  | |  | |  | |  | |  | |  | |  | |  |
| P_12(S)-HETE | diet_1 | yes |  | | diet_3 | | yes | | 1,1787 | | 1 | |  | |  | |  | |  | |  | |  | |  | |  | |  | |  |
|  |  |  |  | |  | |  | |  | |  | |  | |  | |  | |  | |  | |  | |  | |  | |  | |  |
|  |  |  |  | |  | |  | |  | |  | |  | |  | |  | |  | |  | |  | |  | |  | |  | |  |
| P_11,12 EET | control | no |  | | diet_1 | | no | | 0,03246 | | 0,7523 | |  | | P_11,12 EET | | control | | no | |  | | control | | yes | | -0,02825 | | 0,8432 | |  |
| P_11,12 EET | control | no |  | | diet_3 | | no | | 0,04076 | | 0,5573 | |  | | P_11,12 EET | | diet_1 | | no | |  | | diet_1 | | yes | | -0,0053 | | 0,9999 | |  |
| P_11,12 EET | diet_1 | no |  | | diet_3 | | no | | 0,008303 | | 0,9992 | |  | | P_11,12 EET | | diet_3 | | no | |  | | diet_3 | | yes | | -0,0051 | | 0,9999 | |  |
| P_11,12 EET | control | yes |  | | diet_1 | | yes | | 0,0554 | | 0,2699 | |  | |  | |  | |  | |  | |  | |  | |  | |  | |  |
| P_11,12 EET | control | yes |  | | diet_3 | | yes | | 0,06391 | | 0,1661 | |  | |  | |  | |  | |  | |  | |  | |  | |  | |  |
| P_11,12 EET | diet_1 | yes |  | | diet_3 | | yes | | 0,008505 | | 0,9991 | |  | |  | |  | |  | |  | |  | |  | |  | |  | |  |

| Compound | Diet | LPS | vs | Diet | LPS | Estimate | P-value |  | Compound | Diet | LPS | vs | Diet | LPS | Estimate | P-value |
| --- | --- | --- | --- | --- | --- | --- | --- | --- | --- | --- | --- | --- | --- | --- | --- | --- |
| P_5,6 EET | control | no |  | diet_1 | no | 10,9209 | 0,5966 |  | P_5,6 EET | control | no |  | control | yes | -4,7594 | 0,9773 |
| P_5,6 EET | control | no |  | diet_3 | no | 10,9209 | 0,5966 |  | P_5,6 EET | diet_1 | no |  | diet_1 | yes | 8,88E-16 | 1 |
| P_5,6 EET | diet_1 | no |  | diet_3 | no | -4,00E-14 | 1 |  | P_5,6 EET | diet_3 | no |  | diet_3 | yes | -6,9916 | 0,8956 |
| P_5,6 EET | control | yes |  | diet_1 | yes | 15,6804 | 0,2591 |  |  |  |  |  |  |  |  |  |
| P_5,6 EET | control | yes |  | diet_3 | yes | 8,6887 | 0,7887 |  |  |  |  |  |  |  |  |  |
| P_5,6 EET | diet_1 | yes |  | diet_3 | yes | -6,9916 | 0,8956 |  |  |  |  |  |  |  |  |  |
|  |  |  |  |  |  |  |  |  |  |  |  |  |  |  |  |  |
|  |  |  |  |  |  |  |  |  |  |  |  |  |  |  |  |  |
| P_5(S)-HETE | control | no |  | diet_1 | no | 16,25 | 0,028 |  | P_5(S)-HETE | control | no |  | control | yes | -6,1607 | 0,8426 |
| P_5(S)-HETE | control | no |  | diet_3 | no | 16,5 | 0,0246 |  | P_5(S)-HETE | diet_1 | no |  | diet_1 | yes | -9,75 | 0,3959 |
| P_5(S)-HETE | diet_1 | no |  | diet_3 | no | 0,25 | 1 |  | P_5(S)-HETE | diet_3 | no |  | diet_3 | yes | -11,6607 | 0,2439 |
| P_5(S)-HETE | control | yes |  | diet_1 | yes | 12,6607 | 0,1707 |  |  |  |  |  |  |  |  |  |
| P_5(S)-HETE | control | yes |  | diet_3 | yes | 11 | 0,3369 |  |  |  |  |  |  |  |  |  |
| P_5(S)-HETE | diet_1 | yes |  | diet_3 | yes | -1,6607 | 0,9995 |  |  |  |  |  |  |  |  |  |
|  |  |  |  |  |  |  |  |  |  |  |  |  |  |  |  |  |
|  |  |  |  |  |  |  |  |  |  |  |  |  |  |  |  |  |
| P_14,15 EET | control | no |  | diet_1 | no | 8,8125 | 0,1356 |  | P_14,15 EET | control | no |  | control | yes | -0,8304 | 0,9999 |
| P_14,15 EET | control | no |  | diet_3 | no | 8,8125 | 0,1356 |  | P_14,15 EET | diet_1 | no |  | diet_1 | yes | -6,64E-17 | 1 |
| P_14,15 EET | diet_1 | no |  | diet_3 | no | 1,89E-15 | 1 |  | P_14,15 EET | diet_3 | no |  | diet_3 | yes | -1,09E-14 | 1 |
| P_14,15 EET | control | yes |  | diet_1 | yes | 9,6429 | 0,0998 |  |  |  |  |  |  |  |  |  |
| P_14,15 EET | control | yes |  | diet_3 | yes | 9,6429 | 0,1198 |  |  |  |  |  |  |  |  |  |
| P_14,15 EET | diet_1 | yes |  | diet_3 | yes | -8,90E-15 | 1 |  |  |  |  |  |  |  |  |  |
|  |  |  |  |  |  |  |  |  |  |  |  |  |  |  |  |  |
|  |  |  |  |  |  |  |  |  |  |  |  |  |  |  |  |  |
| P_PGB2 | control | no |  | diet_1 | no | 2,629 | 0,9996 |  | P_PGB2 | control | no |  | control | yes | 10,1907 | 0,8541 |
| P_PGB2 | control | no |  | diet_3 | no | 12,097 | 0,7452 |  | P_PGB2 | diet_1 | no |  | diet_1 | yes | -0,2281 | 1 |
| P_PGB2 | diet_1 | no |  | diet_3 | no | 9,468 | 0,8841 |  | P_PGB2 | diet_3 | no |  | diet_3 | yes | 8,1562 | 0,935 |
| P_PGB2 | control | yes |  | diet_1 | yes | -7,7899 | 0,9457 |  |  |  |  |  |  |  |  |  |
| P_PGB2 | control | yes |  | diet_3 | yes | 10,0624 | 0,8634 |  |  |  |  |  |  |  |  |  |
| P_PGB2 | diet_1 | yes |  | diet_3 | yes | 17,8523 | 0,3964 |  |  |  |  |  |  |  |  |  |

| Compound | Diet | LPS | vs | Diet | LPS | Estimate | P-value |  | Compound | Diet | LPS | vs | Diet | LPS | Estimate | P-value |
| --- | --- | --- | --- | --- | --- | --- | --- | --- | --- | --- | --- | --- | --- | --- | --- | --- |
| P_14,15-DiHETrE | control | no |  | diet_1 | no | 19,25 | 0,0001 |  | P_14,15-DiHETrE | control | no |  | control | yes | -10,375 | 0,1024 |
| P_14,15-DiHETrE | control | no |  | diet_3 | no | 21,5 | <.0001 |  | P_14,15-DiHETrE | diet_1 | no |  | diet_1 | yes | -5,8125 | 0,6331 |
| P_14,15-DiHETrE | diet_1 | no |  | diet_3 | no | 2,25 | 0,9904 |  | P_14,15-DiHETrE | diet_3 | no |  | diet_3 | yes | -19,6607 | 0,0001 |
| P_14,15-DiHETrE | control | yes |  | diet_1 | yes | 23,8125 | <.0001 |  |  |  |  |  |  |  |  |  |
| P_14,15-DiHETrE | control | yes |  | diet_3 | yes | 12,2143 | 0,0432 |  |  |  |  |  |  |  |  |  |
| P_14,15-DiHETrE | diet_1 | yes |  | diet_3 | yes | -11,5982 | 0,0498 |  |  |  |  |  |  |  |  |  |
|  |  |  |  |  |  |  |  |  |  |  |  |  |  |  |  |  |
|  |  |  |  |  |  |  |  |  |  |  |  |  |  |  |  |  |
| P_11,12-DiHETrE | control | no |  | diet_1 | no | 19,25 | <.0001 |  | P_11,12-DiHETrE | control | no |  | control | yes | -8,9286 | 0,1586 |
| P_11,12-DiHETrE | control | no |  | diet_3 | no | 25,5 | <.0001 |  | P_11,12-DiHETrE | diet_1 | no |  | diet_1 | yes | -6,0625 | 0,5186 |
| P_11,12-DiHETrE | diet_1 | no |  | diet_3 | no | 6,25 | 0,4851 |  | P_11,12-DiHETrE | diet_3 | no |  | diet_3 | yes | -20,3571 | <.0001 |
| P_11,12-DiHETrE | control | yes |  | diet_1 | yes | 22,1161 | <.0001 |  |  |  |  |  |  |  |  |  |
| P_11,12-DiHETrE | control | yes |  | diet_3 | yes | 14,0714 | 0,0066 |  |  |  |  |  |  |  |  |  |
| P_11,12-DiHETrE | diet_1 | yes |  | diet_3 | yes | -8,0446 | 0,2504 |  |  |  |  |  |  |  |  |  |
|  |  |  |  |  |  |  |  |  |  |  |  |  |  |  |  |  |
|  |  |  |  |  |  |  |  |  |  |  |  |  |  |  |  |  |
| P_5,6-DiHETrE | control | no |  | diet_1 | no | 0,001077 | 1 |  | P_5,6-DiHETrE | control | no |  | control | yes | -0,05305 | 0,0464 |
| P_5,6-DiHETrE | control | no |  | diet_3 | no | 0,008841 | 0,9909 |  | P_5,6-DiHETrE | diet_1 | no |  | diet_1 | yes | 0,001703 | 1 |
| P_5,6-DiHETrE | diet_1 | no |  | diet_3 | no | 0,007764 | 0,995 |  | P_5,6-DiHETrE | diet_3 | no |  | diet_3 | yes | -0,00486 | 0,9995 |
| P_5,6-DiHETrE | control | yes |  | diet_1 | yes | 0,05583 | 0,0346 |  |  |  |  |  |  |  |  |  |
| P_5,6-DiHETrE | control | yes |  | diet_3 | yes | 0,05703 | 0,0315 |  |  |  |  |  |  |  |  |  |
| P_5,6-DiHETrE | diet_1 | yes |  | diet_3 | yes | 0,001202 | 1 |  |  |  |  |  |  |  |  |  |
|  |  |  |  |  |  |  |  |  |  |  |  |  |  |  |  |  |
|  |  |  |  |  |  |  |  |  |  |  |  |  |  |  |  |  |
| P_17(S)-HDoHE | control | no |  | diet_1 | no | -5,2785 | 0,9016 |  | P_17(S)-HDoHE | control | no |  | control | yes | 0 | 1 |
| P_17(S)-HDoHE | control | no |  | diet_3 | no | -4,44E-15 | 1 |  | P_17(S)-HDoHE | diet_1 | no |  | diet_1 | yes | -6,1183 | 0,8357 |
| P_17(S)-HDoHE | diet_1 | no |  | diet_3 | no | 5,2785 | 0,9016 |  | P_17(S)-HDoHE | diet_3 | no |  | diet_3 | yes | -3,3492 | 0,9859 |
| P_17(S)-HDoHE | control | yes |  | diet_1 | yes | -11,3968 | 0,3209 |  |  |  |  |  |  |  |  |  |
| P_17(S)-HDoHE | control | yes |  | diet_3 | yes | -3,3492 | 0,9871 |  |  |  |  |  |  |  |  |  |
| P_17(S)-HDoHE | diet_1 | yes |  | diet_3 | yes | 8,0476 | 0,6558 |  |  |  |  |  |  |  |  |  |

| Compound | Diet | LPS | vs | Diet | LPS | Estimate | P-value |  | Compound | Diet | LPS | vs | Diet | LPS | Estimate | P-value |
| --- | --- | --- | --- | --- | --- | --- | --- | --- | --- | --- | --- | --- | --- | --- | --- | --- |
| P_PGE3 | control | no |  | diet_1 | no | -6,6709 | 0,8739 |  | P_PGE3 | control | no |  | control | yes | 7,03E-13 | 1 |
| P_PGE3 | control | no |  | diet_3 | no | -16,3438 | 0,1497 |  | P_PGE3 | diet_1 | no |  | diet_1 | yes | 6,6709 | 0,8739 |
| P_PGE3 | diet_1 | no |  | diet_3 | no | -9,6729 | 0,6197 |  | P_PGE3 | diet_3 | no |  | diet_3 | yes | 16,3438 | 0,1498 |
| P_PGE3 | control | yes |  | diet_1 | yes | -1,41E-12 | 1 |  |  |  |  |  |  |  |  |  |
| P_PGE3 | control | yes |  | diet_3 | yes | 3,60E-12 | 1 |  |  |  |  |  |  |  |  |  |
| P_PGE3 | diet_1 | yes |  | diet_3 | yes | 5,00E-12 | 1 |  |  |  |  |  |  |  |  |  |
|  |  |  |  |  |  |  |  |  |  |  |  |  |  |  |  |  |
| P_13,14-dihydro-15-keto-PGE2 | control | no |  | diet_1 | no | 9 | 0,5393 |  | P_13,14-dihydro-15-keto-PGE2 | control | no |  | control | yes | -11,1429 | 0,3418 |
| P_13,14-dihydro-15-keto-PGE2 | control | no |  | diet_3 | no | 18,375 | 0,0148 |  | P_13,14-dihydro-15-keto-PGE2 | diet_1 | no |  | diet_1 | yes | -6,9375 | 0,7772 |
| P_13,14-dihydro-15-keto-PGE2 | diet_1 | no |  | diet_3 | no | 9,375 | 0,4948 |  | P_13,14-dihydro-15-keto-PGE2 | diet_3 | no |  | diet_3 | yes | -14,5893 | 0,1056 |
| P_13,14-dihydro-15-keto-PGE2 | control | yes |  | diet_1 | yes | 13,2054 | 0,1769 |  |  |  |  |  |  |  |  |  |
| P_13,14-dihydro-15-keto-PGE2 | control | yes |  | diet_3 | yes | 14,9286 | 0,1113 |  |  |  |  |  |  |  |  |  |
| P_13,14-dihydro-15-keto-PGE2 | diet_1 | yes |  | diet_3 | yes | 1,7232 | 0,9996 |  |  |  |  |  |  |  |  |  |
|  |  |  |  |  |  |  |  |  |  |  |  |  |  |  |  |  |
| P_8-iso-PGF2a | control | no |  | diet_1 | no | -0,00806 | 0,3274 |  | P_8-iso-PGF2a | control | no |  | control | yes | -0,00394 | 0,9238 |
| P_8-iso-PGF2a | control | no |  | diet_3 | no | -0,00718 | 0,4561 |  | P_8-iso-PGF2a | diet_1 | no |  | diet_1 | yes | 0,00564 | 0,7017 |
| P_8-iso-PGF2a | diet_1 | no |  | diet_3 | no | 0,000889 | 0,9999 |  | P_8-iso-PGF2a | diet_3 | no |  | diet_3 | yes | 0,006192 | 0,6479 |
| P_8-iso-PGF2a | control | yes |  | diet_1 | yes | 0,001514 | 0,999 |  |  |  |  |  |  |  |  |  |
| P_8-iso-PGF2a | control | yes |  | diet_3 | yes | 0,002955 | 0,98 |  |  |  |  |  |  |  |  |  |
| P_8-iso-PGF2a | diet_1 | yes |  | diet_3 | yes | 0,001441 | 0,9992 |  |  |  |  |  |  |  |  |  |
|  |  |  |  |  |  |  |  |  |  |  |  |  |  |  |  |  |
| P_PGF2a | control | no |  | diet_1 | no | 0,03687 | 0,9534 |  | P_PGF2a | control | no |  | control | yes | 0,02399 | 0,994 |
| P_PGF2a | control | no |  | diet_3 | no | -0,00591 | 1 |  | P_PGF2a | diet_1 | no |  | diet_1 | yes | 0,04997 | 0,8492 |
| P_PGF2a | diet_1 | no |  | diet_3 | no | -0,04278 | 0,9153 |  | P_PGF2a | diet_3 | no |  | diet_3 | yes | 0,07934 | 0,4824 |
| P_PGF2a | control | yes |  | diet_1 | yes | 0,06284 | 0,7155 |  |  |  |  |  |  |  |  |  |
| P_PGF2a | control | yes |  | diet_3 | yes | 0,04944 | 0,886 |  |  |  |  |  |  |  |  |  |
| P_PGF2a | diet_1 | yes |  | diet_3 | yes | -0,0134 | 0,9996 |  |  |  |  |  |  |  |  |  |

| Compound | Diet | LPS | vs | Diet | LPS | Estimate | P-value |  | Compound | Diet | LPS | vs | Diet | LPS | Estimate | P-value |
| --- | --- | --- | --- | --- | --- | --- | --- | --- | --- | --- | --- | --- | --- | --- | --- | --- |
| P_19,20-DiHoPE | control | no |  | diet_1 | no | -17,5 | <.0001 |  | P_19,20-DiHoPE | control | no |  | control | yes | -13,9464 | 0,0027 |
| P_19,20-DiHoPE | control | no |  | diet_3 | no | -16,75 | 0,0001 |  | P_19,20-DiHoPE | diet_1 | no |  | diet_1 | yes | -11,75 | 0,0116 |
| P_19,20-DiHoPE | diet_1 | no |  | diet_3 | no | 0,75 | 0,9999 |  | P_19,20-DiHoPE | diet_3 | no |  | diet_3 | yes | -20,7679 | <.0001 |
| P_19,20-DiHoPE | control | yes |  | diet_1 | yes | -15,3036 | 0,0008 |  |  |  |  |  |  |  |  |  |
| P_19,20-DiHoPE | control | yes |  | diet_3 | yes | -23,5714 | <.0001 |  |  |  |  |  |  |  |  |  |
| P_19,20-DiHoPE | diet_1 | yes |  | diet_3 | yes | -8,2679 | 0,1736 |  |  |  |  |  |  |  |  |  |
|  |  |  |  |  |  |  |  |  |  |  |  |  |  |  |  |  |
|  |  |  |  |  |  |  |  |  |  |  |  |  |  |  |  |  |
| P_TBXB3 | control | no |  | diet_1 | no | -24,125 | 0,0002 |  | P_TBXB3 | control | no |  | control | yes | -1,2679 | 0,9999 |
| P_TBXB3 | control | no |  | diet_3 | no | -16,6875 | 0,0177 |  | P_TBXB3 | diet_1 | no |  | diet_1 | yes | 24,1875 | 0,0002 |
| P_TBXB3 | diet_1 | no |  | diet_3 | no | 7,4375 | 0,6557 |  | P_TBXB3 | diet_3 | no |  | diet_3 | yes | 14,4196 | 0,0712 |
| P_TBXB3 | control | yes |  | diet_1 | yes | 1,3304 | 0,9998 |  |  |  |  |  |  |  |  |  |
| P_TBXB3 | control | yes |  | diet_3 | yes | -1 | 1 |  |  |  |  |  |  |  |  |  |
| P_TBXB3 | diet_1 | yes |  | diet_3 | yes | -2,3304 | 0,9973 |  |  |  |  |  |  |  |  |  |
|  |  |  |  |  |  |  |  |  |  |  |  |  |  |  |  |  |
|  |  |  |  |  |  |  |  |  |  |  |  |  |  |  |  |  |
| P_TBXB2 | control | no |  | diet_1 | no | -0,3031 | 0,9998 |  | P_TBXB2 | control | no |  | control | yes | 3,8747 | 0,0157 |
| P_TBXB2 | control | no |  | diet_3 | no | 3,0883 | 0,0705 |  | P_TBXB2 | diet_1 | no |  | diet_1 | yes | 5,1232 | 0,0004 |
| P_TBXB2 | diet_1 | no |  | diet_3 | no | 3,3914 | 0,0362 |  | P_TBXB2 | diet_3 | no |  | diet_3 | yes | 2,1477 | 0,4101 |
| P_TBXB2 | control | yes |  | diet_1 | yes | 0,9454 | 0,9577 |  |  |  |  |  |  |  |  |  |
| P_TBXB2 | control | yes |  | diet_3 | yes | 1,3613 | 0,8467 |  |  |  |  |  |  |  |  |  |
| P_TBXB2 | diet_1 | yes |  | diet_3 | yes | 0,4159 | 0,999 |  |  |  |  |  |  |  |  |  |
|  |  |  |  |  |  |  |  |  |  |  |  |  |  |  |  |  |
|  |  |  |  |  |  |  |  |  |  |  |  |  |  |  |  |  |
| P_8,9-DiHETrE | control | no |  | diet_1 | no | 19,8988 | 0,012 |  | P_8,9-DiHETrE | control | no |  | control | yes | -9,5689 | 0,3863 |
| P_8,9-DiHETrE | control | no |  | diet_3 | no | 18,9718 | 0,0163 |  | P_8,9-DiHETrE | diet_1 | no |  | diet_1 | yes | -11,7584 | 0,1863 |
| P_8,9-DiHETrE | diet_1 | no |  | diet_3 | no | -0,927 | 0,9999 |  | P_8,9-DiHETrE | diet_3 | no |  | diet_3 | yes | -16,8509 | 0,0402 |
| P_8,9-DiHETrE | control | yes |  | diet_1 | yes | 17,7093 | 0,0302 |  |  |  |  |  |  |  |  |  |
| P_8,9-DiHETrE | control | yes |  | diet_3 | yes | 11,6899 | 0,2348 |  |  |  |  |  |  |  |  |  |
| P_8,9-DiHETrE | diet_1 | yes |  | diet_3 | yes | -6,0195 | 0,7903 |  |  |  |  |  |  |  |  |  |

| Compound | Diet | LPS | vs | Diet | LPS | Estimate | P-value |  | Compound | Diet | LPS | vs | Diet | LPS | Estimate | P-value |
| --- | --- | --- | --- | --- | --- | --- | --- | --- | --- | --- | --- | --- | --- | --- | --- | --- |
| P_PGE2 | control | no |  | diet_1 | no | 6,0625 | 0,783 |  | P_PGE2 | control | no |  | control | yes | -12,5625 | 0,12 |
| P_PGE2 | control | no |  | diet_3 | no | 19,625 | 0,0018 |  | P_PGE2 | diet_1 | no |  | diet_1 | yes | -11,375 | 0,1672 |
| P_PGE2 | diet_1 | no |  | diet_3 | no | 13,5625 | 0,0608 |  | P_PGE2 | diet_3 | no |  | diet_3 | yes | -14,3304 | 0,0528 |
| P_PGE2 | control | yes |  | diet_1 | yes | 7,25 | 0,6647 |  |  |  |  |  |  |  |  |  |
| P_PGE2 | control | yes |  | diet_3 | yes | 17,8571 | 0,011 |  |  |  |  |  |  |  |  |  |
| P_PGE2 | diet_1 | yes |  | diet_3 | yes | 10,6071 | 0,2612 |  |  |  |  |  |  |  |  |  |
|  |  |  |  |  |  |  |  |  |  |  |  |  |  |  |  |  |
|  |  |  |  |  |  |  |  |  |  |  |  |  |  |  |  |  |
| P_PGD2 | control | no |  | diet_1 | no | 0,0181 | 0,9981 |  | P_PGD2 | control | no |  | control | yes | -0,01273 | 0,9997 |
| P_PGD2 | control | no |  | diet_3 | no | 0,1204 | 0,134 |  | P_PGD2 | diet_1 | no |  | diet_1 | yes | 0,08465 | 0,4333 |
| P_PGD2 | diet_1 | no |  | diet_3 | no | 0,1023 | 0,2529 |  | P_PGD2 | diet_3 | no |  | diet_3 | yes | 0,0125 | 0,9997 |
| P_PGD2 | control | yes |  | diet_1 | yes | 0,1155 | 0,1829 |  |  |  |  |  |  |  |  |  |
| P_PGD2 | control | yes |  | diet_3 | yes | 0,1457 | 0,072 |  |  |  |  |  |  |  |  |  |
| P_PGD2 | diet_1 | yes |  | diet_3 | yes | 0,03019 | 0,9832 |  |  |  |  |  |  |  |  |  |
|  |  |  |  |  |  |  |  |  |  |  |  |  |  |  |  |  |
|  |  |  |  |  |  |  |  |  |  |  |  |  |  |  |  |  |
| P_12,13-DiHOME | control | no |  | diet_1 | no | 15,875 | 0,0074 |  | P_12,13-DiHOME | control | no |  | control | yes | -0,8214 | 1 |
| P_12,13-DiHOME | control | no |  | diet_3 | no | 21 | 0,0002 |  | P_12,13-DiHOME | diet_1 | no |  | diet_1 | yes | -12,375 | 0,0618 |
| P_12,13-DiHOME | diet_1 | no |  | diet_3 | no | 5,125 | 0,8333 |  | P_12,13-DiHOME | diet_3 | no |  | diet_3 | yes | -31,8214 | <.0001 |
| P_12,13-DiHOME | control | yes |  | diet_1 | yes | 4,3214 | 0,9219 |  |  |  |  |  |  |  |  |  |
| P_12,13-DiHOME | control | yes |  | diet_3 | yes | -10 | 0,2632 |  |  |  |  |  |  |  |  |  |
| P_12,13-DiHOME | diet_1 | yes |  | diet_3 | yes | -14,3214 | 0,0268 |  |  |  |  |  |  |  |  |  |
|  |  |  |  |  |  |  |  |  |  |  |  |  |  |  |  |  |
|  |  |  |  |  |  |  |  |  |  |  |  |  |  |  |  |  |
| P_9,10-DiHOME | control | no |  | diet_1 | no | 13,625 | 0,0111 |  | P_9,10-DiHOME | control | no |  | control | yes | -9,1071 | 0,2136 |
| P_9,10-DiHOME | control | no |  | diet_3 | no | 19,5 | 0,0001 |  | P_9,10-DiHOME | diet_1 | no |  | diet_1 | yes | -16,125 | 0,0017 |
| P_9,10-DiHOME | diet_1 | no |  | diet_3 | no | 5,875 | 0,6393 |  | P_9,10-DiHOME | diet_3 | no |  | diet_3 | yes | -30,6071 | <.0001 |
| P_9,10-DiHOME | control | yes |  | diet_1 | yes | 6,6071 | 0,5546 |  |  |  |  |  |  |  |  |  |
| P_9,10-DiHOME | control | yes |  | diet_3 | yes | -2 | 0,9962 |  |  |  |  |  |  |  |  |  |
| P_9,10-DiHOME | diet_1 | yes |  | diet_3 | yes | -8,6071 | 0,2676 |  |  |  |  |  |  |  |  |  |

| Compound | Diet | LPS | vs | | Diet | | LPS | | Estimate | | P-value | |  | | Compound | | Diet | | LPS | | vs | | Diet | | LPS | | Estimate | | P-value | |
| --- | --- | --- | --- | --- | --- | --- | --- | --- | --- | --- | --- | --- | --- | --- | --- | --- | --- | --- | --- | --- | --- | --- | --- | --- | --- | --- | --- | --- | --- | --- |
| P_9,12,13-TriHOME | control | no |  | | diet_1 | | no | | 2,33E-15 | | 1 | |  | | P_9,12,13-TriHOME | | control | | no | |  | | control | | yes | | -9,375 | | 0,7211 | |
| P_9,12,13-TriHOME | control | no |  | | diet_3 | | no | | 9,625 | | 0,6675 | |  | | P_9,12,13-TriHOME | | diet_1 | | no | |  | | diet_1 | | yes | | -3,875 | | 0,9902 | |
| P_9,12,13-TriHOME | diet_1 | no |  | | diet_3 | | no | | 9,625 | | 0,6675 | |  | | P_9,12,13-TriHOME | | diet_3 | | no | |  | | diet_3 | | yes | | -12,5714 | | 0,4228 | |
| P_9,12,13-TriHOME | control | yes |  | | diet_1 | | yes | | 5,5 | | 0,9607 | |  | |  | |  | |  | |  | |  | |  | |  | |  | |
| P_9,12,13-TriHOME | control | yes |  | | diet_3 | | yes | | 6,4286 | | 0,9347 | |  | |  | |  | |  | |  | |  | |  | |  | |  | |
| P_9,12,13-TriHOME | diet_1 | yes |  | | diet_3 | | yes | | 0,9286 | | 1 | |  | |  | |  | |  | |  | |  | |  | |  | |  | |
|  |  |  |  | |  | |  | |  | |  | |  | |  | |  | |  | |  | |  | |  | |  | |  | |
| P_9,10,13-TriHOME | control | no |  | | diet_1 | | no | | -0,625 | | 1 | |  | | P_9,10,13-TriHOME | | control | | no | |  | | control | | yes | | -12,75 | | 0,2955 | |
| P_9,10,13-TriHOME | control | no |  | | diet_3 | | no | | 13 | | 0,2416 | |  | | P_9,10,13-TriHOME | | diet_1 | | no | |  | | diet_3 | | yes | | -4,6964 | | 0,9689 | |
| P_9,10,13-TriHOME | diet_1 | no |  | | diet_3 | | no | | 13,625 | | 0,1985 | |  | | P_9,10,13-TriHOME | | diet_3 | | no | |  | | diet_3 | | yes | | -18,3214 | | 0,0431 | |
| P_9,10,13-TriHOME | control | yes |  | | diet_1 | | yes | | 9 | | 0,6662 | |  | |  | |  | |  | |  | |  | |  | |  | |  | |
| P_9,10,13-TriHOME | control | yes |  | | diet_3 | | yes | | 7,4286 | | 0,8348 | |  | |  | |  | |  | |  | |  | |  | |  | |  | |
| P_9,10,13-TriHOME | diet_1 | yes |  | | diet_3 | | yes | | -1,5714 | | 0,9998 | |  | |  | |  | |  | |  | |  | |  | |  | |  | |
|  |  |  |  | |  | |  | |  | |  | |  | |  | |  | |  | |  | |  | |  | |  | |  | |
| P_UK1 | control | no |  | | diet_1 | | no | | -1,7108 | | 0,9997 | |  | | P_UK1 | | control | | no | |  | | control | | yes | | -15,0854 | | 0,206 | |
| P_UK1 | control | no |  | | diet_3 | | no | | 14,8181 | | 0,2037 | |  | | P_UK1 | | diet_1 | | no | |  | | diet_1 | | yes | | -7,186 | | 0,8293 | |
| P_UK1 | diet_1 | no |  | | diet_3 | | no | | 16,5289 | | 0,1306 | |  | | P_UK1 | | diet_3 | | no | |  | | diet_3 | | yes | | -22,1547 | | 0,0306 | |
| P_UK1 | control | yes |  | | diet_1 | | yes | | 6,1886 | | 0,9056 | |  | |  | |  | |  | |  | |  | |  | |  | |  | |
| P_UK1 | control | yes |  | | diet_3 | | yes | | 7,7489 | | 0,8093 | |  | |  | |  | |  | |  | |  | |  | |  | |  | |
| P_UK1 | diet_1 | yes |  | | diet_3 | | yes | | 1,5602 | | 0,9998 | |  | |  | |  | |  | |  | |  | |  | |  | |  | |
|  |  |  |  | |  | |  | |  | |  | |  | |  | |  | |  | |  | |  | |  | |  | |  | |
| P_UK2 | control | no |  | | diet_1 | | no | | 0,009935 | | 0,5503 | |  | | P_UK2 | | control | | no | |  | | control | | yes | | -0,01578 | | 0,1523 | |
| P_UK2 | control | no |  | | diet_3 | | no | | 0,01349 | | 0,2584 | |  | | P_UK2 | | diet_1 | | no | |  | | diet_1 | | yes | | -0,01241 | | 0,3327 | |
| P_UK2 | diet_1 | no |  | | diet_3 | | no | | 0,003555 | | 0,9879 | |  | | P_UK2 | | diet_3 | | no | |  | | diet_3 | | yes | | -0,01233 | | 0,3511 | |
| P_UK2 | control | yes |  | | diet_1 | | yes | | 0,0133 | | 0,2822 | |  | |  | |  | |  | |  | |  | |  | |  | |  | |
| P_UK2 | control | yes |  | | diet_3 | | yes | | 0,01693 | | 0,1188 | |  | |  | |  | |  | |  | |  | |  | |  | |  | |
| P_UK2 | diet_1 | yes |  | | diet_3 | | yes | | 0,003634 | | 0,9874 | |  | |  | |  | |  | |  | |  | |  | |  | |  | |
| **Compound** | **Diet** | **LPS** | | **vs** | | **Diet** | | **LPS** | | **Estimate** | | **P-value** | |  | | **Compound** | | **Diet** | | **LPS** | | **vs** | | **Diet** | | **LPS** | | **Estimate** | | **P-value** |
| P_UK3 | control | no |  | | diet_1 | | no | | 0,003395 | | 0,979 | |  | | P_UK3 | | control | | no | |  | | control | | yes | | -0,01335 | | 0,1518 | |
| P_UK3 | control | no |  | | diet_3 | | no | | 0,005458 | | 0,8653 | |  | | P_UK3 | | diet_1 | | no | |  | | diet_1 | | yes | | -0,01468 | | 0,0919 | |
| P_UK3 | diet_1 | no |  | | diet_3 | | no | | 0,002063 | | 0,9978 | |  | | P_UK3 | | diet_3 | | no | |  | | diet_3 | | yes | | -0,01499 | | 0,0902 | |
| P_UK3 | control | yes |  | | diet_1 | | yes | | 0,002066 | | 0,998 | |  | |  | |  | |  | |  | |  | |  | |  | |  | |
| P_UK3 | control | yes |  | | diet_3 | | yes | | 0,003815 | | 0,9702 | |  | |  | |  | |  | |  | |  | |  | |  | |  | |
| P_UK3 | diet_1 | yes |  | | diet_3 | | yes | | 0,001749 | | 0,9991 | |  | |  | |  | |  | |  | |  | |  | |  | |  | |
|  |  |  |  | |  | |  | |  | |  | |  | |  | |  | |  | |  | |  | |  | |  | |  | |
|  |  |  |  | |  | |  | |  | |  | |  | |  | |  | |  | |  | |  | |  | |  | |  | |
| P_UK4 | control | no |  | | diet_1 | | no | | 0,007939 | | 0,2666 | |  | | P_UK4 | | control | | no | |  | | control | | yes | | -0,00236 | | 0,9798 | |
| P_UK4 | control | no |  | | diet_3 | | no | | 0,01071 | | 0,0846 | |  | | P_UK4 | | diet_1 | | no | |  | | diet_1 | | yes | | -0,006 | | 0,5234 | |
| P_UK4 | diet_1 | no |  | | diet_3 | | no | | 0,002773 | | 0,957 | |  | | P_UK4 | | diet_3 | | no | |  | | diet_3 | | yes | | -0,00567 | | 0,5967 | |
| P_UK4 | control | yes |  | | diet_1 | | yes | | 0,004298 | | 0,8081 | |  | |  | |  | |  | |  | |  | |  | |  | |  | |
| P_UK4 | control | yes |  | | diet_3 | | yes | | 0,007403 | | 0,3649 | |  | |  | |  | |  | |  | |  | |  | |  | |  | |
| P_UK4 | diet_1 | yes |  | | diet_3 | | yes | | 0,003105 | | 0,9384 | |  | |  | |  | |  | |  | |  | |  | |  | |  | |
|  |  |  |  | |  | |  | |  | |  | |  | |  | |  | |  | |  | |  | |  | |  | |  | |
|  |  |  |  | |  | |  | |  | |  | |  | |  | |  | |  | |  | |  | |  | |  | |  | |
| P_UK5 | control | no |  | | diet_1 | | no | | 0,007889 | | 0,5067 | |  | | P_UK5 | | control | | no | |  | | control | | yes | | -0,01371 | | 0,0859 | |
| P_UK5 | control | no |  | | diet_3 | | no | | 0,009648 | | 0,3103 | |  | | P_UK5 | | diet_1 | | no | |  | | diet_1 | | yes | | -0,0089 | | 0,387 | |
| P_UK5 | diet_1 | no |  | | diet_3 | | no | | 0,001759 | | 0,9983 | |  | | P_UK5 | | diet_3 | | no | |  | | diet_3 | | yes | | -0,01016 | | 0,286 | |
| P_UK5 | control | yes |  | | diet_1 | | yes | | 0,0127 | | 0,1232 | |  | |  | |  | |  | |  | |  | |  | |  | |  | |
| P_UK5 | control | yes |  | | diet_3 | | yes | | 0,0132 | | 0,115 | |  | |  | |  | |  | |  | |  | |  | |  | |  | |
| P_UK5 | diet_1 | yes |  | | diet_3 | | yes | | 0,000501 | | 1 | |  | |  | |  | |  | |  | |  | |  | |  | |  | |
|  |  |  |  | |  | |  | |  | |  | |  | |  | |  | |  | |  | |  | |  | |  | |  | |
|  |  |  |  | |  | |  | |  | |  | |  | |  | |  | |  | |  | |  | |  | |  | |  | |
| P_EPEA | control | no |  | | diet_1 | | no | | -7,7349 | | 0,5474 | |  | | P_EPEA | | control | | no | |  | | control | | yes | | -3,55E-15 | | 1 | |
| P_EPEA | control | no |  | | diet_3 | | no | | -13,7471 | | 0,0789 | |  | | P_EPEA | | diet_1 | | no | |  | | diet_1 | | yes | | -12,0915 | | 0,1454 | |
| P_EPEA | diet_1 | no |  | | diet_3 | | no | | -6,0122 | | 0,7644 | |  | | P_EPEA | | diet_3 | | no | |  | | diet_3 | | yes | | -11,2968 | | 0,2156 | |
| P_EPEA | control | yes |  | | diet_1 | | yes | | -19,8264 | | 0,0092 | |  | |  | |  | |  | |  | |  | |  | |  | |  | |
| P_EPEA | control | yes |  | | diet_3 | | yes | | -25,0439 | | 0,0017 | |  | |  | |  | |  | |  | |  | |  | |  | |  | |
| P_EPEA | diet_1 | yes |  | | diet_3 | | yes | | -5,2175 | | 0,865 | |  | |  | |  | |  | |  | |  | |  | |  | |  | |

| Compound | Diet | LPS | vs | Diet | LPS | Estimate | P-value |  | Compound | Diet | LPS | vs | Diet | LPS | Estimate | P-value |
| --- | --- | --- | --- | --- | --- | --- | --- | --- | --- | --- | --- | --- | --- | --- | --- | --- |
| P_DHEA | control | no |  | diet_1 | no | -16,7675 | 0,0089 |  | P_DHEA | control | no |  | control | yes | -15,4837 | 0,0178 |
| P_DHEA | control | no |  | diet_3 | no | -12,4171 | 0,0635 |  | P_DHEA | diet_1 | no |  | diet_1 | yes | -14,6465 | 0,0233 |
| P_DHEA | diet_1 | no |  | diet_3 | no | 4,3504 | 0,8685 |  | P_DHEA | diet_3 | no |  | diet_3 | yes | -23,4123 | 0,0006 |
| P_DHEA | control | yes |  | diet_1 | yes | -15,9303 | 0,0145 |  |  |  |  |  |  |  |  |  |
| P_DHEA | control | yes |  | diet_3 | yes | -20,3457 | 0,0024 |  |  |  |  |  |  |  |  |  |
| P_DHEA | diet_1 | yes |  | diet_3 | yes | -4,4154 | 0,869 |  |  |  |  |  |  |  |  |  |
|  |  |  |  |  |  |  |  |  |  |  |  |  |  |  |  |  |
|  |  |  |  |  |  |  |  |  |  |  |  |  |  |  |  |  |
| P_AEA | control | no |  | diet_1 | no | 0,2491 | <.0001 |  | P_AEA | control | no |  | control | yes | -0,1614 | <.0001 |
| P_AEA | control | no |  | diet_3 | no | 0,2741 | <.0001 |  | P_AEA | diet_1 | no |  | diet_1 | yes | -0,0382 | 0,4385 |
| P_AEA | diet_1 | no |  | diet_3 | no | 0,02502 | 0,8242 |  | P_AEA | diet_3 | no |  | diet_3 | yes | -0,0435 | 0,3334 |
| P_AEA | control | yes |  | diet_1 | yes | 0,3723 | <.0001 |  |  |  |  |  |  |  |  |  |
| P_AEA | control | yes |  | diet_3 | yes | 0,3921 | <.0001 |  |  |  |  |  |  |  |  |  |
| P_AEA | diet_1 | yes |  | diet_3 | yes | 0,01972 | 0,9364 |  |  |  |  |  |  |  |  |  |
|  |  |  |  |  |  |  |  |  |  |  |  |  |  |  |  |  |
|  |  |  |  |  |  |  |  |  |  |  |  |  |  |  |  |  |
| P_2-AG | control | no |  | diet_1 | no | 14,0536 | 0,0164 |  | P_2-AG | control | no |  | control | yes | 5,3457 | 0,6627 |
| P_2-AG | control | no |  | diet_3 | no | 26,5759 | <.0001 |  | P_2-AG | diet_1 | no |  | diet_1 | yes | 16,4764 | 0,0054 |
| P_2-AG | diet_1 | no |  | diet_3 | no | 12,5223 | 0,0337 |  | P_2-AG | diet_3 | no |  | diet_3 | yes | 2,3645 | 0,9818 |
| P_2-AG | control | yes |  | diet_1 | yes | 25,1843 | 0,0002 |  |  |  |  |  |  |  |  |  |
| P_2-AG | control | yes |  | diet_3 | yes | 23,5947 | 0,0005 |  |  |  |  |  |  |  |  |  |
| P_2-AG | diet_1 | yes |  | diet_3 | yes | -1,5895 | 0,997 |  |  |  |  |  |  |  |  |  |
|  |  |  |  |  |  |  |  |  |  |  |  |  |  |  |  |  |
|  |  |  |  |  |  |  |  |  |  |  |  |  |  |  |  |  |
| P_DLE | control | no |  | diet_1 | no | 24,816 | <.0001 |  | P_DLE | control | no |  | control | yes | -7,7617 | 0,122 |
| P_DLE | control | no |  | diet_3 | no | 24,816 | <.0001 |  | P_DLE | diet_1 | no |  | diet_1 | yes | -13,6959 | 0,0042 |
| P_DLE | diet_1 | no |  | diet_3 | no | 5,11E-15 | 1 |  | P_DLE | diet_3 | no |  | diet_3 | yes | -12,1487 | 0,0116 |
| P_DLE | control | yes |  | diet_1 | no | 32,5777 | <.0001 |  |  |  |  |  |  |  |  |  |
| P_DLE | control | yes |  | diet_3 | yes | 20,4291 | 0,0003 |  |  |  |  |  |  |  |  |  |
| P_DLE | diet_1 | yes |  | diet_3 | yes | 1,5472 | 0,9899 |  |  |  |  |  |  |  |  |  |

| Compound | Diet | LPS | vs | Diet | LPS | Estimate | P-value |  | Compound | Diet | LPS | vs | Diet | LPS | Estimate | P-value |
| --- | --- | --- | --- | --- | --- | --- | --- | --- | --- | --- | --- | --- | --- | --- | --- | --- |
| P_PEA | control | no |  | diet_1 | no | 5,125 | 0,4845 |  | P_PEA | control | no |  | control | yes | -17,9107 | <.0001 |
| P_PEA | control | no |  | diet_3 | no | 14,75 | 0,0001 |  | P_PEA | diet_1 | no |  | diet_1 | yes | -18,25 | <.0001 |
| P_PEA | diet_1 | no |  | diet_3 | no | 9,625 | 0,02 |  | P_PEA | diet_3 | no |  | diet_3 | yes | -31,6607 | <.0001 |
| P_PEA | control | yes |  | diet_1 | yes | 4,7857 | 0,5949 |  |  |  |  |  |  |  |  |  |
| P_PEA | control | yes |  | diet_3 | yes | 1 | 0,9995 |  |  |  |  |  |  |  |  |  |
| P_PEA | diet_1 | yes |  | diet_3 | yes | -3,7857 | 0,796 |  |  |  |  |  |  |  |  |  |
|  |  |  |  |  |  |  |  |  |  |  |  |  |  |  |  |  |
|  |  |  |  |  |  |  |  |  |  |  |  |  |  |  |  |  |
| P_OEA | control | no |  | diet_1 | no | 8,0036 | 0,0231 |  | P_OEA | control | no |  | control | yes | -21,0672 | <.0001 |
| P_OEA | control | no |  | diet_3 | no | 15,883 | <.0001 |  | P_OEA | diet_1 | no |  | diet_1 | yes | -21,4044 | <.0001 |
| P_OEA | diet_1 | no |  | diet_3 | no | 7,8795 | 0,0255 |  | P_OEA | diet_3 | no |  | diet_3 | yes | -26,5084 | <.0001 |
| P_OEA | control | yes |  | diet_1 | yes | 7,6663 | 0,0368 |  |  |  |  |  |  |  |  |  |
| P_OEA | control | yes |  | diet_3 | yes | 10,4418 | 0,0055 |  |  |  |  |  |  |  |  |  |
| P_OEA | diet_1 | yes |  | diet_3 | yes | 2,7755 | 0,7877 |  |  |  |  |  |  |  |  |  |
|  |  |  |  |  |  |  |  |  |  |  |  |  |  |  |  |  |
|  |  |  |  |  |  |  |  |  |  |  |  |  |  |  |  |  |
| P_SEA | control | no |  | diet_1 | no | -2,25 | 0,9803 |  | P_SEA | control | no |  | control | yes | -23,4107 | <.0001 |
| P_SEA | control | no |  | diet_3 | no | 10,75 | 0,0196 |  | P_SEA | diet_1 | no |  | diet_1 | yes | -15,75 | 0,0002 |
| P_SEA | diet_1 | no |  | diet_3 | no | 13 | 0,0028 |  | P_SEA | diet_3 | no |  | diet_3 | yes | -26,5893 | <.0001 |
| P_SEA | control | yes |  | diet_1 | yes | 5,4107 | 0,5801 |  |  |  |  |  |  |  |  |  |
| P_SEA | control | yes |  | diet_3 | yes | 7,5714 | 0,2527 |  |  |  |  |  |  |  |  |  |
| P_SEA | diet_1 | yes |  | diet_3 | yes | 2,1607 | 0,9859 |  |  |  |  |  |  |  |  |  |

| **Compound** | **Diet** | **LPS** | **vs** | **Diet** | **LPS** | **Estimate** | **P-value** |  | **Compound** | **Diet** | **LPS** | **vs** | **Diet** | **LPS** | **Estimate** | **P-value** |
| --- | --- | --- | --- | --- | --- | --- | --- | --- | --- | --- | --- | --- | --- | --- | --- | --- |
| L_AA | control | no |  | diet_1 | no | 18,4532 | 0,0073 |  | L_AA | control | no |  | control | yes | -7,721 | 0,439 |
| L_AA | control | no |  | diet_3 | no | 29,4787 | 0,0002 |  | L_AA | diet_1 | no |  | diet_1 | yes | -3,1028 | 0,9649 |
| L_AA | diet_1 | no |  | diet_3 | no | 11,0256 | 0,1365 |  | L_AA | diet_3 | no |  | diet_3 | yes | -18,8352 | 0,0069 |
| L_AA | control | yes |  | diet_1 | yes | 23,0714 | 0,0015 |  |  |  |  |  |  |  |  |  |
| L_AA | control | yes |  | diet_3 | yes | 18,3646 | 0,0089 |  |  |  |  |  |  |  |  |  |
| L_AA | diet_1 | yes |  | diet_3 | yes | -4,7068 | 0,8408 |  |  |  |  |  |  |  |  |  |
|  |  |  |  |  |  |  |  |  |  |  |  |  |  |  |  |  |
|  |  |  |  |  |  |  |  |  |  |  |  |  |  |  |  |  |
| L_EPA | control | no |  | diet_1 | no | -25,9914 | 0,0006 |  | L_EPA | control | no |  | control | yes | -4,1435 | 0,9245 |
| L_EPA | control | no |  | diet_3 | no | -27,6018 | 0,0003 |  | L_EPA | diet_1 | no |  | diet_1 | yes | 9,1947 | 0,3254 |
| L_EPA | diet_1 | no |  | diet_3 | no | -1,6104 | 0,9987 |  | L_EPA | diet_3 | no |  | diet_3 | yes | -2,8873 | 0,9829 |
| L_EPA | control | yes |  | diet_1 | yes | -12,6532 | 0,105 |  |  |  |  |  |  |  |  |  |
| L_EPA | control | yes |  | diet_3 | yes | -26,3456 | 0,0007 |  |  |  |  |  |  |  |  |  |
| L_EPA | diet_1 | yes |  | diet_3 | yes | -13,6924 | 0,0711 |  |  |  |  |  |  |  |  |  |
|  |  |  |  |  |  |  |  |  |  |  |  |  |  |  |  |  |
|  |  |  |  |  |  |  |  |  |  |  |  |  |  |  |  |  |
| L_DHA | control | no |  | diet_1 | no | -9038,5 | 0,6357 |  | L_DHA | control | no |  | control | yes | -11636 | 0,403 |
| L_DHA | control | no |  | diet_3 | no | -5538,85 | 0,9221 |  | L_DHA | diet_1 | no |  | diet_1 | yes | -2356,07 | 0,9981 |
| L_DHA | diet_1 | no |  | diet_3 | no | 3499,65 | 0,9884 |  | L_DHA | diet_3 | no |  | diet_3 | yes | -16084 | 0,1401 |
| L_DHA | control | yes |  | diet_1 | yes | 241,16 | 1 |  |  |  |  |  |  |  |  |  |
| L_DHA | control | yes |  | diet_3 | yes | -9987,61 | 0,5611 |  |  |  |  |  |  |  |  |  |
| L_DHA | diet_1 | yes |  | diet_3 | yes | -10229 | 0,5291 |  |  |  |  |  |  |  |  |  |
|  |  |  |  |  |  |  |  |  |  |  |  |  |  |  |  |  |
|  |  |  |  |  |  |  |  |  |  |  |  |  |  |  |  |  |
| L_PGE2 | control | no |  | diet_1 | no | 19,9797 | 0,0036 |  | L_PGE2 | control | no |  | control | yes | -7,5699 | 0,4888 |
| L_PGE2 | control | no |  | diet_3 | no | 24,7573 | 0,0006 |  | L_PGE2 | diet_1 | no |  | diet_1 | yes | -8,4361 | 0,3503 |
| L_PGE2 | diet_1 | no |  | diet_3 | no | 4,7776 | 0,8344 |  | L_PGE2 | diet_3 | no |  | diet_3 | yes | -18,9662 | 0,0068 |
| L_PGE2 | control | yes |  | diet_1 | yes | 19,1135 | 0,0065 |  |  |  |  |  |  |  |  |  |
| L_PGE2 | control | yes |  | diet_3 | yes | 13,361 | 0,0755 |  |  |  |  |  |  |  |  |  |
| L_PGE2 | diet_1 | yes |  | diet_3 | yes | -5,7525 | 0,7353 |  |  |  |  |  |  |  |  |  |

| Compound | Diet | LPS | vs | Diet | LPS | Estimate | P-value |  | Compound | Diet | LPS | vs | Diet | LPS | Estimate | P-value |
| --- | --- | --- | --- | --- | --- | --- | --- | --- | --- | --- | --- | --- | --- | --- | --- | --- |
| L_PGD2 | control | no |  | diet_1 | no | 1,1875 | 0,9997 |  | L_PGD2 | control | no |  | control | yes | -8,2589 | 0,4188 |
| L_PGD2 | control | no |  | diet_3 | no | 12,375 | 0,0563 |  | L_PGD2 | diet_1 | no |  | diet_1 | yes | 19,5625 | 0,0005 |
| L_PGD2 | diet_1 | no |  | diet_3 | no | 11,1875 | 0,1065 |  | L_PGD2 | diet_3 | no |  | diet_3 | yes | 8,0089 | 0,4531 |
| L_PGD2 | control | yes |  | diet_1 | yes | 29,0089 | <.0001 |  |  |  |  |  |  |  |  |  |
| L_PGD2 | control | yes |  | diet_3 | yes | 28,6429 | <.0001 |  |  |  |  |  |  |  |  |  |
| L_PGD2 | diet_1 | yes |  | diet_3 | yes | -0,3661 | 1 |  |  |  |  |  |  |  |  |  |
|  |  |  |  |  |  |  |  |  |  |  |  |  |  |  |  |  |
| L_n-acetyl leukotriene E4 | control | no |  | diet_1 | no | 0 | 1 |  | L_n-acetyl leukotriene E4 | control | no |  | control | yes | -16,0714 | 0,0006 |
| L_n-acetyl leukotriene E4 | control | no |  | diet_3 | no | 0 | 1 |  | L_n-acetyl leukotriene E4 | diet_1 | no |  | diet_1 | yes | -8,9375 | 0,1099 |
| L_n-acetyl leukotriene E4 | diet_1 | no |  | diet_3 | no | 0 | 1 |  | L_n-acetyl leukotriene E4 | diet_3 | no |  | diet_3 | yes | 2,88E-14 | 1 |
| L_n-acetyl leukotriene E4 | control | yes |  | diet_1 | yes | 7,1339 | 0,339 |  |  |  |  |  |  |  |  |  |
| L_n-acetyl leukotriene E4 | control | yes |  | diet_3 | yes | 16,0714 | 0,0009 |  |  |  |  |  |  |  |  |  |
| L_n-acetyl leukotriene E4 | diet_1 | yes |  | diet_3 | yes | 8,9375 | 0,1326 |  |  |  |  |  |  |  |  |  |
|  |  |  |  |  |  |  |  |  |  |  |  |  |  |  |  |  |
| L_17 keto- 4(z), 7(z), 10(z), 13 (z), 15 (E), 19(z)-DHA | control | no |  | diet_1 | no | -43,1661 | 0,216 |  | L_17 keto- 4(z), 7(z), 10(z), 13 (z), 15 (E), 19(z)-DHA | control | no |  | control | yes | -24,0707 | 0,7479 |
| L_17 keto- 4(z), 7(z), 10(z), 13 (z), 15 (E), 19(z)-DHA | control | no |  | diet_3 | no | -4,3102 | 0,9998 |  | L_17 keto- 4(z), 7(z), 10(z), 13 (z), 15 (E), 19(z)-DHA | diet_1 | no |  | diet_1 | yes | 28,0965 | 0,6162 |
| L_17 keto- 4(z), 7(z), 10(z), 13 (z), 15 (E), 19(z)-DHA | diet_1 | no |  | diet_3 | no | 38,8558 | 0,3043 |  | L_17 keto- 4(z), 7(z), 10(z), 13 (z), 15 (E), 19(z)-DHA | diet_3 | no |  | diet_3 | yes | -43,0056 | 0,2227 |
| L_17 keto- 4(z), 7(z), 10(z), 13 (z), 15 (E), 19(z)-DHA | control | yes |  | diet_1 | yes | 9,0012 | 0,9948 |  |  |  |  |  |  |  |  |  |
| L_17 keto- 4(z), 7(z), 10(z), 13 (z), 15 (E), 19(z)-DHA | control | yes |  | diet_3 | yes | -23,2451 | 0,7757 |  |  |  |  |  |  |  |  |  |
| L_17 keto- 4(z), 7(z), 10(z), 13 (z), 15 (E), 19(z)-DHA | diet_1 | yes |  | diet_3 | yes | -32,2463 | 0,4889 |  |  |  |  |  |  |  |  |  |

| Compound | Diet | LPS | vs | Diet | LPS | Estimate | P-value |  | Compound | Diet | LPS | vs | Diet | LPS | Estimate | P-value |
| --- | --- | --- | --- | --- | --- | --- | --- | --- | --- | --- | --- | --- | --- | --- | --- | --- |
| L_12,13-DiHOME | control | no |  | diet_1 | no | 1,8374 | 0,9999 |  | L_12,13-DiHOME | control | no |  | control | yes | 10,091 | 0,7791 |
| L_12,13-DiHOME | control | no |  | diet_3 | no | 15,6856 | 0,3797 |  | L_12,13-DiHOME | diet_1 | no |  | diet_1 | yes | 22,7952 | 0,102 |
| L_12,13-DiHOME | diet_1 | no |  | diet_3 | no | 13,8482 | 0,501 |  | L_12,13-DiHOME | diet_3 | no |  | diet_3 | yes | -6,6002 | 0,9507 |
| L_12,13-DiHOME | control | yes |  | diet_1 | yes | 14,5416 | 0,4639 |  |  |  |  |  |  |  |  |  |
| L_12,13-DiHOME | control | yes |  | diet_3 | yes | -1,0056 | 1 |  |  |  |  |  |  |  |  |  |
| L_12,13-DiHOME | diet_1 | yes |  | diet_3 | yes | -15,5472 | 0,3987 |  |  |  |  |  |  |  |  |  |
|  |  |  |  |  |  |  |  |  |  |  |  |  |  |  |  |  |
|  |  |  |  |  |  |  |  |  |  |  |  |  |  |  |  |  |
| L_9,10-DiHOME | control | no |  | diet_1 | no | -11,9315 | 0,9999 |  | L_9,10-DiHOME | control | no |  | control | yes | -22,1853 | 0,9983 |
| L_9,10-DiHOME | control | no |  | diet_3 | no | 76,1423 | 0,7317 |  | L_9,10-DiHOME | diet_1 | no |  | diet_1 | yes | 55,714 | 0,902 |
| L_9,10-DiHOME | diet_1 | no |  | diet_3 | no | 88,0738 | 0,6106 |  | L_9,10-DiHOME | diet_3 | no |  | diet_3 | yes | -100,74 | 0,4979 |
| L_9,10-DiHOME | control | yes |  | diet_1 | yes | 65,9678 | 0,8338 |  |  |  |  |  |  |  |  |  |
| L_9,10-DiHOME | control | yes |  | diet_3 | yes | -2,4172 | 1 |  |  |  |  |  |  |  |  |  |
| L_9,10-DiHOME | diet_1 | yes |  | diet_3 | yes | -68,385 | 0,8135 |  |  |  |  |  |  |  |  |  |
|  |  |  |  |  |  |  |  |  |  |  |  |  |  |  |  |  |
|  |  |  |  |  |  |  |  |  |  |  |  |  |  |  |  |  |
| L_9,12,13-TriHOME | control | no |  | diet_1 | no | 7,375 | 0,6981 |  | L_9,12,13-TriHOME | control | no |  | control | yes | 6,2679 | 0,8395 |
| L_9,12,13-TriHOME | control | no |  | diet_3 | no | 20,5 | 0,0032 |  | L_9,12,13-TriHOME | diet_1 | no |  | diet_1 | yes | 19,25 | 0,0064 |
| L_9,12,13-TriHOME | diet_1 | no |  | diet_3 | no | 13,125 | 0,1268 |  | L_9,12,13-TriHOME | diet_3 | no |  | diet_3 | yes | -12,6607 | 0,1802 |
| L_9,12,13-TriHOME | control | yes |  | diet_1 | yes | 20,3571 | 0,005 |  |  |  |  |  |  |  |  |  |
| L_9,12,13-TriHOME | control | yes |  | diet_3 | yes | 1,5714 | 0,9997 |  |  |  |  |  |  |  |  |  |
| L_9,12,13-TriHOME | diet_1 | yes |  | diet_3 | yes | -18,7857 | 0,0116 |  |  |  |  |  |  |  |  |  |
|  |  |  |  |  |  |  |  |  |  |  |  |  |  |  |  |  |
| L_9,10,13-TriHOME | control | no |  | diet_1 | no | 9,375 | 0,5102 |  | L_9,10,13-TriHOME | control | no |  | control | yes | 6,5357 | 0,8452 |
| L_9,10,13-TriHOME | control | no |  | diet_3 | no | 19,25 | 0,0108 |  | L_9,10,13-TriHOME | diet_1 | no |  | diet_1 | yes | 16,625 | 0,0387 |
| L_9,10,13-TriHOME | diet_1 | no |  | diet_3 | no | 9,875 | 0,4527 |  | L_9,10,13-TriHOME | diet_3 | no |  | diet_3 | yes | -11 | 0,3711 |
| L_9,10,13-TriHOME | control | yes |  | diet_1 | yes | 19,4643 | 0,0136 |  |  |  |  |  |  |  |  |  |
| L_9,10,13-TriHOME | control | yes |  | diet_3 | yes | 1,7143 | 0,9997 |  |  |  |  |  |  |  |  |  |
| L_9,10,13-TriHOME | diet_1 | yes |  | diet_3 | yes | -17,75 | 0,0303 |  |  |  |  |  |  |  |  |  |

| Compound | Diet | LPS | vs | Diet | LPS | Estimate | P-value |  | Compound | Diet | LPS | vs | Diet | LPS | Estimate | P-value |
| --- | --- | --- | --- | --- | --- | --- | --- | --- | --- | --- | --- | --- | --- | --- | --- | --- |
| L_12(S)-HHTrE | control | no |  | diet_1 | no | 15,75 | 0,0036 |  | L_12(S)-HHTrE | control | no |  | control | yes | -6,2143 | 0,6555 |
| L_12(S)-HHTrE | control | no |  | diet_3 | no | 25,875 | <.0001 |  | L_12(S)-HHTrE | diet_1 | no |  | diet_1 | yes | 1,25 | 0,9995 |
| L_12(S)-HHTrE | diet_1 | no |  | diet_3 | no | 10,125 | 0,1317 |  | L_12(S)-HHTrE | diet_3 | no |  | diet_3 | yes | -7,8036 | 0,4139 |
| L_12(S)-HHTrE | control | yes |  | diet_1 | yes | 23,2143 | <.0001 |  |  |  |  |  |  |  |  |  |
| L_12(S)-HHTrE | control | yes |  | diet_3 | yes | 24,2857 | <.0001 |  |  |  |  |  |  |  |  |  |
| L_12(S)-HHTrE | diet_1 | yes |  | diet_3 | yes | 1,0714 | 0,9998 |  |  |  |  |  |  |  |  |  |
|  |  |  |  |  |  |  |  |  |  |  |  |  |  |  |  |  |
|  |  |  |  |  |  |  |  |  |  |  |  |  |  |  |  |  |
| L_9(S)-HODE | control | no |  | diet_1 | no | 12,4357 | 0,6134 |  | L_9(S)-HODE | control | no |  | control | yes | -0,4648 | 1 |
| L_9(S)-HODE | control | no |  | diet_3 | no | 16,2768 | 0,351 |  | L_9(S)-HODE | diet_1 | no |  | diet_1 | yes | -2,4386 | 0,9995 |
| L_9(S)-HODE | diet_1 | no |  | diet_3 | no | 3,8412 | 0,9955 |  | L_9(S)-HODE | diet_3 | no |  | diet_3 | yes | -13,4764 | 0,555 |
| L_9(S)-HODE | control | yes |  | diet_1 | yes | 10,4619 | 0,7688 |  |  |  |  |  |  |  |  |  |
| L_9(S)-HODE | control | yes |  | diet_3 | yes | 3,2652 | 0,9982 |  |  |  |  |  |  |  |  |  |
| L_9(S)-HODE | diet_1 | yes |  | diet_3 | yes | -7,1967 | 0,9368 |  |  |  |  |  |  |  |  |  |
|  |  |  |  |  |  |  |  |  |  |  |  |  |  |  |  |  |
|  |  |  |  |  |  |  |  |  |  |  |  |  |  |  |  |  |
| L_13(S)-HODE | control | no |  | diet_1 | no | 9,3099 | 0,6555 |  | L_13(S)-HODE | control | no |  | control | yes | 9,2116 | 0,6858 |
| L_13(S)-HODE | control | no |  | diet_3 | no | 22,2167 | 0,0377 |  | L_13(S)-HODE | diet_1 | no |  | diet_1 | yes | 15,3554 | 0,2037 |
| L_13(S)-HODE | diet_1 | no |  | diet_3 | no | 12,9068 | 0,3485 |  | L_13(S)-HODE | diet_3 | no |  | diet_3 | yes | -7,8895 | 0,7975 |
| L_13(S)-HODE | control | yes |  | diet_1 | yes | 15,4537 | 0,2178 |  |  |  |  |  |  |  |  |  |
| L_13(S)-HODE | control | yes |  | diet_3 | yes | 5,1157 | 0,9613 |  |  |  |  |  |  |  |  |  |
| L_13(S)-HODE | diet_1 | yes |  | diet_3 | yes | -10,3381 | 0,5848 |  |  |  |  |  |  |  |  |  |
|  |  |  |  |  |  |  |  |  |  |  |  |  |  |  |  |  |
|  |  |  |  |  |  |  |  |  |  |  |  |  |  |  |  |  |
| L_12(S)-HEPE | control | no |  | diet_1 | no | -25,6597 | 0,0041 |  | L_12(S)-HEPE | control | no |  | control | yes | 2,3213 | 0,9974 |
| L_12(S)-HEPE | control | no |  | diet_3 | no | -22,4118 | 0,0112 |  | L_12(S)-HEPE | diet_1 | no |  | diet_1 | yes | 19,5133 | 0,0281 |
| L_12(S)-HEPE | diet_1 | no |  | diet_3 | no | 3,2479 | 0,9874 |  | L_12(S)-HEPE | diet_3 | no |  | diet_3 | yes | -0,0188 | 1 |
| L_12(S)-HEPE | control | yes |  | diet_1 | yes | -8,4678 | 0,6167 |  |  |  |  |  |  |  |  |  |
| L_12(S)-HEPE | control | yes |  | diet_3 | yes | -24,7519 | 0,0063 |  |  |  |  |  |  |  |  |  |
| L_12(S)-HEPE | diet_1 | yes |  | diet_3 | yes | -16,2842 | 0,0822 |  |  |  |  |  |  |  |  |  |

| Compound | Diet | LPS | vs | Diet | LPS | Estimate | P-value |  | Compound | Diet | LPS | vs | Diet | LPS | Estimate | P-value |
| --- | --- | --- | --- | --- | --- | --- | --- | --- | --- | --- | --- | --- | --- | --- | --- | --- |
| L_5(S)-HEPE | control | no |  | diet_1 | no | -4,1884 | <.0001 |  | L_5(S)-HEPE | control | no |  | control | yes | -0,07474 | 1 |
| L_5(S)-HEPE | control | no |  | diet_3 | no | -5,8179 | <.0001 |  | L_5(S)-HEPE | diet_1 | no |  | diet_1 | yes | 1,8367 | 0,2256 |
| L_5(S)-HEPE | diet_1 | no |  | diet_3 | no | -1,6295 | 0,3475 |  | L_5(S)-HEPE | diet_3 | no |  | diet_3 | yes | 0,2786 | 0,9994 |
| L_5(S)-HEPE | control | yes |  | diet_1 | yes | -2,277 | 0,091 |  |  |  |  |  |  |  |  |  |
| L_5(S)-HEPE | control | yes |  | diet_3 | yes | -5,4646 | <.0001 |  |  |  |  |  |  |  |  |  |
| L_5(S)-HEPE | diet_1 | yes |  | diet_3 | yes | -3,1876 | 0,0056 |  |  |  |  |  |  |  |  |  |
|  |  |  |  |  |  |  |  |  |  |  |  |  |  |  |  |  |
|  |  |  |  |  |  |  |  |  |  |  |  |  |  |  |  |  |
| L_20(S)-HETE | control | no |  | diet_1 | no | -1,95E-14 | 1 |  | L_20(S)-HETE | control | no |  | control | yes | -23,7788 | 0,0024 |
| L_20(S)-HETE | control | no |  | diet_3 | no | -4,97E-14 | 1 |  | L_20(S)-HETE | diet_1 | no |  | diet_1 | yes | -6,4145 | 0,7135 |
| L_20(S)-HETE | diet_1 | no |  | diet_3 | no | -3,02E-14 | 1 |  | L_20(S)-HETE | diet_3 | no |  | diet_3 | yes | -3,8412 | 0,9516 |
| L_20(S)-HETE | control | yes |  | diet_1 | yes | 17,3644 | 0,0235 |  |  |  |  |  |  |  |  |  |
| L_20(S)-HETE | control | yes |  | diet_3 | yes | 19,9377 | 0,0095 |  |  |  |  |  |  |  |  |  |
| L_20(S)-HETE | diet_1 | yes |  | diet_3 | yes | 2,5733 | 0,9913 |  |  |  |  |  |  |  |  |  |
|  |  |  |  |  |  |  |  |  |  |  |  |  |  |  |  |  |
|  |  |  |  |  |  |  |  |  |  |  |  |  |  |  |  |  |
| L_15(S)-HETE | control | no |  | diet_1 | no | 21,0952 | 0,0039 |  | L_15(S)-HETE | control | no |  | control | yes | -0,2956 | 1 |
| L_15(S)-HETE | control | no |  | diet_3 | no | 29,1702 | 0,0003 |  | L_15(S)-HETE | diet_1 | no |  | diet_1 | yes | 2,063 | 0,9952 |
| L_15(S)-HETE | diet_1 | no |  | diet_3 | no | 8,0751 | 0,4282 |  | L_15(S)-HETE | diet_3 | no |  | diet_3 | yes | -15,2518 | 0,0394 |
| L_15(S)-HETE | control | yes |  | diet_1 | yes | 23,4538 | 0,0021 |  |  |  |  |  |  |  |  |  |
| L_15(S)-HETE | control | yes |  | diet_3 | yes | 14,214 | 0,066 |  |  |  |  |  |  |  |  |  |
| L_15(S)-HETE | diet_1 | yes |  | diet_3 | yes | -9,2398 | 0,3253 |  |  |  |  |  |  |  |  |  |
|  |  |  |  |  |  |  |  |  |  |  |  |  |  |  |  |  |
|  |  |  |  |  |  |  |  |  |  |  |  |  |  |  |  |  |
| L_11(S)-HETE | control | no |  | diet_1 | no | 21,375 | <.0001 |  | L_11(S)-HETE | control | no |  | control | yes | -6,4464 | 0,4994 |
| L_11(S)-HETE | control | no |  | diet_3 | no | 25,125 | <.0001 |  | L_11(S)-HETE | diet_1 | no |  | diet_1 | yes | -2,375 | 0,984 |
| L_11(S)-HETE | diet_1 | no |  | diet_3 | no | 3,75 | 0,8932 |  | L_11(S)-HETE | diet_3 | no |  | diet_3 | yes | -17,1429 | 0,0004 |
| L_11(S)-HETE | control | yes |  | diet_1 | yes | 25,4464 | <.0001 |  |  |  |  |  |  |  |  |  |
| L_11(S)-HETE | control | yes |  | diet_3 | yes | 14,4286 | 0,0056 |  |  |  |  |  |  |  |  |  |
| L_11(S)-HETE | diet_1 | yes |  | diet_3 | yes | -11,017 | 0,0473 |  |  |  |  |  |  |  |  |  |
| L_12(S)-HETE | control | no |  | diet_1 | no | 15,785 | 0,0413 |  | L_12(S)-HETE | control | no |  | control | yes | 9,2354 | 0,3783 |
| L_12(S)-HETE | control | no |  | diet_3 | no | 24,849 | 0,0021 |  | L_12(S)-HETE | diet_1 | no |  | diet_1 | yes | 18,4873 | 0,0163 |
| L_12(S)-HETE | diet_1 | no |  | diet_3 | no | 9,0639 | 0,3728 |  | L_12(S)-HETE | diet_3 | no |  | diet_3 | yes | -3,9109 | 0,9454 |
| L_12(S)-HETE | control | yes |  | diet_1 | yes | 25,0369 | 0,0024 |  |  |  |  |  |  |  |  |  |
| L_12(S)-HETE | control | yes |  | diet_3 | yes | 11,7027 | 0,1987 |  |  |  |  |  |  |  |  |  |
| L_12(S)-HETE | diet_1 | yes |  | diet_3 | yes | -13,3343 | 0,1075 |  |  |  |  |  |  |  |  |  |
|  |  |  |  |  |  |  |  |  |  |  |  |  |  |  |  |  |
|  |  |  |  |  |  |  |  |  |  |  |  |  |  |  |  |  |
| L_11,12 EET | control | no |  | diet_1 | no | 0,3008 | 0,7658 |  | L_11,12 EET | control | no |  | control | yes | -0,503 | 0,3138 |
| L_11,12 EET | control | no |  | diet_3 | no | 0,4459 | 0,4179 |  | L_11,12 EET | diet_1 | no |  | diet_1 | yes | -0,0128 | 1 |
| L_11,12 EET | diet_1 | no |  | diet_3 | no | 0,1451 | 0,985 |  | L_11,12 EET | diet_3 | no |  | diet_3 | yes | -0,1241 | 0,9929 |
| L_11,12 EET | control | yes |  | diet_1 | yes | 0,791 | 0,0487 |  |  |  |  |  |  |  |  |  |
| L_11,12 EET | control | yes |  | diet_3 | yes | 0,8247 | 0,0408 |  |  |  |  |  |  |  |  |  |
| L_11,12 EET | diet_1 | yes |  | diet_3 | yes | 0,03371 | 1 |  |  |  |  |  |  |  |  |  |
|  |  |  |  |  |  |  |  |  |  |  |  |  |  |  |  |  |
|  |  |  |  |  |  |  |  |  |  |  |  |  |  |  |  |  |
| L_5,6 EET | control | no |  | diet_1 | no | 12,0625 | 0,0604 |  | L_5,6 EET | control | no |  | control | yes | -8,8036 | 0,3317 |
| L_5,6 EET | control | no |  | diet_3 | no | 22,125 | <.0001 |  | L_5,6 EET | diet_1 | no |  | diet_1 | yes | 3 | 0,9778 |
| L_5,6 EET | diet_1 | no |  | diet_3 | no | 10,0625 | 0,1706 |  | L_5,6 EET | diet_3 | no |  | diet_3 | yes | -9,6429 | 0,2388 |
| L_5,6 EET | control | yes |  | diet_1 | yes | 23,8661 | <.0001 |  |  |  |  |  |  |  |  |  |
| L_5,6 EET | control | yes |  | diet_3 | yes | 21,2857 | 0,0003 |  |  |  |  |  |  |  |  |  |
| L_5,6 EET | diet_1 | yes |  | diet_3 | yes | -2,5804 | 0,9903 |  |  |  |  |  |  |  |  |  |
|  |  |  |  |  |  |  |  |  |  |  |  |  |  |  |  |  |
|  |  |  |  |  |  |  |  |  |  |  |  |  |  |  |  |  |
| L_5(S)-HETE | control | no |  | diet_1 | no | 20,1548 | 0,0744 |  | L_5(S)-HETE | control | no |  | control | yes | -5,4402 | 0,9532 |
| L_5(S)-HETE | control | no |  | diet_3 | no | 14,0554 | 0,3076 |  | L_5(S)-HETE | diet_1 | no |  | diet_1 | yes | -3,7011 | 0,9903 |
| L_5(S)-HETE | diet_1 | no |  | diet_3 | no | -6,0994 | 0,9222 |  | L_5(S)-HETE | diet_3 | no |  | diet_3 | yes | -13,0229 | 0,3945 |
| L_5(S)-HETE | control | yes |  | diet_1 | yes | 21,8938 | 0,0527 |  |  |  |  |  |  |  |  |  |
| L_5(S)-HETE | control | yes |  | diet_3 | yes | 6,4726 | 0,914 |  |  |  |  |  |  |  |  |  |
| L_5(S)-HETE | diet_1 | yes |  | diet_3 | yes | -15,4212 | 0,2423 |  |  |  |  |  |  |  |  |  |

| Compound | Diet | LPS | vs | Diet | LPS | Estimate | P-value |  | Compound | Diet | LPS | vs | Diet | LPS | Estimate | P-value |
| --- | --- | --- | --- | --- | --- | --- | --- | --- | --- | --- | --- | --- | --- | --- | --- | --- |
| L_14,15 EET | control | no |  | diet_1 | no | 12,8744 | 0,1338 |  | L_14,15 EET | control | no |  | control | yes | -9,8033 | 0,3834 |
| L_14,15 EET | control | no |  | diet_3 | no | 21,8766 | 0,0052 |  | L_14,15 EET | diet_1 | no |  | diet_1 | yes | -1,3732 | 0,9996 |
| L_14,15 EET | diet_1 | no |  | diet_3 | no | 9,0022 | 0,4338 |  | L_14,15 EET | diet_3 | no |  | diet_3 | yes | -11,5338 | 0,2353 |
| L_14,15 EET | control | yes |  | diet_1 | yes | 21,3045 | 0,0083 |  |  |  |  |  |  |  |  |  |
| L_14,15 EET | control | yes |  | diet_3 | yes | 20,1461 | 0,0156 |  |  |  |  |  |  |  |  |  |
| L_14,15 EET | diet_1 | yes |  | diet_3 | yes | -1,1584 | 0,9999 |  |  |  |  |  |  |  |  |  |
|  |  |  |  |  |  |  |  |  |  |  |  |  |  |  |  |  |
|  |  |  |  |  |  |  |  |  |  |  |  |  |  |  |  |  |
| L_PGB2 | control | no |  | diet_1 | no | -4,44E-16 | 1 |  | L_PGB2 | control | no |  | control | yes | -3,2474 | 0,7889 |
| L_PGB2 | control | no |  | diet_3 | no | -7,11E-15 | 1 |  | L_PGB2 | diet_1 | no |  | diet_1 | yes | -2,9197 | 0,8326 |
| L_PGB2 | diet_1 | no |  | diet_3 | no | -6,66E-15 | 1 |  | L_PGB2 | diet_3 | no |  | diet_3 | yes | 4,20E-15 | 1 |
| L_PGB2 | control | yes |  | diet_1 | yes | 0,3276 | 1 |  |  |  |  |  |  |  |  |  |
| L_PGB2 | control | yes |  | diet_3 | yes | 3,2474 | 0,8078 |  |  |  |  |  |  |  |  |  |
| L_PGB2 | diet_1 | yes |  | diet_3 | yes | 2,9197 | 0,8495 |  |  |  |  |  |  |  |  |  |
|  |  |  |  |  |  |  |  |  |  |  |  |  |  |  |  |  |
|  |  |  |  |  |  |  |  |  |  |  |  |  |  |  |  |  |
| L_LTB4 | control | no |  | diet_1 | no | -28,3827 | 0,0006 |  | L_LTB4 | control | no |  | control | yes | -8,88E-15 | 1 |
| L_LTB4 | control | no |  | diet_3 | no | -24,0722 | 0,0025 |  | L_LTB4 | diet_1 | no |  | diet_1 | yes | 21,6642 | 0,0056 |
| L_LTB4 | diet_1 | no |  | diet_3 | no | 4,3104 | 0,9259 |  | L_LTB4 | diet_3 | no |  | diet_3 | yes | 8,3486 | 0,4948 |
| L_LTB4 | control | yes |  | diet_1 | yes | -6,7185 | 0,6941 |  |  |  |  |  |  |  |  |  |
| L_LTB4 | control | yes |  | diet_3 | yes | -15,7237 | 0,0512 |  |  |  |  |  |  |  |  |  |
| L_LTB4 | diet_1 | yes |  | diet_3 | yes | -9,0052 | 0,4202 |  |  |  |  |  |  |  |  |  |
|  |  |  |  |  |  |  |  |  |  |  |  |  |  |  |  |  |
|  |  |  |  |  |  |  |  |  |  |  |  |  |  |  |  |  |
| L_14,15-DiHETrE | control | no |  | diet_1 | no | 15,1605 | 0,0512 |  | L_14,15-DiHETrE | control | no |  | control | yes | -4,612 | 0,9089 |
| L_14,15-DiHETrE | control | no |  | diet_3 | no | 19,9727 | 0,0089 |  | L_14,15-DiHETrE | diet_1 | no |  | diet_1 | yes | 6,6461 | 0,6832 |
| L_14,15-DiHETrE | diet_1 | no |  | diet_3 | no | 4,8122 | 0,8835 |  | L_14,15-DiHETrE | diet_3 | no |  | diet_3 | yes | 4,2806 | 0,9312 |
| L_14,15-DiHETrE | control | yes |  | diet_1 | yes | 26,4185 | 0,0012 |  |  |  |  |  |  |  |  |  |
| L_14,15-DiHETrE | control | yes |  | diet_3 | yes | 28,8653 | 0,0007 |  |  |  |  |  |  |  |  |  |
| L_14,15-DiHETrE | diet_1 | yes |  | diet_3 | yes | 2,4468 | 0,9937 |  |  |  |  |  |  |  |  |  |

| Compound | Diet | LPS | vs | Diet | LPS | Estimate | P-value |  | Compound | Diet | LPS | vs | Diet | LPS | Estimate | P-value |
| --- | --- | --- | --- | --- | --- | --- | --- | --- | --- | --- | --- | --- | --- | --- | --- | --- |
| L_11,12-DiHETrE | control | no |  | diet_1 | no | 11,448 | 0,2189 |  | L_11,12-DiHETrE | control | no |  | control | yes | -3,9996 | 0,9515 |
| L_11,12-DiHETrE | control | no |  | diet_3 | no | 19,468 | 0,0158 |  | L_11,12-DiHETrE | diet_1 | no |  | diet_1 | yes | 14,5749 | 0,0807 |
| L_11,12-DiHETrE | diet_1 | no |  | diet_3 | no | 8,02 | 0,5445 |  | L_11,12-DiHETrE | diet_3 | no |  | diet_3 | yes | 3,1391 | 0,9823 |
| L_11,12-DiHETrE | control | yes |  | diet_1 | yes | 30,0225 | 0,0007 |  |  |  |  |  |  |  |  |  |
| L_11,12-DiHETrE | control | yes |  | diet_3 | yes | 26,6068 | 0,0022 |  |  |  |  |  |  |  |  |  |
| L_11,12-DiHETrE | diet_1 | yes |  | diet_3 | yes | -3,4157 | 0,9746 |  |  |  |  |  |  |  |  |  |
|  |  |  |  |  |  |  |  |  |  |  |  |  |  |  |  |  |
| L_5,6-DiHETrE | control | no |  | diet_1 | no | 13,5274 | 0,5223 |  | L_5,6-DiHETrE | control | no |  | control | yes | -4,7357 | 0,9879 |
| L_5,6-DiHETrE | control | no |  | diet_3 | no | 14,0986 | 0,4817 |  | L_5,6-DiHETrE | diet_1 | no |  | diet_1 | yes | 9,0232 | 0,8394 |
| L_5,6-DiHETrE | diet_1 | no |  | diet_3 | no | 0,5712 | 1 |  | L_5,6-DiHETrE | diet_3 | no |  | diet_3 | yes | -8,7021 | 0,862 |
| L_5,6-DiHETrE | control | yes |  | diet_1 | yes | 27,2863 | 0,0406 |  |  |  |  |  |  |  |  |  |
| L_5,6-DiHETrE | control | yes |  | diet_3 | yes | 10,1322 | 0,7813 |  |  |  |  |  |  |  |  |  |
| L_5,6-DiHETrE | diet_1 | yes |  | diet_3 | yes | -17,1542 | 0,3022 |  |  |  |  |  |  |  |  |  |
|  |  |  |  |  |  |  |  |  |  |  |  |  |  |  |  |  |
| L_17(S)-HDoHE | control | no |  | diet_1 | no | -1,5948 | 0,2865 |  | L_17(S)-HDoHE | control | no |  | control | yes | 0,2403 | 0,9996 |
| L_17(S)-HDoHE | control | no |  | diet_3 | no | -1,8354 | 0,1584 |  | L_17(S)-HDoHE | diet_1 | no |  | diet_1 | yes | 1,5987 | 0,284 |
| L_17(S)-HDoHE | diet_1 | no |  | diet_3 | no | -0,2406 | 0,9995 |  | L_17(S)-HDoHE | diet_3 | no |  | diet_3 | yes | -0,6786 | 0,9488 |
| L_17(S)-HDoHE | control | yes |  | diet_1 | yes | -0,2364 | 0,9996 |  |  |  |  |  |  |  |  |  |
| L_17(S)-HDoHE | control | yes |  | diet_3 | yes | -2,7543 | 0,0151 |  |  |  |  |  |  |  |  |  |
| L_17(S)-HDoHE | diet_1 | yes |  | diet_3 | yes | -2,5179 | 0,0251 |  |  |  |  |  |  |  |  |  |
|  |  |  |  |  |  |  |  |  |  |  |  |  |  |  |  |  |
| L_13,14-dihydro-15-keto-PGE2 | control | no |  | diet_1 | no | 11,5449 | 0,4337 |  | L_13,14-dihydro-15-keto-PGE2 | control | no |  | control | yes | -3,023 | 0,9952 |
| L_13,14-dihydro-15-keto-PGE2 | control | no |  | diet_3 | no | 12,0125 | 0,3951 |  | L_13,14-dihydro-15-keto-PGE2 | diet_1 | no |  | diet_1 | yes | -2,3908 | 0,9983 |
| L_13,14-dihydro-15-keto-PGE2 | diet_1 | no |  | diet_3 | no | 0,4676 | 1 |  | L_13,14-dihydro-15-keto-PGE2 | diet_3 | no |  | diet_3 | yes | 2,4187 | 0,9983 |
| L_13,14-dihydro-15-keto-PGE2 | control | yes |  | diet_1 | yes | 12,177 | 0,3972 |  |  |  |  |  |  |  |  |  |
| L_13,14-dihydro-15-keto-PGE2 | control | yes |  | diet_3 | yes | 17,4541 | 0,1255 |  |  |  |  |  |  |  |  |  |
| L_13,14-dihydro-15-keto-PGE2 | diet_1 | yes |  | diet_3 | yes | 5,2771 | 0,9463 |  |  |  |  |  |  |  |  |  |

| Compound | Diet | LPS | vs | Diet | LPS | Estimate | P-value |  | Compound | Diet | LPS | vs | Diet | LPS | Estimate | P-value |
| --- | --- | --- | --- | --- | --- | --- | --- | --- | --- | --- | --- | --- | --- | --- | --- | --- |
| L_8-iso-PGF2a | control | no |  | diet_1 | no | -0,02385 | 0,5019 |  | L_8-iso-PGF2a | control | no |  | control | yes | -0,06519 | 0,006 |
| L_8-iso-PGF2a | control | no |  | diet_3 | no | 0,01301 | 0,9139 |  | L_8-iso-PGF2a | diet_1 | no |  | diet_1 | yes | 0,000607 | 1 |
| L_8-iso-PGF2a | diet_1 | no |  | diet_3 | no | 0,03685 | 0,1347 |  | L_8-iso-PGF2a | diet_3 | no |  | diet_3 | yes | -0,03534 | 0,1819 |
| L_8-iso-PGF2a | control | yes |  | diet_1 | yes | 0,04195 | 0,0874 |  |  |  |  |  |  |  |  |  |
| L_8-iso-PGF2a | control | yes |  | diet_3 | yes | 0,04285 | 0,0916 |  |  |  |  |  |  |  |  |  |
| L_8-iso-PGF2a | diet_1 | yes |  | diet_3 | yes | 0,000904 | 1 |  |  |  |  |  |  |  |  |  |
|  |  |  |  |  |  |  |  |  |  |  |  |  |  |  |  |  |
|  |  |  |  |  |  |  |  |  |  |  |  |  |  |  |  |  |
| L_PGF2a | control | no |  | diet_1 | no | 17,0259 | 0,163 |  | L_PGF2a | control | no |  | control | yes | -8,9784 | 0,7393 |
| L_PGF2a | control | no |  | diet_3 | no | 10,3283 | 0,6114 |  | L_PGF2a | diet_1 | no |  | diet_1 | yes | 2,2071 | 0,9992 |
| L_PGF2a | diet_1 | no |  | diet_3 | no | -6,6976 | 0,8945 |  | L_PGF2a | diet_3 | no |  | diet_3 | yes | 5,8277 | 0,9405 |
| L_PGF2a | control | yes |  | diet_1 | yes | 28,2114 | 0,011 |  |  |  |  |  |  |  |  |  |
| L_PGF2a | control | yes |  | diet_3 | yes | 25,1344 | 0,0256 |  |  |  |  |  |  |  |  |  |
| L_PGF2a | diet_1 | yes |  | diet_3 | yes | -3,077 | 0,9963 |  |  |  |  |  |  |  |  |  |
|  |  |  |  |  |  |  |  |  |  |  |  |  |  |  |  |  |
|  |  |  |  |  |  |  |  |  |  |  |  |  |  |  |  |  |
| L_19,20-DiHoPE | control | no |  | diet_1 | no | -4,2849 | 0,1168 |  | L_19,20-DiHoPE | control | no |  | control | yes | -2,666 | 0,528 |
| L_19,20-DiHoPE | control | no |  | diet_3 | no | -5,7481 | 0,0247 |  | L_19,20-DiHoPE | diet_1 | no |  | diet_1 | yes | 0,02362 | 1 |
| L_19,20-DiHoPE | diet_1 | no |  | diet_3 | no | -1,4632 | 0,9142 |  | L_19,20-DiHoPE | diet_3 | no |  | diet_3 | yes | -4,9198 | 0,0666 |
| L_19,20-DiHoPE | control | yes |  | diet_1 | yes | -1,5953 | 0,8909 |  |  |  |  |  |  |  |  |  |
| L_19,20-DiHoPE | control | yes |  | diet_3 | yes | -8,0019 | 0,0033 |  |  |  |  |  |  |  |  |  |
| L_19,20-DiHoPE | diet_1 | yes |  | diet_3 | yes | -6,4066 | 0,014 |  |  |  |  |  |  |  |  |  |
|  |  |  |  |  |  |  |  |  |  |  |  |  |  |  |  |  |
|  |  |  |  |  |  |  |  |  |  |  |  |  |  |  |  |  |
| L_TBXB2 | control | no |  | diet_1 | no | 14,75 | 0,036 |  | L_TBXB2 | control | no |  | control | yes | -6,0714 | 0,8128 |
| L_TBXB2 | control | no |  | diet_3 | no | 17,75 | 0,0067 |  | L_TBXB2 | diet_1 | no |  | diet_1 | yes | 6,125 | 0,784 |
| L_TBXB2 | diet_1 | no |  | diet_3 | no | 3 | 0,9875 |  | L_TBXB2 | diet_3 | no |  | diet_3 | yes | -0,3929 | 1 |
| L_TBXB2 | control | yes |  | diet_1 | yes | 26,9464 | <.0001 |  |  |  |  |  |  |  |  |  |
| L_TBXB2 | control | yes |  | diet_3 | yes | 23,4286 | 0,0005 |  |  |  |  |  |  |  |  |  |
| L_TBXB2 | diet_1 | yes |  | diet_3 | yes | -3,5179 | 0,9783 |  |  |  |  |  |  |  |  |  |

| Compound | Diet | LPS | vs | Diet | LPS | Estimate | P-value |  | Compound | Diet | LPS | vs | Diet | LPS | Estimate | P-value |
| --- | --- | --- | --- | --- | --- | --- | --- | --- | --- | --- | --- | --- | --- | --- | --- | --- |
| L_8,9-DiHETrE | control | no |  | diet_1 | no | 12,9302 | 0,0716 |  | L_8,9-DiHETrE | control | no |  | control | yes | -6,4072 | 0,6326 |
| L_8,9-DiHETrE | control | no |  | diet_3 | no | 27,1829 | 0,0006 |  | L_8,9-DiHETrE | diet_1 | no |  | diet_1 | yes | 6,5769 | 0,5835 |
| L_8,9-DiHETrE | diet_1 | no |  | diet_3 | no | 14,2527 | 0,0437 |  | L_8,9-DiHETrE | diet_3 | no |  | diet_3 | yes | -12,3255 | 0,1019 |
| L_8,9-DiHETrE | control | yes |  | diet_1 | yes | 25,9143 | 0,001 |  |  |  |  |  |  |  |  |  |
| L_8,9-DiHETrE | control | yes |  | diet_3 | yes | 21,2646 | 0,0052 |  |  |  |  |  |  |  |  |  |
| L_8,9-DiHETrE | diet_1 | yes |  | diet_3 | yes | -4,6497 | 0,8536 |  |  |  |  |  |  |  |  |  |
|  |  |  |  |  |  |  |  |  |  |  |  |  |  |  |  |  |
|  |  |  |  |  |  |  |  |  |  |  |  |  |  |  |  |  |
| L_13,14-dihydro-15-keto-PGF2a | control | no |  | diet_1 | no | 17,75 | 0,0002 |  | L_13,14-dihydro-15-keto-PGF2a | control | no |  | control | yes | -7,2857 | 0,3652 |
| L_13,14-dihydro-15-keto-PGF2a | control | no |  | diet_3 | no | 27,75 | <.0001 |  | L_13,14-dihydro-15-keto-PGF2a | diet_1 | no |  | diet_1 | yes | -9,75 | 0,0859 |
| L_13,14-dihydro-15-keto-PGF2a | diet_1 | no |  | diet_3 | no | 10 | 0,0732 |  | L_13,14-dihydro-15-keto-PGF2a | diet_3 | no |  | diet_3 | yes | -12,6071 | 0,0159 |
| L_13,14-dihydro-15-keto-PGF2a | control | yes |  | diet_1 | yes | 15,2857 | 0,002 |  |  |  |  |  |  |  |  |  |
| L_13,14-dihydro-15-keto-PGF2a | control | yes |  | diet_3 | yes | 22,4286 | <.0001 |  |  |  |  |  |  |  |  |  |
| L_13,14-dihydro-15-keto-PGF2a | diet_1 | yes |  | diet_3 | yes | 7,1429 | 0,387 |  |  |  |  |  |  |  |  |  |
|  |  |  |  |  |  |  |  |  |  |  |  |  |  |  |  |  |
|  |  |  |  |  |  |  |  |  |  |  |  |  |  |  |  |  |
| L_lipoxin A4 | control | no |  | diet_1 | no | -0,01691 | 0,9996 |  | L_lipoxin A4 | control | no |  | control | yes | 0,006324 | 1 |
| L_lipoxin A4 | control | no |  | diet_3 | no | -0,157 | 0,0732 |  | L_lipoxin A4 | diet_1 | no |  | diet_1 | yes | 0,1002 | 0,4737 |
| L_lipoxin A4 | diet_1 | no |  | diet_3 | no | -0,1401 | 0,1413 |  | L_lipoxin A4 | diet_3 | no |  | diet_3 | yes | 0,1042 | 0,4683 |
| L_lipoxin A4 | control | yes |  | diet_1 | yes | 0,07695 | 0,7612 |  |  |  |  |  |  |  |  |  |
| L_lipoxin A4 | control | yes |  | diet_3 | yes | -0,05909 | 0,9164 |  |  |  |  |  |  |  |  |  |
| L_lipoxin A4 | diet_1 | yes |  | diet_3 | yes | -0,136 | 0,1917 |  |  |  |  |  |  |  |  |  |

| Compound | Diet | LPS | vs | Diet | LPS | Estimate | P-value |  | Compound | Diet | LPS | vs | Diet | LPS | Estimate | P-value |
| --- | --- | --- | --- | --- | --- | --- | --- | --- | --- | --- | --- | --- | --- | --- | --- | --- |
| L_UK1 | control | no |  | diet_1 | no | 11,9488 | 0,5756 |  | L_UK1 | control | no |  | control | yes | 5,4904 | 0,9703 |
| L_UK1 | control | no |  | diet_3 | no | 23,9488 | 0,0547 |  | L_UK1 | diet_1 | no |  | diet_1 | yes | 4,4429 | 0,9871 |
| L_UK1 | diet_1 | no |  | diet_3 | no | 12 | 0,5716 |  | L_UK1 | diet_3 | no |  | diet_3 | yes | -17,1561 | 0,2508 |
| L_UK1 | control | yes |  | diet_1 | yes | 10,9014 | 0,6743 |  |  |  |  |  |  |  |  |  |
| L_UK1 | control | yes |  | diet_3 | yes | 1,3023 | 1 |  |  |  |  |  |  |  |  |  |
| L_UK1 | diet_1 | yes |  | diet_3 | yes | -9,599 | 0,7714 |  |  |  |  |  |  |  |  |  |
|  |  |  |  |  |  |  |  |  |  |  |  |  |  |  |  |  |
|  |  |  |  |  |  |  |  |  |  |  |  |  |  |  |  |  |
| L_UK2 | control | no |  | diet_1 | no | -4,3194 | 0,9891 |  | L_UK2 | control | no |  | control | yes | -9,8114 | 0,7648 |
| L_UK2 | control | no |  | diet_3 | no | 15,1904 | 0,3586 |  | L_UK2 | diet_1 | no |  | diet_1 | yes | 7,7614 | 0,8832 |
| L_UK2 | diet_1 | no |  | diet_3 | no | 19,5097 | 0,1583 |  | L_UK2 | diet_3 | no |  | diet_3 | yes | -17,1564 | 0,2657 |
| L_UK2 | control | yes |  | diet_1 | yes | 13,2534 | 0,5074 |  |  |  |  |  |  |  |  |  |
| L_UK2 | control | yes |  | diet_3 | yes | 7,8453 | 0,8926 |  |  |  |  |  |  |  |  |  |
| L_UK2 | diet_1 | yes |  | diet_3 | yes | -5,4081 | 0,9732 |  |  |  |  |  |  |  |  |  |
|  |  |  |  |  |  |  |  |  |  |  |  |  |  |  |  |  |
|  |  |  |  |  |  |  |  |  |  |  |  |  |  |  |  |  |
| L_UK3 | control | no |  | diet_1 | no | 0,2655 | 1 |  | L_UK3 | control | no |  | control | yes | -14,8437 | 0,5428 |
| L_UK3 | control | no |  | diet_3 | no | 11,5598 | 0,7509 |  | L_UK3 | diet_1 | no |  | diet_1 | yes | -9,6195 | 0,8605 |
| L_UK3 | diet_1 | no |  | diet_3 | no | 11,2943 | 0,7673 |  | L_UK3 | diet_3 | no |  | diet_3 | yes | -14,213 | 0,5843 |
| L_UK3 | control | yes |  | diet_1 | yes | 5,4897 | 0,9854 |  |  |  |  |  |  |  |  |  |
| L_UK3 | control | yes |  | diet_3 | yes | 12,1905 | 0,724 |  |  |  |  |  |  |  |  |  |
| L_UK3 | diet_1 | yes |  | diet_3 | yes | 6,7008 | 0,9658 |  |  |  |  |  |  |  |  |  |
|  |  |  |  |  |  |  |  |  |  |  |  |  |  |  |  |  |
|  |  |  |  |  |  |  |  |  |  |  |  |  |  |  |  |  |
| L_UK4 | control | no |  | diet_1 | no | -2,9926 | 0,9993 |  | L_UK4 | control | no |  | control | yes | -9,8769 | 0,8791 |
| L_UK4 | control | no |  | diet_3 | no | 9,0941 | 0,906 |  | L_UK4 | diet_1 | no |  | diet_1 | yes | 2,6906 | 0,9996 |
| L_UK4 | diet_1 | no |  | diet_3 | no | 12,0866 | 0,7594 |  | L_UK4 | diet_3 | no |  | diet_3 | yes | -11,7398 | 0,7872 |
| L_UK4 | control | yes |  | diet_1 | yes | 9,575 | 0,8916 |  |  |  |  |  |  |  |  |  |
| L_UK4 | control | yes |  | diet_3 | yes | 7,2312 | 0,965 |  |  |  |  |  |  |  |  |  |
| L_UK4 | diet_1 | yes |  | diet_3 | yes | -2,3438 | 0,9998 |  |  |  |  |  |  |  |  |  |

| Compound | Diet | LPS | vs | Diet | LPS | Estimate | P-value |  | Compound | Diet | LPS | vs | Diet | LPS | Estimate | P-value |
| --- | --- | --- | --- | --- | --- | --- | --- | --- | --- | --- | --- | --- | --- | --- | --- | --- |
| L_UK5 | control | no |  | diet_1 | no | -2,1392 | 0,9997 |  | L_UK5 | control | no |  | control | yes | -9,9904 | 0,7982 |
| L_UK5 | control | no |  | diet_3 | no | 16,7508 | 0,334 |  | L_UK5 | diet_1 | no |  | diet_1 | yes | 2,131 | 0,9997 |
| L_UK5 | diet_1 | no |  | diet_3 | no | 18,89 | 0,2304 |  | L_UK5 | diet_3 | no |  | diet_3 | yes | -19,6155 | 0,2109 |
| L_UK5 | control | yes |  | diet_1 | yes | 9,9822 | 0,7987 |  |  |  |  |  |  |  |  |  |
| L_UK5 | control | yes |  | diet_3 | yes | 7,1257 | 0,9412 |  |  |  |  |  |  |  |  |  |
| L_UK5 | diet_1 | yes |  | diet_3 | yes | -2,8565 | 0,9989 |  |  |  |  |  |  |  |  |  |
|  |  |  |  |  |  |  |  |  |  |  |  |  |  |  |  |  |
|  |  |  |  |  |  |  |  |  |  |  |  |  |  |  |  |  |
| L_epea | control | no |  | diet_1 | no | -1,02E-14 | 1 |  | L_epea | control | no |  | control | yes | -7,99E-15 | 1 |
| L_epea | control | no |  | diet_3 | no | -20,0185 | 0,0003 |  | L_epea | diet_1 | no |  | diet_1 | yes | -23,1182 | <.0001 |
| L_epea | diet_1 | no |  | diet_3 | no | -20,0185 | 0,0003 |  | L_epea | diet_3 | no |  | diet_3 | yes | -6,4473 | 0,3115 |
| L_epea | control | yes |  | diet_1 | yes | -23,1182 | <.0001 |  |  |  |  |  |  |  |  |  |
| L_epea | control | yes |  | diet_3 | yes | -26,4658 | <.0001 |  |  |  |  |  |  |  |  |  |
| L_epea | diet_1 | yes |  | diet_3 | yes | -3,3476 | 0,8551 |  |  |  |  |  |  |  |  |  |
|  |  |  |  |  |  |  |  |  |  |  |  |  |  |  |  |  |
|  |  |  |  |  |  |  |  |  |  |  |  |  |  |  |  |  |
| L_dhea | control | no |  | diet_1 | no | -9,8323 | 0,0902 |  | L_dhea | control | no |  | control | yes | -16,4408 | 0,0035 |
| L_dhea | control | no |  | diet_3 | no | -21,0959 | 0,0004 |  | L_dhea | diet_1 | no |  | diet_1 | yes | -21,5718 | 0,0003 |
| L_dhea | diet_1 | no |  | diet_3 | no | -11,2635 | 0,0438 |  | L_dhea | diet_3 | no |  | diet_3 | yes | -15,3291 | 0,0061 |
| L_dhea | control | yes |  | diet_1 | yes | -14,9634 | 0,0073 |  |  |  |  |  |  |  |  |  |
| L_dhea | control | yes |  | diet_3 | yes | -19,9842 | 0,0008 |  |  |  |  |  |  |  |  |  |
| L_dhea | diet_1 | yes |  | diet_3 | yes | -5,0208 | 0,6621 |  |  |  |  |  |  |  |  |  |
|  |  |  |  |  |  |  |  |  |  |  |  |  |  |  |  |  |
|  |  |  |  |  |  |  |  |  |  |  |  |  |  |  |  |  |
| L_aea | control | no |  | diet_1 | no | 9,4776 | 0,3091 |  | L_aea | control | no |  | control | yes | -24,195 | 0,0018 |
| L_aea | control | no |  | diet_3 | no | -3,8681 | 0,9403 |  | L_aea | diet_1 | no |  | diet_1 | yes | -22,0848 | 0,0029 |
| L_aea | diet_1 | no |  | diet_3 | no | -13,3457 | 0,0799 |  | L_aea | diet_3 | no |  | diet_3 | yes | -9,8014 | 0,3052 |
| L_aea | control | yes |  | diet_1 | yes | 11,5878 | 0,1713 |  |  |  |  |  |  |  |  |  |
| L_aea | control | yes |  | diet_3 | yes | 10,5254 | 0,2667 |  |  |  |  |  |  |  |  |  |
| L_aea | diet_1 | yes |  | diet_3 | yes | -1,0624 | 0,9999 |  |  |  |  |  |  |  |  |  |

| Compound | Diet | LPS | vs | Diet | LPS | Estimate | P-value |  | Compound | Diet | LPS | vs | Diet | LPS | Estimate | P-value |
| --- | --- | --- | --- | --- | --- | --- | --- | --- | --- | --- | --- | --- | --- | --- | --- | --- |
| L_2-ag | control | no |  | diet_1 | no | 18,0607 | 0,0023 |  | L_2-ag | control | no |  | control | yes | 6,6827 | 0,4453 |
| L_2-ag | control | no |  | diet_3 | no | 31,4281 | <.0001 |  | L_2-ag | diet_1 | no |  | diet_1 | yes | 2,4605 | 0,9772 |
| L_2-ag | diet_1 | no |  | diet_3 | no | 13,3674 | 0,0218 |  | L_2-ag | diet_3 | no |  | diet_3 | yes | 3,3045 | 0,928 |
| L_2-ag | control | yes |  | diet_1 | yes | 13,8386 | 0,0188 |  |  |  |  |  |  |  |  |  |
| L_2-ag | control | yes |  | diet_3 | yes | 28,0499 | <.0001 |  |  |  |  |  |  |  |  |  |
| L_2-ag | diet_1 | yes |  | diet_3 | yes | 14,2113 | 0,0157 |  |  |  |  |  |  |  |  |  |
|  |  |  |  |  |  |  |  |  |  |  |  |  |  |  |  |  |
|  |  |  |  |  |  |  |  |  |  |  |  |  |  |  |  |  |
| L_dle | control | no |  | diet_1 | no | 13,9544 | 0,175 |  | L_dle | control | no |  | control | yes | -14,1965 | 0,1853 |
| L_dle | control | no |  | diet_3 | no | 6,1466 | 0,853 |  | L_dle | diet_1 | no |  | diet_1 | yes | -21,465 | 0,0187 |
| L_dle | diet_1 | no |  | diet_3 | no | -7,8078 | 0,6967 |  | L_dle | diet_3 | no |  | diet_3 | yes | -7,2757 | 0,7732 |
| L_dle | control | yes |  | diet_1 | yes | 6,686 | 0,8256 |  |  |  |  |  |  |  |  |  |
| L_dle | control | yes |  | diet_3 | yes | 13,0675 | 0,2741 |  |  |  |  |  |  |  |  |  |
| L_dle | diet_1 | yes |  | diet_3 | yes | 6,3815 | 0,8503 |  |  |  |  |  |  |  |  |  |
|  |  |  |  |  |  |  |  |  |  |  |  |  |  |  |  |  |
|  |  |  |  |  |  |  |  |  |  |  |  |  |  |  |  |  |
| L_pea | control | no |  | diet_1 | no | -17,084 | 0,775 |  | L_pea | control | no |  | control | yes | 2,8174 | 0,9999 |
| L_pea | control | no |  | diet_3 | no | -40,1932 | 0,0804 |  | L_pea | diet_1 | no |  | diet_1 | yes | 33,6966 | 0,1764 |
| L_pea | diet_1 | no |  | diet_3 | no | -23,1092 | 0,5166 |  | L_pea | diet_3 | no |  | diet_3 | yes | 46,6644 | 0,0386 |
| L_pea | control | yes |  | diet_1 | yes | 13,7952 | 0,8944 |  |  |  |  |  |  |  |  |  |
| L_pea | control | yes |  | diet_3 | yes | 3,6538 | 0,9997 |  |  |  |  |  |  |  |  |  |
| L_pea | diet_1 | yes |  | diet_3 | yes | -10,1414 | 0,9683 |  |  |  |  |  |  |  |  |  |
|  |  |  |  |  |  |  |  |  |  |  |  |  |  |  |  |  |
| L_oea | control | no |  | diet_1 | no | 3,6458 | 0,9956 |  | L_oea | control | no |  | control | yes | -48,2253 | <.0001 |
| L_oea | control | no |  | diet_3 | no | 9,3251 | 0,7832 |  | L_oea | diet_1 | no |  | diet_1 | yes | -30,0786 | 0,0019 |
| L_oea | diet_1 | no |  | diet_3 | no | 5,6793 | 0,9673 |  | L_oea | diet_3 | no |  | diet_3 | yes | -30,4911 | 0,0025 |
| L_oea | control | yes |  | diet_1 | yes | 21,7925 | 0,0572 |  |  |  |  |  |  |  |  |  |
| L_oea | control | yes |  | diet_3 | yes | 27,0593 | 0,0127 |  |  |  |  |  |  |  |  |  |
| L_oea | diet_1 | yes |  | diet_3 | yes | 5,2667 | 0,9797 |  |  |  |  |  |  |  |  |  |

| Compound | Diet | LPS | vs | Diet | LPS | Estimate | P-value |  | Compound | Diet | LPS | vs | Diet | LPS | Estimate | P-value |
| --- | --- | --- | --- | --- | --- | --- | --- | --- | --- | --- | --- | --- | --- | --- | --- | --- |
| L_sea | control | no |  | diet_1 | no | -4,5367 | 0,959 |  | L_sea | control | no |  | control | yes | 18,6175 | 0,0555 |
| L_sea | control | no |  | diet_3 | no | -12,2851 | 0,3005 |  | L_sea | diet_1 | no |  | diet_1 | yes | 24,635 | 0,0081 |
| L_sea | diet_1 | no |  | diet_3 | no | -7,7483 | 0,7304 |  | L_sea | diet_3 | no |  | diet_3 | yes | 30,994 | 0,0015 |
| L_sea | control | yes |  | diet_1 | yes | 1,4808 | 0,9998 |  |  |  |  |  |  |  |  |  |
| L_sea | control | yes |  | diet_3 | yes | 0,09142 | 1 |  |  |  |  |  |  |  |  |  |
| L_sea | diet_1 | yes |  | diet_3 | yes | -1,3894 | 0,9998 |  |  |  |  |  |  |  |  |  |

| **Compound** | **Diet** | **LPS** | **vs** | **Diet** | **LPS** | **Estimate** | **P-value** |  | **Compound** | **Diet** | **LPS** | **vs** | **Diet** | **LPS** | **Estimate** | **P-value** |
| --- | --- | --- | --- | --- | --- | --- | --- | --- | --- | --- | --- | --- | --- | --- | --- | --- |
| I_AA | control | no |  | diet_1 | no | 28173 | 0,4301 |  | I_AA | control | no |  | control | yes | -8005,8 | 0,9928 |
| I_AA | control | no |  | diet_3 | no | 17011 | 0,8416 |  | I_AA | diet_1 | no |  | diet_1 | yes | -16262 | 0,8637 |
| I_AA | diet_1 | no |  | diet_3 | no | -11162 | 0,9675 |  | I_AA | diet_3 | no |  | diet_3 | yes | -631,63 | 1 |
| I_AA | control | yes |  | diet_1 | yes | 19916 | 0,752 |  |  |  |  |  |  |  |  |  |
| I_AA | control | yes |  | diet_3 | yes | 24385 | 0,5943 |  |  |  |  |  |  |  |  |  |
| I_AA | diet_1 | yes |  | diet_3 | yes | 4469,22 | 0,9995 |  |  |  |  |  |  |  |  |  |
|  |  |  |  |  |  |  |  |  |  |  |  |  |  |  |  |  |
|  |  |  |  |  |  |  |  |  |  |  |  |  |  |  |  |  |
| I_EPA | control | no |  | diet_1 | no | -16,6361 | 0,0513 |  | I_EPA | control | no |  | control | yes | -1,2562 | 0,9998 |
| I_EPA | control | no |  | diet_3 | no | -28,2924 | 0,0011 |  | I_EPA | diet_1 | no |  | diet_1 | yes | -3,5082 | 0,9774 |
| I_EPA | diet_1 | no |  | diet_3 | no | -11,6563 | 0,2484 |  | I_EPA | diet_3 | no |  | diet_3 | yes | -2,7926 | 0,992 |
| I_EPA | control | yes |  | diet_1 | yes | -18,8881 | 0,0253 |  |  |  |  |  |  |  |  |  |
| I_EPA | control | yes |  | diet_3 | yes | -29,8289 | 0,0008 |  |  |  |  |  |  |  |  |  |
| I_EPA | diet_1 | yes |  | diet_3 | yes | -10,9408 | 0,3108 |  |  |  |  |  |  |  |  |  |
|  |  |  |  |  |  |  |  |  |  |  |  |  |  |  |  |  |
|  |  |  |  |  |  |  |  |  |  |  |  |  |  |  |  |  |
| I_DHA | control | no |  | diet_1 | no | -12,8053 | 0,2647 |  | I_DHA | control | no |  | control | yes | -0,1434 | 1 |
| I_DHA | control | no |  | diet_3 | no | -23,8063 | 0,0109 |  | I_DHA | diet_1 | no |  | diet_1 | yes | -7,1967 | 0,7822 |
| I_DHA | diet_1 | no |  | diet_3 | no | -11,001 | 0,4061 |  | I_DHA | diet_3 | no |  | diet_3 | yes | -6,5336 | 0,8449 |
| I_DHA | control | yes |  | diet_1 | yes | -19,8586 | 0,0377 |  |  |  |  |  |  |  |  |  |
| I_DHA | control | yes |  | diet_3 | yes | -30,1965 | 0,002 |  |  |  |  |  |  |  |  |  |
| I_DHA | diet_1 | yes |  | diet_3 | yes | -10,3379 | 0,4777 |  |  |  |  |  |  |  |  |  |
|  |  |  |  |  |  |  |  |  |  |  |  |  |  |  |  |  |
|  |  |  |  |  |  |  |  |  |  |  |  |  |  |  |  |  |
| I_12(S)-HHTrE | control | no |  | diet_1 | no | 12,1146 | 0,7712 |  | I_12(S)-HHTrE | control | no |  | control | yes | 2,571 | 0,9997 |
| I_12(S)-HHTrE | control | no |  | diet_3 | no | 10,1875 | 0,869 |  | I_12(S)-HHTrE | diet_1 | no |  | diet_1 | yes | -9,6556 | 0,8914 |
| I_12(S)-HHTrE | diet_1 | no |  | diet_3 | no | -1,9271 | 0,9999 |  | I_12(S)-HHTrE | diet_3 | no |  | diet_3 | yes | -18,6699 | 0,392 |
| I_12(S)-HHTrE | control | yes |  | diet_1 | yes | -0,112 | 1 |  |  |  |  |  |  |  |  |  |
| I_12(S)-HHTrE | control | yes |  | diet_3 | yes | -11,0534 | 0,8366 |  |  |  |  |  |  |  |  |  |
| I_12(S)-HHTrE | diet_1 | yes |  | diet_3 | yes | -10,9414 | 0,8378 |  |  |  |  |  |  |  |  |  |

| Compound | Diet | LPS | vs | Diet | LPS | Estimate | P-value |  | Compound | Diet | LPS | vs | Diet | LPS | Estimate | P-value |
| --- | --- | --- | --- | --- | --- | --- | --- | --- | --- | --- | --- | --- | --- | --- | --- | --- |
| I_9(S)-HODE | control | no |  | diet_1 | no | -19,8573 | 1 |  | I_9(S)-HODE | control | no |  | control | yes | -211,37 | 0,714 |
| I_9(S)-HODE | control | no |  | diet_3 | no | 9,7426 | 1 |  | I_9(S)-HODE | diet_1 | no |  | diet_1 | yes | -286,75 | 0,4261 |
| I_9(S)-HODE | diet_1 | no |  | diet_3 | no | 29,5999 | 0,9999 |  | I_9(S)-HODE | diet_3 | no |  | diet_3 | yes | -231,97 | 0,6358 |
| I_9(S)-HODE | control | yes |  | diet_1 | yes | -95,2407 | 0,9851 |  |  |  |  |  |  |  |  |  |
| I_9(S)-HODE | control | yes |  | diet_3 | yes | -10,8558 | 1 |  |  |  |  |  |  |  |  |  |
| I_9(S)-HODE | diet_1 | yes |  | diet_3 | yes | 84,3849 | 0,9913 |  |  |  |  |  |  |  |  |  |
|  |  |  |  |  |  |  |  |  |  |  |  |  |  |  |  |  |
|  |  |  |  |  |  |  |  |  |  |  |  |  |  |  |  |  |
| I_13(S)-HODE | control | no |  | diet_1 | no | 55,0227 | 0,9895 |  | I_13(S)-HODE | control | no |  | control | yes | -92,4212 | 0,9147 |
| I_13(S)-HODE | control | no |  | diet_3 | no | 83,4668 | 0,9391 |  | I_13(S)-HODE | diet_1 | no |  | diet_1 | yes | -270,09 | 0,1121 |
| I_13(S)-HODE | diet_1 | no |  | diet_3 | no | 28,4441 | 0,9995 |  | I_13(S)-HODE | diet_3 | no |  | diet_3 | yes | -161,28 | 0,5517 |
| I_13(S)-HODE | control | yes |  | diet_1 | yes | -122,65 | 0,7785 |  |  |  |  |  |  |  |  |  |
| I_13(S)-HODE | control | yes |  | diet_3 | yes | 14,6098 | 1 |  |  |  |  |  |  |  |  |  |
| I_13(S)-HODE | diet_1 | yes |  | diet_3 | yes | 137,26 | 0,6955 |  |  |  |  |  |  |  |  |  |
|  |  |  |  |  |  |  |  |  |  |  |  |  |  |  |  |  |
|  |  |  |  |  |  |  |  |  |  |  |  |  |  |  |  |  |
| I_15-deoxy-d-12,14-PGJ2 | control | no |  | diet_1 | no | -5,5 | 0,9646 |  | I_15-deoxy-d-12,14-PGJ2 | control | no |  | control | yes | -4,5893 | 0,9862 |
| I_15-deoxy-d-12,14-PGJ2 | control | no |  | diet_3 | no | 4 | 0,9914 |  | I_15-deoxy-d-12,14-PGJ2 | diet_1 | no |  | diet_1 | yes | 9,875 | 0,6975 |
| I_15-deoxy-d-12,14-PGJ2 | diet_1 | no |  | diet_3 | no | 9,5 | 0,7303 |  | I_15-deoxy-d-12,14-PGJ2 | diet_3 | no |  | diet_3 | yes | -5,1607 | 0,9768 |
| I_15-deoxy-d-12,14-PGJ2 | control | yes |  | diet_1 | yes | 8,9643 | 0,7988 |  |  |  |  |  |  |  |  |  |
| I_15-deoxy-d-12,14-PGJ2 | control | yes |  | diet_3 | yes | 3,4286 | 0,9969 |  |  |  |  |  |  |  |  |  |
| I_15-deoxy-d-12,14-PGJ2 | diet_1 | yes |  | diet_3 | yes | -5,5357 | 0,9686 |  |  |  |  |  |  |  |  |  |
|  |  |  |  |  |  |  |  |  |  |  |  |  |  |  |  |  |
|  |  |  |  |  |  |  |  |  |  |  |  |  |  |  |  |  |
| I_12(S)-HEPE | control | no |  | diet_1 | no | -82,4552 | 0,3073 |  | I_12(S)-HEPE | control | no |  | control | yes | 0,3327 | 1 |
| I_12(S)-HEPE | control | no |  | diet_3 | no | -182,67 | 0,0048 |  | I_12(S)-HEPE | diet_1 | no |  | diet_1 | yes | -45,5816 | 0,8194 |
| I_12(S)-HEPE | diet_1 | no |  | diet_3 | no | -100,21 | 0,1562 |  | I_12(S)-HEPE | diet_3 | no |  | diet_3 | yes | -3,2421 | 1 |
| I_12(S)-HEPE | control | yes |  | diet_1 | yes | -128,37 | 0,0522 |  |  |  |  |  |  |  |  |  |
| I_12(S)-HEPE | control | yes |  | diet_3 | yes | -186,24 | 0,0052 |  |  |  |  |  |  |  |  |  |
| I_12(S)-HEPE | diet_1 | yes |  | diet_3 | yes | -57,8715 | 0,6579 |  |  |  |  |  |  |  |  |  |

| Compound | Diet | LPS | vs | Diet | LPS | Estimate | P-value |  | Compound | Diet | LPS | vs | Diet | LPS | Estimate | P-value |
| --- | --- | --- | --- | --- | --- | --- | --- | --- | --- | --- | --- | --- | --- | --- | --- | --- |
| I_5(S)-HEPE | control | no |  | diet_1 | no | -15,375 | 0,0006 |  | I_5(S)-HEPE | control | no |  | control | yes | -0,5357 | 1 |
| I_5(S)-HEPE | control | no |  | diet_3 | no | -28,5 | <.0001 |  | I_5(S)-HEPE | diet_1 | no |  | diet_1 | yes | -5,875 | 0,5118 |
| I_5(S)-HEPE | diet_1 | no |  | diet_3 | no | -13,125 | 0,0046 |  | I_5(S)-HEPE | diet_3 | no |  | diet_3 | yes | -0,03571 | 1 |
| I_5(S)-HEPE | control | yes |  | diet_1 | yes | -20,7143 | <.0001 |  |  |  |  |  |  |  |  |  |
| I_5(S)-HEPE | control | yes |  | diet_3 | yes | -28 | <.0001 |  |  |  |  |  |  |  |  |  |
| I_5(S)-HEPE | diet_1 | yes |  | diet_3 | yes | -7,2857 | 0,313 |  |  |  |  |  |  |  |  |  |
|  |  |  |  |  |  |  |  |  |  |  |  |  |  |  |  |  |
|  |  |  |  |  |  |  |  |  |  |  |  |  |  |  |  |  |
| I_20(S)-HETE | control | no |  | diet_1 | no | -0,2354 | 1 |  | I_20(S)-HETE | control | no |  | control | yes | -2,9553 | 0,5163 |
| I_20(S)-HETE | control | no |  | diet_3 | no | 0,3262 | 0,9999 |  | I_20(S)-HETE | diet_1 | no |  | diet_1 | yes | 0,7951 | 0,9958 |
| I_20(S)-HETE | diet_1 | no |  | diet_3 | no | 0,5617 | 0,9992 |  | I_20(S)-HETE | diet_3 | no |  | diet_3 | yes | -1,8626 | 0,8645 |
| I_20(S)-HETE | control | yes |  | diet_1 | yes | 3,5149 | 0,3471 |  |  |  |  |  |  |  |  |  |
| I_20(S)-HETE | control | yes |  | diet_3 | yes | 1,4189 | 0,9535 |  |  |  |  |  |  |  |  |  |
| I_20(S)-HETE | diet_1 | yes |  | diet_3 | yes | -2,096 | 0,8007 |  |  |  |  |  |  |  |  |  |
|  |  |  |  |  |  |  |  |  |  |  |  |  |  |  |  |  |
|  |  |  |  |  |  |  |  |  |  |  |  |  |  |  |  |  |
| I_15(S)-HETE | control | no |  | diet_1 | no | 42,3628 | 0,8685 |  | I_15(S)-HETE | control | no |  | control | yes | -1,3257 | 1 |
| I_15(S)-HETE | control | no |  | diet_3 | no | 56,5033 | 0,6827 |  | I_15(S)-HETE | diet_1 | no |  | diet_1 | yes | -36,4169 | 0,924 |
| I_15(S)-HETE | diet_1 | no |  | diet_3 | no | 14,1406 | 0,9988 |  | I_15(S)-HETE | diet_3 | no |  | diet_3 | yes | -12,9653 | 0,9992 |
| I_15(S)-HETE | control | yes |  | diet_1 | yes | 7,2716 | 1 |  |  |  |  |  |  |  |  |  |
| I_15(S)-HETE | control | yes |  | diet_3 | yes | 44,8637 | 0,8435 |  |  |  |  |  |  |  |  |  |
| I_15(S)-HETE | diet_1 | yes |  | diet_3 | yes | 37,5921 | 0,9153 |  |  |  |  |  |  |  |  |  |
|  |  |  |  |  |  |  |  |  |  |  |  |  |  |  |  |  |
|  |  |  |  |  |  |  |  |  |  |  |  |  |  |  |  |  |
| I_11(S)-HETE | control | no |  | diet_1 | no | 39,7365 | 0,9506 |  | I_11(S)-HETE | control | no |  | control | yes | -11,918 | 0,9998 |
| I_11(S)-HETE | control | no |  | diet_3 | no | 56,2794 | 0,8245 |  | I_11(S)-HETE | diet_1 | no |  | diet_1 | yes | -45,7947 | 0,9145 |
| I_11(S)-HETE | diet_1 | no |  | diet_3 | no | 16,5429 | 0,999 |  | I_11(S)-HETE | diet_3 | no |  | diet_3 | yes | -22,0649 | 0,9963 |
| I_11(S)-HETE | control | yes |  | diet_1 | yes | 5,8598 | 1 |  |  |  |  |  |  |  |  |  |
| I_11(S)-HETE | control | yes |  | diet_3 | yes | 46,1325 | 0,9135 |  |  |  |  |  |  |  |  |  |
| I_11(S)-HETE | diet_1 | yes |  | diet_3 | yes | 40,2727 | 0,9483 |  |  |  |  |  |  |  |  |  |

| Compound | Diet | LPS | vs | Diet | LPS | Estimate | P-value |  | Compound | Diet | LPS | vs | Diet | LPS | Estimate | P-value |
| --- | --- | --- | --- | --- | --- | --- | --- | --- | --- | --- | --- | --- | --- | --- | --- | --- |
| I_12(S)-HETE | control | no |  | diet_1 | no | 194,01 | 0,5942 |  | I_12(S)-HETE | control | no |  | control | yes | 19,074 | 1 |
| I_12(S)-HETE | control | no |  | diet_3 | no | 216,63 | 0,4879 |  | I_12(S)-HETE | diet_1 | no |  | diet_1 | yes | -114,49 | 0,9201 |
| I_12(S)-HETE | diet_1 | no |  | diet_3 | no | 22,6141 | 1 |  | I_12(S)-HETE | diet_3 | no |  | diet_3 | yes | -28,8705 | 0,9999 |
| I_12(S)-HETE | control | yes |  | diet_1 | yes | 60,4459 | 0,9949 |  |  |  |  |  |  |  |  |  |
| I_12(S)-HETE | control | yes |  | diet_3 | yes | 168,68 | 0,7268 |  |  |  |  |  |  |  |  |  |
| I_12(S)-HETE | diet_1 | yes |  | diet_3 | yes | 108,24 | 0,9374 |  |  |  |  |  |  |  |  |  |
|  |  |  |  |  |  |  |  |  |  |  |  |  |  |  |  |  |
|  |  |  |  |  |  |  |  |  |  |  |  |  |  |  |  |  |
| I_11,12 EET | control | no |  | diet_1 | no | 14,5625 | 0,1443 |  | I_11,12 EET | control | no |  | control | yes | 0,6429 | 1 |
| I_11,12 EET | control | no |  | diet_3 | no | 18,8125 | 0,0266 |  | I_11,12 EET | diet_1 | no |  | diet_1 | yes | -1,4375 | 0,9999 |
| I_11,12 EET | diet_1 | no |  | diet_3 | no | 4,25 | 0,9765 |  | I_11,12 EET | diet_3 | no |  | diet_3 | yes | -0,3125 | 1 |
| I_11,12 EET | control | yes |  | diet_1 | yes | 12,4821 | 0,3171 |  |  |  |  |  |  |  |  |  |
| I_11,12 EET | control | yes |  | diet_3 | yes | 17,8571 | 0,0646 |  |  |  |  |  |  |  |  |  |
| I_11,12 EET | diet_1 | yes |  | diet_3 | yes | 5,375 | 0,9451 |  |  |  |  |  |  |  |  |  |
|  |  |  |  |  |  |  |  |  |  |  |  |  |  |  |  |  |
| I_8,9 EET | control | no |  | diet_1 | no | 4,0871 | 0,9815 |  | I_8,9 EET | control | no |  | control | yes | -0,03038 | 1 |
| I_8,9 EET | control | no |  | diet_3 | no | 19,3784 | 0,0783 |  | I_8,9 EET | diet_1 | no |  | diet_1 | yes | 9,2009 | 0,6668 |
| I_8,9 EET | diet_1 | no |  | diet_3 | no | 15,2913 | 0,2088 |  | I_8,9 EET | diet_3 | no |  | diet_3 | yes | 1,4735 | 0,9999 |
| I_8,9 EET | control | yes |  | diet_1 | yes | 13,3184 | 0,345 |  |  |  |  |  |  |  |  |  |
| I_8,9 EET | control | yes |  | diet_3 | yes | 20,8823 | 0,0692 |  |  |  |  |  |  |  |  |  |
| I_8,9 EET | diet_1 | yes |  | diet_3 | yes | 7,5639 | 0,823 |  |  |  |  |  |  |  |  |  |
|  |  |  |  |  |  |  |  |  |  |  |  |  |  |  |  |  |
|  |  |  |  |  |  |  |  |  |  |  |  |  |  |  |  |  |
| I_5,6 EET | control | no |  | diet_1 | no | 10,9303 | 0,472 |  | I_5,6 EET | control | no |  | control | yes | -7,4446 | 0,8156 |
| I_5,6 EET | control | no |  | diet_3 | no | 7,2777 | 0,8117 |  | I_5,6 EET | diet_1 | no |  | diet_1 | yes | -4,5544 | 0,9668 |
| I_5,6 EET | diet_1 | no |  | diet_3 | no | -3,6527 | 0,9871 |  | I_5,6 EET | diet_3 | no |  | diet_3 | yes | -5,4656 | 0,9385 |
| I_5,6 EET | control | yes |  | diet_1 | yes | 13,8205 | 0,2773 |  |  |  |  |  |  |  |  |  |
| I_5,6 EET | control | yes |  | diet_3 | yes | 9,2567 | 0,68 |  |  |  |  |  |  |  |  |  |
| I_5,6 EET | diet_1 | yes |  | diet_3 | yes | -4,5638 | 0,9704 |  |  |  |  |  |  |  |  |  |

| Compound | Diet | LPS | vs | Diet | LPS | Estimate | P-value |  | Compound | Diet | LPS | vs | Diet | LPS | Estimate | P-value |
| --- | --- | --- | --- | --- | --- | --- | --- | --- | --- | --- | --- | --- | --- | --- | --- | --- |
| I_5(S)-HETE | control | no |  | diet_1 | no | 0,2143 | 0,9938 |  | I_5(S)-HETE | control | no |  | control | yes | -0,379 | 0,9341 |
| I_5(S)-HETE | control | no |  | diet_3 | no | 0,7501 | 0,4852 |  | I_5(S)-HETE | diet_1 | no |  | diet_1 | yes | -1,0658 | 0,1788 |
| I_5(S)-HETE | diet_1 | no |  | diet_3 | no | 0,5359 | 0,7731 |  | I_5(S)-HETE | diet_3 | no |  | diet_3 | yes | -0,3365 | 0,9588 |
| I_5(S)-HETE | control | yes |  | diet_1 | yes | -0,4725 | 0,8541 |  |  |  |  |  |  |  |  |  |
| I_5(S)-HETE | control | yes |  | diet_3 | yes | 0,7927 | 0,4587 |  |  |  |  |  |  |  |  |  |
| I_5(S)-HETE | diet_1 | yes |  | diet_3 | yes | 1,2652 | 0,0923 |  |  |  |  |  |  |  |  |  |
|  |  |  |  |  |  |  |  |  |  |  |  |  |  |  |  |  |
|  |  |  |  |  |  |  |  |  |  |  |  |  |  |  |  |  |
| I_14,15 EET | control | no |  | diet_1 | no | 15,4397 | 0,2404 |  | I_14,15 EET | control | no |  | control | yes | -3,4167 | 0,9941 |
| I_14,15 EET | control | no |  | diet_3 | no | 14,7023 | 0,2802 |  | I_14,15 EET | diet_1 | no |  | diet_1 | yes | -6,2166 | 0,917 |
| I_14,15 EET | diet_1 | no |  | diet_3 | no | -0,7374 | 1 |  | I_14,15 EET | diet_3 | no |  | diet_3 | yes | -3,517 | 0,9933 |
| I_14,15 EET | control | yes |  | diet_1 | yes | 12,6398 | 0,4461 |  |  |  |  |  |  |  |  |  |
| I_14,15 EET | control | yes |  | diet_3 | yes | 14,602 | 0,3366 |  |  |  |  |  |  |  |  |  |
| I_14,15 EET | diet_1 | yes |  | diet_3 | yes | 1,9622 | 0,9996 |  |  |  |  |  |  |  |  |  |
|  |  |  |  |  |  |  |  |  |  |  |  |  |  |  |  |  |
|  |  |  |  |  |  |  |  |  |  |  |  |  |  |  |  |  |
| I_LTB4 | control | no |  | diet_1 | no | 18,5 | 0,0276 |  | I_LTB4 | control | no |  | control | yes | 8,5 | 0,7058 |
| I_LTB4 | control | no |  | diet_3 | no | 21 | 0,0086 |  | I_LTB4 | diet_1 | no |  | diet_1 | yes | -1,875 | 0,9995 |
| I_LTB4 | diet_1 | no |  | diet_3 | no | 2,5 | 0,9978 |  | I_LTB4 | diet_3 | no |  | diet_3 | yes | -1,6429 | 0,9998 |
| I_LTB4 | control | yes |  | diet_1 | yes | 8,125 | 0,7431 |  |  |  |  |  |  |  |  |  |
| I_LTB4 | control | yes |  | diet_3 | yes | 10,8571 | 0,4927 |  |  |  |  |  |  |  |  |  |
| I_LTB4 | diet_1 | yes |  | diet_3 | yes | 2,7321 | 0,9972 |  |  |  |  |  |  |  |  |  |
|  |  |  |  |  |  |  |  |  |  |  |  |  |  |  |  |  |
|  |  |  |  |  |  |  |  |  |  |  |  |  |  |  |  |  |
| I_14,15-DiHETrE | control | no |  | diet_1 | no | 11,9018 | 0,1567 |  | I_14,15-DiHETrE | control | no |  | control | yes | 0,9812 | 0,9999 |
| I_14,15-DiHETrE | control | no |  | diet_3 | no | 23,7694 | 0,0028 |  | I_14,15-DiHETrE | diet_1 | no |  | diet_1 | yes | 13,2865 | 0,0981 |
| I_14,15-DiHETrE | diet_1 | no |  | diet_3 | no | 11,8677 | 0,1585 |  | I_14,15-DiHETrE | diet_3 | no |  | diet_3 | yes | 0,8721 | 1 |
| I_14,15-DiHETrE | control | yes |  | diet_1 | yes | 24,207 | 0,003 |  |  |  |  |  |  |  |  |  |
| I_14,15-DiHETrE | control | yes |  | diet_3 | yes | 23,6603 | 0,0044 |  |  |  |  |  |  |  |  |  |
| I_14,15-DiHETrE | diet_1 | yes |  | diet_3 | yes | -0,5467 | 1 |  |  |  |  |  |  |  |  |  |

| Compound | Diet | LPS | vs | Diet | LPS | Estimate | P-value |  | Compound | Diet | LPS | vs | Diet | LPS | Estimate | P-value |
| --- | --- | --- | --- | --- | --- | --- | --- | --- | --- | --- | --- | --- | --- | --- | --- | --- |
| I_11,12-DiHETrE | control | no |  | diet_1 | no | 9,7718 | 0,4368 |  | I_11,12-DiHETrE | control | no |  | control | yes | -3,4519 | 0,982 |
| I_11,12-DiHETrE | control | no |  | diet_3 | no | 24,3776 | 0,0061 |  | I_11,12-DiHETrE | diet_1 | no |  | diet_1 | yes | 11,5622 | 0,2805 |
| I_11,12-DiHETrE | diet_1 | no |  | diet_3 | no | 14,6058 | 0,1182 |  | I_11,12-DiHETrE | diet_3 | no |  | diet_3 | yes | -6,1621 | 0,8319 |
| I_11,12-DiHETrE | control | yes |  | diet_1 | yes | 24,786 | 0,0062 |  |  |  |  |  |  |  |  |  |
| I_11,12-DiHETrE | control | yes |  | diet_3 | yes | 21,6674 | 0,0177 |  |  |  |  |  |  |  |  |  |
| I_11,12-DiHETrE | diet_1 | yes |  | diet_3 | yes | -3,1185 | 0,9885 |  |  |  |  |  |  |  |  |  |
|  |  |  |  |  |  |  |  |  |  |  |  |  |  |  |  |  |
|  |  |  |  |  |  |  |  |  |  |  |  |  |  |  |  |  |
| I_5,6-DiHETrE | control | no |  | diet_1 | no | 4,7459 | 0,9591 |  | I_5,6-DiHETrE | control | no |  | control | yes | -2,8655 | 0,9959 |
| I_5,6-DiHETrE | control | no |  | diet_3 | no | 20,311 | 0,0424 |  | I_5,6-DiHETrE | diet_1 | no |  | diet_1 | yes | 15,7661 | 0,148 |
| I_5,6-DiHETrE | diet_1 | no |  | diet_3 | no | 15,5651 | 0,156 |  | I_5,6-DiHETrE | diet_3 | no |  | diet_3 | yes | -1,5225 | 0,9998 |
| I_5,6-DiHETrE | control | yes |  | diet_1 | yes | 23,3775 | 0,0198 |  |  |  |  |  |  |  |  |  |
| I_5,6-DiHETrE | control | yes |  | diet_3 | yes | 21,6539 | 0,0352 |  |  |  |  |  |  |  |  |  |
| I_5,6-DiHETrE | diet_1 | yes |  | diet_3 | yes | -1,7236 | 0,9996 |  |  |  |  |  |  |  |  |  |
|  |  |  |  |  |  |  |  |  |  |  |  |  |  |  |  |  |
|  |  |  |  |  |  |  |  |  |  |  |  |  |  |  |  |  |
| I_17(S)-HDoHE | control | no |  | diet_1 | no | -13,2117 | 0,3516 |  | I_17(S)-HDoHE | control | no |  | control | yes | 0,2166 | 1 |
| I_17(S)-HDoHE | control | no |  | diet_3 | no | -18,5745 | 0,1013 |  | I_17(S)-HDoHE | diet_1 | no |  | diet_1 | yes | -12,4277 | 0,4107 |
| I_17(S)-HDoHE | diet_1 | no |  | diet_3 | no | -5,3628 | 0,9508 |  | I_17(S)-HDoHE | diet_3 | no |  | diet_3 | yes | -4,8245 | 0,9694 |
| I_17(S)-HDoHE | control | yes |  | diet_1 | yes | -25,8559 | 0,0165 |  |  |  |  |  |  |  |  |  |
| I_17(S)-HDoHE | control | yes |  | diet_3 | yes | -23,6156 | 0,0314 |  |  |  |  |  |  |  |  |  |
| I_17(S)-HDoHE | diet_1 | yes |  | diet_3 | yes | 2,2403 | 0,9991 |  |  |  |  |  |  |  |  |  |

| Compound | Diet | LPS | vs | Diet | LPS | Estimate | P-value |  | Compound | Diet | LPS | vs | Diet | LPS | Estimate | P-value |
| --- | --- | --- | --- | --- | --- | --- | --- | --- | --- | --- | --- | --- | --- | --- | --- | --- |
| I_PGE3 | control | no |  | diet_1 | no | -15,3436 | 0,0342 |  | I_PGE3 | control | no |  | control | yes | -4,8412 | 0,859 |
| I_PGE3 | control | no |  | diet_3 | no | -19,7055 | 0,0063 |  | I_PGE3 | diet_1 | no |  | diet_1 | yes | -13,091 | 0,0818 |
| I_PGE3 | diet_1 | no |  | diet_3 | no | -4,3619 | 0,9011 |  | I_PGE3 | diet_3 | no |  | diet_3 | yes | -15,6929 | 0,0305 |
| I_PGE3 | control | yes |  | diet_1 | yes | -23,5935 | 0,0015 |  |  |  |  |  |  |  |  |  |
| I_PGE3 | control | yes |  | diet_3 | yes | -30,5572 | 0,0002 |  |  |  |  |  |  |  |  |  |
| I_PGE3 | diet_1 | yes |  | diet_3 | yes | -6,9637 | 0,5977 |  |  |  |  |  |  |  |  |  |
|  |  |  |  |  |  |  |  |  |  |  |  |  |  |  |  |  |
|  |  |  |  |  |  |  |  |  |  |  |  |  |  |  |  |  |
| I_PGD3 | control | no |  | diet_1 | no | -16,1572 | 0,0822 |  | I_PGD3 | control | no |  | control | yes | 0,3184 | 1 |
| I_PGD3 | control | no |  | diet_3 | no | -23,113 | 0,0096 |  | I_PGD3 | diet_1 | no |  | diet_1 | yes | -11,2038 | 0,3328 |
| I_PGD3 | diet_1 | no |  | diet_3 | no | -6,9558 | 0,7638 |  | I_PGD3 | diet_3 | no |  | diet_3 | yes | 2,9792 | 0,9917 |
| I_PGD3 | control | yes |  | diet_1 | yes | -27,6793 | 0,0028 |  |  |  |  |  |  |  |  |  |
| I_PGD3 | control | yes |  | diet_3 | yes | -20,4522 | 0,026 |  |  |  |  |  |  |  |  |  |
| I_PGD3 | diet_1 | yes |  | diet_3 | yes | 7,2271 | 0,7466 |  |  |  |  |  |  |  |  |  |
|  |  |  |  |  |  |  |  |  |  |  |  |  |  |  |  |  |
|  |  |  |  |  |  |  |  |  |  |  |  |  |  |  |  |  |
| I_13,14-dihydro-15-keto-PGD2 | control | no |  | diet_1 | no | 14,125 | 0,0831 |  | I_13,14-dihydro-15-keto-PGD2 | control | no |  | control | yes | -6 | 0,8625 |
| I_13,14-dihydro-15-keto-PGD2 | control | no |  | diet_3 | no | 20,125 | 0,0039 |  | I_13,14-dihydro-15-keto-PGD2 | diet_1 | no |  | diet_1 | yes | -8,625 | 0,5441 |
| I_13,14-dihydro-15-keto-PGD2 | diet_1 | no |  | diet_3 | no | 6 | 0,8446 |  | I_13,14-dihydro-15-keto-PGD2 | diet_3 | no |  | diet_3 | yes | -3,6964 | 0,9807 |
| I_13,14-dihydro-15-keto-PGD2 | control | yes |  | diet_1 | yes | 11,5 | 0,269 |  |  |  |  |  |  |  |  |  |
| I_13,14-dihydro-15-keto-PGD2 | control | yes |  | diet_3 | yes | 22,4286 | 0,0024 |  |  |  |  |  |  |  |  |  |
| I_13,14-dihydro-15-keto-PGD2 | diet_1 | yes |  | diet_3 | yes | 10,9286 | 0,3218 |  |  |  |  |  |  |  |  |  |

| Compound | Diet | LPS | vs | Diet | LPS | Estimate | P-value |  | Compound | Diet | LPS | vs | Diet | LPS | Estimate | P-value |
| --- | --- | --- | --- | --- | --- | --- | --- | --- | --- | --- | --- | --- | --- | --- | --- | --- |
| I_13,14-dihydro-15-keto-PGE2 | control | no |  | diet_1 | no | 20 | 0,0004 |  | I_13,14-dihydro-15-keto-PGE2 | control | no |  | control | yes | -2,75 | 0,9884 |
| I_13,14-dihydro-15-keto-PGE2 | control | no |  | diet_3 | no | 22,5 | <.0001 |  | I_13,14-dihydro-15-keto-PGE2 | diet_1 | no |  | diet_1 | yes | -8,625 | 0,3435 |
| I_13,14-dihydro-15-keto-PGE2 | diet_1 | no |  | diet_3 | no | 2,5 | 0,9912 |  | I_13,14-dihydro-15-keto-PGE2 | diet_3 | no |  | diet_3 | yes | 2,4643 | 0,993 |
| I_13,14-dihydro-15-keto-PGE2 | control | yes |  | diet_1 | yes | 14,125 | 0,029 |  |  |  |  |  |  |  |  |  |
| I_13,14-dihydro-15-keto-PGE2 | control | yes |  | diet_3 | yes | 27,7143 | <.0001 |  |  |  |  |  |  |  |  |  |
| I_13,14-dihydro-15-keto-PGE2 | diet_1 | yes |  | diet_3 | yes | 13,5893 | 0,0393 |  |  |  |  |  |  |  |  |  |
|  |  |  |  |  |  |  |  |  |  |  |  |  |  |  |  |  |
|  |  |  |  |  |  |  |  |  |  |  |  |  |  |  |  |  |
| I_PGF2b | control | no |  | diet_1 | no | 1,7019 | 0,9018 |  | I_PGF2b | control | no |  | control | yes | 0,2094 | 1 |
| I_PGF2b | control | no |  | diet_3 | no | 1,1266 | 0,9813 |  | I_PGF2b | diet_1 | no |  | diet_1 | yes | -0,9695 | 0,9904 |
| I_PGF2b | diet_1 | no |  | diet_3 | no | -0,5752 | 0,9992 |  | I_PGF2b | diet_3 | no |  | diet_3 | yes | -0,8637 | 0,9944 |
| I_PGF2b | control | yes |  | diet_1 | yes | 0,5229 | 0,9995 |  |  |  |  |  |  |  |  |  |
| I_PGF2b | control | yes |  | diet_3 | yes | 0,05349 | 1 |  |  |  |  |  |  |  |  |  |
| I_PGF2b | diet_1 | yes |  | diet_3 | yes | -0,4695 | 0,9997 |  |  |  |  |  |  |  |  |  |
|  |  |  |  |  |  |  |  |  |  |  |  |  |  |  |  |  |
|  |  |  |  |  |  |  |  |  |  |  |  |  |  |  |  |  |
| I_8-iso-PGF2a | control | no |  | diet_1 | no | 15,8031 | 0,3755 |  | I_8-iso-PGF2a | control | no |  | control | yes | -2,1928 | 0,9997 |
| I_8-iso-PGF2a | control | no |  | diet_3 | no | 12,422 | 0,608 |  | I_8-iso-PGF2a | diet_1 | no |  | diet_1 | yes | -14,8746 | 0,4343 |
| I_8-iso-PGF2a | diet_1 | no |  | diet_3 | no | -3,381 | 0,9973 |  | I_8-iso-PGF2a | diet_3 | no |  | diet_3 | yes | -0,286 | 1 |
| I_8-iso-PGF2a | control | yes |  | diet_1 | yes | 3,1212 | 0,9983 |  |  |  |  |  |  |  |  |  |
| I_8-iso-PGF2a | control | yes |  | diet_3 | yes | 14,3288 | 0,5023 |  |  |  |  |  |  |  |  |  |
| I_8-iso-PGF2a | diet_1 | yes |  | diet_3 | yes | 11,2075 | 0,7099 |  |  |  |  |  |  |  |  |  |

| Compound | Diet | LPS | vs | Diet | LPS | Estimate | P-value |  | Compound | Diet | LPS | vs | Diet | LPS | Estimate | P-value |
| --- | --- | --- | --- | --- | --- | --- | --- | --- | --- | --- | --- | --- | --- | --- | --- | --- |
| I_PGF2a | control | no |  | diet_1 | no | 16,9124 | 0,335 |  | I_PGF2a | control | no |  | control | yes | 3,1772 | 0,9984 |
| I_PGF2a | control | no |  | diet_3 | no | 13,3175 | 0,5657 |  | I_PGF2a | diet_1 | no |  | diet_1 | yes | -11,0212 | 0,7289 |
| I_PGF2a | diet_1 | no |  | diet_3 | no | -3,5949 | 0,9968 |  | I_PGF2a | diet_3 | no |  | diet_3 | yes | 2,5493 | 0,9994 |
| I_PGF2a | control | yes |  | diet_1 | yes | 2,714 | 0,9992 |  |  |  |  |  |  |  |  |  |
| I_PGF2a | control | yes |  | diet_3 | yes | 12,6895 | 0,6404 |  |  |  |  |  |  |  |  |  |
| I_PGF2a | diet_1 | yes |  | diet_3 | yes | 9,9755 | 0,8081 |  |  |  |  |  |  |  |  |  |
|  |  |  |  |  |  |  |  |  |  |  |  |  |  |  |  |  |
|  |  |  |  |  |  |  |  |  |  |  |  |  |  |  |  |  |
| I_10(S)-17(S)-DiHDoHE | control | no |  | diet_1 | no | -1,3768 | 0,8378 |  | I_10(S)-17(S)-DiHDoHE | control | no |  | control | yes | 0,5337 | 0,9976 |
| I_10(S)-17(S)-DiHDoHE | control | no |  | diet_3 | no | -2,9228 | 0,1392 |  | I_10(S)-17(S)-DiHDoHE | diet_1 | no |  | diet_1 | yes | -1,5453 | 0,7621 |
| I_10(S)-17(S)-DiHDoHE | diet_1 | no |  | diet_3 | no | -1,546 | 0,7617 |  | I_10(S)-17(S)-DiHDoHE | diet_3 | no |  | diet_3 | yes | -1,024 | 0,9544 |
| I_10(S)-17(S)-DiHDoHE | control | yes |  | diet_1 | yes | -3,4557 | 0,0634 |  |  |  |  |  |  |  |  |  |
| I_10(S)-17(S)-DiHDoHE | control | yes |  | diet_3 | yes | -4,4804 | 0,0096 |  |  |  |  |  |  |  |  |  |
| I_10(S)-17(S)-DiHDoHE | diet_1 | yes |  | diet_3 | yes | -1,0247 | 0,9543 |  |  |  |  |  |  |  |  |  |
|  |  |  |  |  |  |  |  |  |  |  |  |  |  |  |  |  |
|  |  |  |  |  |  |  |  |  |  |  |  |  |  |  |  |  |
| I_19,20-DiHoPE | control | no |  | diet_1 | no | -18,9371 | 0,0052 |  | I_19,20-DiHoPE | control | no |  | control | yes | -5,2806 | 0,7653 |
| I_19,20-DiHoPE | control | no |  | diet_3 | no | -19,2797 | 0,0046 |  | I_19,20-DiHoPE | diet_1 | no |  | diet_1 | yes | -3,3951 | 0,9442 |
| I_19,20-DiHoPE | diet_1 | no |  | diet_3 | no | -0,3426 | 1 |  | I_19,20-DiHoPE | diet_3 | no |  | diet_3 | yes | -16,6623 | 0,0153 |
| I_19,20-DiHoPE | control | yes |  | diet_1 | yes | -17,0516 | 0,0132 |  |  |  |  |  |  |  |  |  |
| I_19,20-DiHoPE | control | yes |  | diet_3 | yes | -30,6615 | 0,0002 |  |  |  |  |  |  |  |  |  |
| I_19,20-DiHoPE | diet_1 | yes |  | diet_3 | yes | -13,6098 | 0,051 |  |  |  |  |  |  |  |  |  |

| Compound | Diet | LPS | vs | Diet | LPS | Estimate | P-value |  | Compound | Diet | LPS | vs | Diet | LPS | Estimate | P-value |
| --- | --- | --- | --- | --- | --- | --- | --- | --- | --- | --- | --- | --- | --- | --- | --- | --- |
| I_TBXB3 | control | no |  | diet_1 | no | -15,724 | 0,0853 |  | I_TBXB3 | control | no |  | control | yes | -6,3778 | 0,8133 |
| I_TBXB3 | control | no |  | diet_3 | no | -23,6967 | 0,0072 |  | I_TBXB3 | diet_1 | no |  | diet_1 | yes | -16,2216 | 0,0732 |
| I_TBXB3 | diet_1 | no |  | diet_3 | no | -7,9727 | 0,6365 |  | I_TBXB3 | diet_3 | no |  | diet_3 | yes | -2,6413 | 0,9946 |
| I_TBXB3 | control | yes |  | diet_1 | yes | -25,5679 | 0,0046 |  |  |  |  |  |  |  |  |  |
| I_TBXB3 | control | yes |  | diet_3 | yes | -19,9602 | 0,0271 |  |  |  |  |  |  |  |  |  |
| I_TBXB3 | diet_1 | yes |  | diet_3 | yes | 5,6076 | 0,8783 |  |  |  |  |  |  |  |  |  |
|  |  |  |  |  |  |  |  |  |  |  |  |  |  |  |  |  |
| I_TBXB2 | control | no |  | diet_1 | no | 13,271 | 0,9838 |  | I_TBXB2 | control | no |  | control | yes | -55,4999 | 0,1565 |
| I_TBXB2 | control | no |  | diet_3 | no | 22,6153 | 0,8668 |  | I_TBXB2 | diet_1 | no |  | diet_1 | yes | -49,5195 | 0,2283 |
| I_TBXB2 | diet_1 | no |  | diet_3 | no | 9,3443 | 0,9967 |  | I_TBXB2 | diet_3 | no |  | diet_3 | yes | -22,2243 | 0,8795 |
| I_TBXB2 | control | yes |  | diet_1 | yes | 19,2514 | 0,9286 |  |  |  |  |  |  |  |  |  |
| I_TBXB2 | control | yes |  | diet_3 | yes | 55,8909 | 0,1591 |  |  |  |  |  |  |  |  |  |
| I_TBXB2 | diet_1 | yes |  | diet_3 | yes | 36,6395 | 0,5156 |  |  |  |  |  |  |  |  |  |
|  |  |  |  |  |  |  |  |  |  |  |  |  |  |  |  |  |
| I_8,9-DiHETrE | control | no |  | diet_1 | no | 8,3375 | 0,6925 |  | I_8,9-DiHETrE | control | no |  | control | yes | -5,4056 | 0,931 |
| I_8,9-DiHETrE | control | no |  | diet_3 | no | 22,4613 | 0,0201 |  | I_8,9-DiHETrE | diet_1 | no |  | diet_1 | yes | 9,3206 | 0,5945 |
| I_8,9-DiHETrE | diet_1 | no |  | diet_3 | no | 14,1239 | 0,2085 |  | I_8,9-DiHETrE | diet_3 | no |  | diet_3 | yes | -9,1541 | 0,6297 |
| I_8,9-DiHETrE | control | yes |  | diet_1 | yes | 23,0636 | 0,0193 |  |  |  |  |  |  |  |  |  |
| I_8,9-DiHETrE | control | yes |  | diet_3 | yes | 18,7129 | 0,073 |  |  |  |  |  |  |  |  |  |
| I_8,9-DiHETrE | diet_1 | yes |  | diet_3 | yes | -4,3508 | 0,9712 |  |  |  |  |  |  |  |  |  |
|  |  |  |  |  |  |  |  |  |  |  |  |  |  |  |  |  |
| I_13,14-dihydro-15-keto-PGF2a | control | no |  | diet_1 | no | 22,5 | 0,0095 |  | I_13,14-dihydro-15-keto-PGF2a | control | no |  | control | yes | 1,0479 | 0,9999 |
| I_13,14-dihydro-15-keto-PGF2a | control | no |  | diet_3 | no | 26,5368 | 0,0028 |  |  |  |  |  |  |  |  |  |
| I_13,14-dihydro-15-keto-PGF2a | diet_1 | no |  | diet_3 | no | 4,0368 | 0,9618 |  | I_13,14-dihydro-15-keto-PGF2a | diet_3 | no |  | diet_3 | yes | -7,1192 | 0,7407 |
| I_13,14-dihydro-15-keto-PGF2a | control | yes |  | diet_1 | yes | 15,4313 | 0,0995 |  |  |  |  |  |  |  |  |  |
| I_13,14-dihydro-15-keto-PGF2a | control | yes |  | diet_3 | yes | 18,3697 | 0,0463 |  |  |  |  |  |  |  |  |  |
| I_13,14-dihydro-15-keto-PGF2a | diet_1 | yes |  | diet_3 | yes | 2,9384 | 0,9913 |  |  |  |  |  |  |  |  |  |

| Compound | Diet | LPS | vs | Diet | LPS | Estimate | P-value |  | Compound | Diet | LPS | vs | Diet | LPS | Estimate | P-value |
| --- | --- | --- | --- | --- | --- | --- | --- | --- | --- | --- | --- | --- | --- | --- | --- | --- |
| I_lipoxin A4 | control | no |  | diet_1 | no | -7,9234 | 0,7581 |  | I_lipoxin A4 | control | no |  | control | yes | -5,9703 | 0,9145 |
| I_lipoxin A4 | control | no |  | diet_3 | no | -19,6822 | 0,0583 |  | I_lipoxin A4 | diet_1 | no |  | diet_1 | yes | 9,815 | 0,5803 |
| I_lipoxin A4 | diet_1 | no |  | diet_3 | no | -11,7588 | 0,4054 |  | I_lipoxin A4 | diet_3 | no |  | diet_3 | yes | 1,4795 | 0,9998 |
| I_lipoxin A4 | control | yes |  | diet_1 | yes | 7,8619 | 0,7834 |  |  |  |  |  |  |  |  |  |
| I_lipoxin A4 | control | yes |  | diet_3 | yes | -12,2324 | 0,4228 |  |  |  |  |  |  |  |  |  |
| I_lipoxin A4 | diet_1 | yes |  | diet_3 | yes | -20,0942 | 0,061 |  |  |  |  |  |  |  |  |  |
|  |  |  |  |  |  |  |  |  |  |  |  |  |  |  |  |  |
|  |  |  |  |  |  |  |  |  |  |  |  |  |  |  |  |  |
| I_PGE2 | control | no |  | diet_1 | no | 286,12 | 0,8026 |  | I_PGE2 | control | no |  | control | yes | -243,9 | 0,8853 |
| I_PGE2 | control | no |  | diet_3 | no | 299,18 | 0,7737 |  | I_PGE2 | diet_1 | no |  | diet_1 | yes | -365,42 | 0,6124 |
| I_PGE2 | diet_1 | no |  | diet_3 | no | 13,0603 | 1 |  | I_PGE2 | diet_3 | no |  | diet_3 | yes | -181,19 | 0,9635 |
| I_PGE2 | control | yes |  | diet_1 | yes | 164,6 | 0,9756 |  |  |  |  |  |  |  |  |  |
| I_PGE2 | control | yes |  | diet_3 | yes | 361,88 | 0,6264 |  |  |  |  |  |  |  |  |  |
| I_PGE2 | diet_1 | yes |  | diet_3 | yes | 197,29 | 0,9484 |  |  |  |  |  |  |  |  |  |
|  |  |  |  |  |  |  |  |  |  |  |  |  |  |  |  |  |
|  |  |  |  |  |  |  |  |  |  |  |  |  |  |  |  |  |
| I_PGD2 | control | no |  | diet_1 | no | 4,3157 | 0,988 |  | I_PGD2 | control | no |  | control | yes | -10,4582 | 0,6983 |
| I_PGD2 | control | no |  | diet_3 | no | 8,3549 | 0,8368 |  | I_PGD2 | diet_1 | no |  | diet_1 | yes | -14,604 | 0,3682 |
| I_PGD2 | diet_1 | no |  | diet_3 | no | 4,0392 | 0,9911 |  | I_PGD2 | diet_3 | no |  | diet_3 | yes | -0,00395 | 1 |
| I_PGD2 | control | yes |  | diet_1 | yes | 0,1699 | 1 |  |  |  |  |  |  |  |  |  |
| I_PGD2 | control | yes |  | diet_3 | yes | 18,8091 | 0,1854 |  |  |  |  |  |  |  |  |  |
| I_PGD2 | diet_1 | yes |  | diet_3 | yes | 18,6393 | 0,1793 |  |  |  |  |  |  |  |  |  |
|  |  |  |  |  |  |  |  |  |  |  |  |  |  |  |  |  |
|  |  |  |  |  |  |  |  |  |  |  |  |  |  |  |  |  |
| I_Leukotriene E4 | control | no |  | diet_1 | no | 11,3916 | 0,6479 |  | I_Leukotriene E4 | control | no |  | control | yes | -5,5056 | 0,9731 |
| I_Leukotriene E4 | control | no |  | diet_3 | no | 11,3916 | 0,6479 |  | I_Leukotriene E4 | diet_1 | no |  | diet_1 | yes | 0 | 1 |
| I_Leukotriene E4 | diet_1 | no |  | diet_3 | no | 1,42E-14 | 1 |  | I_Leukotriene E4 | diet_3 | no |  | diet_3 | yes | -8,6066 | 0,8506 |
| I_Leukotriene E4 | control | yes |  | diet_1 | yes | 16,8971 | 0,2819 |  |  |  |  |  |  |  |  |  |
| I_Leukotriene E4 | control | yes |  | diet_3 | yes | 8,2905 | 0,8732 |  |  |  |  |  |  |  |  |  |
| I_Leukotriene E4 | diet_1 | yes |  | diet_3 | yes | -8,6066 | 0,8506 |  |  |  |  |  |  |  |  |  |

| Compound | Diet | LPS | vs | Diet | LPS | Estimate | P-value |  | Compound | Diet | LPS | vs | Diet | LPS | Estimate | P-value |
| --- | --- | --- | --- | --- | --- | --- | --- | --- | --- | --- | --- | --- | --- | --- | --- | --- |
| I_n-acetyl leukotriene E4 | control | no |  | diet_1 | no | 0 | 1 |  | I_n-acetyl leukotriene E4 | control | no |  | control | yes | -17,8571 | <.0001 |
| I_n-acetyl leukotriene E4 | control | no |  | diet_3 | no | 3,55E-15 | 1 |  | I_n-acetyl leukotriene E4 | diet_1 | no |  | diet_1 | yes | -5 | 0,6394 |
| I_n-acetyl leukotriene E4 | diet_1 | no |  | diet_3 | no | 3,55E-15 | 1 |  | I_n-acetyl leukotriene E4 | diet_3 | no |  | diet_3 | yes | -22,4286 | <.0001 |
| I_n-acetyl leukotriene E4 | control | yes |  | diet_1 | yes | 12,8571 | 0,0055 |  |  |  |  |  |  |  |  |  |
| I_n-acetyl leukotriene E4 | control | yes |  | diet_3 | yes | -4,5714 | 0,7726 |  |  |  |  |  |  |  |  |  |
| I_n-acetyl leukotriene E4 | diet_1 | yes |  | diet_3 | yes | -17,4286 | <.0001 |  |  |  |  |  |  |  |  |  |
|  |  |  |  |  |  |  |  |  |  |  |  |  |  |  |  |  |
|  |  |  |  |  |  |  |  |  |  |  |  |  |  |  |  |  |
| I_Leukotriene D4 | control | no |  | diet_1 | no | 19,8181 | 0,0014 |  | I_Leukotriene D4 | control | no |  | control | yes | 3,0282 | 0,9077 |
| I_Leukotriene D4 | control | no |  | diet_3 | no | 19,8181 | 0,0014 |  | I_Leukotriene D4 | diet_1 | no |  | diet_1 | yes | 3,89E-17 | 1 |
| I_Leukotriene D4 | diet_1 | no |  | diet_3 | no | 1,06E-14 | 1 |  | I_Leukotriene D4 | diet_3 | no |  | diet_3 | yes | -3,46E-14 | 1 |
| I_Leukotriene D4 | control | yes |  | diet_1 | yes | 16,7899 | 0,005 |  |  |  |  |  |  |  |  |  |
| I_Leukotriene D4 | control | yes |  | diet_3 | yes | 16,7899 | 0,0061 |  |  |  |  |  |  |  |  |  |
| I_Leukotriene D4 | diet_1 | yes |  | diet_3 | yes | -2,40E-14 | 1 |  |  |  |  |  |  |  |  |  |

| Compound | Diet | LPS | vs | Diet | LPS | Estimate | P-value |  | Compound | Diet | LPS | vs | Diet | LPS | Estimate | P-value |
| --- | --- | --- | --- | --- | --- | --- | --- | --- | --- | --- | --- | --- | --- | --- | --- | --- |
| I_17 keto- 4(z), 7(z), 10(z), 13 (z), 15 (E), 19(z)-DHA | control | no |  | diet_1 | no | 2,6288 | 0,9976 |  | I_17 keto- 4(z), 7(z), 10(z), 13 (z), 15 (E), 19(z)-DHA | control | no |  | control | yes | 13,888 | 0,2918 |
| I_17 keto- 4(z), 7(z), 10(z), 13 (z), 15 (E), 19(z)-DHA | control | no |  | diet_3 | no | -13,8315 | 0,277 |  | I_17 keto- 4(z), 7(z), 10(z), 13 (z), 15 (E), 19(z)-DHA | diet_1 | no |  | diet_1 | yes | -5,1775 | 0,9512 |
| I_17 keto- 4(z), 7(z), 10(z), 13 (z), 15 (E), 19(z)-DHA | diet_1 | no |  | diet_3 | no | -16,4602 | 0,1466 |  | I_17 keto- 4(z), 7(z), 10(z), 13 (z), 15 (E), 19(z)-DHA | diet_3 | no |  | diet_3 | yes | 0,649 | 1 |
| I_17 keto- 4(z), 7(z), 10(z), 13 (z), 15 (E), 19(z)-DHA | control | yes |  | diet_1 | yes | -16,4367 | 0,1606 |  |  |  |  |  |  |  |  |  |
| I_17 keto- 4(z), 7(z), 10(z), 13 (z), 15 (E), 19(z)-DHA | control | yes |  | diet_3 | yes | -27,0705 | 0,0112 |  |  |  |  |  |  |  |  |  |
| I_17 keto- 4(z), 7(z), 10(z), 13 (z), 15 (E), 19(z)-DHA | diet_1 | yes |  | diet_3 | yes | -10,6338 | 0,5502 |  |  |  |  |  |  |  |  |  |
|  |  |  |  |  |  |  |  |  |  |  |  |  |  |  |  |  |
|  |  |  |  |  |  |  |  |  |  |  |  |  |  |  |  |  |
| I_12,13-DiHOME | control | no |  | diet_1 | no | -7,8802 | 0,9075 |  | I_12,13-DiHOME | control | no |  | control | yes | 3,3091 | 0,998 |
| I_12,13-DiHOME | control | no |  | diet_3 | no | 5,6588 | 0,9752 |  | I_12,13-DiHOME | diet_1 | no |  | diet_1 | yes | 14,3273 | 0,4898 |
| I_12,13-DiHOME | diet_1 | no |  | diet_3 | no | 13,5389 | 0,5449 |  | I_12,13-DiHOME | diet_3 | no |  | diet_3 | yes | -9,3074 | 0,846 |
| I_12,13-DiHOME | control | yes |  | diet_1 | yes | 3,138 | 0,9985 |  |  |  |  |  |  |  |  |  |
| I_12,13-DiHOME | control | yes |  | diet_3 | yes | -6,9577 | 0,9509 |  |  |  |  |  |  |  |  |  |
| I_12,13-DiHOME | diet_1 | yes |  | diet_3 | yes | -10,0957 | 0,8002 |  |  |  |  |  |  |  |  |  |
|  |  |  |  |  |  |  |  |  |  |  |  |  |  |  |  |  |
|  |  |  |  |  |  |  |  |  |  |  |  |  |  |  |  |  |
| I_9,10-DiHOME | control | no |  | diet_1 | no | -11,1588 | 0,8081 |  | I_9,10-DiHOME | control | no |  | control | yes | -1,541 | 1 |
| I_9,10-DiHOME | control | no |  | diet_3 | no | -1,2644 | 1 |  | I_9,10-DiHOME | diet_1 | no |  | diet_1 | yes | 11,9771 | 0,762 |
| I_9,10-DiHOME | diet_1 | no |  | diet_3 | no | 9,8944 | 0,871 |  | I_9,10-DiHOME | diet_3 | no |  | diet_3 | yes | -2,4731 | 0,9997 |
| I_9,10-DiHOME | control | yes |  | diet_1 | yes | 2,3593 | 0,9998 |  |  |  |  |  |  |  |  |  |
| I_9,10-DiHOME | control | yes |  | diet_3 | yes | -2,1966 | 0,9999 |  |  |  |  |  |  |  |  |  |
| I_9,10-DiHOME | diet_1 | yes |  | diet_3 | yes | -4,5559 | 0,9952 |  |  |  |  |  |  |  |  |  |

| Compound | Diet | LPS | vs | Diet | LPS | Estimate | P-value |  | Compound | Diet | LPS | vs | Diet | LPS | Estimate | P-value |
| --- | --- | --- | --- | --- | --- | --- | --- | --- | --- | --- | --- | --- | --- | --- | --- | --- |
| I_9,12,13-TriHOME | control | no |  | diet_1 | no | -3,5 | 0,9981 |  | I_9,12,13-TriHOME | control | no |  | control | yes | 0,8689 | 1 |
| I_9,12,13-TriHOME | control | no |  | diet_3 | no | -4,0905 | 0,996 |  | I_9,12,13-TriHOME | diet_1 | no |  | diet_1 | yes | 11,5677 | 0,7465 |
| I_9,12,13-TriHOME | diet_1 | no |  | diet_3 | no | -0,5905 | 1 |  | I_9,12,13-TriHOME | diet_3 | no |  | diet_3 | yes | 21,2984 | 0,2028 |
| I_9,12,13-TriHOME | control | yes |  | diet_1 | yes | 7,1988 | 0,954 |  |  |  |  |  |  |  |  |  |
| I_9,12,13-TriHOME | control | yes |  | diet_3 | yes | 16,339 | 0,4539 |  |  |  |  |  |  |  |  |  |
| I_9,12,13-TriHOME | diet_1 | yes |  | diet_3 | yes | 9,1402 | 0,8858 |  |  |  |  |  |  |  |  |  |
|  |  |  |  |  |  |  |  |  |  |  |  |  |  |  |  |  |
|  |  |  |  |  |  |  |  |  |  |  |  |  |  |  |  |  |
| I_9,10,13-TriHOME | control | no |  | diet_1 | no | 1,0807 | 1 |  | I_9,10,13-TriHOME | control | no |  | control | yes | 6,1119 | 0,9595 |
| I_9,10,13-TriHOME | control | no |  | diet_3 | no | -3,4329 | 0,9965 |  | I_9,10,13-TriHOME | diet_1 | no |  | diet_1 | yes | 13,7725 | 0,4696 |
| I_9,10,13-TriHOME | diet_1 | no |  | diet_3 | no | -4,5136 | 0,9877 |  | I_9,10,13-TriHOME | diet_3 | no |  | diet_3 | yes | 10,4852 | 0,7331 |
| I_9,10,13-TriHOME | control | yes |  | diet_1 | yes | 8,7414 | 0,847 |  |  |  |  |  |  |  |  |  |
| I_9,10,13-TriHOME | control | yes |  | diet_3 | yes | 0,9404 | 1 |  |  |  |  |  |  |  |  |  |
| I_9,10,13-TriHOME | diet_1 | yes |  | diet_3 | yes | -7,801 | 0,8967 |  |  |  |  |  |  |  |  |  |
|  |  |  |  |  |  |  |  |  |  |  |  |  |  |  |  |  |
|  |  |  |  |  |  |  |  |  |  |  |  |  |  |  |  |  |
| I_UK1 | control | no |  | diet_1 | no | -2,1306 | 0,7127 |  | I_UK1 | control | no |  | control | yes | 0,3758 | 0,9998 |
| I_UK1 | control | no |  | diet_3 | no | 0,1587 | 1 |  | I_UK1 | diet_1 | no |  | diet_1 | yes | 1,3297 | 0,9413 |
| I_UK1 | diet_1 | no |  | diet_3 | no | 2,2893 | 0,6532 |  | I_UK1 | diet_3 | no |  | diet_3 | yes | 0,7263 | 0,996 |
| I_UK1 | control | yes |  | diet_1 | yes | -1,1767 | 0,966 |  |  |  |  |  |  |  |  |  |
| I_UK1 | control | yes |  | diet_3 | yes | 0,5092 | 0,9993 |  |  |  |  |  |  |  |  |  |
| I_UK1 | diet_1 | yes |  | diet_3 | yes | 1,6859 | 0,8661 |  |  |  |  |  |  |  |  |  |

| Compound | Diet | LPS | vs | Diet | LPS | Estimate | P-value |  | Compound | Diet | LPS | vs | Diet | LPS | Estimate | P-value |
| --- | --- | --- | --- | --- | --- | --- | --- | --- | --- | --- | --- | --- | --- | --- | --- | --- |
| I_UK2 | control | no |  | diet_1 | no | -18,3275 | 0,1314 |  | I_UK2 | control | no |  | control | yes | -3,3384 | 0,9951 |
| I_UK2 | control | no |  | diet_3 | no | -1,9488 | 0,9996 |  | I_UK2 | diet_1 | no |  | diet_1 | yes | 10,1706 | 0,6343 |
| I_UK2 | diet_1 | no |  | diet_3 | no | 16,3788 | 0,2029 |  | I_UK2 | diet_3 | no |  | diet_3 | yes | -0,4014 | 1 |
| I_UK2 | control | yes |  | diet_1 | yes | -4,8186 | 0,975 |  |  |  |  |  |  |  |  |  |
| I_UK2 | control | yes |  | diet_3 | yes | 0,9883 | 1 |  |  |  |  |  |  |  |  |  |
| I_UK2 | diet_1 | yes |  | diet_3 | yes | 5,8068 | 0,9464 |  |  |  |  |  |  |  |  |  |
|  |  |  |  |  |  |  |  |  |  |  |  |  |  |  |  |  |
|  |  |  |  |  |  |  |  |  |  |  |  |  |  |  |  |  |
| I_UK3 | control | no |  | diet_1 | no | -0,05182 | 0,9121 |  | I_UK3 | control | no |  | control | yes | 0,01083 | 0,9999 |
| I_UK3 | control | no |  | diet_3 | no | 0,01798 | 0,9992 |  | I_UK3 | diet_1 | no |  | diet_1 | yes | 0,04129 | 0,964 |
| I_UK3 | diet_1 | no |  | diet_3 | no | 0,0698 | 0,7621 |  | I_UK3 | diet_3 | no |  | diet_3 | yes | 0,004099 | 1 |
| I_UK3 | control | yes |  | diet_1 | yes | -0,02136 | 0,9982 |  |  |  |  |  |  |  |  |  |
| I_UK3 | control | yes |  | diet_3 | yes | 0,01125 | 0,9999 |  |  |  |  |  |  |  |  |  |
| I_UK3 | diet_1 | yes |  | diet_3 | yes | 0,03261 | 0,9876 |  |  |  |  |  |  |  |  |  |
|  |  |  |  |  |  |  |  |  |  |  |  |  |  |  |  |  |
|  |  |  |  |  |  |  |  |  |  |  |  |  |  |  |  |  |
| I_UK4 | control | no |  | diet_1 | no | -0,00902 | 0,9959 |  | I_UK4 | control | no |  | control | yes | 0,009516 | 0,9953 |
| I_UK4 | control | no |  | diet_3 | no | 0,001392 | 1 |  | I_UK4 | diet_1 | no |  | diet_1 | yes | 0,02115 | 0,8606 |
| I_UK4 | diet_1 | no |  | diet_3 | no | 0,01042 | 0,9921 |  | I_UK4 | diet_3 | no |  | diet_3 | yes | 0,01859 | 0,9184 |
| I_UK4 | control | yes |  | diet_1 | yes | 0,002611 | 1 |  |  |  |  |  |  |  |  |  |
| I_UK4 | control | yes |  | diet_3 | yes | 0,01047 | 0,9933 |  |  |  |  |  |  |  |  |  |
| I_UK4 | diet_1 | yes |  | diet_3 | yes | 0,007855 | 0,9981 |  |  |  |  |  |  |  |  |  |
|  |  |  |  |  |  |  |  |  |  |  |  |  |  |  |  |  |
|  |  |  |  |  |  |  |  |  |  |  |  |  |  |  |  |  |
| I_UK5 | control | no |  | diet_1 | no | -10,0488 | 0,8741 |  | I_UK5 | control | no |  | control | yes | -2,7004 | 0,9996 |
| I_UK5 | control | no |  | diet_3 | no | 5,7478 | 0,9866 |  | I_UK5 | diet_1 | no |  | diet_1 | yes | 8,727 | 0,9246 |
| I_UK5 | diet_1 | no |  | diet_3 | no | 15,7966 | 0,5454 |  | I_UK5 | diet_3 | no |  | diet_3 | yes | -2,8491 | 0,9995 |
| I_UK5 | control | yes |  | diet_1 | yes | 1,3786 | 1 |  |  |  |  |  |  |  |  |  |
| I_UK5 | control | yes |  | diet_3 | yes | 5,5991 | 0,9892 |  |  |  |  |  |  |  |  |  |
| I_UK5 | diet_1 | yes |  | diet_3 | yes | 4,2205 | 0,9969 |  |  |  |  |  |  |  |  |  |

| Compound | Diet | LPS | vs | Diet | LPS | Estimate | P-value |  | Compound | Diet | LPS | vs | Diet | LPS | Estimate | P-value |
| --- | --- | --- | --- | --- | --- | --- | --- | --- | --- | --- | --- | --- | --- | --- | --- | --- |
| I_epea | control | no |  | diet_1 | no | -15,1964 | 0,0085 |  | I_epea | control | no |  | control | yes | 2,0756 | 0,9893 |
| I_epea | control | no |  | diet_3 | no | -26,5315 | <.0001 |  | I_epea | diet_1 | no |  | diet_1 | yes | -2,937 | 0,947 |
| I_epea | diet_1 | no |  | diet_3 | no | -11,3351 | 0,0541 |  | I_epea | diet_3 | no |  | diet_3 | yes | -2,0727 | 0,9894 |
| I_epea | control | yes |  | diet_1 | yes | -20,209 | 0,0012 |  |  |  |  |  |  |  |  |  |
| I_epea | control | yes |  | diet_3 | yes | -30,6799 | <.0001 |  |  |  |  |  |  |  |  |  |
| I_epea | diet_1 | yes |  | diet_3 | yes | -10,4708 | 0,0944 |  |  |  |  |  |  |  |  |  |
|  |  |  |  |  |  |  |  |  |  |  |  |  |  |  |  |  |
|  |  |  |  |  |  |  |  |  |  |  |  |  |  |  |  |  |
| I_dhea | control | no |  | diet_1 | no | -14 | 0,0283 |  | I_dhea | control | no |  | control | yes | -10,4286 | 0,2105 |
| I_dhea | control | no |  | diet_3 | no | -25,625 | <.0001 |  | I_dhea | diet_1 | no |  | diet_1 | yes | -11,125 | 0,1312 |
| I_dhea | diet_1 | no |  | diet_3 | no | -11,625 | 0,1029 |  | I_dhea | diet_3 | no |  | diet_3 | yes | -4,9464 | 0,8785 |
| I_dhea | control | yes |  | diet_1 | yes | -14,6964 | 0,0252 |  |  |  |  |  |  |  |  |  |
| I_dhea | control | yes |  | diet_3 | yes | -20,1429 | 0,0013 |  |  |  |  |  |  |  |  |  |
| I_dhea | diet_1 | yes |  | diet_3 | yes | -5,4464 | 0,8289 |  |  |  |  |  |  |  |  |  |
|  |  |  |  |  |  |  |  |  |  |  |  |  |  |  |  |  |
|  |  |  |  |  |  |  |  |  |  |  |  |  |  |  |  |  |
| I_aea | control | no |  | diet_1 | no | 13,125 | 0,1741 |  | I_aea | control | no |  | control | yes | -15,6071 | 0,0819 |
| I_aea | control | no |  | diet_3 | no | 7 | 0,7885 |  | I_aea | diet_1 | no |  | diet_1 | yes | -10,5 | 0,3965 |
| I_aea | diet_1 | no |  | diet_3 | no | -6,125 | 0,8664 |  | I_aea | diet_3 | no |  | diet_3 | yes | -5,8929 | 0,898 |
| I_aea | control | yes |  | diet_1 | yes | 18,2321 | 0,0266 |  |  |  |  |  |  |  |  |  |
| I_aea | control | yes |  | diet_3 | yes | 16,7143 | 0,0648 |  |  |  |  |  |  |  |  |  |
| I_aea | diet_1 | yes |  | diet_3 | yes | -1,5179 | 0,9998 |  |  |  |  |  |  |  |  |  |
|  |  |  |  |  |  |  |  |  |  |  |  |  |  |  |  |  |
|  |  |  |  |  |  |  |  |  |  |  |  |  |  |  |  |  |
| I_2-ag | control | no |  | diet_1 | no | 21,5013 | 0,0073 |  | I_2-ag | control | no |  | control | yes | -0,666 | 1 |
| I_2-ag | control | no |  | diet_3 | no | 23,0277 | 0,0043 |  | I_2-ag | diet_1 | no |  | diet_1 | yes | -2,6717 | 0,9923 |
| I_2-ag | diet_1 | no |  | diet_3 | no | 1,5264 | 0,9994 |  | I_2-ag | diet_3 | no |  | diet_3 | yes | -0,6767 | 1 |
| I_2-ag | control | yes |  | diet_1 | yes | 19,4956 | 0,0182 |  |  |  |  |  |  |  |  |  |
| I_2-ag | control | yes |  | diet_3 | yes | 23,017 | 0,0067 |  |  |  |  |  |  |  |  |  |
| I_2-ag | diet_1 | yes |  | diet_3 | yes | 3,5214 | 0,977 |  |  |  |  |  |  |  |  |  |

| Compound | Diet | LPS | vs | Diet | LPS | Estimate | P-value |  | Compound | Diet | LPS | vs | Diet | LPS | Estimate | P-value |
| --- | --- | --- | --- | --- | --- | --- | --- | --- | --- | --- | --- | --- | --- | --- | --- | --- |
| I_dle | control | no |  | diet_1 | no | 11,125 | 0,4094 |  | I_dle | control | no |  | control | yes | -13,3929 | 0,2493 |
| I_dle | control | no |  | diet_3 | no | 7,625 | 0,7772 |  | I_dle | diet_1 | no |  | diet_1 | yes | -12,875 | 0,2541 |
| I_dle | diet_1 | no |  | diet_3 | no | -3,5 | 0,9903 |  | I_dle | diet_3 | no |  | diet_3 | yes | -5,4464 | 0,943 |
| I_dle | control | yes |  | diet_1 | yes | 11,6429 | 0,3972 |  |  |  |  |  |  |  |  |  |
| I_dle | control | yes |  | diet_3 | yes | 15,5714 | 0,147 |  |  |  |  |  |  |  |  |  |
| I_dle | diet_1 | yes |  | diet_3 | yes | 3,9286 | 0,986 |  |  |  |  |  |  |  |  |  |
|  |  |  |  |  |  |  |  |  |  |  |  |  |  |  |  |  |
|  |  |  |  |  |  |  |  |  |  |  |  |  |  |  |  |  |
| I_pea | control | no |  | diet_1 | no | 10,1562 | 0,7655 |  | I_pea | control | no |  | control | yes | -8,2526 | 0,8852 |
| I_pea | control | no |  | diet_3 | no | 1,6445 | 0,9999 |  | I_pea | diet_1 | no |  | diet_1 | yes | -18,8545 | 0,2128 |
| I_pea | diet_1 | no |  | diet_3 | no | -8,5117 | 0,8667 |  | I_pea | diet_3 | no |  | diet_3 | yes | -18,0445 | 0,2575 |
| I_pea | control | yes |  | diet_1 | yes | -0,4456 | 1 |  |  |  |  |  |  |  |  |  |
| I_pea | control | yes |  | diet_3 | yes | -8,1474 | 0,8948 |  |  |  |  |  |  |  |  |  |
| I_pea | diet_1 | yes |  | diet_3 | yes | -7,7018 | 0,9106 |  |  |  |  |  |  |  |  |  |
|  |  |  |  |  |  |  |  |  |  |  |  |  |  |  |  |  |
|  |  |  |  |  |  |  |  |  |  |  |  |  |  |  |  |  |
| I_oea | control | no |  | diet_1 | no | 13,7946 | 0,4586 |  | I_oea | control | no |  | control | yes | -8,8131 | 0,8348 |
| I_oea | control | no |  | diet_3 | no | 10,7992 | 0,6859 |  | I_oea | diet_1 | no |  | diet_1 | yes | -17,3722 | 0,2457 |
| I_oea | diet_1 | no |  | diet_3 | no | -2,9954 | 0,9981 |  | I_oea | diet_3 | no |  | diet_3 | yes | -11,5853 | 0,6383 |
| I_oea | control | yes |  | diet_1 | yes | 5,2355 | 0,9775 |  |  |  |  |  |  |  |  |  |
| I_oea | control | yes |  | diet_3 | yes | 8,0269 | 0,8854 |  |  |  |  |  |  |  |  |  |
| I_oea | diet_1 | yes |  | diet_3 | yes | 2,7915 | 0,9987 |  |  |  |  |  |  |  |  |  |
|  |  |  |  |  |  |  |  |  |  |  |  |  |  |  |  |  |
|  |  |  |  |  |  |  |  |  |  |  |  |  |  |  |  |  |
| I_sea | control | no |  | diet_1 | no | 17,8548 | 0,8659 |  | I_sea | control | no |  | control | yes | -30,8352 | 0,4646 |
| I_sea | control | no |  | diet_3 | no | 31,4046 | 0,4202 |  | I_sea | diet_1 | no |  | diet_1 | yes | -33,3022 | 0,3637 |
| I_sea | diet_1 | no |  | diet_3 | no | 13,5498 | 0,952 |  | I_sea | diet_3 | no |  | diet_3 | yes | -60,0138 | 0,0349 |
| I_sea | control | yes |  | diet_1 | yes | 15,3878 | 0,929 |  |  |  |  |  |  |  |  |  |
| I_sea | control | yes |  | diet_3 | yes | 2,226 | 1 |  |  |  |  |  |  |  |  |  |
| I_sea | diet_1 | yes |  | diet_3 | yes | -13,1618 | 0,9617 |  |  |  |  |  |  |  |  |  |

| **Compound** | **Diet** | **LPS** | **vs** | **Diet** | **LPS** | **Estimate** | **P-value** |  | **Compound** | **Diet** | **LPS** | **vs** | **Diet** | **LPS** | **Estimate** | **P-value** |
| --- | --- | --- | --- | --- | --- | --- | --- | --- | --- | --- | --- | --- | --- | --- | --- | --- |
| F_AA | control | no |  | diet_1 | no | 6790,62 | 0,0511 |  | F_AA | control | no |  | control | yes | -3352,71 | 0,5814 |
| F_AA | control | no |  | diet_3 | no | 5605,27 | 0,13 |  | F_AA | diet_1 | no |  | diet_1 | yes | -4372,6 | 0,3153 |
| F_AA | diet_1 | no |  | diet_3 | no | -1185,34 | 0,9894 |  | F_AA | diet_3 | no |  | diet_3 | yes | -9433,8 | 0,0068 |
| F_AA | control | yes |  | diet_1 | yes | 5770,72 | 0,1201 |  |  |  |  |  |  |  |  |  |
| F_AA | control | yes |  | diet_3 | yes | -475,83 | 0,9999 |  |  |  |  |  |  |  |  |  |
| F_AA | diet_1 | yes |  | diet_3 | yes | -6246,55 | 0,0832 |  |  |  |  |  |  |  |  |  |
|  |  |  |  |  |  |  |  |  |  |  |  |  |  |  |  |  |
|  |  |  |  |  |  |  |  |  |  |  |  |  |  |  |  |  |
| F_EPA | control | no |  | diet_1 | no | -15,5 | 0,0244 |  | F_EPA | control | no |  | control | yes | -2,924 | 0,9766 |
| F_EPA | control | no |  | diet_3 | no | -24,5737 | 0,0007 |  | F_EPA | diet_1 | no |  | diet_1 | yes | -7,3997 | 0,4911 |
| F_EPA | diet_1 | no |  | diet_3 | no | -9,0737 | 0,2946 |  | F_EPA | diet_3 | no |  | diet_3 | yes | -11,0457 | 0,1502 |
| F_EPA | control | yes |  | diet_1 | yes | -19,9757 | 0,0043 |  |  |  |  |  |  |  |  |  |
| F_EPA | control | yes |  | diet_3 | yes | -32,6954 | <.0001 |  |  |  |  |  |  |  |  |  |
| F_EPA | diet_1 | yes |  | diet_3 | yes | -12,7197 | 0,0787 |  |  |  |  |  |  |  |  |  |
|  |  |  |  |  |  |  |  |  |  |  |  |  |  |  |  |  |
|  |  |  |  |  |  |  |  |  |  |  |  |  |  |  |  |  |
| F_DHA | control | no |  | diet_1 | no | -2933,12 | 0,3422 |  | F_DHA | control | no |  | control | yes | -3797,78 | 0,1627 |
| F_DHA | control | no |  | diet_3 | no | -2026,1 | 0,686 |  | F_DHA | diet_1 | no |  | diet_1 | yes | 2049,89 | 0,6763 |
| F_DHA | diet_1 | no |  | diet_3 | no | 907,02 | 0,9827 |  | F_DHA | diet_3 | no |  | diet_3 | yes | -213,08 | 1 |
| F_DHA | control | yes |  | diet_1 | yes | 2914,54 | 0,3768 |  |  |  |  |  |  |  |  |  |
| F_DHA | control | yes |  | diet_3 | yes | 1558,6 | 0,8832 |  |  |  |  |  |  |  |  |  |
| F_DHA | diet_1 | yes |  | diet_3 | yes | -1355,95 | 0,9216 |  |  |  |  |  |  |  |  |  |
|  |  |  |  |  |  |  |  |  |  |  |  |  |  |  |  |  |
|  |  |  |  |  |  |  |  |  |  |  |  |  |  |  |  |  |
| F_12(S)-HHTrE | control | no |  | diet_1 | no | 14,4076 | 0,1787 |  | F_12(S)-HHTrE | control | no |  | control | yes | -15,0525 | 0,1609 |
| F_12(S)-HHTrE | control | no |  | diet_3 | no | 12,192 | 0,315 |  | F_12(S)-HHTrE | diet_1 | no |  | diet_1 | yes | -22,1327 | 0,0187 |
| F_12(S)-HHTrE | diet_1 | no |  | diet_3 | no | -2,2156 | 0,9984 |  | F_12(S)-HHTrE | diet_3 | no |  | diet_3 | yes | -15,2774 | 0,1514 |
| F_12(S)-HHTrE | control | yes |  | diet_1 | yes | 7,3274 | 0,787 |  |  |  |  |  |  |  |  |  |
| F_12(S)-HHTrE | control | yes |  | diet_3 | yes | 11,967 | 0,3649 |  |  |  |  |  |  |  |  |  |
| F_12(S)-HHTrE | diet_1 | yes |  | diet_3 | yes | 4,6396 | 0,9592 |  |  |  |  |  |  |  |  |  |

| Compound | Diet | LPS | vs | Diet | LPS | Estimate | P-value |  | Compound | Diet | LPS | vs | Diet | LPS | Estimate | P-value |
| --- | --- | --- | --- | --- | --- | --- | --- | --- | --- | --- | --- | --- | --- | --- | --- | --- |
| F_15(S)-HETE | control | no |  | diet_1 | no | 24,144 | 0,0056 |  | F_15(S)-HETE | control | no |  | control | yes | -4,9261 | 0,9318 |
| F_15(S)-HETE | control | no |  | diet_3 | no | 16,7634 | 0,0611 |  | F_15(S)-HETE | diet_1 | no |  | diet_1 | yes | -14,3589 | 0,1301 |
| F_15(S)-HETE | diet_1 | no |  | diet_3 | no | -7,3806 | 0,7119 |  | F_15(S)-HETE | diet_3 | no |  | diet_3 | yes | -0,9647 | 1 |
| F_15(S)-HETE | control | yes |  | diet_1 | yes | 14,7112 | 0,1318 |  |  |  |  |  |  |  |  |  |
| F_15(S)-HETE | control | yes |  | diet_3 | yes | 20,7249 | 0,0239 |  |  |  |  |  |  |  |  |  |
| F_15(S)-HETE | diet_1 | yes |  | diet_3 | yes | 6,0136 | 0,8592 |  |  |  |  |  |  |  |  |  |
|  |  |  |  |  |  |  |  |  |  |  |  |  |  |  |  |  |
|  |  |  |  |  |  |  |  |  |  |  |  |  |  |  |  |  |
| F_11(S)-HETE | control | no |  | diet_1 | no | 15,3655 | 0,1976 |  | F_11(S)-HETE | control | no |  | control | yes | -13,1631 | 0,3402 |
| F_11(S)-HETE | control | no |  | diet_3 | no | 13,7556 | 0,288 |  | F_11(S)-HETE | diet_1 | no |  | diet_1 | yes | -19,8453 | 0,062 |
| F_11(S)-HETE | diet_1 | no |  | diet_3 | no | -1,6098 | 0,9998 |  | F_11(S)-HETE | diet_3 | no |  | diet_3 | yes | -18,1946 | 0,1023 |
| F_11(S)-HETE | control | yes |  | diet_1 | yes | 8,6833 | 0,7278 |  |  |  |  |  |  |  |  |  |
| F_11(S)-HETE | control | yes |  | diet_3 | yes | 8,7241 | 0,7337 |  |  |  |  |  |  |  |  |  |
| F_11(S)-HETE | diet_1 | yes |  | diet_3 | yes | 0,04082 | 1 |  |  |  |  |  |  |  |  |  |
|  |  |  |  |  |  |  |  |  |  |  |  |  |  |  |  |  |
|  |  |  |  |  |  |  |  |  |  |  |  |  |  |  |  |  |
| F_12(S)-HETE | control | no |  | diet_1 | no | 18,5327 | 0,1891 |  | F_12(S)-HETE | control | no |  | control | yes | -6,7103 | 0,9371 |
| F_12(S)-HETE | control | no |  | diet_3 | no | 8,4903 | 0,8452 |  | F_12(S)-HETE | diet_1 | no |  | diet_1 | yes | -18,4303 | 0,1931 |
| F_12(S)-HETE | diet_1 | no |  | diet_3 | no | -10,0423 | 0,7397 |  | F_12(S)-HETE | diet_3 | no |  | diet_3 | yes | 5,74 | 0,9664 |
| F_12(S)-HETE | control | yes |  | diet_1 | yes | 6,8127 | 0,9333 |  |  |  |  |  |  |  |  |  |
| F_12(S)-HETE | control | yes |  | diet_3 | yes | 20,9407 | 0,128 |  |  |  |  |  |  |  |  |  |
| F_12(S)-HETE | diet_1 | yes |  | diet_3 | yes | 14,128 | 0,4421 |  |  |  |  |  |  |  |  |  |
|  |  |  |  |  |  |  |  |  |  |  |  |  |  |  |  |  |
|  |  |  |  |  |  |  |  |  |  |  |  |  |  |  |  |  |
| F_11,12 EET | control | no |  | diet_1 | no | 0,1214 | 0,0055 |  | F_11,12 EET | control | no |  | control | yes | -0,01786 | 0,9939 |
| F_11,12 EET | control | no |  | diet_3 | no | 0,1177 | 0,0077 |  | F_11,12 EET | diet_1 | no |  | diet_1 | yes | -0,08271 | 0,119 |
| F_11,12 EET | diet_1 | no |  | diet_3 | no | -0,00379 | 1 |  | F_11,12 EET | diet_3 | no |  | diet_3 | yes | -0,1099 | 0,0206 |
| F_11,12 EET | control | yes |  | diet_1 | yes | 0,0566 | 0,5249 |  |  |  |  |  |  |  |  |  |
| F_11,12 EET | control | yes |  | diet_3 | yes | 0,02563 | 0,9733 |  |  |  |  |  |  |  |  |  |
| F_11,12 EET | diet_1 | yes |  | diet_3 | yes | -0,03097 | 0,9327 |  |  |  |  |  |  |  |  |  |

| Compound | Diet | LPS | vs | Diet | LPS | Estimate | P-value |  | Compound | Diet | LPS | vs | Diet | LPS | Estimate | P-value |
| --- | --- | --- | --- | --- | --- | --- | --- | --- | --- | --- | --- | --- | --- | --- | --- | --- |
| F_8,9 EET | control | no |  | diet_1 | no | 0,08755 | 0,9882 |  | F_8,9 EET | control | no |  | control | yes | -0,1263 | 0,9501 |
| F_8,9 EET | control | no |  | diet_3 | no | -0,1602 | 0,8572 |  | F_8,9 EET | diet_1 | no |  | diet_1 | yes | -0,2426 | 0,514 |
| F_8,9 EET | diet_1 | no |  | diet_3 | no | -0,2478 | 0,4905 |  | F_8,9 EET | diet_3 | no |  | diet_3 | yes | -0,00942 | 1 |
| F_8,9 EET | control | yes |  | diet_1 | yes | -0,02869 | 1 |  |  |  |  |  |  |  |  |  |
| F_8,9 EET | control | yes |  | diet_3 | yes | -0,04335 | 0,9997 |  |  |  |  |  |  |  |  |  |
| F_8,9 EET | diet_1 | yes |  | diet_3 | yes | -0,01465 | 1 |  |  |  |  |  |  |  |  |  |
|  |  |  |  |  |  |  |  |  |  |  |  |  |  |  |  |  |
|  |  |  |  |  |  |  |  |  |  |  |  |  |  |  |  |  |
| F_5,6 EET | control | no |  | diet_1 | no | 0,9462 | 0,6092 |  | F_5,6 EET | control | no |  | control | yes | -0,3993 | 0,9821 |
| F_5,6 EET | control | no |  | diet_3 | no | 0,4622 | 0,9634 |  | F_5,6 EET | diet_1 | no |  | diet_1 | yes | -0,9588 | 0,5973 |
| F_5,6 EET | diet_1 | no |  | diet_3 | no | -0,4839 | 0,9559 |  | F_5,6 EET | diet_3 | no |  | diet_3 | yes | -1,503 | 0,2116 |
| F_5,6 EET | control | yes |  | diet_1 | yes | 0,3868 | 0,9844 |  |  |  |  |  |  |  |  |  |
| F_5,6 EET | control | yes |  | diet_3 | yes | -0,6414 | 0,8923 |  |  |  |  |  |  |  |  |  |
| F_5,6 EET | diet_1 | yes |  | diet_3 | yes | -1,0281 | 0,5544 |  |  |  |  |  |  |  |  |  |
|  |  |  |  |  |  |  |  |  |  |  |  |  |  |  |  |  |
|  |  |  |  |  |  |  |  |  |  |  |  |  |  |  |  |  |
| F_5(S)-HETE | control | no |  | diet_1 | no | 14,9022 | 0,1133 |  | F_5(S)-HETE | control | no |  | control | yes | -13,0648 | 0,2043 |
| F_5(S)-HETE | control | no |  | diet_3 | no | 7,1432 | 0,7336 |  | F_5(S)-HETE | diet_1 | no |  | diet_1 | yes | -18,6891 | 0,0354 |
| F_5(S)-HETE | diet_1 | no |  | diet_3 | no | -7,759 | 0,6674 |  | F_5(S)-HETE | diet_3 | no |  | diet_3 | yes | -24,4167 | 0,0068 |
| F_5(S)-HETE | control | yes |  | diet_1 | yes | 9,2779 | 0,5159 |  |  |  |  |  |  |  |  |  |
| F_5(S)-HETE | control | yes |  | diet_3 | yes | -4,2087 | 0,9627 |  |  |  |  |  |  |  |  |  |
| F_5(S)-HETE | diet_1 | yes |  | diet_3 | yes | -13,4865 | 0,1816 |  |  |  |  |  |  |  |  |  |
|  |  |  |  |  |  |  |  |  |  |  |  |  |  |  |  |  |
|  |  |  |  |  |  |  |  |  |  |  |  |  |  |  |  |  |
| F_14,15 EET | control | no |  | diet_1 | no | 0,1105 | 0,3471 |  | F_14,15 EET | control | no |  | control | yes | -0,04472 | 0,9512 |
| F_14,15 EET | control | no |  | diet_3 | no | 0,1048 | 0,3967 |  | F_14,15 EET | diet_1 | no |  | diet_1 | yes | 0,001067 | 1 |
| F_14,15 EET | diet_1 | no |  | diet_3 | no | -0,00569 | 1 |  | F_14,15 EET | diet_3 | no |  | diet_3 | yes | -0,09714 | 0,4845 |
| F_14,15 EET | control | yes |  | diet_1 | yes | 0,1563 | 0,1078 |  |  |  |  |  |  |  |  |  |
| F_14,15 EET | control | yes |  | diet_3 | yes | 0,05237 | 0,9154 |  |  |  |  |  |  |  |  |  |
| F_14,15 EET | diet_1 | yes |  | diet_3 | yes | -0,1039 | 0,4194 |  |  |  |  |  |  |  |  |  |

| Compound | Diet | LPS | vs | Diet | LPS | Estimate | P-value |  | Compound | Diet | LPS | vs | Diet | LPS | Estimate | P-value |
| --- | --- | --- | --- | --- | --- | --- | --- | --- | --- | --- | --- | --- | --- | --- | --- | --- |
| F_PGB2 | control | no |  | diet_1 | no | -0,01633 | 0,8726 |  | F_PGB2 | control | no |  | control | yes | -0,02135 | 0,7225 |
| F_PGB2 | control | no |  | diet_3 | no | -0,01875 | 0,7943 |  | F_PGB2 | diet_1 | no |  | diet_1 | yes | 0,01035 | 0,9801 |
| F_PGB2 | diet_1 | no |  | diet_3 | no | -0,00242 | 1 |  | F_PGB2 | diet_3 | no |  | diet_3 | yes | 0,01487 | 0,9215 |
| F_PGB2 | control | yes |  | diet_1 | yes | 0,01536 | 0,9109 |  |  |  |  |  |  |  |  |  |
| F_PGB2 | control | yes |  | diet_3 | yes | 0,01747 | 0,8722 |  |  |  |  |  |  |  |  |  |
| F_PGB2 | diet_1 | yes |  | diet_3 | yes | 0,002107 | 1 |  |  |  |  |  |  |  |  |  |
|  |  |  |  |  |  |  |  |  |  |  |  |  |  |  |  |  |
|  |  |  |  |  |  |  |  |  |  |  |  |  |  |  |  |  |
| F_9(S)-HODE | control | no |  | diet_1 | no | 15,7178 | 0,0723 |  | F_9(S)-HODE | control | no |  | control | yes | -11,2437 | 0,2926 |
| F_9(S)-HODE | control | no |  | diet_3 | no | 17,034 | 0,0473 |  | F_9(S)-HODE | diet_1 | no |  | diet_1 | yes | -26,459 | 0,0024 |
| F_9(S)-HODE | diet_1 | no |  | diet_3 | no | 1,3162 | 0,9998 |  | F_9(S)-HODE | diet_3 | no |  | diet_3 | yes | -19,5722 | 0,0227 |
| F_9(S)-HODE | control | yes |  | diet_1 | yes | 0,5025 | 1 |  |  |  |  |  |  |  |  |  |
| F_9(S)-HODE | control | yes |  | diet_3 | yes | 8,7054 | 0,5506 |  |  |  |  |  |  |  |  |  |
| F_9(S)-HODE | diet_1 | yes |  | diet_3 | yes | 8,2029 | 0,5938 |  |  |  |  |  |  |  |  |  |
|  |  |  |  |  |  |  |  |  |  |  |  |  |  |  |  |  |
|  |  |  |  |  |  |  |  |  |  |  |  |  |  |  |  |  |
| F_LTB4 | control | no |  | diet_1 | no | -1,07E-14 | 1 |  | F_LTB4 | control | no |  | control | yes | -3,625 | 0,6256 |
| F_LTB4 | control | no |  | diet_3 | no | -5,33E-14 | 1 |  | F_LTB4 | diet_1 | no |  | diet_1 | yes | 0 | 1 |
| F_LTB4 | diet_1 | no |  | diet_3 | no | -4,26E-14 | 1 |  | F_LTB4 | diet_3 | no |  | diet_3 | yes | -22,7234 | <.0001 |
| F_LTB4 | control | yes |  | diet_1 | yes | 3,625 | 0,6256 |  |  |  |  |  |  |  |  |  |
| F_LTB4 | control | yes |  | diet_3 | yes | -19,0985 | <.0001 |  |  |  |  |  |  |  |  |  |
| F_LTB4 | diet_1 | yes |  | diet_3 | yes | -22,7234 | <.0001 |  |  |  |  |  |  |  |  |  |
|  |  |  |  |  |  |  |  |  |  |  |  |  |  |  |  |  |
|  |  |  |  |  |  |  |  |  |  |  |  |  |  |  |  |  |
| F_14,15-DiHETrE | control | no |  | diet_1 | no | 0,4372 | 0,0045 |  | F_14,15-DiHETrE | control | no |  | control | yes | 0,1562 | 0,5144 |
| F_14,15-DiHETrE | control | no |  | diet_3 | no | 0,3823 | 0,0113 |  | F_14,15-DiHETrE | diet_1 | no |  | diet_1 | yes | -0,04773 | 0,9916 |
| F_14,15-DiHETrE | diet_1 | no |  | diet_3 | no | -0,05493 | 0,9842 |  | F_14,15-DiHETrE | diet_3 | no |  | diet_3 | yes | -0,4655 | 0,0035 |
| F_14,15-DiHETrE | control | yes |  | diet_1 | yes | 0,2333 | 0,1685 |  |  |  |  |  |  |  |  |  |
| F_14,15-DiHETrE | control | yes |  | diet_3 | yes | -0,2394 | 0,1687 |  |  |  |  |  |  |  |  |  |
| F_14,15-DiHETrE | diet_1 | yes |  | diet_3 | yes | -0,4727 | 0,0032 |  |  |  |  |  |  |  |  |  |

| Compound | Diet | LPS | vs | Diet | LPS | Estimate | P-value |  | Compound | Diet | LPS | vs | Diet | LPS | Estimate | P-value |
| --- | --- | --- | --- | --- | --- | --- | --- | --- | --- | --- | --- | --- | --- | --- | --- | --- |
| F_11,12-DiHETrE | control | no |  | diet_1 | no | 0,1677 | 0,0064 |  | F_11,12-DiHETrE | control | no |  | control | yes | 0,009283 | 0,9998 |
| F_11,12-DiHETrE | control | no |  | diet_3 | no | 0,1612 | 0,0086 |  | F_11,12-DiHETrE | diet_1 | no |  | diet_1 | yes | -0,01054 | 0,9996 |
| F_11,12-DiHETrE | diet_1 | no |  | diet_3 | no | -0,00652 | 1 |  | F_11,12-DiHETrE | diet_3 | no |  | diet_3 | yes | -0,1209 | 0,059 |
| F_11,12-DiHETrE | control | yes |  | diet_1 | yes | 0,1479 | 0,0179 |  |  |  |  |  |  |  |  |  |
| F_11,12-DiHETrE | control | yes |  | diet_3 | yes | 0,03104 | 0,9556 |  |  |  |  |  |  |  |  |  |
| F_11,12-DiHETrE | diet_1 | yes |  | diet_3 | yes | -0,1169 | 0,0703 |  |  |  |  |  |  |  |  |  |
|  |  |  |  |  |  |  |  |  |  |  |  |  |  |  |  |  |
|  |  |  |  |  |  |  |  |  |  |  |  |  |  |  |  |  |
| F_5,6-DiHETrE | control | no |  | diet_1 | no | 21,8668 | 0,0233 |  | F_5,6-DiHETrE | control | no |  | control | yes | -3,5629 | 0,9858 |
| F_5,6-DiHETrE | control | no |  | diet_3 | no | 19,6186 | 0,0434 |  | F_5,6-DiHETrE | diet_1 | no |  | diet_1 | yes | -12,5842 | 0,2837 |
| F_5,6-DiHETrE | diet_1 | no |  | diet_3 | no | -2,2483 | 0,9981 |  | F_5,6-DiHETrE | diet_3 | no |  | diet_3 | yes | -18,136 | 0,0733 |
| F_5,6-DiHETrE | control | yes |  | diet_1 | yes | 12,8455 | 0,2854 |  |  |  |  |  |  |  |  |  |
| F_5,6-DiHETrE | control | yes |  | diet_3 | yes | 5,0455 | 0,9446 |  |  |  |  |  |  |  |  |  |
| F_5,6-DiHETrE | diet_1 | yes |  | diet_3 | yes | -7,8001 | 0,7369 |  |  |  |  |  |  |  |  |  |
|  |  |  |  |  |  |  |  |  |  |  |  |  |  |  |  |  |
|  |  |  |  |  |  |  |  |  |  |  |  |  |  |  |  |  |
| F_17(S)-HDoHE | control | no |  | diet_1 | no | -8,6443 | 0,7179 |  | F_17(S)-HDoHE | control | no |  | control | yes | -4,1022 | 0,9832 |
| F_17(S)-HDoHE | control | no |  | diet_3 | no | -17,3962 | 0,1237 |  | F_17(S)-HDoHE | diet_1 | no |  | diet_1 | yes | -11,947 | 0,4221 |
| F_17(S)-HDoHE | diet_1 | no |  | diet_3 | no | -8,7519 | 0,7083 |  | F_17(S)-HDoHE | diet_3 | no |  | diet_3 | yes | -6,8665 | 0,8716 |
| F_17(S)-HDoHE | control | yes |  | diet_1 | yes | -16,4891 | 0,1692 |  |  |  |  |  |  |  |  |  |
| F_17(S)-HDoHE | control | yes |  | diet_3 | yes | -20,1605 | 0,0773 |  |  |  |  |  |  |  |  |  |
| F_17(S)-HDoHE | diet_1 | yes |  | diet_3 | yes | -3,6713 | 0,9897 |  |  |  |  |  |  |  |  |  |
|  |  |  |  |  |  |  |  |  |  |  |  |  |  |  |  |  |
|  |  |  |  |  |  |  |  |  |  |  |  |  |  |  |  |  |
| F_PGE3 | control | no |  | diet_1 | no | -6,3161 | 0,5489 |  | F_PGE3 | control | no |  | control | yes | -1,0925 | 0,9996 |
| F_PGE3 | control | no |  | diet_3 | no | -17,9598 | 0,0043 |  | F_PGE3 | diet_1 | no |  | diet_1 | yes | -17,4434 | 0,0054 |
| F_PGE3 | diet_1 | no |  | diet_3 | no | -11,6437 | 0,0715 |  | F_PGE3 | diet_3 | no |  | diet_3 | yes | -14,4662 | 0,0225 |
| F_PGE3 | control | yes |  | diet_1 | yes | -22,6669 | 0,0007 |  |  |  |  |  |  |  |  |  |
| F_PGE3 | control | yes |  | diet_3 | yes | -31,3335 | <.0001 |  |  |  |  |  |  |  |  |  |
| F_PGE3 | diet_1 | yes |  | diet_3 | yes | -8,6666 | 0,2626 |  |  |  |  |  |  |  |  |  |

| Compound | Diet | LPS | vs | Diet | LPS | Estimate | P-value |  | Compound | Diet | LPS | vs | Diet | LPS | Estimate | P-value |
| --- | --- | --- | --- | --- | --- | --- | --- | --- | --- | --- | --- | --- | --- | --- | --- | --- |
| F_PGD3 | control | no |  | diet_1 | no | -10,6111 | 0,2373 |  | F_PGD3 | control | no |  | control | yes | -3,55E-15 | 1 |
| F_PGD3 | control | no |  | diet_3 | no | -18,599 | 0,0133 |  | F_PGD3 | diet_1 | no |  | diet_1 | yes | -11,4606 | 0,1794 |
| F_PGD3 | diet_1 | no |  | diet_3 | no | -7,9879 | 0,5037 |  | F_PGD3 | diet_3 | no |  | diet_3 | yes | -13,0081 | 0,1104 |
| F_PGD3 | control | yes |  | diet_1 | yes | -22,0717 | 0,0041 |  |  |  |  |  |  |  |  |  |
| F_PGD3 | control | yes |  | diet_3 | yes | -31,6071 | 0,0002 |  |  |  |  |  |  |  |  |  |
| F_PGD3 | diet_1 | yes |  | diet_3 | yes | -9,5355 | 0,3416 |  |  |  |  |  |  |  |  |  |
|  |  |  |  |  |  |  |  |  |  |  |  |  |  |  |  |  |
|  |  |  |  |  |  |  |  |  |  |  |  |  |  |  |  |  |
| F_13,14-dihydro-15-keto-PGE2 | control | no |  | diet_1 | no | 17,5625 | 0,0108 |  | F_13,14-dihydro-15-keto-PGE2 | control | no |  | control | yes | -9,8571 | 0,3911 |
| F_13,14-dihydro-15-keto-PGE2 | control | no |  | diet_3 | no | 15,5 | 0,0327 |  | F_13,14-dihydro-15-keto-PGE2 | diet_1 | no |  | diet_1 | yes | -6,5625 | 0,7611 |
| F_13,14-dihydro-15-keto-PGE2 | diet_1 | no |  | diet_3 | no | -2,0625 | 0,9982 |  | F_13,14-dihydro-15-keto-PGE2 | diet_3 | no |  | diet_3 | yes | -13,2857 | 0,1155 |
| F_13,14-dihydro-15-keto-PGE2 | control | yes |  | diet_1 | yes | 20,8571 | 0,0024 |  |  |  |  |  |  |  |  |  |
| F_13,14-dihydro-15-keto-PGE2 | control | yes |  | diet_3 | yes | 12,0714 | 0,2155 |  |  |  |  |  |  |  |  |  |
| F_13,14-dihydro-15-keto-PGE2 | diet_1 | yes |  | diet_3 | yes | -8,7857 | 0,5187 |  |  |  |  |  |  |  |  |  |
|  |  |  |  |  |  |  |  |  |  |  |  |  |  |  |  |  |
|  |  |  |  |  |  |  |  |  |  |  |  |  |  |  |  |  |
| F_13(S)-HODE | control | no |  | diet_1 | no | 20,9911 | 0,0397 |  | F_13(S)-HODE | control | no |  | control | yes | -4,8585 | 0,9606 |
| F_13(S)-HODE | control | no |  | diet_3 | no | 21,1413 | 0,038 |  | F_13(S)-HODE | diet_1 | no |  | diet_1 | yes | -24,5465 | 0,0148 |
| F_13(S)-HODE | diet_1 | no |  | diet_3 | no | 0,1502 | 1 |  | F_13(S)-HODE | diet_3 | no |  | diet_3 | yes | -13,214 | 0,3097 |
| F_13(S)-HODE | control | yes |  | diet_1 | yes | 1,3031 | 0,9999 |  |  |  |  |  |  |  |  |  |
| F_13(S)-HODE | control | yes |  | diet_3 | yes | 12,7857 | 0,3499 |  |  |  |  |  |  |  |  |  |
| F_13(S)-HODE | diet_1 | yes |  | diet_3 | yes | 11,4826 | 0,4446 |  |  |  |  |  |  |  |  |  |

| Compound | Diet | LPS | vs | Diet | LPS | Estimate | P-value |  | Compound | Diet | LPS | vs | Diet | LPS | Estimate | P-value |
| --- | --- | --- | --- | --- | --- | --- | --- | --- | --- | --- | --- | --- | --- | --- | --- | --- |
| F_8-iso-PGF2a | control | no |  | diet_1 | no | 13,875 | 0,1546 |  | F_8-iso-PGF2a | control | no |  | control | yes | -1,5536 | 0,9998 |
| F_8-iso-PGF2a | control | no |  | diet_3 | no | 21,375 | 0,0057 |  | F_8-iso-PGF2a | diet_1 | no |  | diet_1 | yes | 0,375 | 1 |
| F_8-iso-PGF2a | diet_1 | no |  | diet_3 | no | 7,5 | 0,7611 |  | F_8-iso-PGF2a | diet_3 | no |  | diet_3 | yes | -14,7857 | 0,1333 |
| F_8-iso-PGF2a | control | yes |  | diet_1 | yes | 15,8036 | 0,0917 |  |  |  |  |  |  |  |  |  |
| F_8-iso-PGF2a | control | yes |  | diet_3 | yes | 8,1429 | 0,7492 |  |  |  |  |  |  |  |  |  |
| F_8-iso-PGF2a | diet_1 | yes |  | diet_3 | yes | -7,6607 | 0,7709 |  |  |  |  |  |  |  |  |  |
|  |  |  |  |  |  |  |  |  |  |  |  |  |  |  |  |  |
|  |  |  |  |  |  |  |  |  |  |  |  |  |  |  |  |  |
| F_PGF2a | control | no |  | diet_1 | no | 12,9992 | 0,5072 |  | F_PGF2a | control | no |  | control | yes | -14,6142 | 0,4038 |
| F_PGF2a | control | no |  | diet_3 | no | 8,7998 | 0,823 |  | F_PGF2a | diet_1 | no |  | diet_1 | yes | -18,2105 | 0,1999 |
| F_PGF2a | diet_1 | no |  | diet_3 | no | -4,1994 | 0,9906 |  | F_PGF2a | diet_3 | no |  | diet_3 | yes | -10,3418 | 0,723 |
| F_PGF2a | control | yes |  | diet_1 | yes | 9,4029 | 0,7902 |  |  |  |  |  |  |  |  |  |
| F_PGF2a | control | yes |  | diet_3 | yes | 13,0722 | 0,5252 |  |  |  |  |  |  |  |  |  |
| F_PGF2a | diet_1 | yes |  | diet_3 | yes | 3,6692 | 0,9952 |  |  |  |  |  |  |  |  |  |
|  |  |  |  |  |  |  |  |  |  |  |  |  |  |  |  |  |
|  |  |  |  |  |  |  |  |  |  |  |  |  |  |  |  |  |
| F_10(S)-17(S)-DiHDoHE | control | no |  | diet_1 | no | -4,1188 | 0,9644 |  | F_10(S)-17(S)-DiHDoHE | control | no |  | control | yes | -1,3297 | 0,9998 |
| F_10(S)-17(S)-DiHDoHE | control | no |  | diet_3 | no | -16,1532 | 0,0875 |  | F_10(S)-17(S)-DiHDoHE | diet_1 | no |  | diet_1 | yes | -17,9911 | 0,0507 |
| F_10(S)-17(S)-DiHDoHE | diet_1 | no |  | diet_3 | no | -12,0344 | 0,2763 |  | F_10(S)-17(S)-DiHDoHE | diet_3 | no |  | diet_3 | yes | -10,2528 | 0,4449 |
| F_10(S)-17(S)-DiHDoHE | control | yes |  | diet_1 | yes | -20,7802 | 0,0251 |  |  |  |  |  |  |  |  |  |
| F_10(S)-17(S)-DiHDoHE | control | yes |  | diet_3 | yes | -25,0763 | 0,0084 |  |  |  |  |  |  |  |  |  |
| F_10(S)-17(S)-DiHDoHE | diet_1 | yes |  | diet_3 | yes | -4,2961 | 0,9612 |  |  |  |  |  |  |  |  |  |

| Compound | Diet | LPS | vs | Diet | LPS | Estimate | P-value |  | Compound | Diet | LPS | vs | Diet | LPS | Estimate | P-value |
| --- | --- | --- | --- | --- | --- | --- | --- | --- | --- | --- | --- | --- | --- | --- | --- | --- |
| F_19,20-DiHoPE | control | no |  | diet_1 | no | -10,375 | 0,0603 |  | F_19,20-DiHoPE | control | no |  | control | yes | -0,8036 | 0,9999 |
| F_19,20-DiHoPE | control | no |  | diet_3 | no | -18,375 | <.0001 |  | F_19,20-DiHoPE | diet_1 | no |  | diet_1 | yes | -10,75 | 0,0469 |
| F_19,20-DiHoPE | diet_1 | no |  | diet_3 | no | -8 | 0,24 |  | F_19,20-DiHoPE | diet_3 | no |  | diet_3 | yes | -15 | 0,0028 |
| F_19,20-DiHoPE | control | yes |  | diet_1 | yes | -20,3214 | <.0001 |  |  |  |  |  |  |  |  |  |
| F_19,20-DiHoPE | control | yes |  | diet_3 | yes | -32,5714 | <.0001 |  |  |  |  |  |  |  |  |  |
| F_19,20-DiHoPE | diet_1 | yes |  | diet_3 | yes | -12,25 | 0,0219 |  |  |  |  |  |  |  |  |  |
|  |  |  |  |  |  |  |  |  |  |  |  |  |  |  |  |  |
|  |  |  |  |  |  |  |  |  |  |  |  |  |  |  |  |  |
| F_TBXB3 | control | no |  | diet_1 | no | -7,4905 | 0,6018 |  | F_TBXB3 | control | no |  | control | yes | -4,5598 | 0,918 |
| F_TBXB3 | control | no |  | diet_3 | no | -15,5467 | 0,0494 |  | F_TBXB3 | diet_1 | no |  | diet_1 | yes | -17,6917 | 0,0226 |
| F_TBXB3 | diet_1 | no |  | diet_3 | no | -8,0562 | 0,532 |  | F_TBXB3 | diet_3 | no |  | diet_3 | yes | -17,4961 | 0,0264 |
| F_TBXB3 | control | yes |  | diet_1 | yes | -20,6224 | 0,0086 |  |  |  |  |  |  |  |  |  |
| F_TBXB3 | control | yes |  | diet_3 | yes | -28,4829 | 0,0007 |  |  |  |  |  |  |  |  |  |
| F_TBXB3 | diet_1 | yes |  | diet_3 | yes | -7,8605 | 0,5688 |  |  |  |  |  |  |  |  |  |
|  |  |  |  |  |  |  |  |  |  |  |  |  |  |  |  |  |
|  |  |  |  |  |  |  |  |  |  |  |  |  |  |  |  |  |
| F_TBXB2 | control | no |  | diet_1 | no | 8,3746 | 0,8674 |  | F_TBXB2 | control | no |  | control | yes | -16,2748 | 0,3288 |
| F_TBXB2 | control | no |  | diet_3 | no | 6,6008 | 0,9452 |  | F_TBXB2 | diet_1 | no |  | diet_1 | yes | -21,1407 | 0,1248 |
| F_TBXB2 | diet_1 | no |  | diet_3 | no | -1,7737 | 0,9999 |  | F_TBXB2 | diet_3 | no |  | diet_3 | yes | -18,5904 | 0,2146 |
| F_TBXB2 | control | yes |  | diet_1 | yes | 3,5086 | 0,9967 |  |  |  |  |  |  |  |  |  |
| F_TBXB2 | control | yes |  | diet_3 | yes | 4,2852 | 0,9918 |  |  |  |  |  |  |  |  |  |
| F_TBXB2 | diet_1 | yes |  | diet_3 | yes | 0,7766 | 1 |  |  |  |  |  |  |  |  |  |
|  |  |  |  |  |  |  |  |  |  |  |  |  |  |  |  |  |
|  |  |  |  |  |  |  |  |  |  |  |  |  |  |  |  |  |
| F_8,9-DiHETrE | control | no |  | diet_1 | no | 0,279 | 0,0044 |  | F_8,9-DiHETrE | control | no |  | control | yes | 0,1044 | 0,4905 |
| F_8,9-DiHETrE | control | no |  | diet_3 | no | 0,2935 | 0,003 |  | F_8,9-DiHETrE | diet_1 | no |  | diet_1 | yes | -0,05637 | 0,9077 |
| F_8,9-DiHETrE | diet_1 | no |  | diet_3 | no | 0,0145 | 0,9998 |  | F_8,9-DiHETrE | diet_3 | no |  | diet_3 | yes | -0,2495 | 0,0111 |
| F_8,9-DiHETrE | control | yes |  | diet_1 | yes | 0,1183 | 0,3687 |  |  |  |  |  |  |  |  |  |
| F_8,9-DiHETrE | control | yes |  | diet_3 | yes | -0,06039 | 0,8957 |  |  |  |  |  |  |  |  |  |
| F_8,9-DiHETrE | diet_1 | yes |  | diet_3 | yes | -0,1786 | 0,0797 |  |  |  |  |  |  |  |  |  |

| Compound | Diet | LPS | vs | Diet | LPS | Estimate | P-value |  | Compound | Diet | LPS | vs | Diet | LPS | Estimate | P-value |
| --- | --- | --- | --- | --- | --- | --- | --- | --- | --- | --- | --- | --- | --- | --- | --- | --- |
| F_13,14-dihydro-15-keto-PGF2a | control | no |  | diet_1 | no | 0,1725 | 0,8411 |  | F_13,14-dihydro-15-keto-PGF2a | control | no |  | control | yes | -0,3248 | 0,311 |
| F_13,14-dihydro-15-keto-PGF2a | control | no |  | diet_3 | no | 0,209 | 0,7154 |  | F_13,14-dihydro-15-keto-PGF2a | diet_1 | no |  | diet_1 | yes | -0,06717 | 0,9968 |
| F_13,14-dihydro-15-keto-PGF2a | diet_1 | no |  | diet_3 | no | 0,03644 | 0,9998 |  | F_13,14-dihydro-15-keto-PGF2a | diet_3 | no |  | diet_3 | yes | -0,05741 | 0,9986 |
| F_13,14-dihydro-15-keto-PGF2a | control | yes |  | diet_1 | yes | 0,4302 | 0,1081 |  |  |  |  |  |  |  |  |  |
| F_13,14-dihydro-15-keto-PGF2a | control | yes |  | diet_3 | yes | 0,4764 | 0,0685 |  |  |  |  |  |  |  |  |  |
| F_13,14-dihydro-15-keto-PGF2a | diet_1 | yes |  | diet_3 | yes | 0,04619 | 0,9995 |  |  |  |  |  |  |  |  |  |
|  |  |  |  |  |  |  |  |  |  |  |  |  |  |  |  |  |
| F_lipoxin A4 | control | no |  | diet_1 | no | -7,11E-15 | 1 |  | F_lipoxin A4 | control | no |  | control | yes | -5,33E-15 | 1 |
| F_lipoxin A4 | control | no |  | diet_3 | no | -12,9471 | 0,0074 |  | F_lipoxin A4 | diet_1 | no |  | diet_1 | yes | -2,0688 | 0,9797 |
| F_lipoxin A4 | diet_1 | no |  | diet_3 | no | -12,9471 | 0,0074 |  | F_lipoxin A4 | diet_3 | no |  | diet_3 | yes | -12,4126 | 0,0124 |
| F_lipoxin A4 | control | yes |  | diet_1 | yes | -2,0688 | 0,9818 |  |  |  |  |  |  |  |  |  |
| F_lipoxin A4 | control | yes |  | diet_3 | yes | -25,3597 | <.0001 |  |  |  |  |  |  |  |  |  |
| F_lipoxin A4 | diet_1 | yes |  | diet_3 | yes | -23,2909 | <.0001 |  |  |  |  |  |  |  |  |  |
|  |  |  |  |  |  |  |  |  |  |  |  |  |  |  |  |  |
| F_PGE2 | control | no |  | diet_1 | no | 14,6419 | 0,2767 |  | F_PGE2 | control | no |  | control | yes | -15,4528 | 0,2378 |
| F_PGE2 | control | no |  | diet_3 | no | 9,0737 | 0,7217 |  | F_PGE2 | diet_1 | no |  | diet_1 | yes | -24,3739 | 0,0251 |
| F_PGE2 | diet_1 | no |  | diet_3 | no | -5,5682 | 0,9479 |  | F_PGE2 | diet_3 | no |  | diet_3 | yes | -18,1006 | 0,1286 |
| F_PGE2 | control | yes |  | diet_1 | yes | 5,7208 | 0,9438 |  |  |  |  |  |  |  |  |  |
| F_PGE2 | control | yes |  | diet_3 | yes | 6,4259 | 0,9148 |  |  |  |  |  |  |  |  |  |
| F_PGE2 | diet_1 | yes |  | diet_3 | yes | 0,7051 | 1 |  |  |  |  |  |  |  |  |  |
|  |  |  |  |  |  |  |  |  |  |  |  |  |  |  |  |  |
| F_PGD2 | control | no |  | diet_1 | no | 13,8957 | 0,5481 |  | F_PGD2 | control | no |  | control | yes | -5,3312 | 0,9843 |
| F_PGD2 | control | no |  | diet_3 | no | 13,1999 | 0,597 |  | F_PGD2 | diet_1 | no |  | diet_1 | yes | -13,5673 | 0,5711 |
| F_PGD2 | diet_1 | no |  | diet_3 | no | -0,6958 | 1 |  | F_PGD2 | diet_3 | no |  | diet_3 | yes | -15,8887 | 0,4279 |
| F_PGD2 | control | yes |  | diet_1 | yes | 5,6596 | 0,9797 |  |  |  |  |  |  |  |  |  |
| F_PGD2 | control | yes |  | diet_3 | yes | 2,6424 | 0,9994 |  |  |  |  |  |  |  |  |  |
| F_PGD2 | diet_1 | yes |  | diet_3 | yes | -3,0172 | 0,9989 |  |  |  |  |  |  |  |  |  |

| Compound | Diet | LPS | vs | Diet | LPS | Estimate | P-value |  | Compound | Diet | LPS | vs | Diet | LPS | Estimate | P-value |
| --- | --- | --- | --- | --- | --- | --- | --- | --- | --- | --- | --- | --- | --- | --- | --- | --- |
| F_Leukotriene E4 | control | no |  | diet_1 | no | 4,1533 | 0,9807 |  | F_Leukotriene E4 | control | no |  | control | yes | -2,8613 | 0,9965 |
| F_Leukotriene E4 | control | no |  | diet_3 | no | 4,1533 | 0,9807 |  | F_Leukotriene E4 | diet_1 | no |  | diet_1 | yes | 0 | 1 |
| F_Leukotriene E4 | diet_1 | no |  | diet_3 | no | 6,39E-14 | 1 |  | F_Leukotriene E4 | diet_3 | no |  | diet_3 | yes | -11,6966 | 0,4432 |
| F_Leukotriene E4 | control | yes |  | diet_1 | yes | 7,0146 | 0,8535 |  |  |  |  |  |  |  |  |  |
| F_Leukotriene E4 | control | yes |  | diet_3 | yes | -4,682 | 0,9693 |  |  |  |  |  |  |  |  |  |
| F_Leukotriene E4 | diet_1 | yes |  | diet_3 | yes | -11,6966 | 0,4432 |  |  |  |  |  |  |  |  |  |
|  |  |  |  |  |  |  |  |  |  |  |  |  |  |  |  |  |
|  |  |  |  |  |  |  |  |  |  |  |  |  |  |  |  |  |
| F_Leukotriene D4 | control | no |  | diet_1 | no | 4,1738 | 0,9579 |  | F_Leukotriene D4 | control | no |  | control | yes | -6,038 | 0,8437 |
| F_Leukotriene D4 | control | no |  | diet_3 | no | 4,1738 | 0,9579 |  | F_Leukotriene D4 | diet_1 | no |  | diet_1 | yes | -12,52 | 0,2166 |
| F_Leukotriene D4 | diet_1 | no |  | diet_3 | no | 3,55E-15 | 1 |  | F_Leukotriene D4 | diet_3 | no |  | diet_3 | yes | -24,8837 | 0,0053 |
| F_Leukotriene D4 | control | yes |  | diet_1 | yes | -2,3082 | 0,9971 |  |  |  |  |  |  |  |  |  |
| F_Leukotriene D4 | control | yes |  | diet_3 | yes | -14,672 | 0,1318 |  |  |  |  |  |  |  |  |  |
| F_Leukotriene D4 | diet_1 | yes |  | diet_3 | yes | -12,3638 | 0,239 |  |  |  |  |  |  |  |  |  |
|  |  |  |  |  |  |  |  |  |  |  |  |  |  |  |  |  |
|  |  |  |  |  |  |  |  |  |  |  |  |  |  |  |  |  |
| F_17 keto- 4(z), 7(z), 10(z), 13 (z), 15 (E), 19(z)-DHA | control | no |  | diet_1 | no | 1,78E-15 | 1 |  | F_17 keto- 4(z), 7(z), 10(z), 13 (z), 15 (E), 19(z)-DHA | control | no |  | control | yes | -3,55E-15 | 1 |
| F_17 keto- 4(z), 7(z), 10(z), 13 (z), 15 (E), 19(z)-DHA | control | no |  | diet_3 | no | 0 | 1 |  | F_17 keto- 4(z), 7(z), 10(z), 13 (z), 15 (E), 19(z)-DHA | diet_1 | no |  | diet_1 | yes | -10,0956 | 0,0208 |
| F_17 keto- 4(z), 7(z), 10(z), 13 (z), 15 (E), 19(z)-DHA | diet_1 | no |  | diet_3 | no | -1,78E-15 | 1 |  | F_17 keto- 4(z), 7(z), 10(z), 13 (z), 15 (E), 19(z)-DHA | diet_3 | no |  | diet_3 | yes | -24,5611 | <.0001 |
| F_17 keto- 4(z), 7(z), 10(z), 13 (z), 15 (E), 19(z)-DHA | control | yes |  | diet_1 | yes | -10,0956 | 0,0251 |  |  |  |  |  |  |  |  |  |
| F_17 keto- 4(z), 7(z), 10(z), 13 (z), 15 (E), 19(z)-DHA | control | yes |  | diet_3 | yes | -24,5611 | <.0001 |  |  |  |  |  |  |  |  |  |
| F_17 keto- 4(z), 7(z), 10(z), 13 (z), 15 (E), 19(z)-DHA | diet_1 | yes |  | diet_3 | yes | -14,4654 | 0,0027 |  |  |  |  |  |  |  |  |  |

| Compound | Diet | LPS | vs | Diet | LPS | Estimate | P-value |  | Compound | Diet | LPS | vs | Diet | LPS | Estimate | P-value |
| --- | --- | --- | --- | --- | --- | --- | --- | --- | --- | --- | --- | --- | --- | --- | --- | --- |
| F_12,13-DiHOME | control | no |  | diet_1 | no | 760,84 | 0,0026 |  | F_12,13-DiHOME | control | no |  | control | yes | 350,47 | 0,466 |
| F_12,13-DiHOME | control | no |  | diet_3 | no | 811,89 | 0,0012 |  | F_12,13-DiHOME | diet_1 | no |  | diet_1 | yes | -402,77 | 0,2774 |
| F_12,13-DiHOME | diet_1 | no |  | diet_3 | no | 51,0585 | 0,9998 |  | F_12,13-DiHOME | diet_3 | no |  | diet_3 | yes | -1585,7 | <.0001 |
| F_12,13-DiHOME | control | yes |  | diet_1 | yes | 7,5902 | 1 |  |  |  |  |  |  |  |  |  |
| F_12,13-DiHOME | control | yes |  | diet_3 | yes | -1124,28 | <.0001 |  |  |  |  |  |  |  |  |  |
| F_12,13-DiHOME | diet_1 | yes |  | diet_3 | yes | -1131,87 | <.0001 |  |  |  |  |  |  |  |  |  |
|  |  |  |  |  |  |  |  |  |  |  |  |  |  |  |  |  |
|  |  |  |  |  |  |  |  |  |  |  |  |  |  |  |  |  |
| F_9,10-DiHOME | control | no |  | diet_1 | no | 301,41 | 0,0107 |  | F_9,10-DiHOME | control | no |  | control | yes | 77,7237 | 0,8656 |
| F_9,10-DiHOME | control | no |  | diet_3 | no | 369,54 | 0,0025 |  | F_9,10-DiHOME | diet_1 | no |  | diet_1 | yes | -164,35 | 0,2299 |
| F_9,10-DiHOME | diet_1 | no |  | diet_3 | no | 68,1355 | 0,9051 |  | F_9,10-DiHOME | diet_3 | no |  | diet_3 | yes | -520,54 | 0,0002 |
| F_9,10-DiHOME | control | yes |  | diet_1 | yes | 59,3295 | 0,9506 |  |  |  |  |  |  |  |  |  |
| F_9,10-DiHOME | control | yes |  | diet_3 | yes | -228,73 | 0,0767 |  |  |  |  |  |  |  |  |  |
| F_9,10-DiHOME | diet_1 | yes |  | diet_3 | yes | -288,06 | 0,0178 |  |  |  |  |  |  |  |  |  |
|  |  |  |  |  |  |  |  |  |  |  |  |  |  |  |  |  |
|  |  |  |  |  |  |  |  |  |  |  |  |  |  |  |  |  |
| F_9,12,13-TriHOME | control | no |  | diet_1 | no | 6210,41 | 0,0743 |  | F_9,12,13-TriHOME | control | no |  | control | yes | 2754,85 | 0,7356 |
| F_9,12,13-TriHOME | control | no |  | diet_3 | no | 5144,86 | 0,1726 |  | F_9,12,13-TriHOME | diet_1 | no |  | diet_1 | yes | -4225,05 | 0,3331 |
| F_9,12,13-TriHOME | diet_1 | no |  | diet_3 | no | -1065,55 | 0,9931 |  | F_9,12,13-TriHOME | diet_3 | no |  | diet_3 | yes | -1589,54 | 0,9621 |
| F_9,12,13-TriHOME | control | yes |  | diet_1 | yes | -769,49 | 0,9986 |  |  |  |  |  |  |  |  |  |
| F_9,12,13-TriHOME | control | yes |  | diet_3 | yes | 800,47 | 0,9983 |  |  |  |  |  |  |  |  |  |
| F_9,12,13-TriHOME | diet_1 | yes |  | diet_3 | yes | 1569,96 | 0,964 |  |  |  |  |  |  |  |  |  |
|  |  |  |  |  |  |  |  |  |  |  |  |  |  |  |  |  |
|  |  |  |  |  |  |  |  |  |  |  |  |  |  |  |  |  |
| F_9,10,13-TriHOME | control | no |  | diet_1 | no | 3810,15 | 0,0117 |  | F_9,10,13-TriHOME | control | no |  | control | yes | 1340,84 | 0,682 |
| F_9,10,13-TriHOME | control | no |  | diet_3 | no | 3088,38 | 0,0428 |  | F_9,10,13-TriHOME | diet_1 | no |  | diet_1 | yes | -2824,58 | 0,0688 |
| F_9,10,13-TriHOME | diet_1 | no |  | diet_3 | no | -721,77 | 0,9564 |  | F_9,10,13-TriHOME | diet_3 | no |  | diet_3 | yes | -1374,79 | 0,6609 |
| F_9,10,13-TriHOME | control | yes |  | diet_1 | yes | -355,27 | 0,9984 |  |  |  |  |  |  |  |  |  |
| F_9,10,13-TriHOME | control | yes |  | diet_3 | yes | 372,75 | 0,9983 |  |  |  |  |  |  |  |  |  |
| F_9,10,13-TriHOME | diet_1 | yes |  | diet_3 | yes | 728,02 | 0,9606 |  |  |  |  |  |  |  |  |  |

| Compound | Diet | LPS | vs | Diet | LPS | Estimate | P-value |  | Compound | Diet | LPS | vs | Diet | LPS | Estimate | P-value |
| --- | --- | --- | --- | --- | --- | --- | --- | --- | --- | --- | --- | --- | --- | --- | --- | --- |
| F_UK1 | control | no |  | diet_1 | no | 14,4913 | 0,2116 |  | F_UK1 | control | no |  | control | yes | 4,1039 | 0,9796 |
| F_UK1 | control | no |  | diet_3 | no | 20,333 | 0,0478 |  | F_UK1 | diet_1 | no |  | diet_1 | yes | -13,7709 | 0,2509 |
| F_UK1 | diet_1 | no |  | diet_3 | no | 5,8417 | 0,905 |  | F_UK1 | diet_3 | no |  | diet_3 | yes | -20,3183 | 0,0569 |
| F_UK1 | control | yes |  | diet_1 | yes | -3,3835 | 0,9913 |  |  |  |  |  |  |  |  |  |
| F_UK1 | control | yes |  | diet_3 | yes | -4,0892 | 0,9824 |  |  |  |  |  |  |  |  |  |
| F_UK1 | diet_1 | yes |  | diet_3 | yes | -0,7057 | 1 |  |  |  |  |  |  |  |  |  |
|  |  |  |  |  |  |  |  |  |  |  |  |  |  |  |  |  |
|  |  |  |  |  |  |  |  |  |  |  |  |  |  |  |  |  |
| F_UK2 | control | no |  | diet_1 | no | 0,06543 | 0,1775 |  | F_UK2 | control | no |  | control | yes | -0,04496 | 0,6038 |
| F_UK2 | control | no |  | diet_3 | no | 0,09057 | 0,0212 |  | F_UK2 | diet_1 | no |  | diet_1 | yes | -0,1196 | 0,001 |
| F_UK2 | diet_1 | no |  | diet_3 | no | 0,02515 | 0,9374 |  | F_UK2 | diet_3 | no |  | diet_3 | yes | -0,2078 | <.0001 |
| F_UK2 | control | yes |  | diet_1 | yes | -0,00925 | 0,9994 |  |  |  |  |  |  |  |  |  |
| F_UK2 | control | yes |  | diet_3 | yes | -0,07225 | 0,1519 |  |  |  |  |  |  |  |  |  |
| F_UK2 | diet_1 | yes |  | diet_3 | yes | -0,06299 | 0,2424 |  |  |  |  |  |  |  |  |  |
|  |  |  |  |  |  |  |  |  |  |  |  |  |  |  |  |  |
|  |  |  |  |  |  |  |  |  |  |  |  |  |  |  |  |  |
| F_UK3 | control | no |  | diet_1 | no | 0,02591 | 0,8231 |  | F_UK3 | control | no |  | control | yes | -0,07358 | 0,0668 |
| F_UK3 | control | no |  | diet_3 | no | 0,01924 | 0,9381 |  | F_UK3 | diet_1 | no |  | diet_1 | yes | -0,1305 | 0,0015 |
| F_UK3 | diet_1 | no |  | diet_3 | no | -0,00667 | 0,9995 |  | F_UK3 | diet_3 | no |  | diet_3 | yes | -0,1291 | 0,0021 |
| F_UK3 | control | yes |  | diet_1 | yes | -0,03099 | 0,7277 |  |  |  |  |  |  |  |  |  |
| F_UK3 | control | yes |  | diet_3 | yes | -0,0363 | 0,6238 |  |  |  |  |  |  |  |  |  |
| F_UK3 | diet_1 | yes |  | diet_3 | yes | -0,00531 | 0,9998 |  |  |  |  |  |  |  |  |  |
|  |  |  |  |  |  |  |  |  |  |  |  |  |  |  |  |  |
|  |  |  |  |  |  |  |  |  |  |  |  |  |  |  |  |  |
| F_UK4 | control | no |  | diet_1 | no | 0,04343 | 0,1087 |  | F_UK4 | control | no |  | control | yes | -0,03849 | 0,2002 |
| F_UK4 | control | no |  | diet_3 | no | 0,03658 | 0,2206 |  | F_UK4 | diet_1 | no |  | diet_1 | yes | -0,07789 | 0,0023 |
| F_UK4 | diet_1 | no |  | diet_3 | no | -0,00685 | 0,997 |  | F_UK4 | diet_3 | no |  | diet_3 | yes | -0,09582 | 0,0005 |
| F_UK4 | control | yes |  | diet_1 | yes | 0,004024 | 0,9998 |  |  |  |  |  |  |  |  |  |
| F_UK4 | control | yes |  | diet_3 | yes | -0,02075 | 0,7747 |  |  |  |  |  |  |  |  |  |
| F_UK4 | diet_1 | yes |  | diet_3 | yes | -0,02478 | 0,6122 |  |  |  |  |  |  |  |  |  |

| Compound | Diet | LPS | vs | Diet | LPS | Estimate | P-value |  | Compound | Diet | LPS | vs | Diet | LPS | Estimate | P-value |
| --- | --- | --- | --- | --- | --- | --- | --- | --- | --- | --- | --- | --- | --- | --- | --- | --- |
| F_UK5 | control | no |  | diet_1 | no | 0,02922 | 0,6827 |  | F_UK5 | control | no |  | control | yes | -0,05506 | 0,1015 |
| F_UK5 | control | no |  | diet_3 | no | 0,04094 | 0,326 |  | F_UK5 | diet_1 | no |  | diet_1 | yes | -0,0774 | 0,0045 |
| F_UK5 | diet_1 | no |  | diet_3 | no | 0,01173 | 0,9911 |  | F_UK5 | diet_3 | no |  | diet_3 | yes | -0,1311 | <.0001 |
| F_UK5 | control | yes |  | diet_1 | yes | 0,006869 | 0,9994 |  |  |  |  |  |  |  |  |  |
| F_UK5 | control | yes |  | diet_3 | yes | -0,03506 | 0,5693 |  |  |  |  |  |  |  |  |  |
| F_UK5 | diet_1 | yes |  | diet_3 | yes | -0,04192 | 0,3376 |  |  |  |  |  |  |  |  |  |
|  |  |  |  |  |  |  |  |  |  |  |  |  |  |  |  |  |
|  |  |  |  |  |  |  |  |  |  |  |  |  |  |  |  |  |
| F_epea | control | no |  | diet_1 | no | -12,004 | 0,0008 |  | F_epea | control | no |  | control | yes | 8,88E-16 | 1 |
| F_epea | control | no |  | diet_3 | no | -27,2536 | <.0001 |  | F_epea | diet_1 | no |  | diet_1 | yes | -8,992 | 0,008 |
| F_epea | diet_1 | no |  | diet_3 | no | -15,2496 | <.0001 |  | F_epea | diet_3 | no |  | diet_3 | yes | -5,7418 | 0,1334 |
| F_epea | control | yes |  | diet_1 | yes | -20,996 | <.0001 |  |  |  |  |  |  |  |  |  |
| F_epea | control | yes |  | diet_3 | yes | -32,9954 | <.0001 |  |  |  |  |  |  |  |  |  |
| F_epea | diet_1 | yes |  | diet_3 | yes | -11,9994 | 0,001 |  |  |  |  |  |  |  |  |  |
|  |  |  |  |  |  |  |  |  |  |  |  |  |  |  |  |  |
|  |  |  |  |  |  |  |  |  |  |  |  |  |  |  |  |  |
| F_dhea | control | no |  | diet_1 | no | -13,875 | <.0001 |  | F_dhea | control | no |  | control | yes | -4,8214 | 0,2587 |
| F_dhea | control | no |  | diet_3 | no | -28,125 | <.0001 |  | F_dhea | diet_1 | no |  | diet_1 | yes | -10,25 | 0,0003 |
| F_dhea | diet_1 | no |  | diet_3 | no | -14,25 | <.0001 |  | F_dhea | diet_3 | no |  | diet_3 | yes | -8,125 | 0,0076 |
| F_dhea | control | yes |  | diet_1 | yes | -19,3036 | <.0001 |  |  |  |  |  |  |  |  |  |
| F_dhea | control | yes |  | diet_3 | yes | -31,4286 | <.0001 |  |  |  |  |  |  |  |  |  |
| F_dhea | diet_1 | yes |  | diet_3 | yes | -12,125 | <.0001 |  |  |  |  |  |  |  |  |  |
|  |  |  |  |  |  |  |  |  |  |  |  |  |  |  |  |  |
|  |  |  |  |  |  |  |  |  |  |  |  |  |  |  |  |  |
| F_aea | control | no |  | diet_1 | no | 2,1176 | <.0001 |  | F_aea | control | no |  | control | yes | 0,3404 | 0,5921 |
| F_aea | control | no |  | diet_3 | no | 1,9487 | <.0001 |  | F_aea | diet_1 | no |  | diet_1 | yes | 0,05905 | 0,9996 |
| F_aea | diet_1 | no |  | diet_3 | no | -0,1689 | 0,951 |  | F_aea | diet_3 | no |  | diet_3 | yes | -0,04333 | 0,9999 |
| F_aea | control | yes |  | diet_1 | yes | 1,8362 | 0,0001 |  |  |  |  |  |  |  |  |  |
| F_aea | control | yes |  | diet_3 | yes | 1,5649 | 0,0005 |  |  |  |  |  |  |  |  |  |
| F_aea | diet_1 | yes |  | diet_3 | yes | -0,2712 | 0,772 |  |  |  |  |  |  |  |  |  |

| Compound | Diet | LPS | vs | Diet | LPS | Estimate | P-value |  | Compound | Diet | LPS | vs | Diet | LPS | Estimate | P-value |
| --- | --- | --- | --- | --- | --- | --- | --- | --- | --- | --- | --- | --- | --- | --- | --- | --- |
| F_2-ag | control | no |  | diet_1 | no | 23,7314 | 0,0027 |  | F_2-ag | control | no |  | control | yes | -8,8874 | 0,4318 |
| F_2-ag | control | no |  | diet_3 | no | 16,0987 | 0,0381 |  | F_2-ag | diet_1 | no |  | diet_1 | yes | -8,7451 | 0,4254 |
| F_2-ag | diet_1 | no |  | diet_3 | no | -7,6326 | 0,558 |  | F_2-ag | diet_3 | no |  | diet_3 | yes | -15,0551 | 0,0622 |
| F_2-ag | control | yes |  | diet_1 | yes | 23,8736 | 0,0031 |  |  |  |  |  |  |  |  |  |
| F_2-ag | control | yes |  | diet_3 | yes | 9,931 | 0,3459 |  |  |  |  |  |  |  |  |  |
| F_2-ag | diet_1 | yes |  | diet_3 | yes | -13,9426 | 0,0911 |  |  |  |  |  |  |  |  |  |
|  |  |  |  |  |  |  |  |  |  |  |  |  |  |  |  |  |
|  |  |  |  |  |  |  |  |  |  |  |  |  |  |  |  |  |
| F_dle | control | no |  | diet_1 | no | 0,4495 | 0,0016 |  | F_dle | control | no |  | control | yes | 0,06429 | 0,9558 |
| F_dle | control | no |  | diet_3 | no | 0,4631 | 0,0013 |  | F_dle | diet_1 | no |  | diet_1 | yes | -0,1105 | 0,6968 |
| F_dle | diet_1 | no |  | diet_3 | no | 0,01352 | 1 |  | F_dle | diet_3 | no |  | diet_3 | yes | -0,1703 | 0,3229 |
| F_dle | control | yes |  | diet_1 | yes | 0,2748 | 0,047 |  |  |  |  |  |  |  |  |  |
| F_dle | control | yes |  | diet_3 | yes | 0,2285 | 0,1269 |  |  |  |  |  |  |  |  |  |
| F_dle | diet_1 | yes |  | diet_3 | yes | -0,04626 | 0,9891 |  |  |  |  |  |  |  |  |  |
|  |  |  |  |  |  |  |  |  |  |  |  |  |  |  |  |  |
|  |  |  |  |  |  |  |  |  |  |  |  |  |  |  |  |  |
| F_pea | control | no |  | diet_1 | no | 6,75 | 0,5433 |  | F_pea | control | no |  | control | yes | -6,4286 | 0,6292 |
| F_pea | control | no |  | diet_3 | no | 4,5 | 0,8661 |  | F_pea | diet_1 | no |  | diet_1 | yes | -23 | <.0001 |
| F_pea | diet_1 | no |  | diet_3 | no | -2,25 | 0,9928 |  | F_pea | diet_3 | no |  | diet_3 | yes | -28,5 | <.0001 |
| F_pea | control | yes |  | diet_1 | yes | -9,8214 | 0,187 |  |  |  |  |  |  |  |  |  |
| F_pea | control | yes |  | diet_3 | yes | -17,5714 | 0,0023 |  |  |  |  |  |  |  |  |  |
| F_pea | diet_1 | yes |  | diet_3 | yes | -7,75 | 0,4291 |  |  |  |  |  |  |  |  |  |
|  |  |  |  |  |  |  |  |  |  |  |  |  |  |  |  |  |
|  |  |  |  |  |  |  |  |  |  |  |  |  |  |  |  |  |
| F_oea | control | no |  | diet_1 | no | 10 | 0,0076 |  | F_oea | control | no |  | control | yes | -9,7857 | 0,0132 |
| F_oea | control | no |  | diet_3 | no | 16 | <.0001 |  | F_oea | diet_1 | no |  | diet_1 | yes | -23,875 | <.0001 |
| F_oea | diet_1 | no |  | diet_3 | no | 6 | 0,2473 |  | F_oea | diet_3 | no |  | diet_3 | yes | -33,2143 | <.0001 |
| F_oea | control | yes |  | diet_1 | yes | -4,0893 | 0,6859 |  |  |  |  |  |  |  |  |  |
| F_oea | control | yes |  | diet_3 | yes | -7,4286 | 0,1255 |  |  |  |  |  |  |  |  |  |
| F_oea | diet_1 | yes |  | diet_3 | yes | -3,3393 | 0,8348 |  |  |  |  |  |  |  |  |  |

| Compound | Diet | LPS | vs | Diet | LPS | Estimate | P-value |  | Compound | Diet | LPS | vs | Diet | LPS | Estimate | P-value |
| --- | --- | --- | --- | --- | --- | --- | --- | --- | --- | --- | --- | --- | --- | --- | --- | --- |
| F_sea | control | no |  | diet_1 | no | -5,9623 | 0,4695 |  | F_sea | control | no |  | control | yes | 6,6085 | 0,3853 |
| F_sea | control | no |  | diet_3 | no | -14,4941 | 0,0073 |  | F_sea | diet_1 | no |  | diet_1 | yes | 8,5833 | 0,1531 |
| F_sea | diet_1 | no |  | diet_3 | no | -8,5318 | 0,1569 |  | F_sea | diet_3 | no |  | diet_3 | yes | 11,9247 | 0,0311 |
| F_sea | control | yes |  | diet_1 | yes | -3,9875 | 0,8195 |  |  |  |  |  |  |  |  |  |
| F_sea | control | yes |  | diet_3 | yes | -9,1779 | 0,1337 |  |  |  |  |  |  |  |  |  |
| F_sea | diet_1 | yes |  | diet_3 | yes | -5,1903 | 0,6205 |  |  |  |  |  |  |  |  |  |
|  |  |  |  |  |  |  |  |  |  |  |  |  |  |  |  |  |
|  |  |  |  |  |  |  |  |  |  |  |  |  |  |  |  |  |
| F_12(S)-HEPE | control | no |  | diet_1 | no | -12,6531 | 0,1052 |  | F_12(S)-HEPE | control | no |  | control | yes | -0,6253 | 1 |
| F_12(S)-HEPE | control | no |  | diet_3 | no | -26,0715 | 0,0008 |  | F_12(S)-HEPE | diet_1 | no |  | diet_1 | yes | -9,5102 | 0,3079 |
| F_12(S)-HEPE | diet_1 | no |  | diet_3 | no | -13,4184 | 0,0793 |  | F_12(S)-HEPE | diet_3 | no |  | diet_3 | yes | -5,5219 | 0,798 |
| F_12(S)-HEPE | control | yes |  | diet_1 | yes | -21,5379 | 0,0041 |  |  |  |  |  |  |  |  |  |
| F_12(S)-HEPE | control | yes |  | diet_3 | yes | -30,968 | 0,0002 |  |  |  |  |  |  |  |  |  |
| F_12(S)-HEPE | diet_1 | yes |  | diet_3 | yes | -9,4301 | 0,3253 |  |  |  |  |  |  |  |  |  |
|  |  |  |  |  |  |  |  |  |  |  |  |  |  |  |  |  |
|  |  |  |  |  |  |  |  |  |  |  |  |  |  |  |  |  |
| F_5(S)-HEPE | control | no |  | diet_1 | no | -13,3062 | 0,0031 |  | F_5(S)-HEPE | control | no |  | control | yes | -1,0233 | 0,9984 |
| F_5(S)-HEPE | control | no |  | diet_3 | no | -21,8757 | <.0001 |  | F_5(S)-HEPE | diet_1 | no |  | diet_1 | yes | -11,7949 | 0,0076 |
| F_5(S)-HEPE | diet_1 | no |  | diet_3 | no | -8,5695 | 0,0556 |  | F_5(S)-HEPE | diet_3 | no |  | diet_3 | yes | -13,4315 | 0,0034 |
| F_5(S)-HEPE | control | yes |  | diet_1 | yes | -24,0778 | <.0001 |  |  |  |  |  |  |  |  |  |
| F_5(S)-HEPE | control | yes |  | diet_3 | yes | -34,284 | <.0001 |  |  |  |  |  |  |  |  |  |
| F_5(S)-HEPE | diet_1 | yes |  | diet_3 | yes | -10,2061 | 0,0227 |  |  |  |  |  |  |  |  |  |
