# Supplementary material for: Fish oil and inflammatory status alter the n-3 to n-6 balance of the endocannabinoid and oxylipin metabolomes in mouse plasma and tissues
Source: Metabolomics. 2012 Apr 11;8(6):1130–47. doi: 10.1007/s11306-012-0421-9 (PMC3483099; doi:10.1007/s11306-012-0421-9)
Supplement: Supplementary file 7 — Supplementary material 7 (DOCX 17 kb) [file 11306_2012_421_MOESM7_ESM.docx]

S-7

Rank-transformed variabeles, split per compartment

| Plasma |
| --- |
| P_11,12-DiHETrE |
| P_12(S)-HEPE |
| P_12,13-DiHOME |
| P_13(S)-HODE |
| P_13,14-dihydro-15-keto-PGE2 |
| P_14,15 EET |
| P_14,15-DiHETrE |
| P_17(S)-HDoHE |
| P_19,20-DiHoPE |
| P_2-AG |
| P_5(S)-HEPE |
| P_5(S)-HETE |
| P_5,6 EET |
| P_8,9-DiHETrE |
| P_9(S)-HODE |
| P_9,10,13-TriHOME |
| P_9,10-DiHOME |
| P_9,12,13-TriHOME |
| P_DHEA |
| P_DLE |
| P_EPA |
| P_EPEA |
| P_OEA |
| P_PEA |
| P_PGB2 |
| P_PGE2 |
| P_PGE3 |
| P_SEA |
| P_TBXB3 |
| P_UK1 |

| Liver |
| --- |
| L_11(S)-HETE |
| L_11,12-DiHETrE |
| L_12(S)-HEPE |
| L_12(S)-HETE |
| L_12(S)-HHTrE |
| L_12,13-DiHOME |
| L_13(S)-HODE |
| L_13,14-dihydro-15-keto-PGE2 |
| L_13,14-dihydro-15-keto-PGF2a |
| L_14,15 EET |
| L_14,15-DiHETrE |
| L_15(S)-HETE |
| L_20(S)-HETE |
| L_2-ag |
| L_5(S)-HETE |
| L_5,6 EET |
| L_5,6-DiHETrE |
| L_8,9-DiHETrE |
| L_9(S)-HODE |
| L_9,10,13-TriHOME |
| L_9,12,13-TriHOME |
| L_AA |
| L_aea |
| L_dhea |
| L_dle |
| L_EPA |
| L_epea |
| L_LTB4 |
| L_n-acetyl leukotriene E4 |
| L_PGB2 |
| L_PGD2 |
| L_PGE2 |
| L_PGF2a |
| L_TBXB2 |
| L_UK1 |
| L_UK2 |
| L_UK3 |
| L_UK4 |
| L_UK5 |

| Ileum |
| --- |
| I_11,12 EET |
| I_11,12-DiHETrE |
| I_12(S)-HHTrE |
| I_12,13-DiHOME |
| I_13,14-dihydro-15-keto-PGD2 |
| I_13,14-dihydro-15-keto-PGE2 |
| I_13,14-dihydro-15-keto-PGF2a |
| I_14,15 EET |
| I_14,15-DiHETrE |
| I_15-deoxy-d-12,14-PGJ2 |
| I_17 keto- 4(z), 7(z), 10(z), 13 (z), 15 (E), 19(z)-DHA |
| I_17(S)-HDoHE |
| I_19,20-DiHoPE |
| I_2-ag |
| I_5(S)-HEPE |
| I_5,6 EET |
| I_5,6-DiHETrE |
| I_8,9 EET |
| I_8,9-DiHETrE |
| I_8-iso-PGF2a |
| I_9,10,13-TriHOME |
| I_9,10-DiHOME |
| I_9,12,13-TriHOME |
| I_aea |
| I_DHA |
| I_dhea |
| I_dle |
| I_EPA |
| I_epea |
| I_Leukotriene D4 |
| I_Leukotriene E4 |
| I_lipoxin A4 |
| I_LTB4 |
| I_n-acetyl leukotriene E4 |
| I_oea |
| I_pea |
| I_PGD2 |
| I_PGD3 |
| I_PGE3 |
| I_PGF2a |
| I_TBXB3 |
| I_UK2 |
| I_UK5 |

| Adipose tissue |
| --- |
| F_10(S)-17(S)-DiHDoHE |
| F_11(S)-HETE |
| F_12(S)-HEPE |
| F_12(S)-HETE |
| F_12(S)-HHTrE |
| F_13(S)-HODE |
| F_13,14-dihydro-15-keto-PGE2 |
| F_15(S)-HETE |
| F_17 keto- 4(z), 7(z), 10(z), 13 (z), 15 (E), 19(z)-DHA |
| F_17(S)-HDoHE |
| F_19,20-DiHoPE |
| F_2-ag |
| F_5(S)-HEPE |
| F_5(S)-HETE |
| F_5,6-DiHETrE |
| F_8-iso-PGF2a |
| F_9(S)-HODE |
| F_dhea |
| F_EPA |
| F_epea |
| F_Leukotriene D4 |
| F_Leukotriene E4 |
| F_lipoxin A4 |
| F_LTB4 |
| F_oea |
| F_pea |
| F_PGD2 |
| F_PGD3 |
| F_PGE2 |
| F_PGE3 |
| F_PGF2a |
| F_TBXB2 |
| F_TBXB3 |
| F_UK1 |
