# Supplementary material for: Fish oil and inflammatory status alter the n-3 to n-6 balance of the endocannabinoid and oxylipin metabolomes in mouse plasma and tissues
Source: Metabolomics. 2012 Apr 11;8(6):1130–47. doi: 10.1007/s11306-012-0421-9 (PMC3483099; doi:10.1007/s11306-012-0421-9)

## Slide 1
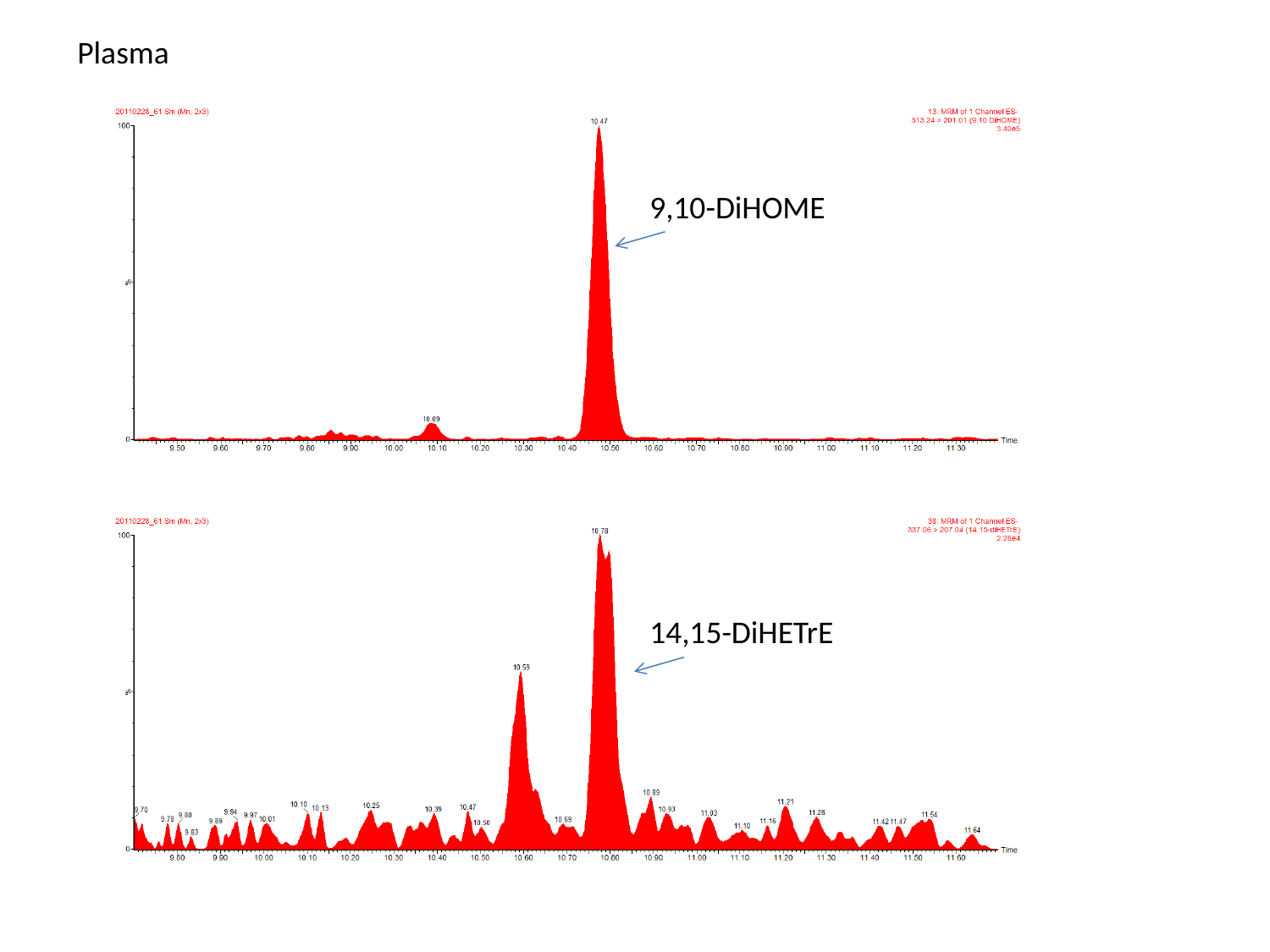

Plasma
9,10-DiHOME
14,15-DiHETrE

## Slide 2
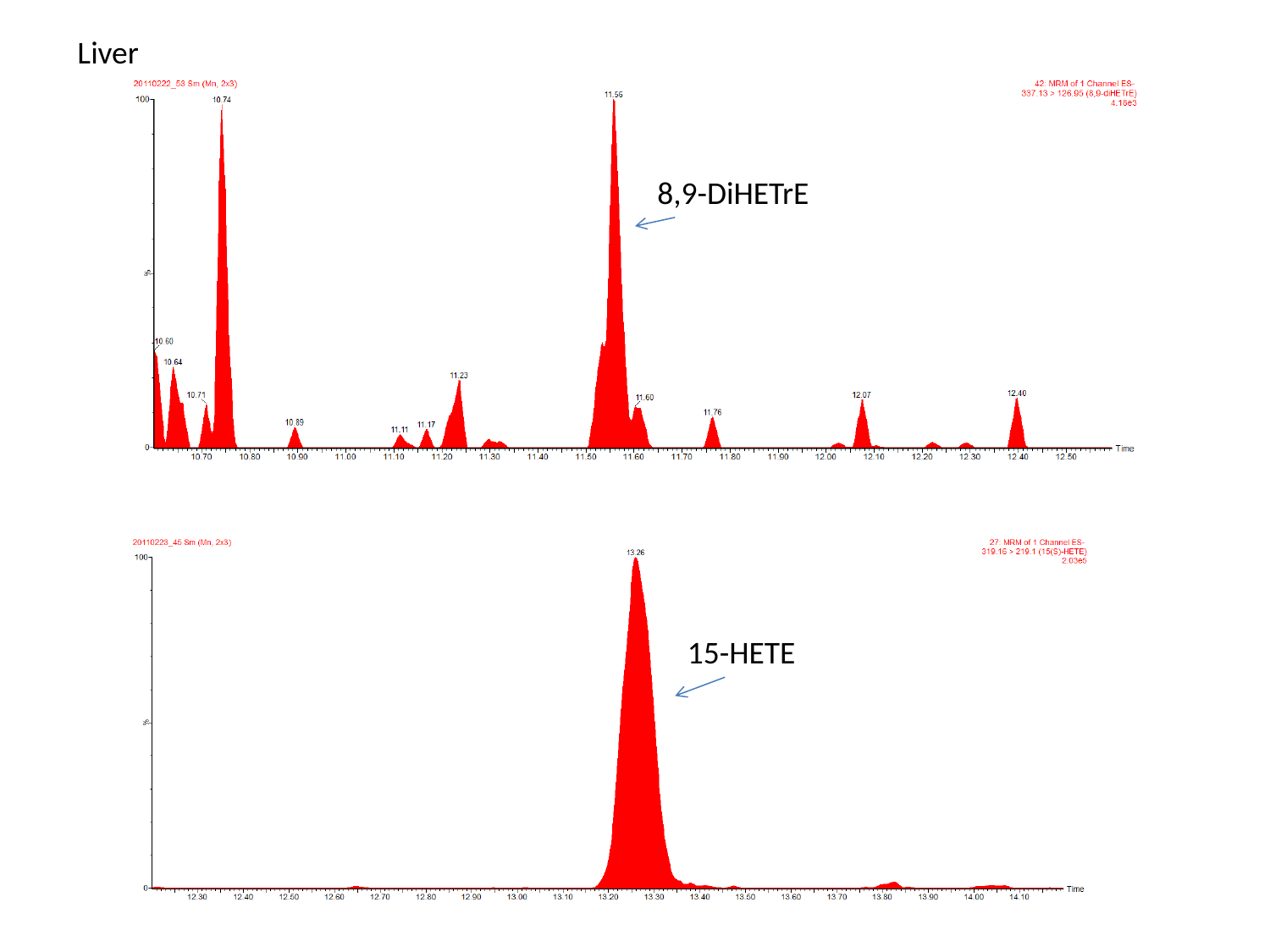

Liver
8,9-DiHETrE
15-HETE

## Slide 3
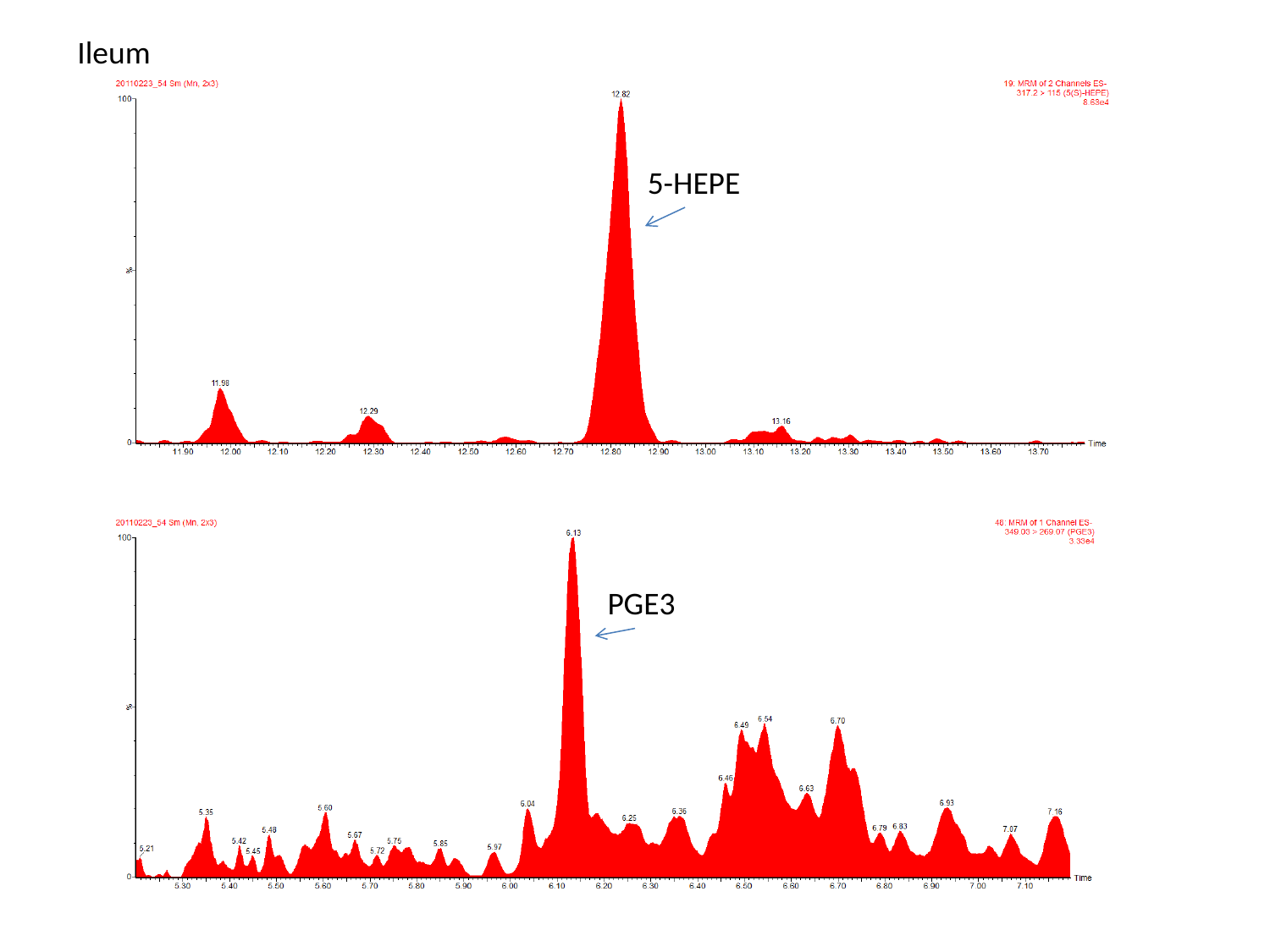

Ileum
5-HEPE
PGE3

## Slide 4
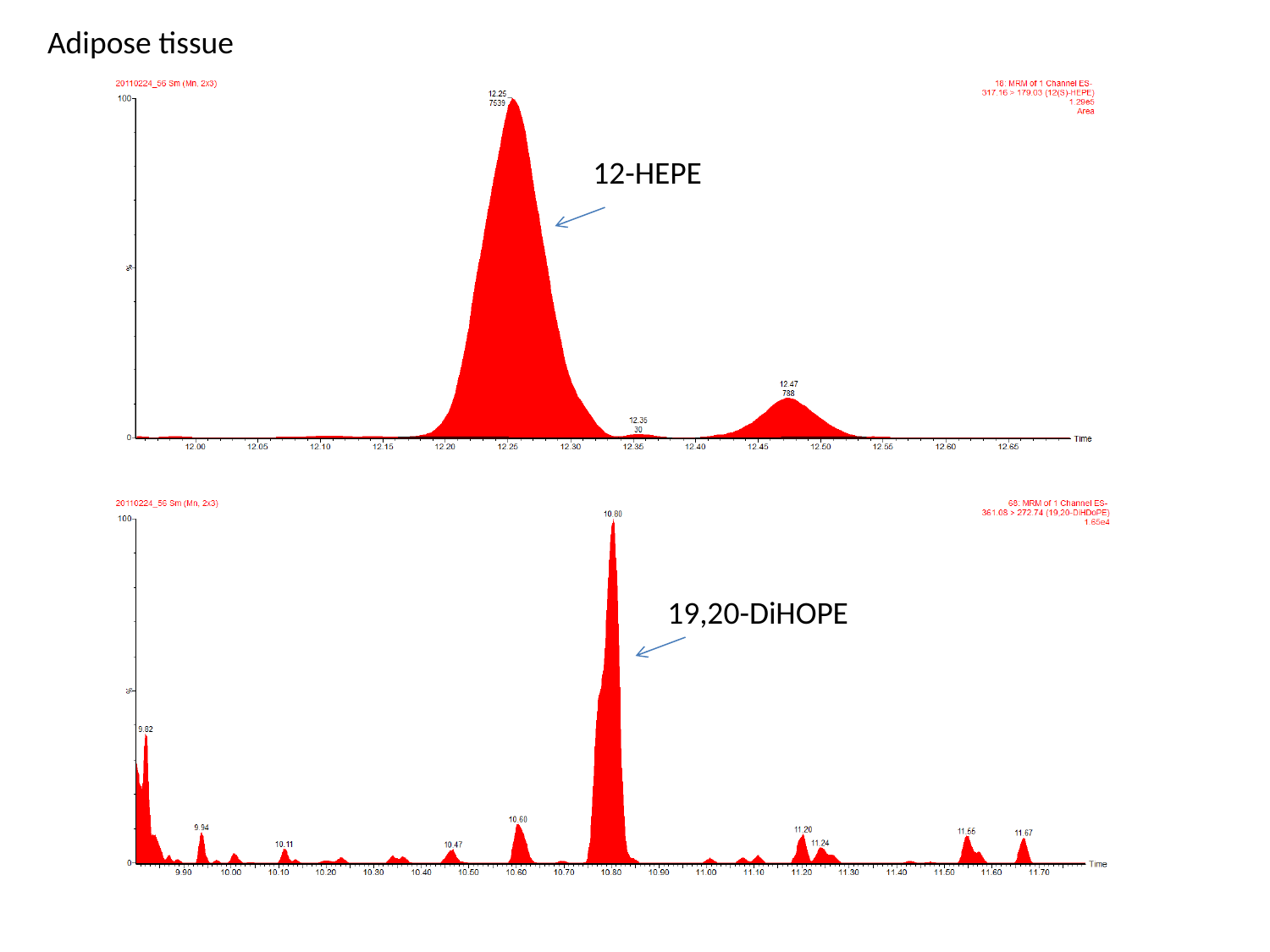

Adipose tissue
12-HEPE
19,20-DiHOPE

## Slide 5
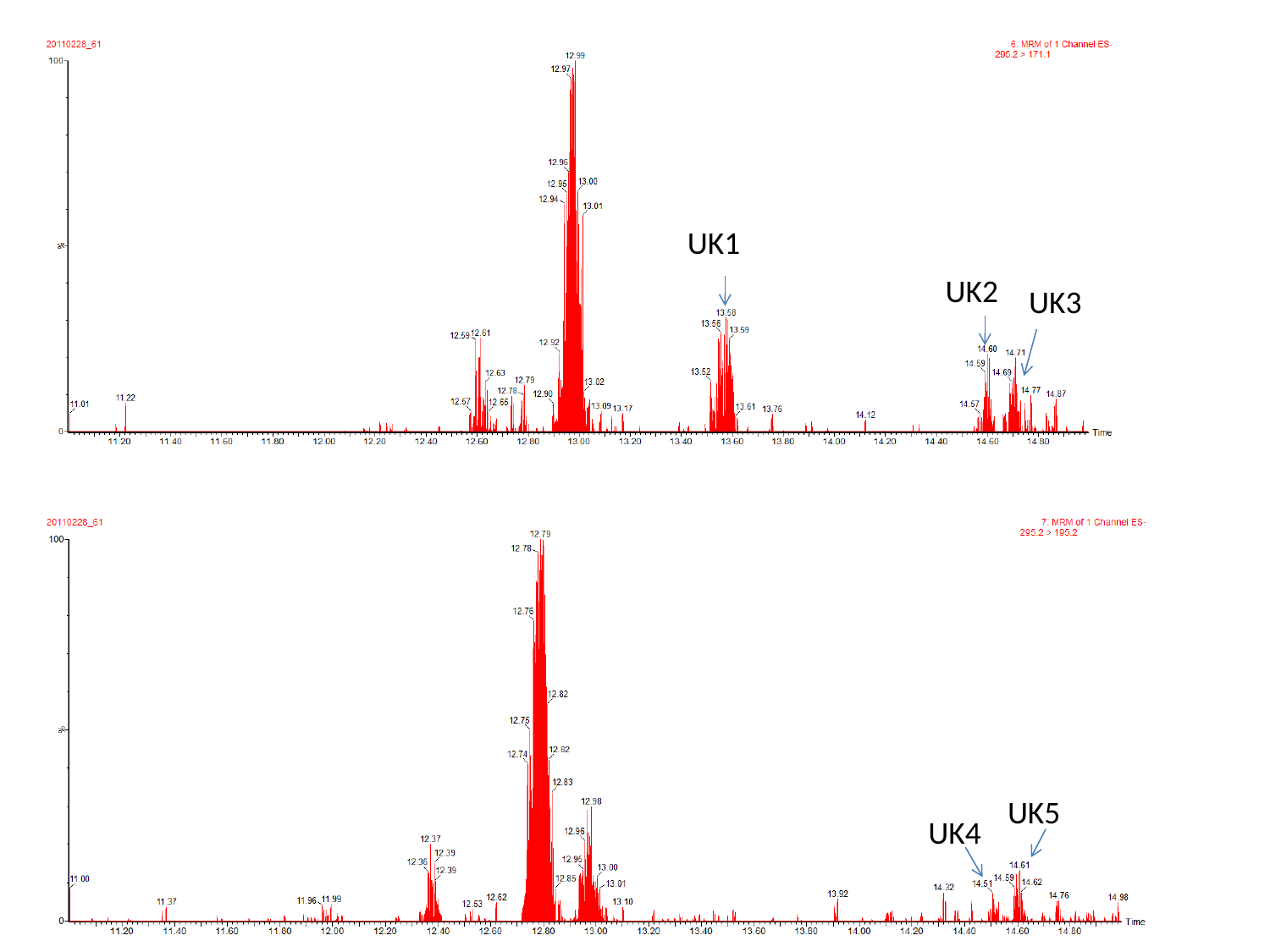

UK1
UK2
UK3
UK5
UK4

## Slide 6
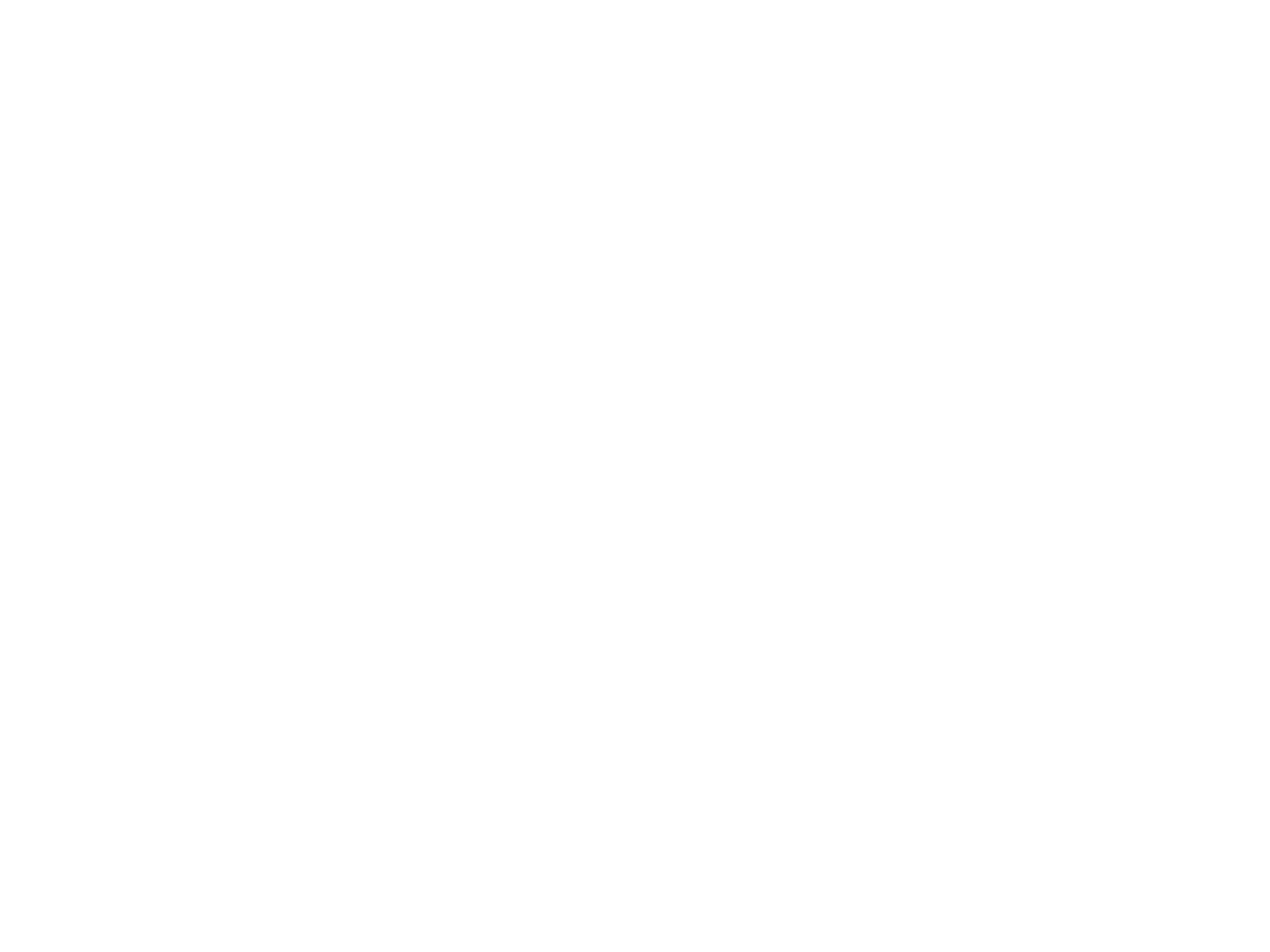

Supplement: Supplementary file 8 — Supplementary material 8 (PPTX 370 kb) [file 11306_2012_421_MOESM8_ESM.pptx]
